# Disentangling metabolic functions of bacteria in the honey bee gut

**DOI:** 10.1371/journal.pbio.2003467

**Published:** 2017-12-12

**Authors:** Lucie Kešnerová, Ruben A. T. Mars, Kirsten M. Ellegaard, Michaël Troilo, Uwe Sauer, Philipp Engel

**Affiliations:** 1 Department of Fundamental Microbiology, University of Lausanne, Lausanne, Switzerland; 2 Institute of Molecular Systems Biology, ETH Zürich, Zürich, Switzerland; Stanford University School of Medicine, United States of America

## Abstract

It is presently unclear how much individual community members contribute to the overall metabolic output of a gut microbiota. To address this question, we used the honey bee, which harbors a relatively simple and remarkably conserved gut microbiota with striking parallels to the mammalian system and importance for bee health. Using untargeted metabolomics, we profiled metabolic changes in gnotobiotic bees that were colonized with the complete microbiota reconstituted from cultured strains. We then determined the contribution of individual community members in mono-colonized bees and recapitulated our findings using in vitro cultures. Our results show that the honey bee gut microbiota utilizes a wide range of pollen-derived substrates, including flavonoids and outer pollen wall components, suggesting a key role for degradation of recalcitrant secondary plant metabolites and pollen digestion. In turn, multiple species were responsible for the accumulation of organic acids and aromatic compound degradation intermediates. Moreover, a specific gut symbiont, *Bifidobacterium asteroides*, stimulated the production of host hormones known to impact bee development. While we found evidence for cross-feeding interactions, approximately 80% of the identified metabolic changes were also observed in mono-colonized bees, with *Lactobacilli* being responsible for the largest share of the metabolic output. These results show that, despite prolonged evolutionary associations, honey bee gut bacteria can independently establish and metabolize a wide range of compounds in the gut. Our study reveals diverse bacterial functions that are likely to contribute to bee health and provide fundamental insights into how metabolic activities are partitioned within gut communities.

## Author summary

Honey bees are important pollinators that harbor a relatively simple gut microbiota with striking parallels to the mammalian system. This makes them relevant models to study gut microbiota functions and their impact on host health. We applied untargeted metabolomics to characterize metabolic changes induced by the gut microbiota and to characterize the contributions of the major community members. We find that the gut microbiota digests recalcitrant substrates derived from the pollen-based diet of bees. Most metabolic changes could be explained by the activity of individual community members, suggesting substrate specificity and independent metabolic functions. We did identify some cross-feeding interactions between species, including for pyruvate. Our study provides novel insights into the functional understanding of the bee gut microbiota and provides a framework for applying untargeted metabolomics to disentangle metabolic functions of gut bacteria.

## Introduction

Metabolic activities of the microbiota are key for symbiotic interactions in the gut and impact health and disease of the host in manifold ways. Gut bacteria facilitate the breakdown of refractory or toxic dietary compounds [1–3], produce metabolites that promote host growth and physiology [4–7], and modulate immune functions in the gut [8] and other tissues [9,10]. Moreover, metabolic activity is the basis for energy and biomass production, resulting in bacterial growth and the occupation of ecological niches conferring colonization resistance against pathogenic microbes [11]. Substrates of gut bacteria predominantly originate from the diet of the host [2,12], making diet the major modulator of the composition and metabolic activity of the gut microbiota [13,14].

The substantial metabolic potential of the animal gut microbiota has been profiled by the direct sequencing of functional gene content (i.e., shotgun metagenomics) [15–18]. However, it is challenging to predict functional metabolic output from such sequencing data. With recent advances in the coverage and throughput of untargeted screening metabolomics [19–21], it has become feasible to quantify metabolic changes in microbiota or host tissues at large coverage and throughput. Besides identifying metabolites connected to human health and disease [22–30], untargeted screening metabolomics holds considerable promise to unravel metabolic functions of individual microbiota members in animals with divergent dietary preferences. However, such mono-colonization studies are complicated by the highly variable and species-rich composition of most animal microbiota. Thus, gut communities of reduced complexity are valuable models to disentangle metabolic functions of the constituent species.

Like mammals, honey bees harbor a highly specialized gut microbiota. However, in contrast to mammals, the honey bee gut microbiota is surprisingly simple and consistent, with seven species (categorized by clustering at 97% sequence identity of the 16S rRNA) accounting on average for >90% of the entire gut community in bees sampled across continents [31]. This microbiota is composed of four Proteobacteria (*Gilliamella apicola*, *Snodgrassella alvi*, *Frischella perrara*, and *Bartonella apis*), which mostly reside in the ileum, and two Firmicutes (*Lactobacillus* spp. Firm-4 and Firm-5) and one Actinobacterium *(B*. *asteroides*), which are predominantly found in the rectum. These specific locations suggest that bacteria occupy different metabolic niches in the bee gut and potentially engage in syntrophic interactions [32,33].

The honey bee gut microbiota has marked effects on the host. It promotes host weight gain and hormone signaling under laboratory settings [34] and stimulates the immune system of the host [35,36]. In addition, honey bees are ecologically and economically essential pollinators that have experienced increased mortality in recent years [37,38], which could in part be due to disturbances of their microbiota composition [39–42].

Genomic analyses and in vitro experiments have shown that fermentation of sugars and complex carbohydrates (e.g., pectin) into organic acids [15,32,43,44] is a prominent metabolic activity of the gut microbiota [34]. Lacking, however, is a detailed understanding of the consumption of diet-derived substrates and how individual community members contribute to the metabolic activities in vivo. For instance, it is elusive whether analogously to mammals, recalcitrant dietary compounds (especially from pollen) are broken down by the microbiota in the hindgut (i.e., large intestine composed of ileum and rectum), while more accessible compounds are reportedly absorbed by the host in the midgut (i.e., small intestine) [45–47].

**Fig 1:**
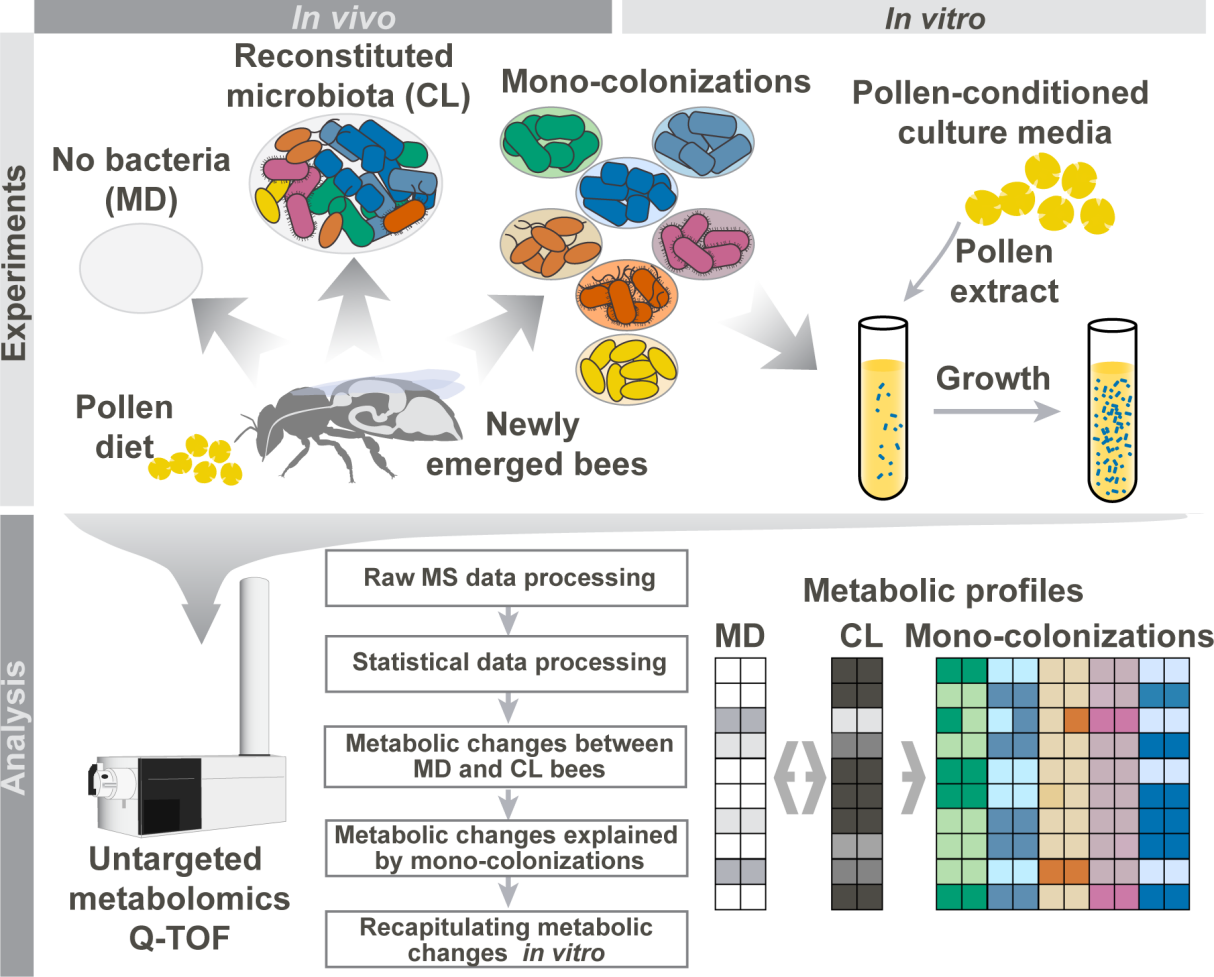
Overview of the experimental setup to characterize metabolic activities of the honey bee gut microbiota. Newly emerged adult bees were either kept microbiota-depleted (MD), colonized with a reconstituted community of the seven predominant species of the bee gut microbiota (CL), or mono-colonized with one of the seven species separately. Bees received sterilized bee pollen and sugar water as diet. Ten days after colonization, metabolites were extracted from the honey bee guts and subjected to untargeted metabolomics to (1) reveal overall metabolic changes in CL versus MD bees and (2) identify which community member could explain these metabolic changes in the gut. As a control, we additionally analyzed 10-d-old hive bees that were colonized by the native microbiota under natural conditions in the colony (not shown in this figure). To recapitulate findings in vitro, individual community members were cultured in pollen-conditioned medium, and metabolic changes in this medium were profiled using untargeted metabolomics. MS, mass spectrometry; Q-TOF, quadrupole-time of flight.

To profile the metabolic output of the honey bee gut microbiota and its individual members, we employed gnotobiotic bee colonizations and in vitro experiments in conjunction with untargeted metabolomics (Fig 1). We first characterized robust metabolic differences between microbiota-depleted bees and bees colonized with a reconstituted community composed of the seven major bacterial species of the gut microbiota. Subsequently, we analyzed bees colonized with each community member separately to assay their potential contribution to the overall metabolic output of the gut microbiota. Finally, we recapitulated our results in vitro using pollen-conditioned medium. Our systematic approach provides unprecedented insights into the metabolic activities of the honey bee gut microbiota and demonstrates the possibility to use metabolomics in combination with gnotobiotic animal models to disentangle functions of individual gut microbiota members.

## Results and discussion

### Experimental reconstitution of the honey bee gut microbiota

**Fig 2:**
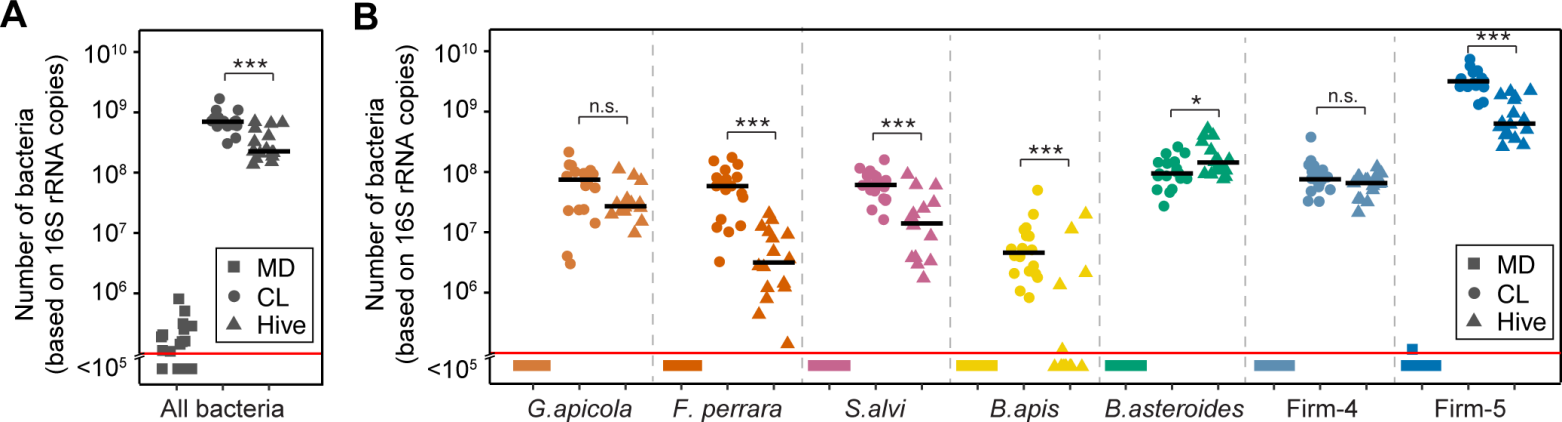
Bacterial colonization levels in the guts of microbiota-depleted (MD), colonized (CL), and hive bees. (A) Total bacterial loads in the gut of 10-d-old MD bees (*n* = 21), CL bees (*n* = 18), and hive bees (*n* = 16) were assessed by quantitative PCR (qPCR) with universal bacterial 16S rRNA primers. (B) The bacterial loads of the seven predominant community members used for experimental colonizations were assessed by qPCR with species-specific 16S rRNA primers for the same bees as shown in panel A. Black lines show median values. Samples with <10^5^ bacterial cells per gut are shown below the red line, which we consider the threshold of detection. Primer characteristics are summarized in S2 Table. n.s., not significant; *P < 0.05; **P < 0.01; and ***P < 0.001 (Wilcoxon Rank Sum test, Benjamini and Hochberg adjusted [BH adj.]). The numerical data can be found in S1 Data.

To characterize the metabolic output of the honey bee gut microbiota, we colonized newly emerged bees with selected bacterial strains previously isolated from the bee gut. The reconstituted bacterial community consisted of 11 strains (S1 Table) covering the seven predominant species of the bee gut microbiota described above. We used two strains for *G*. *apicola* and four strains for Firm-5 in order to cover the extensive genetic diversity within these species [44,48]. Exposure of newly emerged adult bees to this community resulted in the successful establishment of all seven species, with a total of approximately 10^9^ bacterial cells per gut after 10 d of colonization; hereafter, these are referred to as CL bees (Fig 2A). In contrast, non-colonized bees had total bacterial loads of <10^6^ cells per gut, an observation consistent with previous studies [32,49]. In the following, we will refer to these bees as microbiota-depleted (MD) because they were not colonized with detectable levels of typical honey bee gut bacteria as determined with species-specific qPCR primers (<10^5^ bacterial cells, except for one bee that was slightly above this cut-off for Firm-5) (Fig 2B). However, these bees may have harbored low levels of environmental microbes, as they were not kept under sterile conditions, especially in cases in which the bacterial loads were slightly above our detection limit of 10^5^ bacterial cells (Fig 2A). It is also important to point out that newly emerged bees can occasionally be contaminated with specific bee gut bacteria, resulting in “MD” bees that in fact are colonized. Therefore, to be able to exclude such bees from further analysis, it is essential to determine the microbiota status of gnotobiotic bees using the qPCR assays presented in this study or an equivalent method.

Compared to hive bees of the same age, bacterial abundances of most species were slightly elevated in CL bees. However, in both groups the Firm-5 species was consistently the most abundant community member, while *B*. *apis* colonized at relatively low levels. This is in line with recent 16S rRNA gene-based community analyses [31,50,51], and we thus conclude that the selected strains assembled into a structured community resembling the native honey bee gut microbiota. Overall, this analysis validates our gnotobiotic bee system as a tool for microbiota reconstitution experiments and enables the study of microbiota functions under controlled laboratory conditions.

### Metabolic changes in the honey bee gut upon colonization with the reconstituted bacterial community

To reveal microbiota-induced metabolome changes in the gut, we dissected the combined mid- and hindgut of MD, CL, and hive bees and analyzed water-extracted homogenates of these gut samples by untargeted metabolomics [21]. In total, we detected 24,899 mass-to-charge features (ions), 1,079 of which could be annotated by matching their accurate mass-to-sum formulas of compounds in the full Kyoto Encyclopedia of Genes and Genomes (KEGG) database (S2A Data). These 1,079 ions putatively correspond to 3,270 metabolites (S2B Data), since this method cannot separate isobaric compounds. For statistical analysis, we continued with the annotated ions, and for ion changes with multiple annotations, we provided the most likely annotation based on information from literature and genomic data.

**Fig 3:**
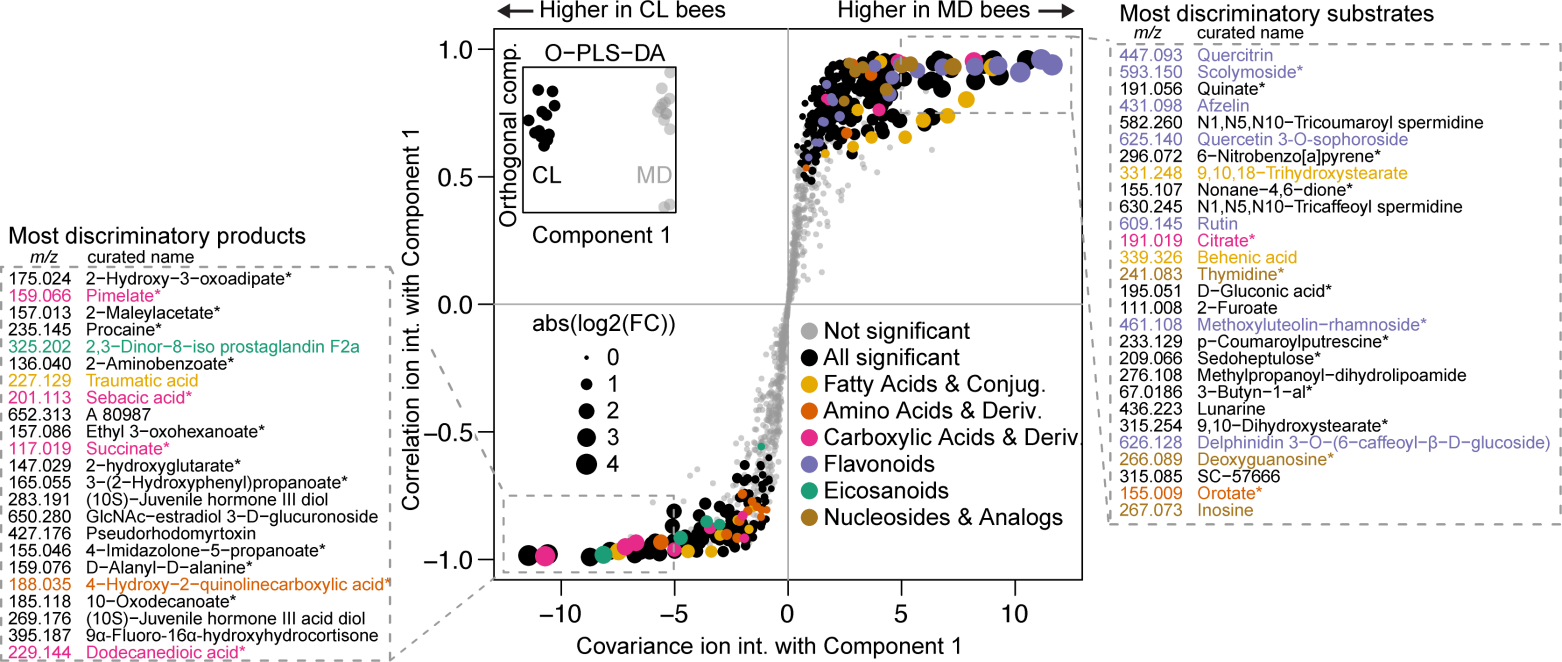
Metabolite changes between microbiota-depleted (MD) and colonized (CL) bees. An Orthogonal Projection of Least Squares-Differentiation Analysis (OPLS-DA) based S-plot of metabolite changes shows the ions responsible for CL and MD separation. The inset shows OPLS-DA separation between CL and MD along the component that was used for correlating ion intensities. Experiment 2 data (see S2A Data) was used for this plot, and annotated ions that were not robustly significantly different between CL and MD in both experiments are plotted in grey. Ions with a first annotation belonging to an enriched category (see S3A Data) are plotted in color, except for the category “amino acids and derivatives”, which did not meet the significance threshold for enrichment but was deemed relevant. The “purine nucleosides and analogues” and “pyrimidine nucleosides and analogues” categories were combined into “nucleosides and analogs” for coloring only. The boxed areas show the *m/z* [M-H^+^]^-^ of the ion and the first annotation name of the most discriminatory ions, sorted by covariance. Asterisks indicate ions with ambiguous annotations. The numerical data can be found in S1 Data. Conjug., conjugates; Deriv., derivatives; FC, fold change; int., intensity.

Principal component analysis on the ion intensities revealed that CL and MD bees separate into two distinct clusters, which suggests colonization-specific metabolic profiles (S1 Fig). In two independent experiments, a total of 372 ions exhibited significant changes between CL and MD bees (Welch’s *t* test, Benjamini and Hochberg adjusted [BH adj.] *P* ≤ 0.01, S2C Data). A subset of 240 ions (65%) were more abundant in MD bees, suggesting that the cognate metabolites are utilized by the gut microbiota. These ions are hereafter referred to as bacterial substrates. Conversely, 132 ions were more abundant in CL bees and are hereafter referred to as bacterial products, indicating that they are produced either by the microbiota or by the host in response to the microbiota. To facilitate the biological interpretation of these multitude metabolic changes, we carried out two analyses. First, we looked at whether certain compound classes were overrepresented among the subsets of bacterial substrates and products (S3A Data). Second, we sorted ion changes based on their ability to explain the difference between the CL and MD metabolome profiles in an Orthogonal Projection of Least Squares-Differentiation Analysis (OPLS-DA) (Fig 3) [52].

### Major bee gut microbiota substrate classes

We first focused on the 240 substrate ions that were more abundant in MD versus CL bees and potentially correspond to metabolites utilized by the microbiota (S2C Data). We found 3 compound classes to be strongly enriched: “flavonoid*s*” (20 of 36 annotated ions, one-sided Fisher’s exact test, *P* < 0.001) and both “purine nucleosides and analogues” and “pyrimidine nucleosides and analogues” (in total eight of nine annotated ions, both *P* < 0.01). Seven flavonoids, three nucleosides, and a nucleoside precursor (orotate, *m/z* 155.009) were also among the 28 substrate ions with the most discriminatory power for distinguishing CL versus MD bees as based on OPLS-DA (Fig 3). Other ions among these most discriminatory substrates included two ω-hydroxy acids (*m/z* 315.254 and *m/z* 331.248) and three phenolamides (*m/z* 582.260, *m/z* 630.245, and *m/z* 233.129) from the outer pollen wall, as well as quinate (*m/z* 191.056) and citrate (*m/z* 191.019), both of which had previously been predicted to be utilized by certain community members of the honey bee gut microbiota [53,54]. Because they are the most remarkable groups among the identified substrates, nucleosides, flavonoids, and pollen wall-specific compounds will be discussed in more detail.

#### Nucleosides

Nucleosides are essential biomass components that can also serve as bacterial energy sources. The majority of the honey bee gut microbiota members lack genetic capacity for either purine or pyrimidine biosynthesis [33]. Instead, they seem to rely on salvage pathways and the uptake of nucleosides from the environment, providing a likely explanation for the observed depletion of these compounds in the gut of CL bees (S2A Fig). However, we cannot exclude an effect of altered host nucleoside metabolism on these changes, especially as nucleoside transporters are present in the intestinal epithelial cells of animals [55].

#### Flavonoids

Flavonoids are secondary metabolites that can make up 2%–4% of the dry weight of pollen [56,57] and this is in concordance with their identification as important diet-derived substrates of the honey bee gut microbiota. Flavonoids typically consist of two phenyl rings and one heterocyclic ring, the so-called aglycone. Glycosylation of this aglycone in diverse positions generates the remarkable flavonoid structural and functional diversity [58]. Among the bacterial substrates, we identified several glycosylated flavonoids (S2B Fig) that are known to be present in pollen, such as rutin or quercitrin [56]. We unambiguously confirmed the identity of three of these ions as the glycosylated flavonoids afzelin, quercitrin, and rutin using tandem mass spectrometry (MS/MS) fragmentation and spectral similarity calculations (see S1 Text, S3 Fig and S4 Data).

Several mammalian gut bacteria can convert flavonoids by either deglycosylation, thus releasing flavonoid aglycones, or C-ring cleavage, resulting in the accumulation of breakdown products of the aromatic backbone [59]. We mined the 132 bacterial products to look for these signatures of flavonoid conversions in the bee gut and identified several ions annotated as non-glycosylated flavonoids (S2B Fig). However, none of them significantly accumulated in CL versus MD bees, which would have been expected when deglycosylation was the only mechanism of flavonoid conversion. In contrast, we identified four ions among the bacterial products that could result from biodegradation of aromatic amino acids and flavonoid aglycones: two ions annotated as hydroxy- and dihydroxyphenylpropionate (*m/z* 165.055 and *m/z* 181.050), both of which are known C-ring cleavage products of flavonoids, and two ions annotated as maleylacetate (*m/z* 157.013) and hydroxy-3-oxoadipate (*m/z* 175.024), which are intermediates of aromatic compound degradation pathways (S2B Fig) [60–63]. Strikingly, three of these four ions were among the most discriminatory products for CL versus MD bees (Fig 3). Moreover, in a recent metabolomics study by Zheng et al. [34], similar intermediates were shown to accumulate in the hindgut of colonized bees (S2 Text and S5 Data).

#### Pollen wall compounds

The two ω-hydroxy acid ions (9,10,18-trihydroxystearate, *m/z* 315.254, and 9,10-dihydroxystearate, *m/z* 331.248) that were identified among the most discriminatory substrate ions (Fig 3 and S2C Fig) have been reported to be major constituents of sporopollenin [64], the biochemically inert and heterogeneous biopolymer forming the rigid structure of the outer pollen wall, the exine [65]. Phenolamides such as those utilized by the gut microbiota (N1,N5,N10-tricoumaroyl spermidine, *m/z* 582.260; N1,N5,N10-tricaffeoyl spermidine, *m/z* 630.245; and p-coumaroylputrescine, *m/z* 233.129; Fig 3 and S2C Fig) are also compounds of the exine as they are deposited on top and into its cavities as part of the pollen coat [66]. Remarkably, the pollen coat is also where most flavonoids are thought to be located in pollen grains [67].

Our findings on the utilization of flavonoids, ω-hydroxy acids, and phenolamides thus suggest that the honey bee gut microbiota contributes to the digestion of the rigid outer pollen wall. Easily accessible pollen nutrients (such as amino acids, sugars, and vitamins) are likely taken up by the host in the midgut, leaving these more recalcitrant compounds for the microbiota in the hindgut. This is in line with what is known about the biogeography and microbial ecology in the mammalian intestine [68]. Besides being utilized as an energy and carbon source, the conversion of secondary plant metabolites from pollen may have additional benefits for the microbiota and the host. For example, phenolamides and flavonoids both have been reported to exert antimicrobial activities, conceivably because their breakdown products increase the antioxidant potential in the gut, which could reduce inflammation and pathogen susceptibility [66,69]. In addition, mammalian flavonoid-metabolizing bacteria have a major impact on the bioavailability of flavonoids, and flavonoids are implicated in modulating weight gain by affecting host signaling [70,71]. This makes it tempting to speculate that flavonoid metabolism in the bee gut contributes to the microbiota dependent weight gain of honey bees that was observed in a previous study [34].

### Products of the gut microbiota include fermentation products and host-derived metabolites

We next looked into the 132 ions that were more abundant in CL versus MD bees and thus represent possible metabolites produced by the microbiota (S2C Data). Again, we used enrichment analyses and OPLS-DA (Fig 3) to prioritize the most important product ions. Three compound classes were to some extent enriched among the bacterial products (S3A Data): “carboxylic acids and derivatives” (seven of 26 annotated ions, one-sided Fisher’s exact test, *P* < 0.03), “fatty acids and conjugates” (seven of 29 annotated ions *P* < 0.05), and “eicosanoids” (five of eight annotated ions, *P* < 0.01).

#### Fermentation products

Both the “carboxylic acids and derivatives” and “fatty acids and conjugates” categories contain known bacterial fermentation products, several of which accumulated in CL bees (succinate, *m/z* 117.019; pimelate, *m/z* 159.066; sebacic acid, *m/z* 201.113; butyrate, *m/z* 87.044; and valerate, *m/z* 101.060). This is in agreement with previous studies suggesting that fermentation is the predominant metabolic activity of bee gut bacteria [33,34]. Three of these fermentation products (succinate, pimelate, and sebacic acid) were among the 23 most discriminatory products, which highlights the substantial and consistent accumulation of these compounds in the presence of the microbiota (Fig 3 and S2D Fig). Using targeted metabolomics [72], we confirmed the strong accumulation of succinate in the gut of CL bees and determined absolute concentrations of other organic acids in CL and MD bees (S3 Text, S4 Fig and S6 Data). In addition, accumulation of fermentation products is one of the main microbiota dependent trends found in our study and that of Zheng et al. [34] (for details on the comparison, see S2 Text).

#### Host-derived metabolites

The third enriched product category (“eicosanoids”) includes five ions whose masses match to prostaglandins (S2E Fig), which are broadly conserved hormone-like lipids in animals. In insects, prostaglandins have been implicated in reproduction, fluid secretion, and activation of the immune system [73] through induction of prophenoloxidase, phagocytosis, and hemocyte spreading. None of the five prostaglandins annotated in our study have been functionally characterized in honey bees.

Besides eicosanoids, we identified a second group of host-derived metabolites induced by the microbiota. These are three derivatives of juvenile hormone III (S2E Fig), two of which were among the most discriminatory product ions (Fig 3, *m/z* 283.191 and *m/z* 269.176). Juvenile hormone III plays an important role in regulating the growth, development, and reproduction of insects. In adult honey bees, it controls the pace of the developmental maturation from young nurse bees to older forager bees [74]. This process is linked to nutrition [75] and could therefore be affected by metabolic activities of gut bacteria. Notably, juvenile hormone derivatives in the gut may have local functions distinct from those in the brain or hemolymph, as was shown for heteropteran linden bugs [76].

### Gut metabolic profiles of colonized bees and hive bees show substantial overlap

To assess how much of the total metabolic output can be identified in hive bees under natural conditions, we analyzed the gut metabolome of 10-d-old hive bees that were exposed to social interactions and natural dietary resources and were colonized by the native gut microbiota. Principal component analysis revealed that hive bees clustered separately from CL bees (S1 Fig). This may be explained in part by the different diet of hive bees, the presence of multiple strains in a bee colony, and the impact of the environment on the gut metabolism. However, we found that 27 of the 28 most discriminatory substrate ions and 15 of the 22 most discriminatory product ions showed qualitatively the same changes in hive bees as in CL bees (S2D Data, Welch’s *t* test, BH adj. *P* ≤ 0.01). On the substrate side, this included most flavonoid ions, all nucleosides, quinate, and citrate, as well as the ions annotated as ω-hydroxy acids and phenolamides from the outer pollen wall. On the product side, we found four of the five prostaglandins and one of the juvenile hormone derivatives to be significantly increased in hive bees relative to MD bees, suggesting that these host-derived metabolites are also induced under natural conditions. Moreover, ions corresponding to fermentation products were either significantly increased (sebacic acid and valerate) or showed a trend towards increased levels (succinate and pimelate) in hive bees. The same was the case for the four ions corresponding to possible degradation products of flavonoids (hydroxy- and dihydroxyphenylpropionate, maleylacetate, and hydroxy-3-oxoadipate; S2A Data). We conclude that the remarkable overlap of metabolic changes between hive bees and CL bees highlights the relevance of our findings.

### Mono-colonizations explain 80% of the overall metabolic output of the honey bee gut microbiota

We thus far presented evidence for substrates and products of the complete microbiota in the honey bee gut. To elucidate which community members might be responsible for these transformations, we conducted mono-colonizations of MD bees with all seven bacterial species (again using a mix of four and two strains together for Firm-5 and *G*. *apicola*, respectively). All species successfully established in the gut of MD bees, with other community members being generally below the limit of detection (<10^5^ bacterial cells) (S5 Fig). We again extracted metabolites from the mid- and hindgut of individual bees to address how many of the 372 robust ion changes can be explained by one or multiple mono-colonizations (S2A Data), i.e., show qualitatively the same change as in CL bees (analysis of variance [ANOVA] followed by Tukey honest significant difference [HSD] post hoc test at 99% confidence, *P* ≤ 0.05) (S7 Data and S8 Data). Extended results of this analysis can be found in S2A Data.

**Fig 4:**
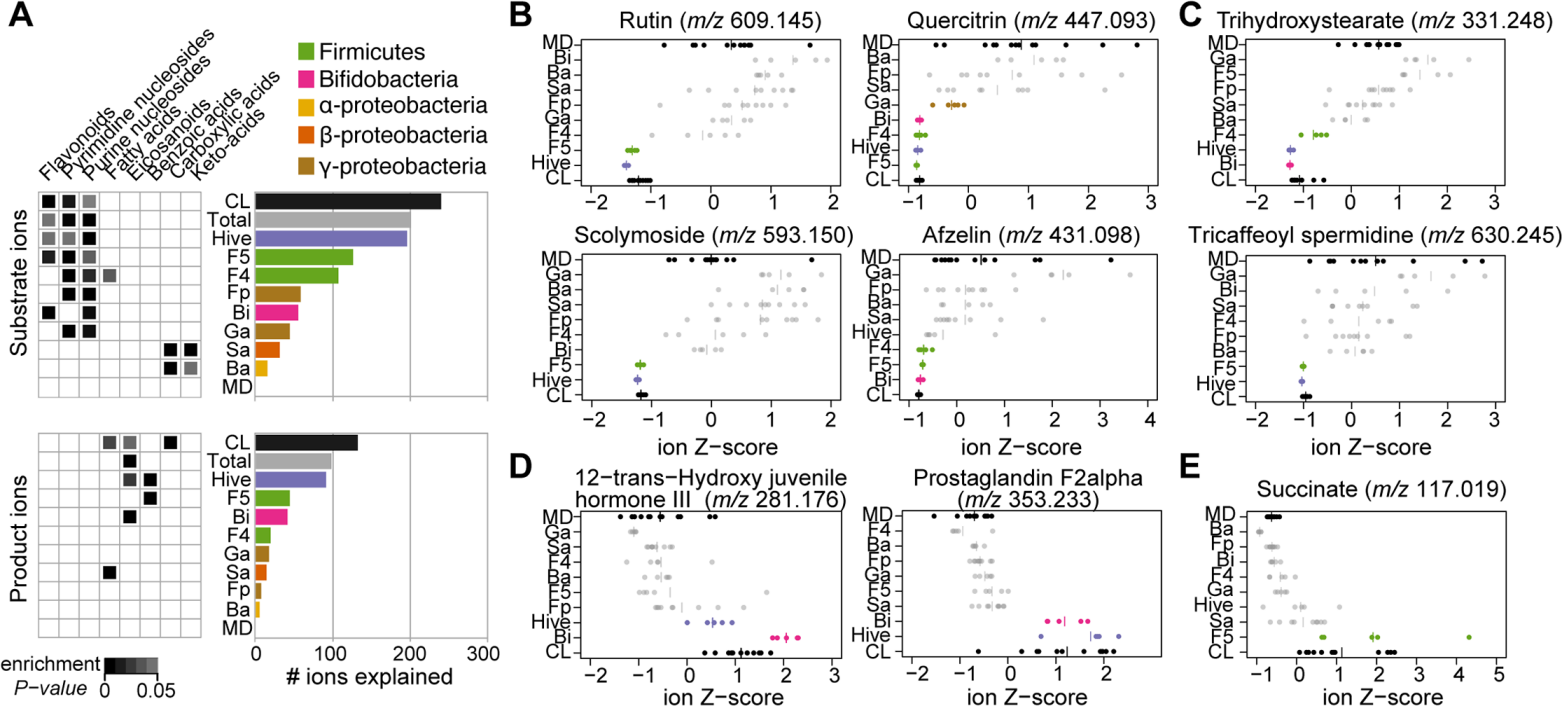
Overview of metabolite changes explained by different community members of the bee gut microbiota. (A) Bar graphs show the fraction of the metabolic changes explained by mono-colonizations and hive bees for substrates (240 ions) and products (132 ions). The category “Total” indicates the total number of ions explained by mono-colonizations, thus excluding hive bees. Heatmap representation of enrichment *P* values (one-sided Fisher’s exact test *P* < 0.05) are provided for compound categories enriched in one or several mono-colonizations. (B–E) Z-score transformed ion intensities of selected substrate and product ions are shown for all treatment groups. (B) Four glycosylated flavonoid substrates. (C) Two substrates from the outer pollen wall. (D) Two products corresponding to host-derived metabolites. (E) Succinate, one of the major fermentation products. Groups depicted in color highlight treatment groups displaying a significant difference compared to MD bees in the same direction as the CL versus MD difference (one-way analysis of variance [ANOVA], Tukey honest significant difference [HSD] post hoc test at 99% confidence, *P* ≤ 0.05). Plots for all 372 ions are provided in S8 Data. Ba, *B*. *apis* mono-colonized; Bi, *B*. *asteroides* mono-colonized; CL, colonized with the reconstituted microbiota; F4, Firm-4 mono-colonized; F5, Firm-5 mono-colonized; Fp, *F*. *perrara* mono-colonized; Ga, *G*. *apicola* mono-colonized; Hive, hive bees; MD, microbiota-depleted; Sa, *S*. *alvi* mono-colonized. The numerical results of the full enrichment analysis, bar graphs, and mono-colonization plots are provided in S3 Data, S1 Data and S8 Data, respectively.

Remarkably, using these significance cutoffs, 299 of the 372 (80%) robust changes between MD and CL bees could be explained by one or multiple mono-colonizations. This included 201 (84%) substrate and 98 (74%) product ions. The two *Lactobacilli* species (Firm-5 and Firm-4) explained most changes, followed by *B*. *asteroides* and the two Gammaproteobacteria (Fig 4A). Interestingly, the relative contribution to substrate conversion and product accumulation varied between mono-colonizations. For example, *B*. *asteroides* contributed relatively little to the conversion of substrates but seemed to be responsible for the production of a relatively large fraction of bacterial products. The Firm-4 species showed the opposite pattern, explaining relatively many bacterial substrates but a small fraction of bacterial products. Ion changes identified in CL bees but not in any of the mono-colonizations (in total 20%*)* may be due to our strict significance cutoffs, additive metabolic activities, or concerted functions of the community members, such as cross-feeding or interspecies metabolic feedback.

#### Substrates explained by mono-colonizations

We again used enrichment analysis (one-sided Fisher’s exact test, *P* ≤ 0.05), to get a high-level view of the functions of distinct community members in the conversion or production of certain compound classes (Fig 4A). This analysis revealed that all community members, except for *S*. *alvi* and *B*. *apis*, contributed to the disappearance of nucleosides in the bee gut (see also S2A Fig). Remarkably, *S*. *alvi* and *B*. *apis* encode complete nucleoside biosynthesis pathways and therefore do not have to rely on external nucleoside resources [32,54]. In turn, they seem to preferentially convert carboxylic acids and keto acids in the gut (malate, *m/z* 133.013; fumarate, *m/z* 115.003; citrate, *m/z* 191.019; and α-ketoglutarate, *m/z* 145.014), which is consistent with the presence of complete tricarboxylic acid (TCA) cycles and several carboxylate transporters in the genomes of these bacteria [32,54]. Flavonoids were enriched substrates for Firm-5 (11 of 126 substrate ions, *P* < 0.01) and *B*. *asteroides* (six of 55 substrate ions, *P* < 0.01), and many flavonoids were also utilized by Firm-4 (seven of 107 substrate ions, *P* = 0.051) (Fig 4A and S2B Fig). Interestingly, rutin (*m/z* 609.145) and scolymoside (*m/z* 593.150) were exclusively depleted in the Firm-5 mono-colonization, while afzelin (*m/z* 431.098) was also utilized by Firm-4 and *B*. *asteroides*, and quercitrin (*m/z* 447.093) even by *G*. *apicola* (Fig 4B). Similar patterns were also found for other flavonoids (S2B Fig), suggesting substrate specificity for the utilization of these pollen-derived compounds among community members.

In total, 27 of the 28 most discriminatory substrates (Fig 3) could be explained by at least one mono-colonization. The two ω-hydroxy acids ions from the outer pollen wall were exclusively utilized by *B*. *asteroides* and Firm-4, while the three phenolamides from the pollen coat were only depleted in the presence of Firm-5 (Fig 4C and S2C Fig). In contrast, quinate and citrate were utilized by several community members, suggesting that their almost complete depletion in CL and hive bees could be the result of a communal effort (S2A Data).

#### Products explained by mono-colonizations

For bacterial products, 21 of the 23 most discriminatory ions for CL versus MD bees could be explained by at least one mono-colonization. Strikingly, *B*. *asteroides* explained the accumulation of all host-derived prostaglandins and was also responsible for the induction of two of the three juvenile hormone derivatives, suggesting that this community member has a distinct influence on the host (Fig 4D and S2E Fig). Notably, *S*. *alvi*, Firm-4, and Firm-5 also affected the production of some of these metabolites, but not to the same extent as *B*. *asteroides*.

Three major fermentation products that accumulated in CL bees could be explained by mono-colonizations. Succinate (Fig 3E) and pimelate ions were produced exclusively in bees colonized with Firm-5, and valerate only in the *B*. *asteroides* colonized bees (S2D Fig and S2A Data). Finally, we found that ions corresponding to aromatic compound degradation intermediates accumulated in bees colonized with Firm-4 and Firm-5 (S2B Fig), which also explained most flavonoid utilization of the bee gut microbiota. This suggests that Firm-4 and Firm-5 do not only deglycosylate flavonoids, conceivably through expression of glycoside hydrolases [44], but may additionally degrade the aromatic backbone.

### In vitro recapitulation of metabolic functions of community members

**Fig 5:**
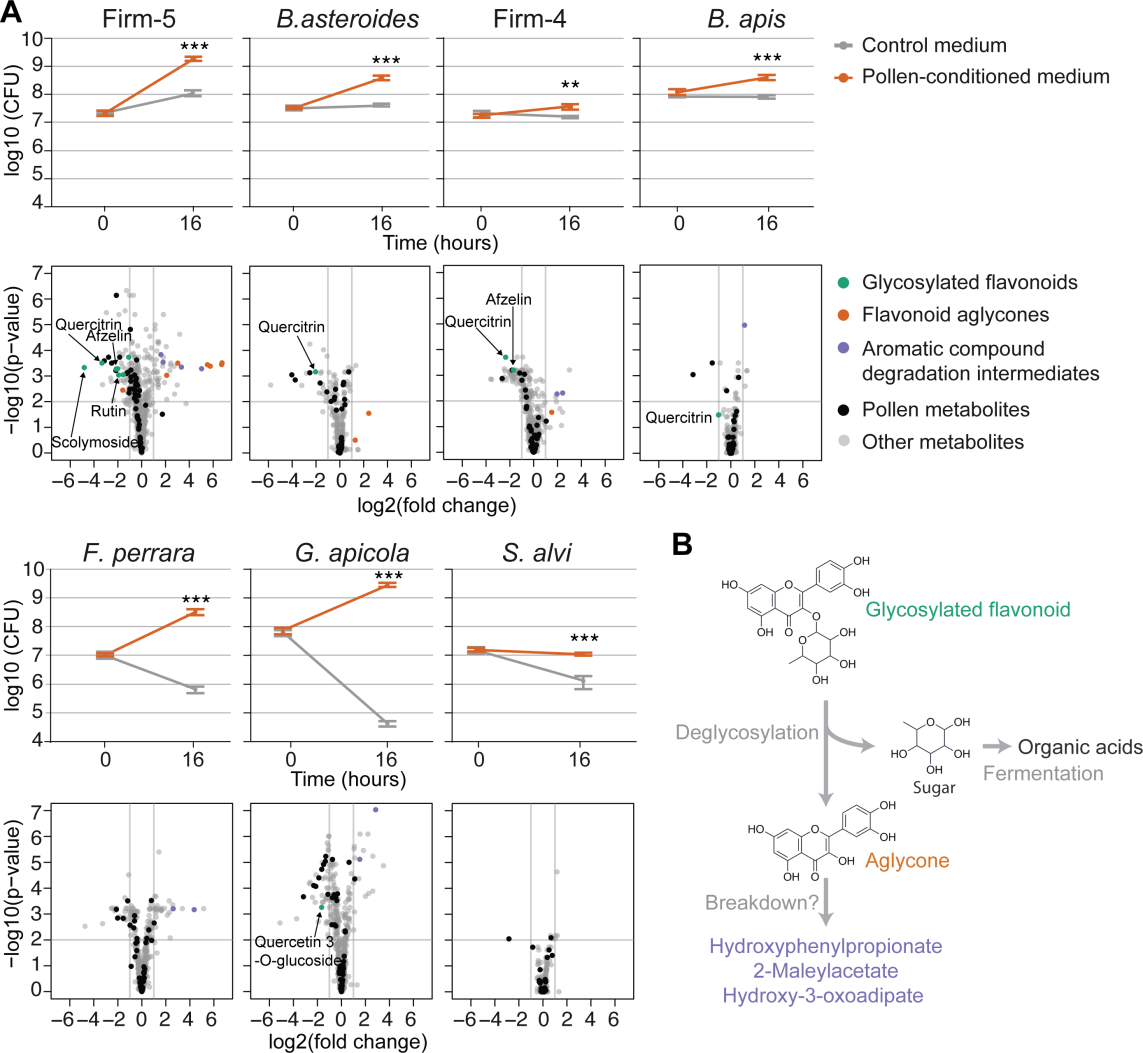
Recapitulation of flavonoid degradation patterns by gut bacteria during in vitro growth in pollen-conditioned medium. (A) Line graphs show the growth of each community member in control medium and pollen-conditioned medium based on colony-forming unit (CFU) counts at time points 0 h and 16 h. Values are the mean of five replicates, with error bars indicating standard deviation. **P < 0.01, ***P < 0.001 (Welch’s *t* test). Volcano plots of significance (Welch’s *t* test Benjamini and Hochberg adjusted [BH adj.] *P* value) versus log2(fold change) show metabolic changes in pollen-conditioned medium at time point 16 h relative to 0 h. Ions identified as pollen derived are highlighted in black. Ions annotated as glycosylated flavonoids, flavonoid aglycones (non-glycosylated flavonoids), or putative flavonoid breakdown products are shown in color when they displayed log2(fold changes) ≥ |1|. Other annotated ions are plotted in grey. (B) Model for the metabolism of flavonoids in the bee gut. Flavonoids are deglycosylated by specific bee gut bacteria, resulting in the release of flavonoid aglycones. The sugar residues are likely fermented into organic acids. Accumulation of several intermediates of aromatic compound degradation pathways, both in vivo and in vitro, suggests that the aglycone may be broken down further. The numerical values of the line graphs and the volcano plots can be found in S1 Data and S9 Data, respectively.

Our in vivo results strongly suggest that specific gut bacteria utilize distinct substrates from the pollen diet of bees. This prompted us to test (1) whether the bacterial species could grow in vitro on a pollen-based culture medium and (2) whether this would result in the metabolic conversions of the same compounds as was observed in vivo. To this end, we water-extracted metabolites from the same pollen batch that was used for feeding the bees and analyzed the metabolic composition of this extract using untargeted and targeted metabolomics. Detailed results are presented in S4 Text and show that pollen extract contains physiologically meaningful levels of nutrients and is expectedly enriched in “amino acids and derivatives,” “flavonoids,” “monosaccharides,” and “carboxylic acids and derivatives” (S6 Fig). Strikingly, all community members, except for *S*. *alvi*, showed substantial growth in the presence of this pollen extract compared to the nutrient-limited base media in which little or no growth was observed after 16 h of incubation (Fig 5A).

We then profiled the metabolome of growth media before and after bacterial incubation in a separate metabolomics experiment. We annotated a total of 1,031 ions (S9 Data), of which 427 (41%) were also present among the 1,079 ions from the in vivo dataset. In line with their growth profiles, the largest number of depleted metabolites (log2(FC) ≥ |1| and Welch’s *t* test BH adj. *P* ≤ 0.01) was found for the growth cultures of Firm-5, followed by *G*. *apicola*, Firm-4, *F*. *perrara*, *B*. *asteroides*, *B*. *apis*, and *S*. *alvi* (Fig 5A).

Using strict criteria, we identified 17 ions (13 pollen-derived substrates and four bacterial products), which were explained in vivo and in vitro by the same species (S7 Fig and S3 Table). Seven of these 13 substrates belonged to the most discriminatory substrate ions for CL versus MD bees (Fig 3): three flavonoids (quercitrin, afzelin, and rutin), one nucleoside (inosine), and ions annotated as quinate, citrate, and 2-fuorate. The fact that different community members were responsible for the conversion of some of these substrates (*B*. *asteroides*, Firm-4, Firm-5, *F*. *perrara*, *B*. *apis*, and *G*. *apicola*) demonstrates that our in vitro cultures allowed us to recapitulate metabolic activities covering the entire community.

We found remarkably overlapping substrate specificity for four flavonoids in vitro and in vivo, with the Firm-5 species being the only member capable of converting rutin and scolymoside, while quercitrin and afzelin were also utilized by Firm-4, and quercitrin was additionally used by *B*. *asteroides* and *B*. *apis* (Fig 5A). Among the four in vitro recapitulated products were three of the four ions corresponding to putative breakdown products of flavonoids (S7 Fig and S3 Table). These ions accumulated in vivo and in vitro in the presence of Firm-4 and/or Firm-5, providing further evidence for breakdown of the polyphenolic ring structure of flavonoids. However, we also found that deglycosylated flavonoids (i.e., aglycones) significantly accumulated in cultures of Firm-5 and showed a trend towards accumulation for Firm-4 and *B*. *asteroides* (Fig 5A). Based on these results, we propose that flavonoid degradation involves two steps (Fig 5B): (1) deglycosylation of sugar residues and their subsequent fermentation and (2) the breakdown of the polyphenol backbone. The second step could be relatively slow, explaining why aglycones accumulated in vitro (16 h), but not in vivo (10 d). Alternatively, the accumulation of some of these aromatic compound degradation intermediates could also result from the metabolism of other substrates such as aromatic amino acids.

An obvious difference in our in vitro experiments compared to the in vivo situation is the absence of the host, which may predigest pollen grains before gut bacteria utilize pollen-derived metabolites. For example, certain sugars and amino acids are expected to be present in lower amounts in vivo because of host absorption. Conversely, the host may also provide physicochemical conditions that support the growth of some community members. This could explain the poor growth of *S*. *alvi* in vitro, especially as in vivo *S*. *alvi* is tightly associated with the gut epithelium and other gut bacteria such as *G*. *apicola* [77].

### Evidence for cross-feeding in the bee gut microbiota

Microbial species in gut communities can organize into food chains, where one species provides metabolites that can be utilized by others. Such metabolites may be released from insoluble dietary particles via bacterial degradation or can be generated as waste products of metabolism [2]. To identify possible metabolic interactions between community members of the bee gut microbiota, we focused on ions that in vivo significantly accumulated in some mono-colonizations and were depleted in others (S2A Data). A total of 27 ions showed such opposing changes between two or several mono-colonizations (S4 Table).

An example of a potential metabolic interaction identified in our dataset is the liberation and consumption of one of the major bacterial substrates in CL bees, 9-10-18-trihydroxystearate (*m/z* 331.248), originating from the outer pollen wall. In our mono-colonization experiments, the corresponding ion was depleted in Firm-4 and *B*. *asteroides* but accumulated in the case of Firm-5 and *G*. *apicola* (Fig 4C). This suggests that the latter two species facilitate the release of this ω-hydroxy acid from the outer pollen wall, possibly rendering it more accessible for further degradation by Firm-4 and *B*. *asteroides*.

**Fig 6:**
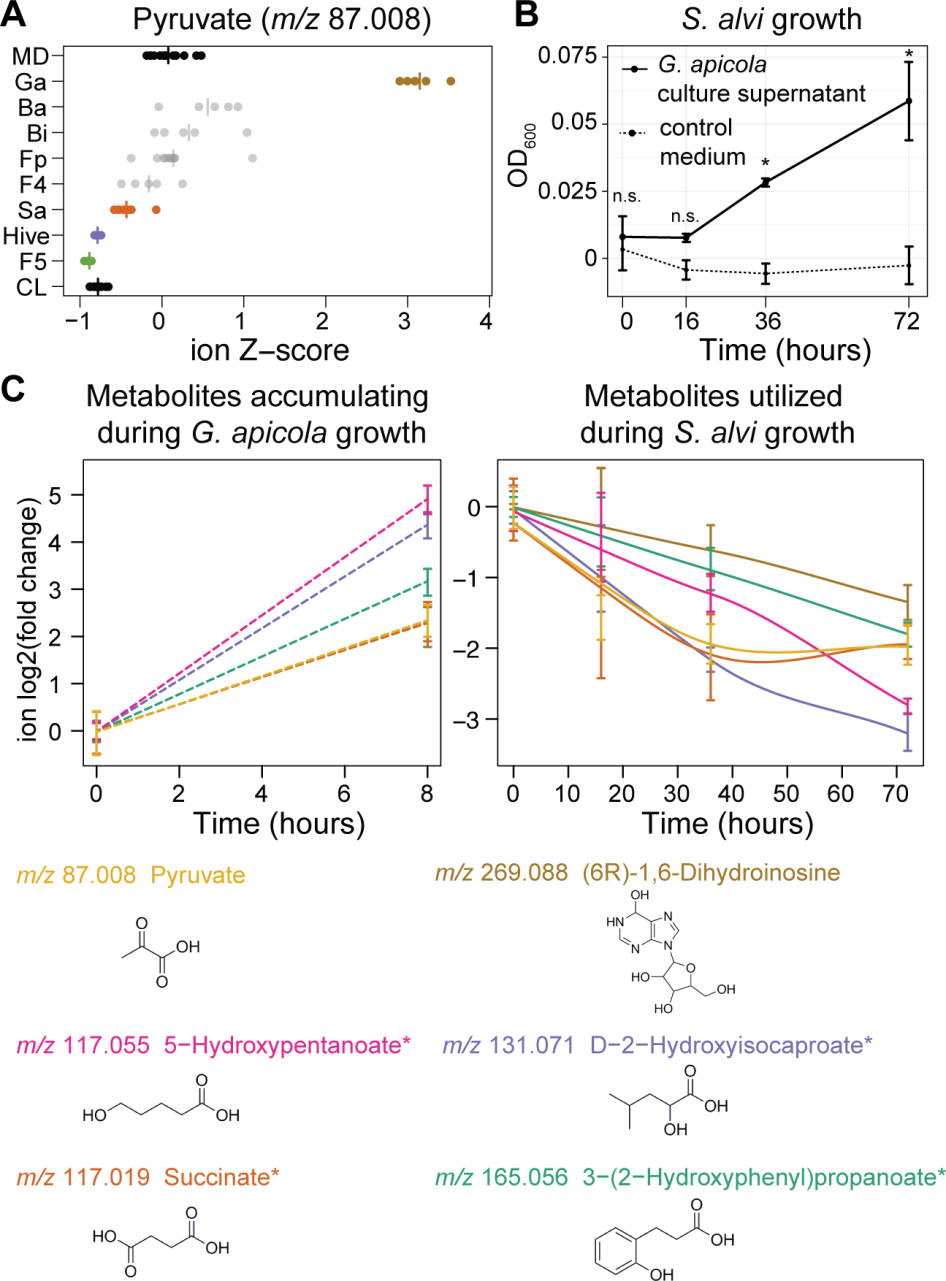
Cross-feeding between *G*. *apicola* and *S*. *alvi*. (A) Evidence for cross-feeding of pyruvate in the honey bee gut. Z-score transformed ion intensities revealed that the ion annotated as pyruvate accumulated in bees mono-colonized with *G*. *apicola* but was depleted in hive bees, CL bees, and bees mono-colonized with *S*. *alvi* and Firm-5. (B) Growth improvement of *S*. *alvi* in *G*. *apicola-*conditioned medium. *S*. *alvi* was grown in pollen-conditioned medium in the presence (black line) or absence (dashed line) of *G*. *apicola* culture supernatant (50%, v/v). Growth was determined based on OD_600_ at time points 0 h, 16 h, 36 h, and 72 h. n.s., not significant; * *P* < 0.05 (Welch’s *t* test, Benjamini and Hochberg adjusted [BH adj.]). (C) Six potentially cross-fed ions that accumulated during in vitro growth of *G*. *apicola* (left subpanel) and were consumed by *S*. *alvi* when it was grown in the presence *G*. *apicola* culture supernatant (right subpanel). Data from panels B and C come from the same experiment. Smoothed lines are added for interpretation purposes only in panel C and are dashed in the left subpanel because they are drawn through two points only. Error bars represent the standard deviation based on three replicate cultures. Chemical structures of the first annotation of each ion are shown. Asterisks indicate ions with ambiguous annotations. The numerical data of panel A can be retrieved from S8 Data. All other values are available in S1 Data.

A second example is pyruvate (*m/z* 87.008), which substantially accumulated in the gut of bees mono-colonized with *G*. *apicola* but was utilized as a substrate by other bacteria such as *S*. *alvi* and Firm-5 (Fig 6A). A syntrophic interaction between *G*. *apicola* and *S*. *alvi* had previously been suggested, because these bacteria are colocalized on the epithelial surface of the ileum [77] and harbor complementary metabolic capacities [32]. To test for potential cross-feeding of pyruvate from *G*. *apicola* to *S*. *alvi*, we supplemented the pollen-conditioned medium of *S*. *alvi* with culture supernatant of *G*. *apicola* cultures. The growth of *S*. *alvi* was slightly but significantly improved in the conditioned medium compared to the control medium (Fig 6B). Metabolome analysis (S10 Data) revealed six ions that accumulated during the growth of *G*. *apicola* and were utilized from the conditioned medium by *S*. *alvi* (Fig 6C). Besides pyruvate, these were ions corresponding to three putative fermentation products, a nucleoside derivative, and hydroxyphenylpropionate. We determined the concentration of pyruvate biochemically and showed that *G*. *apicola* indeed produces high levels of pyruvate (approximately 4 mM) and that this is subsequently utilized by *S*. *alvi* (S8 Fig). These results confirm our predictions from the in vivo dataset and show that bee gut bacteria engage in cross-feeding interactions. While not essential for gut colonization in itself as based on our mono-colonization experiments, such interactions may be important for community assembly and resilience and reflect the longstanding coexistence among these gut bacteria.

### Conclusions

The simple composition and experimental amenability of the honey bee gut microbiota facilitated our systems-level approach. We reconstituted the honey bee gut microbiota from cultured strains, characterized the metabolic output of the complete microbiota, identified the contributions of individual community members in vivo, and recapitulated their activities in vitro. Our results provide unprecedented insights into the metabolic functions of bee gut bacteria.

**Fig 7:**
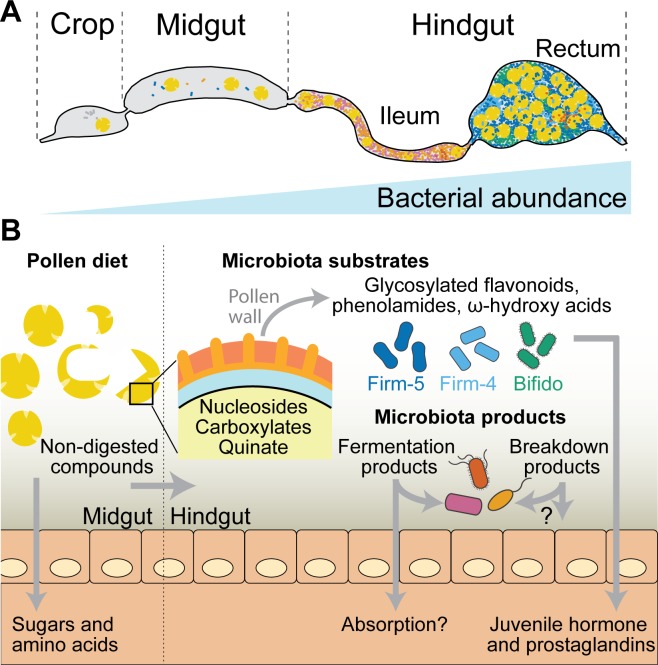
Summary of the metabolic activities of the bee gut microbiota identified in this study. (A) Schematic representation of the bee gut depicting the crop, midgut, and hindgut. The hindgut is divided into the ileum and the rectum, where the highest bacterial densities are found. Bacteria in the ileum are shown in magenta and orange (mostly Proteobacteria), and those in the rectum are shown in green and blue (mostly *Lactobacilli* and *Bifidobacteria*). Pollen grains are shown in yellow. (B) Pollen is likely predigested in the midgut, where bacterial levels are relatively low [45]. Here, the host absorbs accessible pollen-derived compounds such as simple sugars (glucose or fructose) and amino acids [46,47]. Nondigested pollen compounds enter the hindgut, where bacterial density is higher. We found nucleosides, various carboxylic acids (e.g., citrate, malate, and fumarate), and aromatic compounds (such as quinate) from pollen to be utilized by bee gut bacteria. In the posterior part of the hindgut (rectum), three community members (Firm-5, Firm-4, and *B*. *asteroides*) metabolize major components of the outer pollen wall, including flavonoids, phenolamides, and ω-hydroxy acids. The metabolic activities of the microbiota lead to the accumulation of fermentation products and intermediates of aromatic compound degradation. Some of the bacterial products may be utilized by other community members, as exemplified by the cross-feeding between *G*. *apicola* and *S*. *alvi*, or absorbed by the host. In addition, the gut symbiont *B*. *asteroides* seems to increase the production of several host metabolites (juvenile hormone derivatives and prostaglandins) that have key functions in immunity and physiology.

As in the mammalian and termite gut ecosystem [1,68], we conclude that most substrates utilized by the bee gut microbiota are indigestible compounds that originate from the diet of the host and accumulate in the hindgut where bacterial density is the highest (Fig 7). Such compounds include plant metabolites from the outer pollen wall, such as ω-hydroxy acids, phenolamides, and flavonoid glycosides. While one of the bee gut bacteria had previously been identified as utilizing a major pollen polysaccharide (pectin) [5], our data provides, to our knowledge, the first evidence for a role of the gut microbiota in breaking down outer pollen wall components. Bacterial fermentation of these pollen-derived compounds resulted in the accumulation of organic acids (e.g., succinate) and putative polyphenol degradation products, which are likely to impact the physicochemical conditions in the colonized gut. In addition, we found that host-derived signaling molecules are induced by *B*. *asteroides*, suggesting a specific interaction of this gut symbiont with the host.

Based on the mono-colonization experiments, we conclude that most metabolic output of the bee gut microbiota can be explained by the metabolic activities of individual community members. This suggests different metabolic niches in the gut, which could be in part explained by the distinct distribution of bacteria along the gut [77]. While we have found evidence for cross-feeding (e.g., between *G*. *apicola* and *S*. *alvi*), the metabolic exchange between bacteria seems not to be essential for gut colonization, as each community member was able to colonize on its own. This may also be the case for gut bacteria of other animals, as they cannot rely on the presence of specific interaction partners in the highly dynamic gut environment but rather adapt to diet-derived nutrients. However, the bee gut microbiota is relatively simple, and interspecies metabolic exchanges may be essential to establish in more complex communities such as those of the termite or mammalian gut.

The metabolic activities identified in this study are likely underlying the symbiotic functions of the bee gut microbiota and thus may be directly linked to the microbiota’s impact on bee health and physiology [34,41]. The large metabolic overlap between colonized and hive bees demonstrates the relevance of our findings and validates our gnotobiotic bee model. Moreover, our study highlights the versatility of high-throughput untargeted metabolomics to disentangle metabolic functions in microbial ecosystems. We believe that this systematic approach can be extended to other gnotobiotic animals to enable a better understanding of the diversity of metabolic activities and functions that are present in microbial communities.

## Materials and methods

### Honey bee experiments

Newly emerged bees were obtained from a healthy-looking colony of *Apis mellifera carnica* located at the University of Lausanne. In short, dark-eyed pupae were carefully removed from capped brood cells with sterile tweezers and transferred to sterilized plastic boxes as described previously [36]. Boxes with pupae were kept with a source of sterile sugar water (50% sucrose solution, w/v) at 35°C with 80%–90% humidity for 2 d until the bees emerged, followed by a reduction in temperature to 32°C. For each box, one or two newly emerged bees were dissected, and their homogenized hindguts (in 1 ml 1x PBS) cultured on growth media as described below. To minimize the chance of including contaminated bees in colonization experiments, we excluded cages for which bacterial growth was observed for the tested bees. For the colonization of newly emerged bees, bacterial strains were inoculated from glycerol stocks and restreaked twice. Details on bacterial strains and culture conditions can be found in S1 Table. Bacterial cells were harvested and resuspended in 1x PBS/sugar water (1:1, v/v) at an OD_600_ of 1. For colonization, bacterial suspensions were added to a source of sterilized pollen and provided to the newly emerged bees (for details, see S5 Text). MD bees were kept under the same conditions, with the same food sources, but without being exposed to bacteria. The mid- and hindgut (S9 Fig) of gnotobiotic bees were dissected at day 10 post colonization and stored at −80°C until further use.

To obtain age-controlled hive bees, several brood frames without adult bees were transferred from the hive to a ventilated Styrofoam box that was kept in an incubator at 32–34°C overnight. The next morning, the newly emerged bees were collected, marked on the thorax with a pen, and reintroduced into the hive. These bees were recollected 10 d later, and their mid- and hindguts were dissected and stored at −80°C until further use.

The colonization experiment was repeated at two different time points of the year (spring and fall, referred to as experiment 1 and experiment 2 in this study). Whenever possible, we included bees from both experiments in our analysis, such as for CL and MD bees. However, this was not possible for all mono-colonizations because of bacterial contaminations (as detected by qPCR) or in a few cases because of the presence of above-threshold viral loads. The precise numbers of bees included per condition are listed in S5 Table.

### Determining bacterial loads in the gut of honey bees

Bacterial loads were determined by qPCR using universal bacterial and species-specific 16S rRNA primers on DNA samples obtained from the gut tissues used for metabolomics analysis. Details on DNA/RNA extraction methods are given in S5 Text. Each DNA sample was screened with 11 different sets of primers targeting the actin gene of *A*. *mellifera*, the universal 16S rRNA region, and the species-specific 16S rRNA region of nine bacterial species, including the seven species used in this study and two non-core species frequently found in the gut of *A*. *mellifera*: Alpha-2.1 and *Lactobacillus kunkeei*. Primers used for this qPCR analysis are listed in S2 Table. We also screened all gut samples for the presence of viruses. Samples that were contaminated with other bacteria than the desired ones (i.e., >10^5^ bacteria cells detected by qPCR) or that had high virus titers were excluded from the analysis where possible (S5 Table, S5 Fig and S5 Text). The minimum information for publication of qPCR experiments (MIQE) guidelines were followed throughout the data analysis of the qPCR experiments [78]. Details on the qPCR analysis can be found in S5 Text.

### In vitro growth on pollen extracts

Bacteria were precultured on solid media from −80°C glycerol stocks before liquid cultures were inoculated for in vitro growth experiments. For *G*. *apicola* ELS0169, *S*. *alvi* wkB2, *F*. *perrara* PEB0191, and *B*. *apis* PEB0149, we used a modified M9 minimal medium supplemented with casamino acids and vitamins (http://dx.doi.org/10.17504/protocols.io.kdqcs5w). For *B*. *asteroides* ESL0170, the Firm-5 strains, and Firm-4 Hon2N, we used carbohydrate-free MRS (cfMRS) medium [79]. Bacteria were harvested from plates or spun down from overnight liquid cultures (the latter only for *Lactobacilli* and *B*. *asteroides*) and resuspended in the corresponding minimal medium. Freshly prepared liquid cultures were supplemented with either 10% (v/v) ddH_2_O or pollen extract and inoculated at a final OD_600_ of 0.05 (see S5 Text for details on pollen extract preparation). Half of the culture was immediately processed to determine colony-forming units (CFUs) and to harvest supernatants for metabolomics at time point 0 h, i.e., before growth. The other half of the culture was incubated for 16 h according to the conditions listed in S1 Table and then processed in the same way as the sample taken at time point 0 h. For CFU counting, serial dilutions were plated on solid media and incubated under the species-specific culturing conditions. For metabolomics analysis, the remaining bacterial culture was spun down at 20,000x *g* at room temperature for 10 min, and 300 μl of the culture supernatant was transferred to a fresh tube stored at −80°C until further processing. Five replicates were included for each species and treatment group.

For the cross-feeding experiment, *G*. *apicola* strain ESL0169 was grown for 8 h in pollen-supplemented M9 medium as described above to an OD_600_ of 0.11–0.15. Cultures were subsequently sterile filtered and mixed with fresh pollen-supplemented M9 medium 1:1 (v/v) in a total volume of 3 ml in a 12-well plate. For the control condition, non-inoculated pollen-supplemented M9 medium was incubated for 8 h, sterile filtered, and mixed with fresh pollen-supplemented M9 medium 1:1 (v/v). Then, *S*. *alvi* wkB2 was added to each well at a final OD_600_ of 0.05. The growth of *S*. *alvi* was assessed by OD_600_ measurements of a 100-μl aliquot in a 96-well plate with FLUOstar Omega microplate reader (Huberlab, Switzerland). As *S*. *alvi* tends to form aggregates, each culture was thoroughly mixed by pipetting up and down before transferring the aliquot and recording the OD_600_. For metabolomics analysis, supernatants were sampled at time points 0 h and 8 h for the *G*. *apicola* cultures and at time points 0, 16, 36, and 72 h for the *S*. *alvi* cultures. For biochemical quantification of pyruvate, we used a Pyruvate Assay Procedure kit (K-PYRUV, Megazyme, United States) according to the manufacturer’s microplate assay instructions. Samples of 8, 6, 4, 2, 1, 0.5, 0.25, and 0.125 mM pyruvate were used to generate the standard curve (slope = 0.062, intercept = 0.023, R^2^ = 0.987). Standards and samples were measured in triplicate at 340 nm with an Infinite M200PRO microplate reader (Tecan, Switzerland).

### Metabolite extraction and profiling

Metabolites from gut and pollen samples were water-extracted after mechanical disruption, and supernatants from the in vitro experiments were harvested by centrifugation. Gut samples were preselected based on their wet-weight (arithmetic mean 55.1 mg, standard deviation 9.9). Ten times more water than the gut wet weight (v/w) was added, and the samples were homogenized with 0.1 mm zirconia beads (0.1 mm dia. Zirconia/Silica beads; Carl Roth) in a Fast-Prep24 5G homogenizer (MP Biomedicals) at 6 m/s for 45 s. While most of the homogenate was snap-frozen in liquid nitrogen for subsequent DNA/RNA extraction, aliquots of 100 μl were diluted 1:1 with water for metabolite extractions. To do so, the diluted aliquots were incubated in a preheated thermomixer at 80°C and 1,400 rpm for 3 min. After each minute, the samples were vortexed for 10 s. Subsequently, the samples were centrifuged at 20,000x g and 4°C for 5 min, and 150 μl of the resulting supernatant was transferred to a new tube and centrifuged again at 20,000x g for 30 min. Samples for untargeted metabolomics analysis were further diluted 10x in water. All samples were stored at −80°C before metabolomics analysis.

For untargeted analysis, samples were injected into an Agilent 6550 time-of-flight mass spectrometer (ESI-iFunnel Q-TOF, Agilent Technologies) operated in negative mode, at 4 Ghz, high resolution, and with a mass / charge (*m/z*) range of 50−1,000 [21]. The mobile phase was 60:40 isopropanol:water (v/v) and 1 mM NH_4_F at pH 9.0 supplemented with hexakis(1H, 1H, 3H- tetrafluoropropoxy)phosphazine and 3-amino-1-propanesulfonic acid for online mass correction. After processing of raw data as described in [21], *m/z* features (ions) were annotated by matching their accurate mass-to-sum formulas of compounds in the KEGG database with 0.001 Da mass accuracy and accounting for deprotonation [M-H^+^]^-^. The complete KEGG database was used because it has broad coverage of plant, bacterial, and insect metabolic pathways. Notably, this metabolomics method cannot distinguish between isobaric compounds, e.g., metabolites having identical *m/z* values, and in-source fragmentation cannot be accounted for. The raw data of samples from the three sets of experiments (bee gut samples, in vitro supernatants, and cross-feeding supernatants) were processed and annotated separately to accommodate their different sample matrices or times of measurement. These data can be explored in S2 Data, S9 Data and S10 Data. Raw data processing and annotation took place in MATLAB (MATLAB 2015b, The Mathworks, Natick) as described previously [21], and downstream processing and statistical tests were performed in R (version 3.3.2, R Foundation for Statistical Computing, Vienna, Austria).

Selected metabolite samples were measured in targeted fashion using ultra-high-pressure chromatography-coupled tandem mass spectrometry as described before [72]. Metabolite quantifications were performed by interpolating observed intensities to a standard curve of the metabolite using a linear model (R^2^ ≥ 0.95). Metabolites with standard curves of R^2^ ≤ 0.95 or in which intensities had to be extrapolated can be interpreted as relative changes only and were labeled in grey in all plots (S6B Fig). We used the weights of the extracted material to express the concentrations in millimole per gram of gut or gram of pollen. The dataset can be found in S6 Data.

Flavonoid ions were targeted for MS/MS fragmentation as [M-H^+^]^-^ electrospray derivatives with a window size of ± 4 m/z in Q1. Fragmentation of the precursor ion was performed by collision-induced dissociation at 0, 10, 20, and 40 eV collision energy, and fragment-ion spectra were recorded in scanning mode by high-resolution time-of-flight MS. Spectra were interpreted using MetFrag [80], and spectral cosine similarity scores were calculated between reference spectra that were obtained in-house or library spectra from MassBank of North America (MoNA, http://mona.fiehnlab.ucdavis.edu/). For further details, see S5 Text.

### Untargeted metabolomics data analysis

All steps of the downstream data analysis were performed in R (R Foundation for Statistical Computing, Vienna, Austria). Samples from double injections (technical replicates) were confirmed to be highly similar and averaged. Subsequent analyses were performed on these averaged ion intensities, which are available in S2D Data, S9 Data and S10 Data.

Principal component analysis (*pca* function in R) on ion intensities was used for the multivariate inspection of co-clustering of samples from different groups. For reasons of transparency, we carried out four different PCAs on all annotated ions or the subset of ions with robust changes between CL and MD bees and on log2-normalized or Z-score normalized ion intensities. Z-score transformation was used to remove the domination of high-intensity ions.

Ions that were deemed robustly different between the CL and MD bees were those that were significantly different (Welch *t* test with Benjamini and Hochberg correction ≤ 0.01, *t*.*test* and *p*.*adjust(x*, *method =“BH”)* in R) between CL and MD in both independent experiments. Differences between MD and CL samples were expressed as log2(fold change) values for both experiments separately and for pooled data of both experiments (see S2A Data). Fold changes were based on the arithmetic mean of the CL samples divided by the arithmetic mean of the MD samples. The standard error of the log2(fold change) was computed as the square root of the sum of the squared standard errors of the log2-transformed intensities of both CL and MD.

Enrichment analyses were computed on compound class categories from KEGG (in-house database), which are added in the column “compound class” in S2A Data. Some ions with ambiguous annotations had a compound class associated with multiple of these annotations. However, supported by the observation that compound classes between alternative annotations were often the same (or highly related), only the compound class of the first annotation was used as input for one-sided Fischer’s exact tests (*fisher*.*test(x*, *alternative =“greater”)* in R) on a 2 x 2 contingency table for every compound class. Compound classes associated with a single ion were removed from the results because they were deemed not biologically meaningful.

Ions were sorted based on to what extent they are responsible for explaining the separation between the CL and MD groups from experiment 2. To do this, these datasets were selected as the input for an OPLS-DA (*opls* from the ropls R package). The correlation and covariance between the log10-transformed ion intensities of included samples and the *opls* “scoreMN” output were computed with the *cor* and *cov* functions in R, respectively. The resulting scores were plotted in a so-called S-plot (Fig 3). The substrate and product ions most responsible for the separation were selected based on an absolute correlation ≥0.8 and an absolute covariance of ≥5. Because such analyses can be prone to overfitting, we tested the sensitivity of the most discriminatory ion selection by implementing a “leave one out strategy” and concluded that the selected ions are robust (>80% present in all 1,000 permutations).

One-way ANOVA (*aov* adjusted with *TukeyHSD(x*, *conf*.*level = 0*.*99)* in R) was performed between all bee gut samples after selecting the relevant samples from the data matrix and normalizing the intensities to ion standard (Z-) scores (i.e., by row) by subtracting for every ion its arithmetic mean intensity and dividing the resulting values by the standard deviation of its respective ion intensity. The results of the full ANOVA analysis can be explored in S7 Data. For this study, the focus was on differences between any group and MD bees, which were considered significant when having a Tukey HSD post hoc adjusted *P* value ≤ 0.05. When for a specific mono-colonization group this significance cut-off was met and the direction of the change was the same as that for CL versus MD, the ion was considered to be “explained” by this group.

In order to enrich for pollen ions, we only considered ions with an arithmetic mean intensity of ≥10,000 in the pollen samples, in addition to being highly significantly different from water-matrix control samples (Welch *t* test with BH correction ≤ 0.001, *t*.*test* and *p*.*adjust(x*, *method = “BH”)* in R combined with log2(fold change) difference of ≥ 2).

For the in vitro data (S9 Data), the goal was to identify pollen substrates and bacterial products for which changes in levels were observed in vivo and in vitro. Pollen ions were mapped by matching the top annotation formula of both datasets. For all media-strain combinations, we performed a statistical comparison (Welch *t* test with BH correction, *t*.*test* and *p*.*adjust(x*, *method =“BH”)* in R) between the time points 16 h and 0 h and considered only those ions with a log2(fold change) of ≥|1| and BH adj. *P* value of ≤0.01 as significant in vitro products or substrates. In order to be certain that only pollen-derived substrates were included, for every strain only ions that displayed a significant negative log2(fold change) exclusively in the base medium supplemented with pollen extract were considered as in vitro pollen substrates.

To identify ions that might be cross-fed between *G*. *apicola* and *S*. *alvi*, ions were selected that increased during the growth of *G*. *apicola* and were depleted when *S*. *alvi* was grown in this conditioned medium mixed 1:1 with fresh base medium. To do this, all ion intensities for both strains (S10 Data) were split and transformed to log2(fold change) with respect to the first time point of sampling. Ions that had a log2(fold change) of ≥1 during *G*. *apicola* growth and a log2(fold change) of ≤−1 during *S*. *alvi* growth were selected. The raw data and R code for recapitulating the metabolomics data analysis can be found in S11 Data.

## Supporting information

S1 Data Numerical values underlying the graphs in Fig 2, Fig 3, Fig 4, Fig 5, Fig 6, S1 Fig, S5 Fig and S8 Fig. (XLSX)

S2 Data In vivo untargeted metabolomics data. (XLSX)

S3 Data Compound category enrichment analysis. (XLSX)

S4 Data Tandem mass spectrometry (MS/MS) fragmentations of flavonoids. (XLSX)

S5 Data Comparison between Zheng et al. [34] and our study. (XLSX)

S6 Data Targeted metabolomics data. (XLSX)

S7 Data R list object with the ANOVA results of all in vivo treatment comparisons. (ZIP)

S8 Data Mono-colonization plots of all 372 robust ion changes and R list object containing the numerical values of the Z-scores. (ZIP)

S9 Data In vitro untargeted metabolomics data. (XLSX)

S10 Data Cross-feeding untargeted metabolomics data. (XLSX)

S11 Data Raw data and R code of the metabolomics data analysis. (ZIP)

S1 Fig Principal component analysis of the metabolic profiles of microbiota-depleted (MD), colonized (CL), and hive bees. (A) Principal component analysis (PCA) based on log2-transformed ion intensities of all 1,079 annotated ions. (B) PCA based on Z-score normalized ion intensities of all 1,079 annotated ions. (C) PCA based on log2-transformed ion intensities of the 372 ions that show significant changes between CL and MD bees. (C) PCA based on Z-score normalized ion intensities of the 372 ions that show significant changes between CL and MD bees. CL and MD bees come from two independent experiments (see S5 Text and S5 Table) conducted at two different time points of the same year. Hive bees utilized for metabolomics analysis came from the second experiment. We used log2-transformed and Z-score normalized ion intensities to show the large effect of high-intensity ions on the percentage of variance explained (PC1). We conducted the analysis on all ions and the subset of significant ion changes to illustrate that the two seeming outlying samples in B are due to ions that are not of relevance for our subsequent analysis. PC1, principal component 1; PC2, principal component 2. The numerical data can be found in S1 Data.
(TIF)

S2 Fig Log2(fold change) between CL and MD bees and mono-colonization results for ions belonging to five different compound groups. (A) Pyrimidine nucleosides and analogues (green) and purine nucleosides and analogues (orange). (B) Glycosylated flavonoids (green), non-glycosylated flavonoids (orange), and intermediates of aromatic compound degradation pathways (purple). (C) Pollen wall components with ω-hydroxy acids (green) and phenolamides (orange). (D) Carboxylic acids and derivatives (green) and keto acids and derivatives (orange). (E) Host metabolites with eicosanoids (green) and juvenile hormone derivatives (orange). For all five panels, the log2(fold change) values between CL and MD bees are plotted as bar graphs for each ion. The first annotation of each ion is provided. An asterisk indicates ambiguous annotations. Asterisks below/above bars indicate significant fold changes (based on Welch’s *t* test on two independent experiments, both experiments Benjamini and Hochberg adjusted [BH adj.] *P* < 0.01). ANOVA results for the comparison of each treatment group versus MD bees are depicted below each bar graph. Significant metabolite changes in vivo are indicated by black squares. Grey shading indicates that the metabolite was annotated in the in vitro dataset, and an asterisk in the black square indicates that the same metabolic change was recapitulated in vitro. Note that only metabolites with log2(fold changes) of ≥|1| and BH adj. *P* < 0.01 (Welch’s *t* test) from the in vitro experiments were considered. Ba, *B*. *apis* mono-colonized; Bi, *B*. *asteroides* mono-colonized; CL, colonized with the reconstituted microbiota; F4, Firm-4 mono-colonized; F5, Firm-5 mono-colonized; Fp, *F*. *perrara* mono-colonized; Ga, *G*. *apicola* mono-colonized; Hive, hive bees; MD, microbiota-depleted; Sa, *S*. *alvi* mono-colonized. The numerical data of the bar graphs can be extracted from S2A Data.
(TIF)

S3 Fig Example spectral similarity analysis for ion #821 (*m/z* 431.099), corresponding to afzelin. (A) displays fragmentation spectrum for ion #821 on top and a reference spectrum for afzelin below (CCMSLIB00000845703, MassBank of North America). (B) displays fragmentation spectrum for ion #821 on top and the reference spectrum obtained in house for the pure vitexin standard. A high spectral similarity score with afzelin standard confirms the annotation of ion #821 as afzelin. For masses of the fragments, raw ion spectra, and alternative annotations, see S4 Data.
(TIF)

S4 Fig Targeted metabolomics, i.e., liquid chromatography-tandem mass spectrometry (LC-MS/MS) analysis of selected metabolites. (A) Correlation in log2(fold change) of metabolites or ions annotated both in LC-MS/MS and quadrupole-time of flight (Q-TOF). Identical data are plotted in both panels, with the left panel presenting the names of the metabolites (or the most likely annotation of the corresponding ion) and the right panel presenting the standard error of the log2(fold change) for both methods. (B) Concentrations of selected metabolites in pollen extracts and gut samples. Grey zones indicate extrapolation from the standard curve, i.e., relative changes. The line indicates the arithmetic mean concentration. The numerical data of untargeted metabolomics can be extracted from S2A Data. The numerical data of targeted metabolomics can be found in S6 Data.
(TIF)

S5 Fig Bacterial colonization levels in guts of mono-colonized bees. Each plot presents the colonization levels of a specific bee gut bacterial associate as based on quantitative PCR (qPCR) with species-specific 16S rRNA primers (S2 Table). In addition to the seven major community members used in our colonization experiments, we also screened for Alpha-2.1 and *L*. *kunkeei*, as these two species can constitute common contaminants in gnotobiotic bees. The results are shown according to the 10 treatment groups on the *x*-axis, i.e., hive bees, CL bees, MD bees (same as in Fig 2), and mono-colonized bees (Ga, Fp, Sa, Ba, Bi, F4, and F5). Open and filled circles indicate samples coming from experiment 1 and experiment 2, respectively. The red line corresponds to 10^5^ bacterial cells per gut, which we consider as our threshold of presence of the given bacterial species. All values below this limit of the qPCR assay are plotted below this line. A few samples had slightly higher values than 10^5^ for *S*. *alvi*, Firm-5, *B*. *asteroides*, and *L*. *kunkeei*. They were still included in our analysis to increase statistical power. Based on the metabolomics analysis and the recapitulation of our findings in vitro, we feel confident that these possible contaminants had a negligible effect on our results. Median values are shown as black lines for hive bee samples, CL bee samples, and mono-colonization samples corresponding to the primer pair used. Ba, *B*. *apis* mono-colonized; Bi, *B*. *asteroids* mono-colonized; CL, colonized with the reconstituted microbiota; F4, Firm-4 mono-colonized; F5, Firm-5 mono-colonized; Fp, *F*. *perrara* mono-colonized; Ga, *G*. *apicola* mono-colonized; Hive, hive bees; MD, microbiota-depleted; Sa, *S*. *alvi* mono-colonized. The numerical data can be found in S1 Data.
(TIF)

S6 Fig Metabolites detected in the pollen diet of bees using untargeted quadrupole-time of flight (Q-TOF) and targeted metabolomics, i.e., liquid chromatography-tandem mass spectrometry (LC-MS/MS). (A) A volcano plot represents ions enriched in pollen (black or colored dots). Colored dots belong to enriched categories based on their main annotation (significance levels mentioned in text), except for “nucleosides and analogs,” which consists of the pooled groups “purine nucleosides and analogues,” 4/5, one-sided Fishers exact test *P* = 0.066, and “pyrimidine nucleosides and analogues,” 3/4, *P* = 0.148. (B) Concentrations of metabolites detected in pollen extract using targeted LC-MS/MS. Three replicates are shown as dots, with a line indicating the arithmetic mean. Metabolites that could be quantified using interpolations from standard curves are indicated in black. Metabolites for which standard curves did not meet the linearity criteria or for which values had to be extrapolated are plotted in grey. The inset shows identical data with a rescaled *x*-axis for more fine-grained inspection. The numerical values of the volcano plot and the targeted metabolomics can be found in S2A Data and S6 Data, respectively.
(TIF)

S7 Fig Metabolic changes recapitulated in vitro during growth on pollen-conditioned medium. Volcano plots of significance (Welch’s *t* test Benjamini and Hochberg adjusted [BH adj.] *P* value) versus log2(fold change) showing metabolic changes in pollen-conditioned medium at time point 16 h relative to 0 h. Ions identified as likely pollen-derived are highlighted in black. Ions highlighted in red correspond to metabolites that showed robust changes between colonized (CL) and microbiota-depleted (MD) bees in vivo and are explained in vivo and in vitro by the same community member. Annotations of these ions are given and summarized in S3 Table. Other annotated ions are plotted in grey. The numerical values can be extracted from S9 Data.
(TIF)

S8 Fig Growth improvement of *S*. *alvi* in *G*. *apicola-*conditioned medium and quantification of pyruvate using a biochemical assay. (A) Independent cross-feeding experiment that was carried out in the same way as the experiment presented in Fig 6B. *S*. *alvi* was grown in pollen-conditioned medium in the presence (black line) or absence (dashed line) of *G*. *apicola* culture supernatant (50%, v/v). (B) Quantification of pyruvate in the pollen extract-based culture medium before and after growth of *G*. *apicola*. (C) Quantification of pyruvate in the *G*. *apicola*-conditioned culture medium during growth of *S*. *alvi* at different time points. Data from panel B and C come from the cross-feeding experiment presented in panel A. The numerical values can be found in S1 Data.
(TIF)

S9 Fig Dissected gut of a hive bee. Different gut regions and connected tissues are indicated. Dashed lines depict borders of different gut regions. The red dashed lines indicate the part of the gut taken for metabolomics analysis and DNA extraction/quantitative PCR (qPCR) analysis.
(TIF)

S1 Table Bacterial strains used in this study. (DOCX)

S2 Table Primers used in this study and standard curve characteristics. (DOCX)

S3 Table List of ions explained in vivo and in vitro by the same community members. (DOCX)

S4 Table List of ions for which we detected possible cross-feeding based on ANOVA results. (DOCX)

S5 Table Total number of bees analyzed by quantitative PCR (qPCR) and number of bees selected for metabolomics analysis. (DOCX)

S1 Text Tandem mass spectrometry (MS/MS) analysis to establish identity of selected flavonoids. (PDF)

S2 Text Overlap between metabolomics data in Zheng et al. and this study. (PDF)

S3 Text Overlap between targeted and untargeted metabolomics for colonized (CL) and microbiota-depleted (MD) bees. (PDF)

S4 Text Pollen ions and targeted metabolomics on pollen extracts. (PDF)

S5 Text Supporting methods. (PDF)

## Abbreviations

BH adj: Benjamini and Hochberg adjusted
CFU: colony-forming unit
CL: colonized
KEGG: Kyoto Encyclopedia of Genes and Genomes
HSD: honest significant difference
LC-MS/MS: liquid chromatography-tandem mass spectrometry
MD: microbiota-depleted
MS/MS: tandem mass spectrometry
OPLS-DA: Orthogonal Projection of Least Squares-Differentiation Analysis
qPCR: quantitative PCR
Q-TOF: quadrupole-time of flight
TCA: tricarboxylic acid
