# Supplementary figures and images for: Disentangling metabolic functions of bacteria in the honey bee gut (part 2 of 3)
Source: PLoS Biol. 2017 Dec 12;15(12):e2003467. doi: 10.1371/journal.pbio.2003467 (PMC5726620; doi:10.1371/journal.pbio.2003467)

Quinate\*

# 286 191.056 microbial substrate

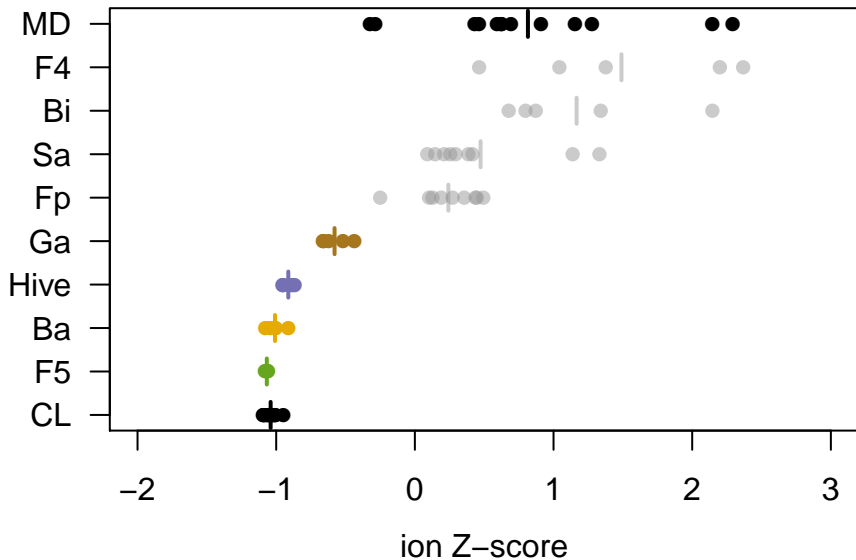

Supplement: S8 Data — (ZIP) [file pbio.2003467.s008.zip › Z-score_plots/286 microbial substrate Quinate.pdf]

# D-Mycinoside

# 287 191.091 microbial substrate

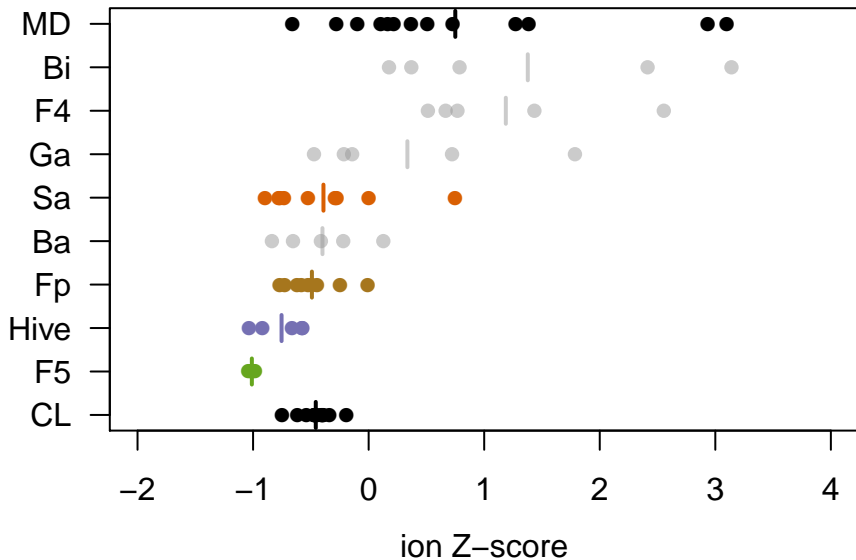

Supplement: S8 Data — (ZIP) [file pbio.2003467.s008.zip › Z-score_plots/287 microbial substrate D-Mycinose.pdf]

# Scytalone\*

# 293 193.050 microbial substrate

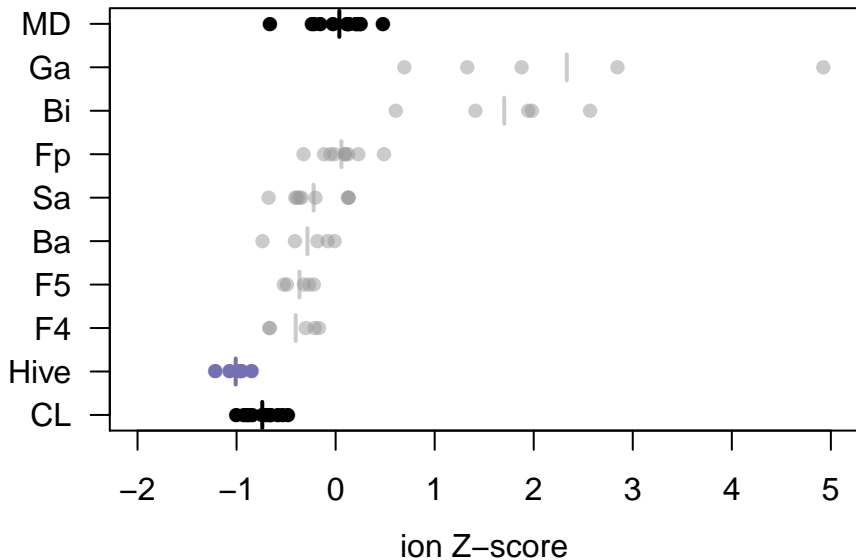

Supplement: S8 Data — (ZIP) [file pbio.2003467.s008.zip › Z-score_plots/293 microbial substrate Scytalone.pdf]

**5-O-Methyl-myo-inositol\***  
**# 294 193.071 microbial substrate**

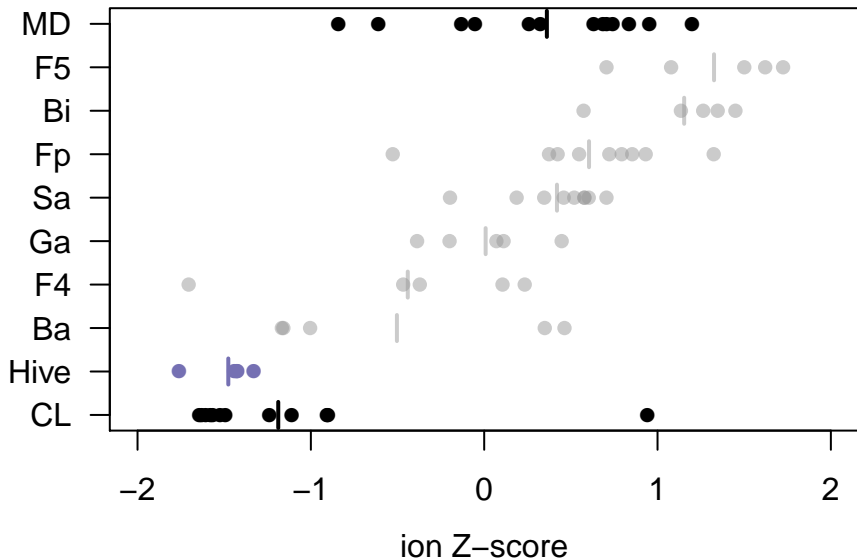

Supplement: S8 Data — (ZIP) [file pbio.2003467.s008.zip › Z-score_plots/294 microbial substrate 5-O-Methyl-myo-inositol.pdf]

**L-Tyrosine methyl ester\***  
**# 297 194.082 microbial product**

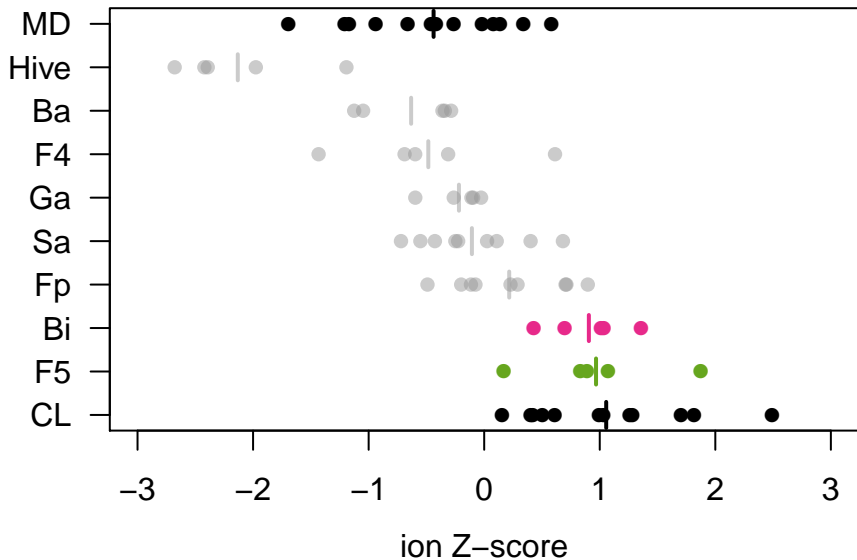

Supplement: S8 Data — (ZIP) [file pbio.2003467.s008.zip › Z-score_plots/297 microbial product L-Tyrosine methyl ester.pdf]

**D-Gluconic acid\***  
**# 300 195.051 microbial substrate**

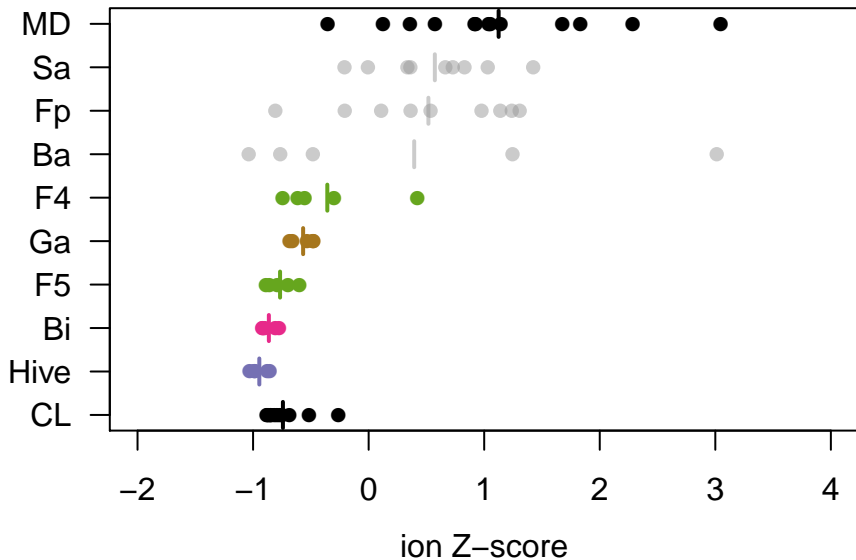

Supplement: S8 Data — (ZIP) [file pbio.2003467.s008.zip › Z-score_plots/300 microbial substrate D-Gluconic acid.pdf]

**O-Phospho-L-homoserine\***  
**# 302 198.018 microbial product**

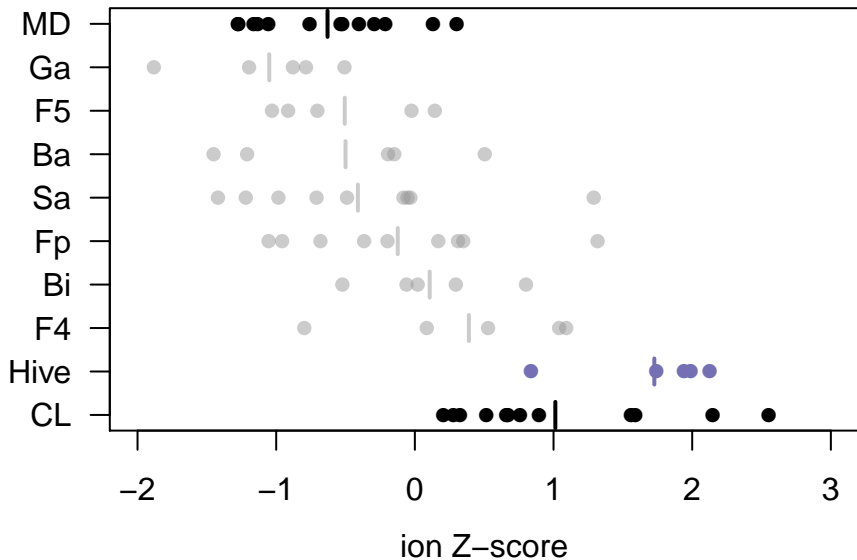

Supplement: S8 Data — (ZIP) [file pbio.2003467.s008.zip › Z-score_plots/302 microbial product O-Phospho-L-homoserine.pdf]

**Clavulanic acid\***  
**# 303 198.040 microbial product**

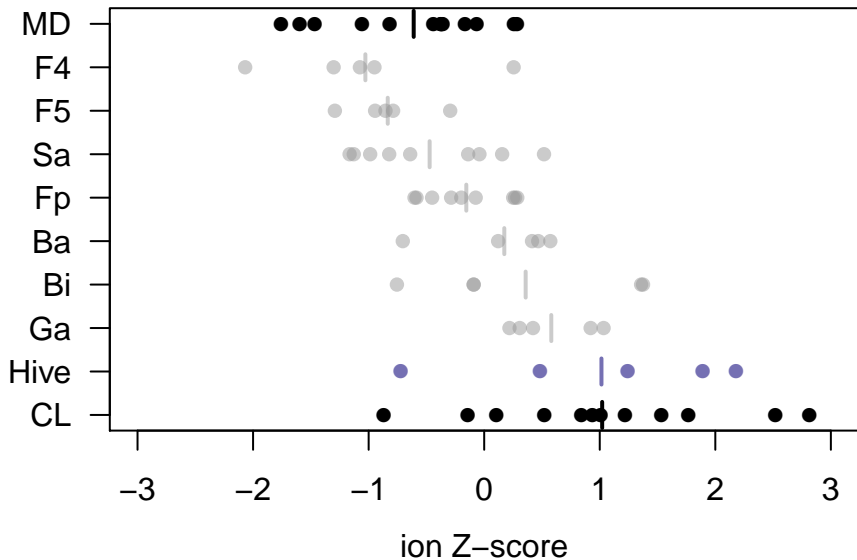

Supplement: S8 Data — (ZIP) [file pbio.2003467.s008.zip › Z-score_plots/303 microbial product Clavulanic acid.pdf]

**2,2',3-Trihydroxybiphenyl\***  
**# 310 201.055 microbial product**

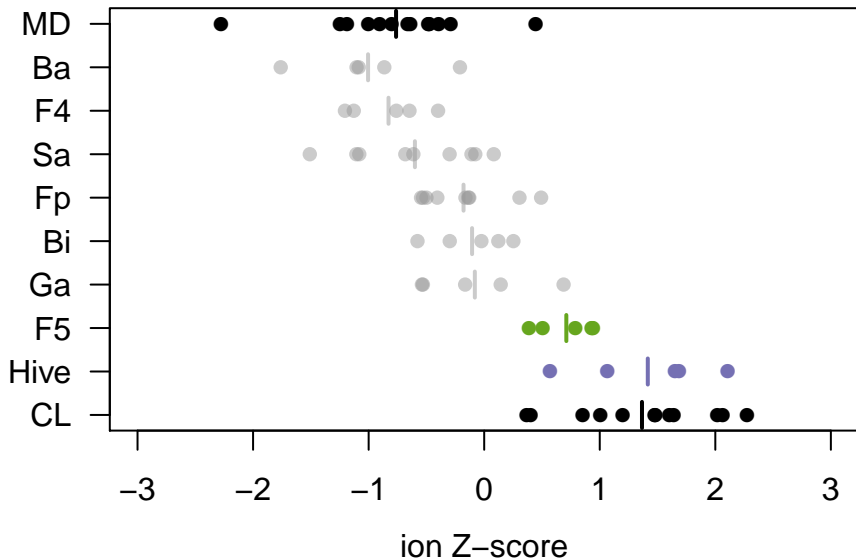

Supplement: S8 Data — (ZIP) [file pbio.2003467.s008.zip › Z-score_plots/310 microbial product 2,2',3-Trihydroxybiphenyl.pdf]

# Metamitron

# 311 201.078 microbial product

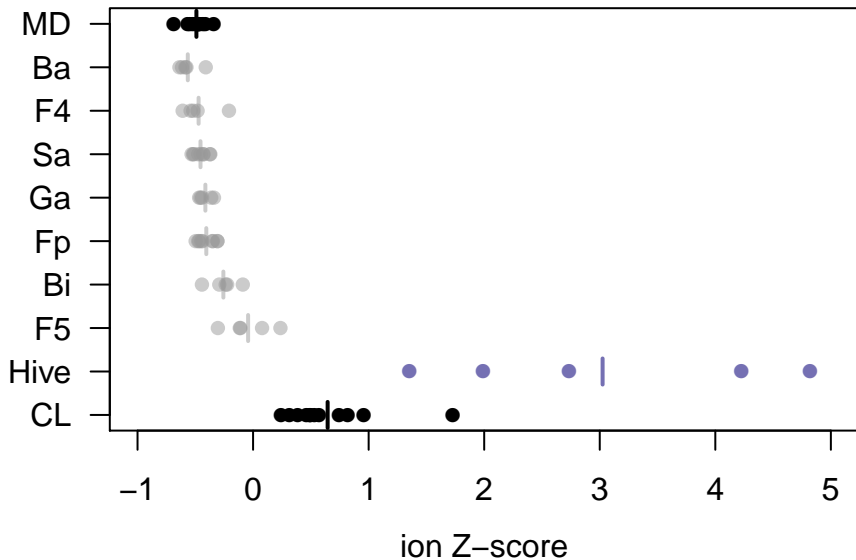

Supplement: S8 Data — (ZIP) [file pbio.2003467.s008.zip › Z-score_plots/311 microbial product Metamitron.pdf]

**Proclavaminic acid\***  
**# 312 201.088 microbial product**

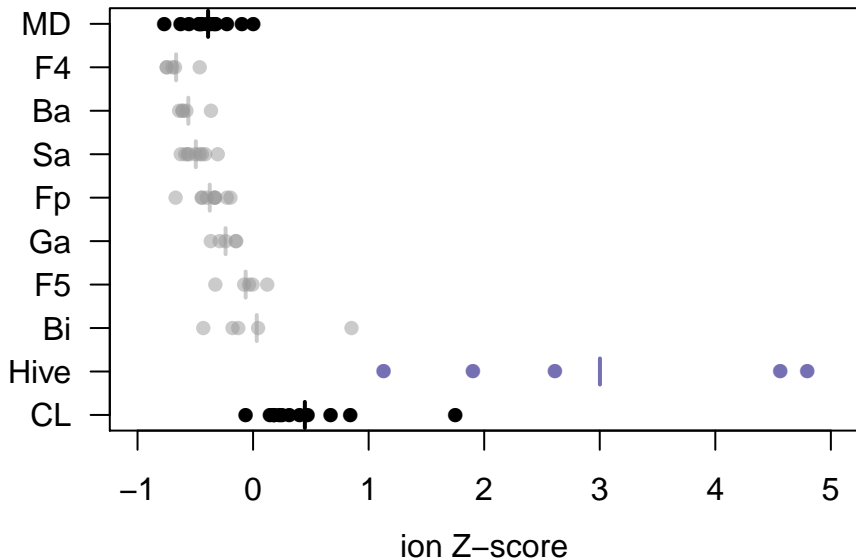

Supplement: S8 Data — (ZIP) [file pbio.2003467.s008.zip › Z-score_plots/312 microbial product Proclavaminic acid.pdf]

# Sebacic acid\*

# 313 201.113 microbial product

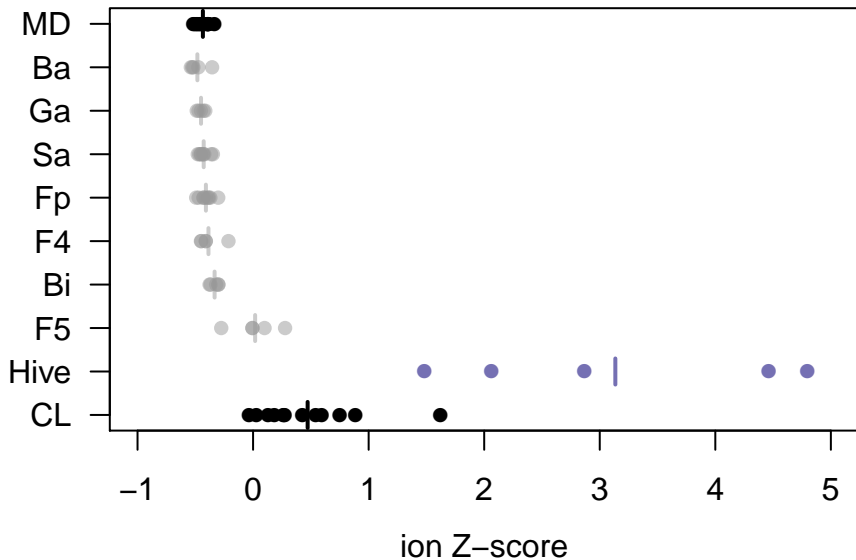

Supplement: S8 Data — (ZIP) [file pbio.2003467.s008.zip › Z-score_plots/313 microbial product Sebacic acid.pdf]

**N-Acetyl-D-fucosamine\***  
**# 319 204.086 microbial substrate**

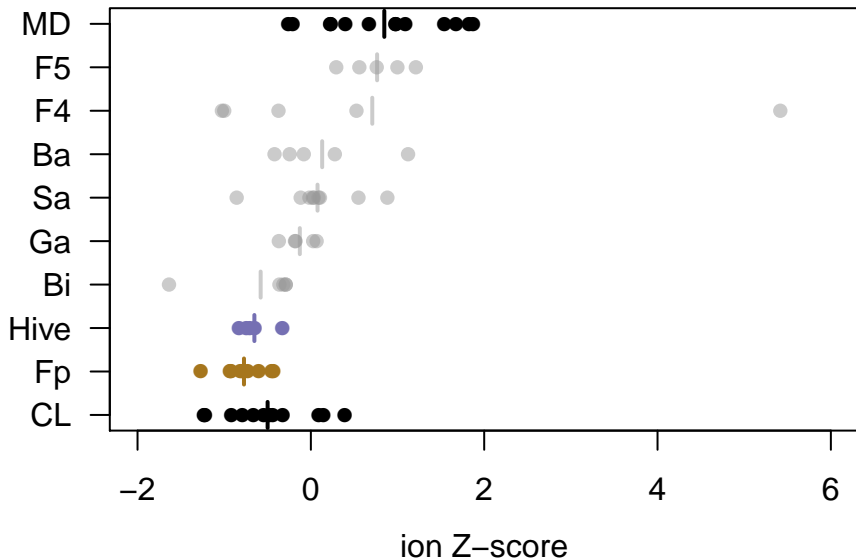

Supplement: S8 Data — (ZIP) [file pbio.2003467.s008.zip › Z-score_plots/319 microbial substrate N-Acetyl-D-fucosamine.pdf]

**1-Aminocyclopropane-1-carboxylate\***  
**# 32 100.040 microbial substrate**

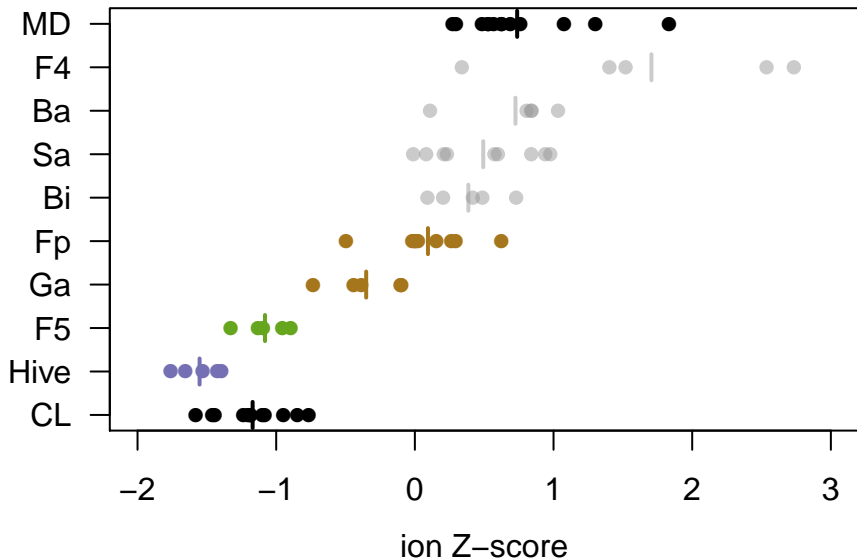

Supplement: S8 Data — (ZIP) [file pbio.2003467.s008.zip › Z-score_plots/32 microbial substrate 1-Aminocyclopropane-1-carboxylate.pdf]

**2-(2'-Methylthio)ethylmalic acid\***  
**# 327 207.032 microbial product**

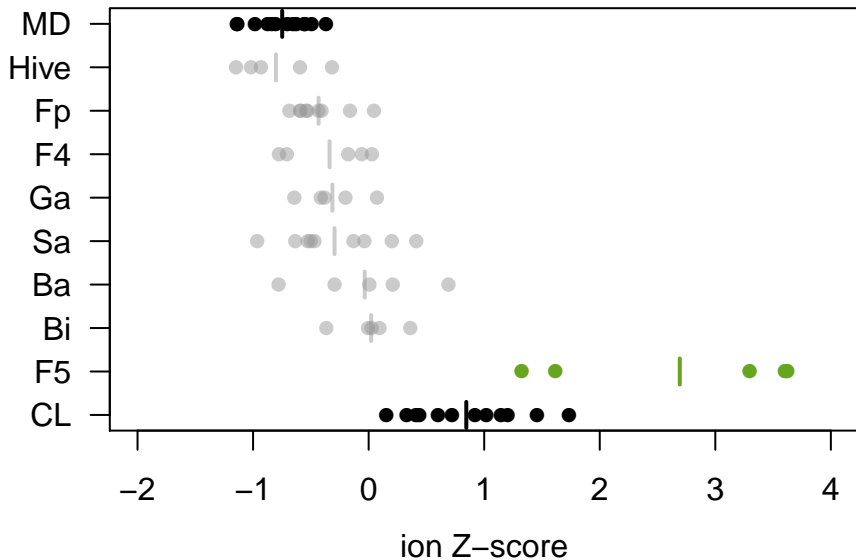

Supplement: S8 Data — (ZIP) [file pbio.2003467.s008.zip › Z-score_plots/327 microbial product 2-(2'-Methylthio)ethylmalic acid.pdf]

**L-Kynurenine\***  
**# 328 207.077 microbial substrate**

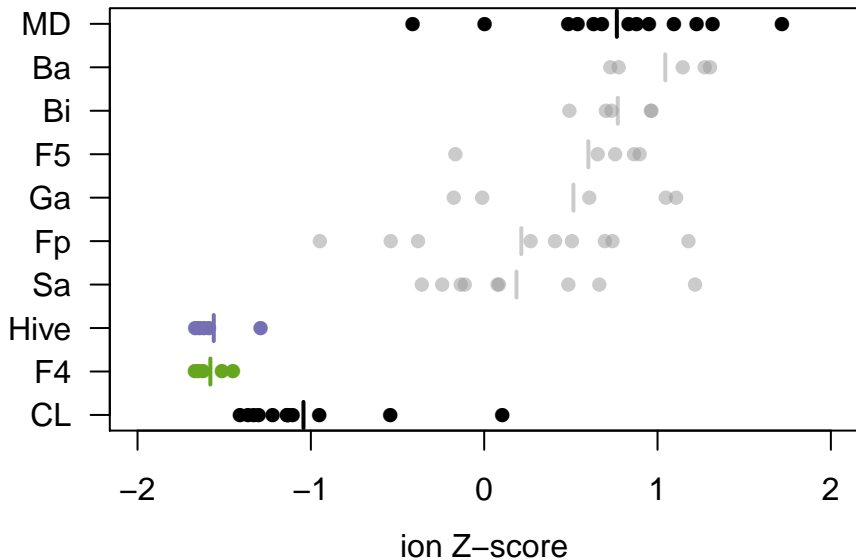

Supplement: S8 Data — (ZIP) [file pbio.2003467.s008.zip › Z-score_plots/328 microbial substrate L-Kynurenine.pdf]

**Sedoheptulose\***  
**# 333 209.066 microbial substrate**

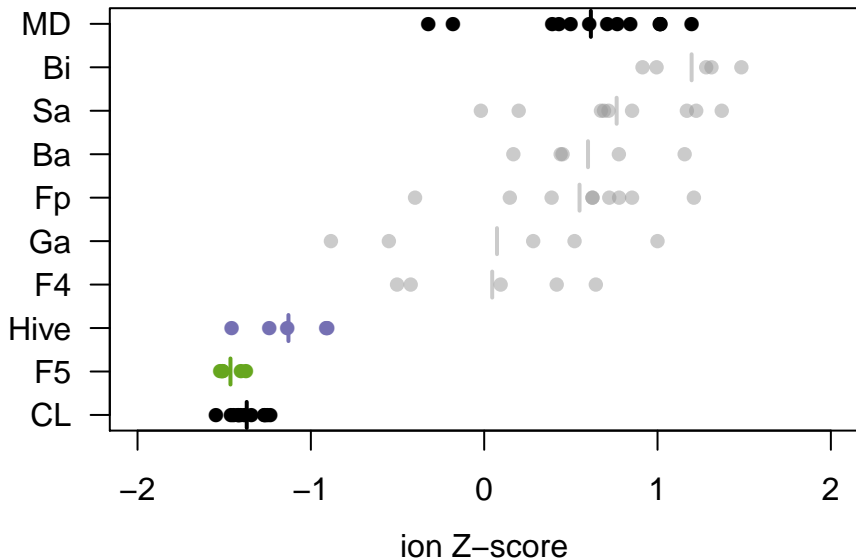

Supplement: S8 Data — (ZIP) [file pbio.2003467.s008.zip › Z-score_plots/333 microbial substrate Sedoheptulose.pdf]

**Sinapyl alcohol\***  
**# 334 209.082 microbial substrate**

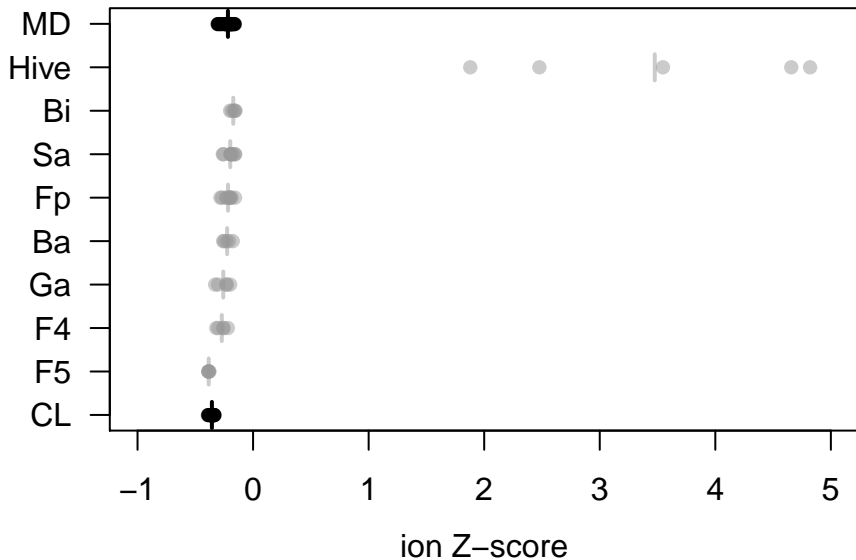

Supplement: S8 Data — (ZIP) [file pbio.2003467.s008.zip › Z-score_plots/334 microbial substrate Sinapyl alcohol.pdf]

**6-Carboxy-5,6,7,8-tetrahydropterin**  
**# 336 210.062 microbial substrate**

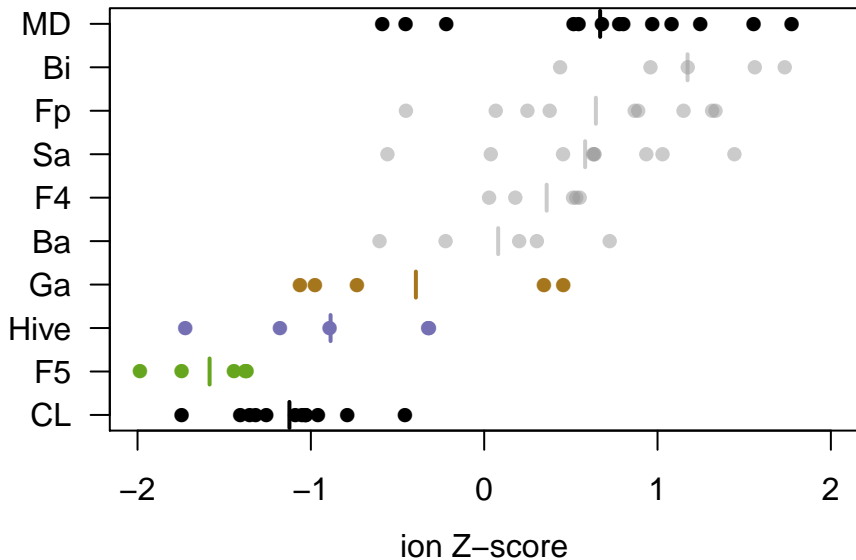

Supplement: S8 Data — (ZIP) [file pbio.2003467.s008.zip › Z-score_plots/336 microbial substrate 6-Carboxy-5,6,7,8-tetrahydropterin.pdf]

**Mescaline\***  
**# 337 210.114 microbial product**

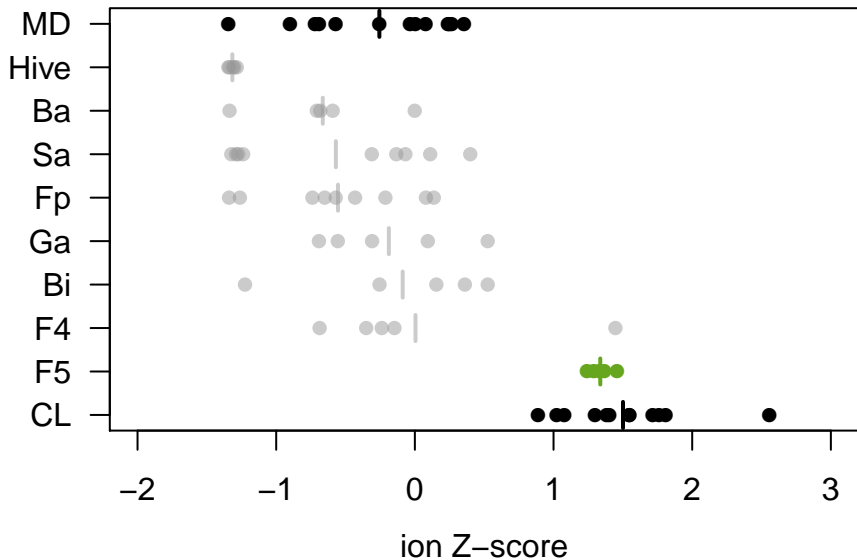

Supplement: S8 Data — (ZIP) [file pbio.2003467.s008.zip › Z-score_plots/337 microbial product Mescaline.pdf]

# Elaeokanine C

# 338 210.150 microbial substrate

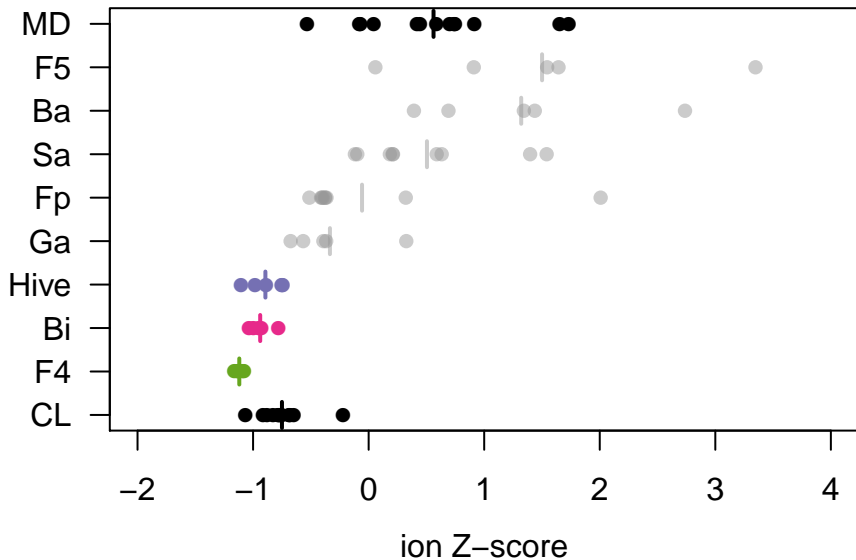

Supplement: S8 Data — (ZIP) [file pbio.2003467.s008.zip › Z-score_plots/338 microbial substrate Elaeokanine C.pdf]

**2-Oxobutanoate\***  
**# 34 101.024 microbial substrate**

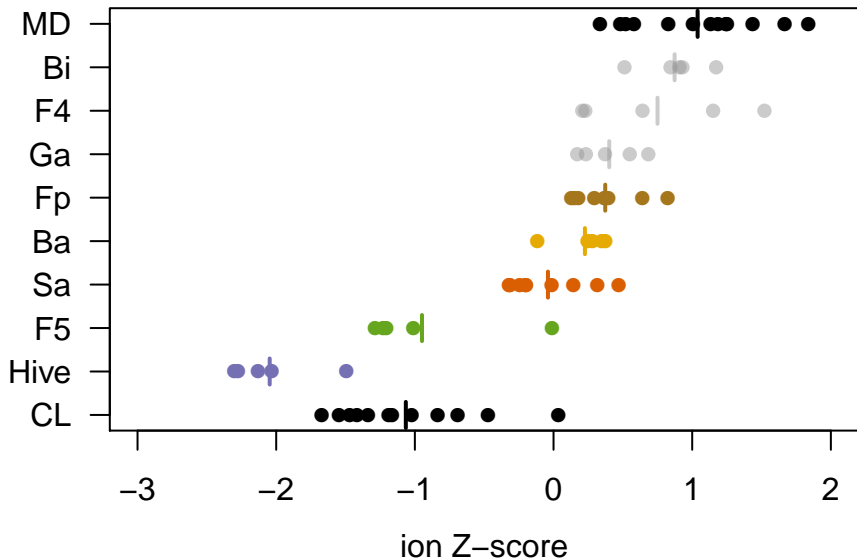

Supplement: S8 Data — (ZIP) [file pbio.2003467.s008.zip › Z-score_plots/34 microbial substrate 2-Oxobutanoate.pdf]

# Dethiobiotin

# 349 213.124 microbial substrate

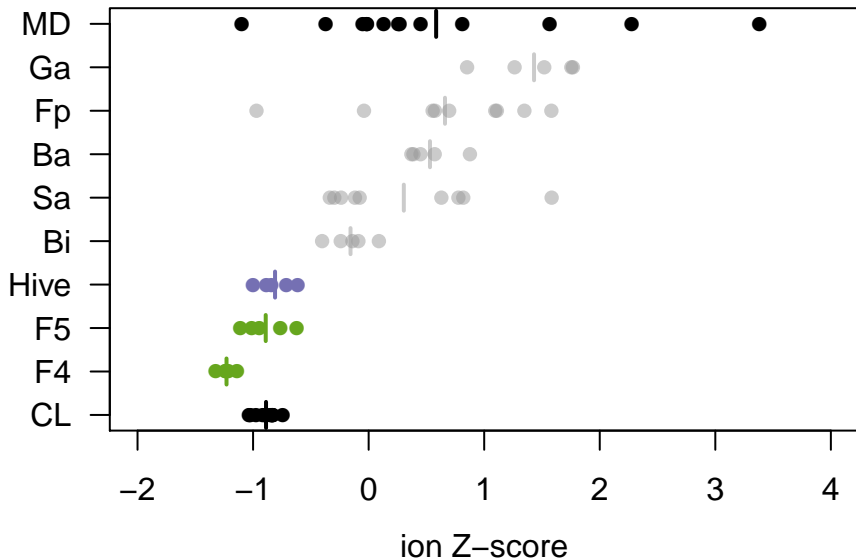

Supplement: S8 Data — (ZIP) [file pbio.2003467.s008.zip › Z-score_plots/349 microbial substrate Dethiobiotin.pdf]

Valerate\*

# 35 101.060 microbial product

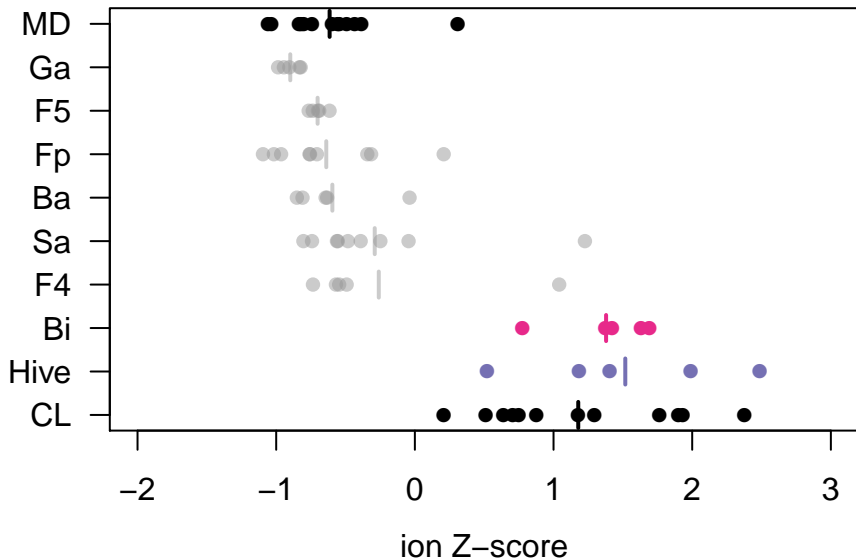

Supplement: S8 Data — (ZIP) [file pbio.2003467.s008.zip › Z-score_plots/35 microbial product Valerate.pdf]

# 3-Oxododecanoic acid

# 350 213.149 microbial product

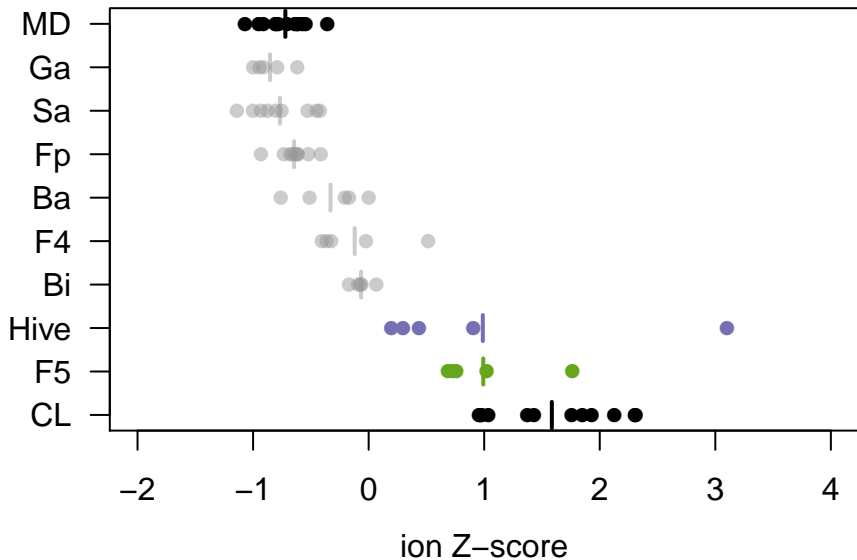

Supplement: S8 Data — (ZIP) [file pbio.2003467.s008.zip › Z-score_plots/350 microbial product 3-Oxododecanoic acid.pdf]

**2-Amino-9,10-epoxy-8-oxodecanoic acid**  
**# 353 214.108 microbial substrate**

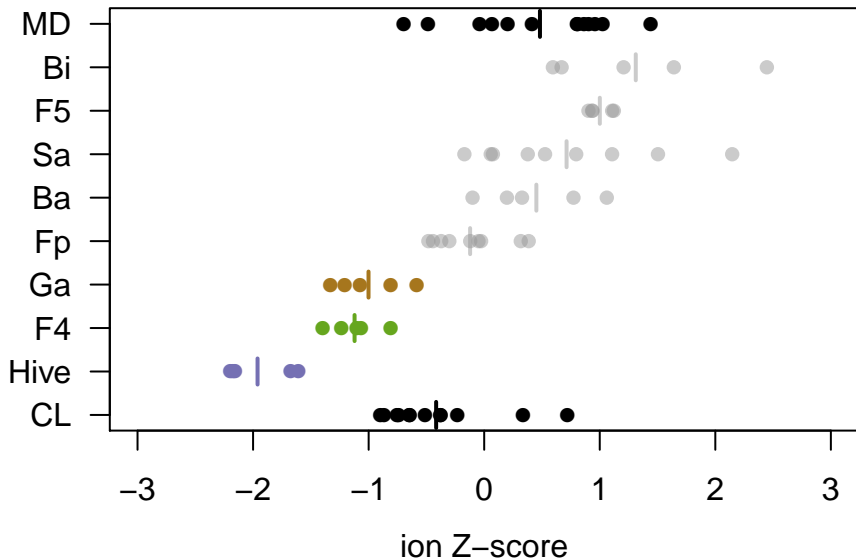

Supplement: S8 Data — (ZIP) [file pbio.2003467.s008.zip › Z-score_plots/353 microbial substrate 2-Amino-9,10-epoxy-8-oxodecanoic acid.pdf]

**12-Hydroxydodecanoic acid**  
**# 356 215.165 microbial substrate**

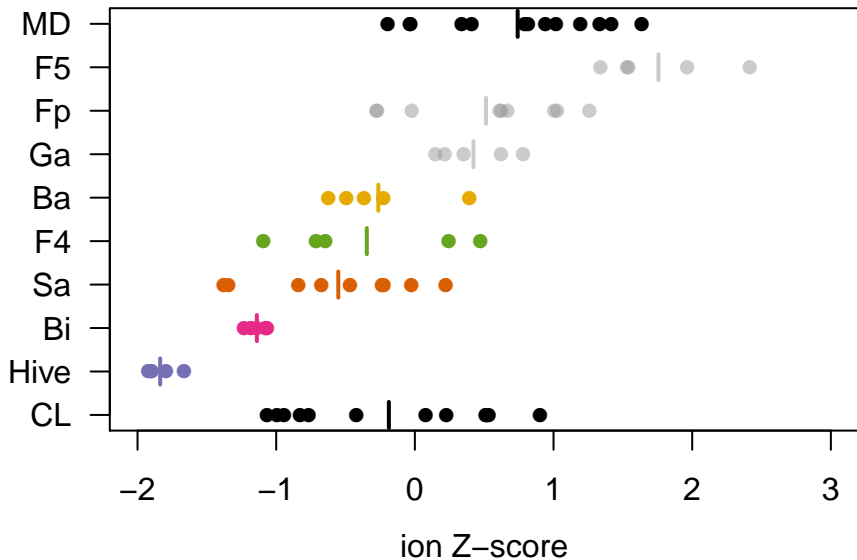

Supplement: S8 Data — (ZIP) [file pbio.2003467.s008.zip › Z-score_plots/356 microbial substrate 12-Hydroxydodecanoic acid.pdf]

# 2-(Hydroxymethyl)-3-(acetamidomethylene)succin

# 357 216.051 microbial product

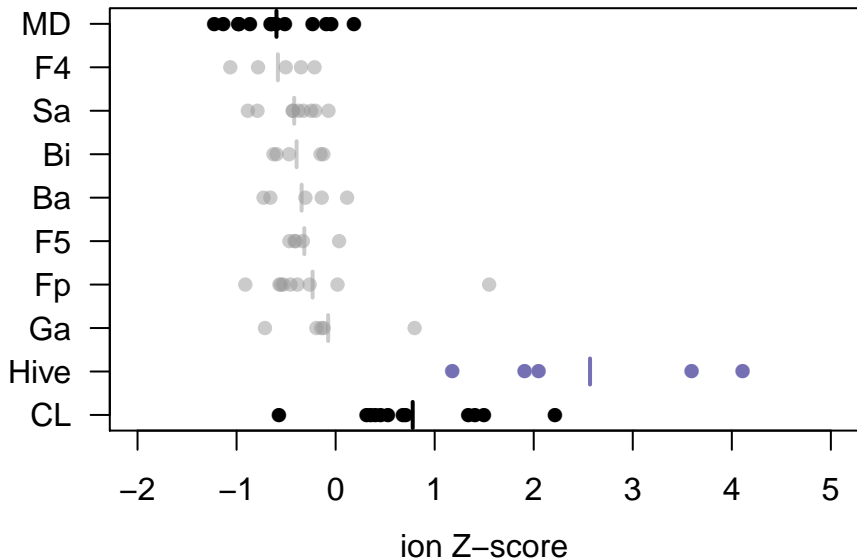

Supplement: S8 Data — (ZIP) [file pbio.2003467.s008.zip › Z-score_plots/357 microbial product 2-(Hydroxymethyl)-3-(acetamidomethylene)succinate.pdf]

**(S)-beta-Methylindolepyruvate\***  
**# 358 216.066 microbial product**

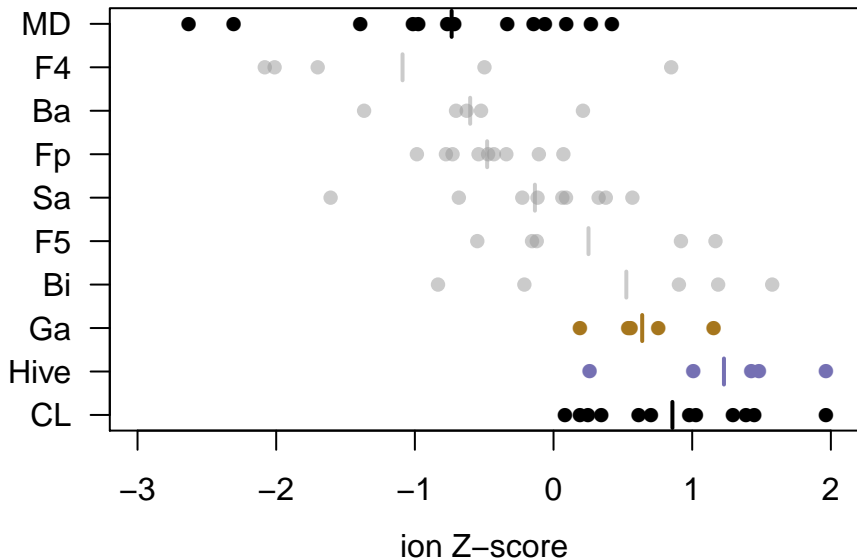

Supplement: S8 Data — (ZIP) [file pbio.2003467.s008.zip › Z-score_plots/358 microbial product (S)-beta-Methylindolepyruvate.pdf]

# O-Propanoylcarnitine

# 360 216.124 microbial substrate

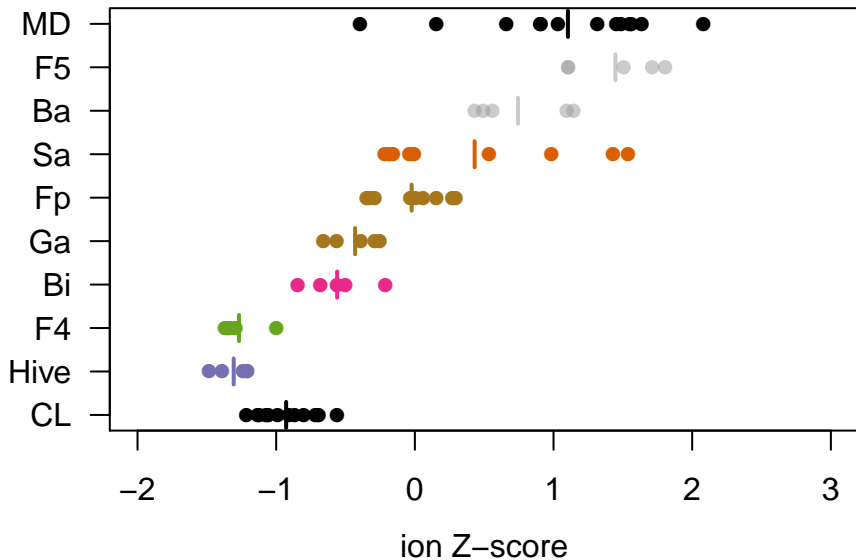

Supplement: S8 Data — (ZIP) [file pbio.2003467.s008.zip › Z-score_plots/360 microbial substrate O-Propanoylcarnitine.pdf]

**N-Acetylserotonin\***  
**# 363 217.099 microbial product**

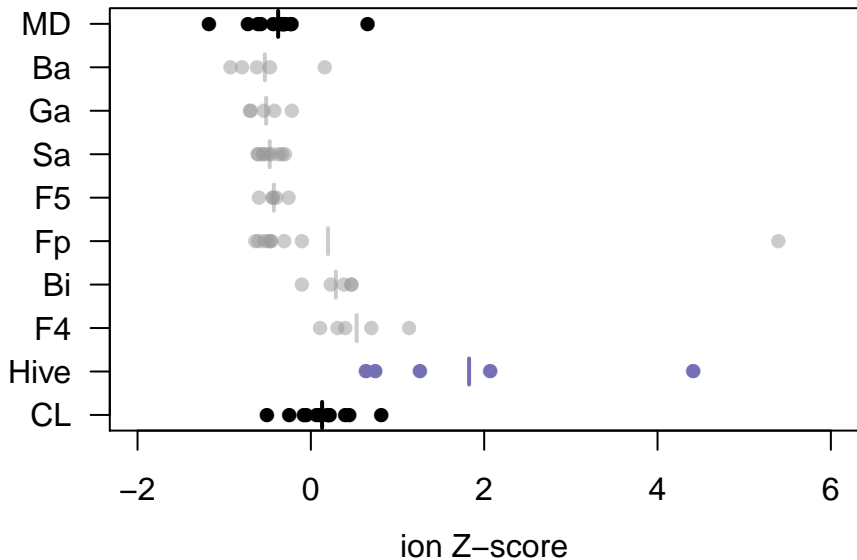

Supplement: S8 Data — (ZIP) [file pbio.2003467.s008.zip › Z-score_plots/363 microbial product N-Acetylserotonin.pdf]

# D-Lysopine

# 364 217.119 microbial substrate

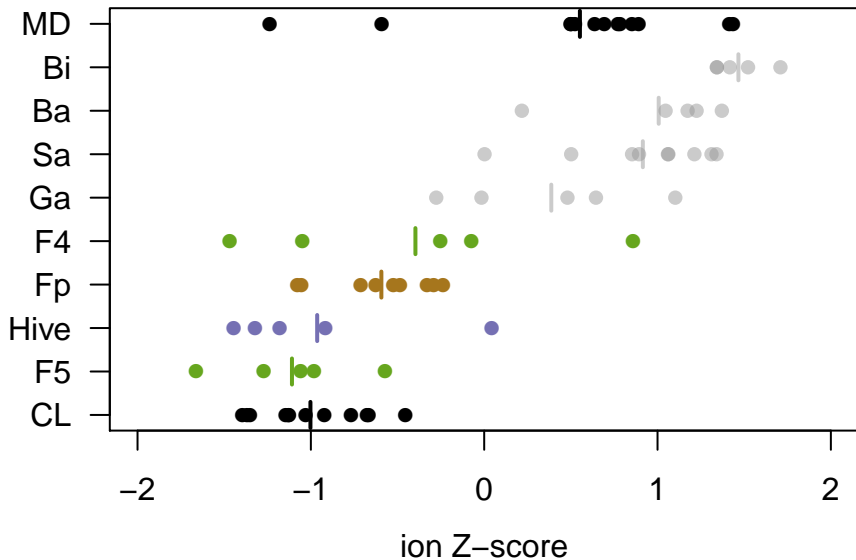

Supplement: S8 Data — (ZIP) [file pbio.2003467.s008.zip › Z-score_plots/364 microbial substrate D-Lysopine.pdf]

# Pantothenate

# 365 218.103 microbial substrate

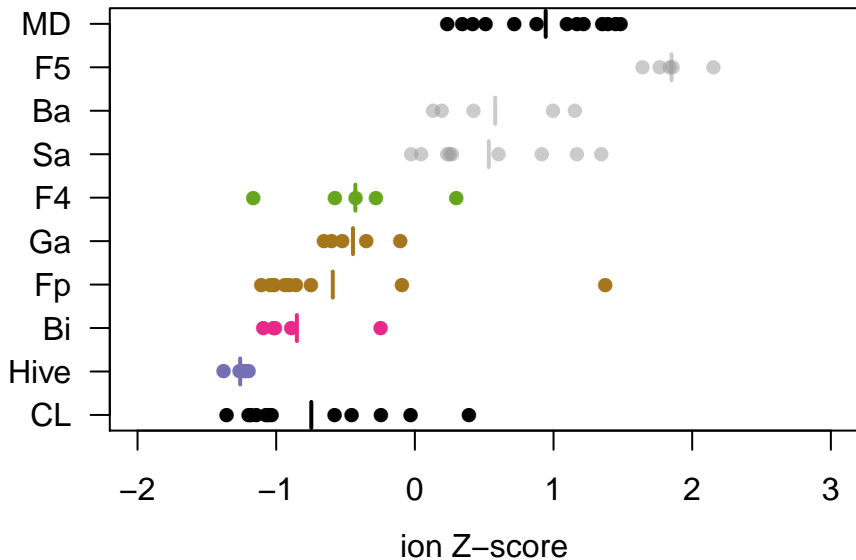

Supplement: S8 Data — (ZIP) [file pbio.2003467.s008.zip › Z-score_plots/365 microbial substrate Pantothenate.pdf]

# Pentahomomethionine

# 366 218.122 microbial substrate

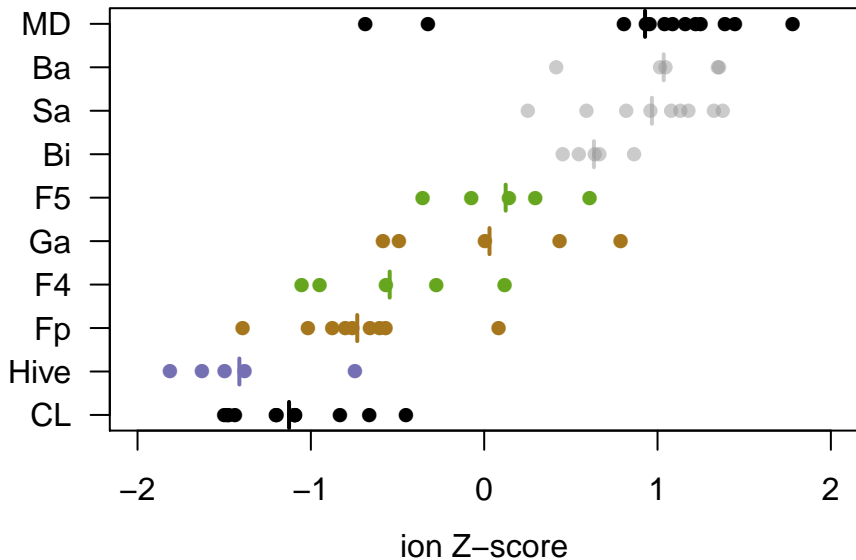

Supplement: S8 Data — (ZIP) [file pbio.2003467.s008.zip › Z-score_plots/366 microbial substrate Pentahomomethionine.pdf]

# 5-Hydroxy-L-tryptophan\*

# 367 219.077 microbial product

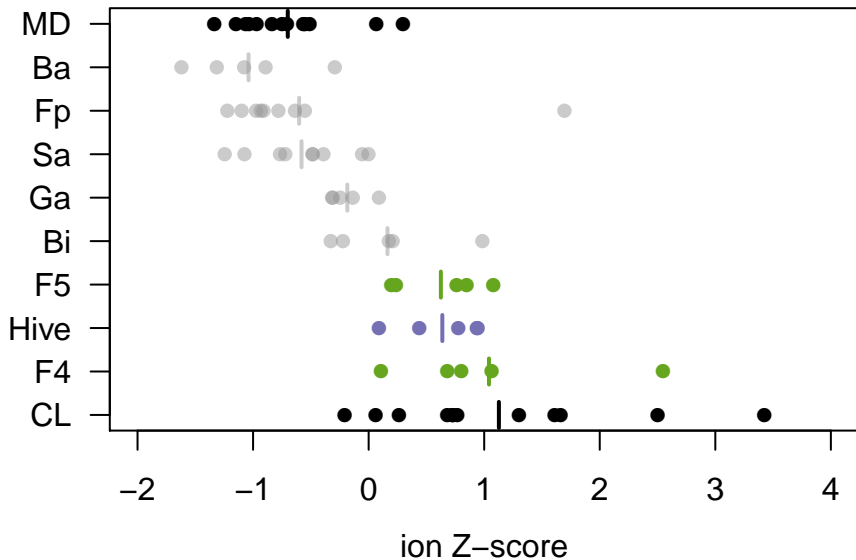

Supplement: S8 Data — (ZIP) [file pbio.2003467.s008.zip › Z-score_plots/367 microbial product 5-Hydroxy-L-tryptophan.pdf]

**N-Acetyl-D-glucosamine\***  
**# 370 220.083 microbial substrate**

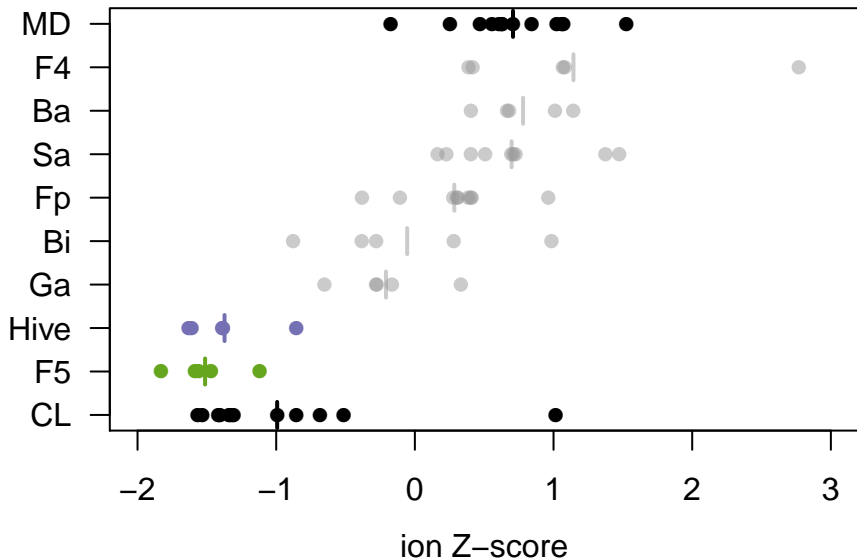

Supplement: S8 Data — (ZIP) [file pbio.2003467.s008.zip › Z-score_plots/370 microbial substrate N-Acetyl-D-glucosamine.pdf]

# Formetanate

# 371 220.108 microbial substrate

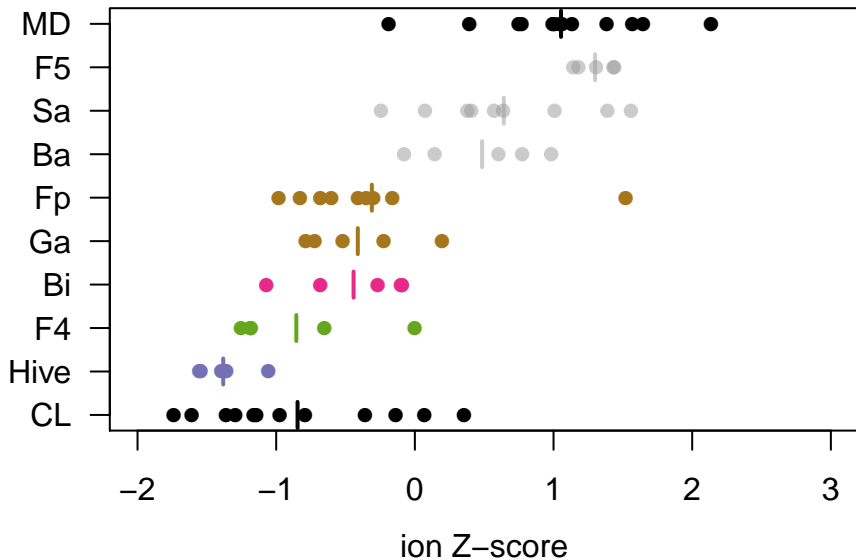

Supplement: S8 Data — (ZIP) [file pbio.2003467.s008.zip › Z-score_plots/371 microbial substrate Formetanate.pdf]

**Dehydrovomifoliol\***  
**# 374 221.118 microbial substrate**

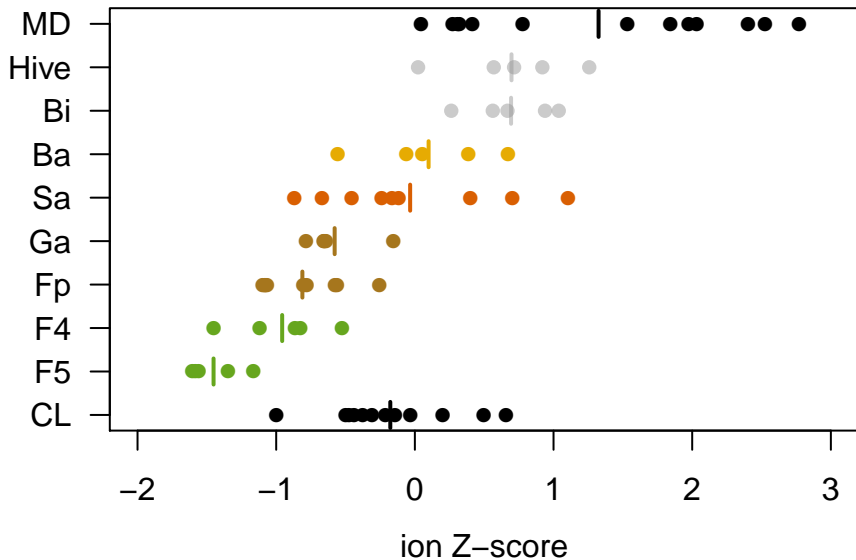

Supplement: S8 Data — (ZIP) [file pbio.2003467.s008.zip › Z-score_plots/374 microbial substrate Dehydrovomifoliol.pdf]

# Rishitin\*

# 375 221.154 microbial substrate

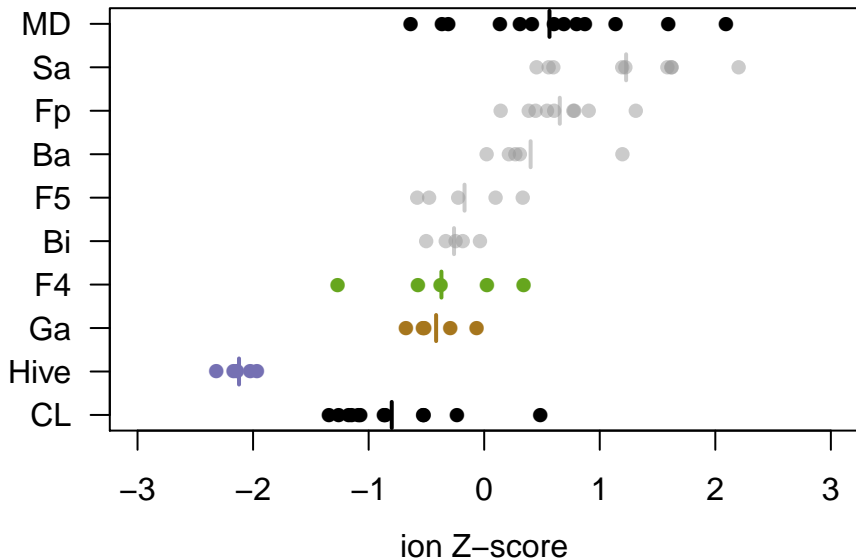

Supplement: S8 Data — (ZIP) [file pbio.2003467.s008.zip › Z-score_plots/375 microbial substrate Rishitin.pdf]

**4-Hydroxybutyric acid\***  
**# 38 103.039 microbial product**

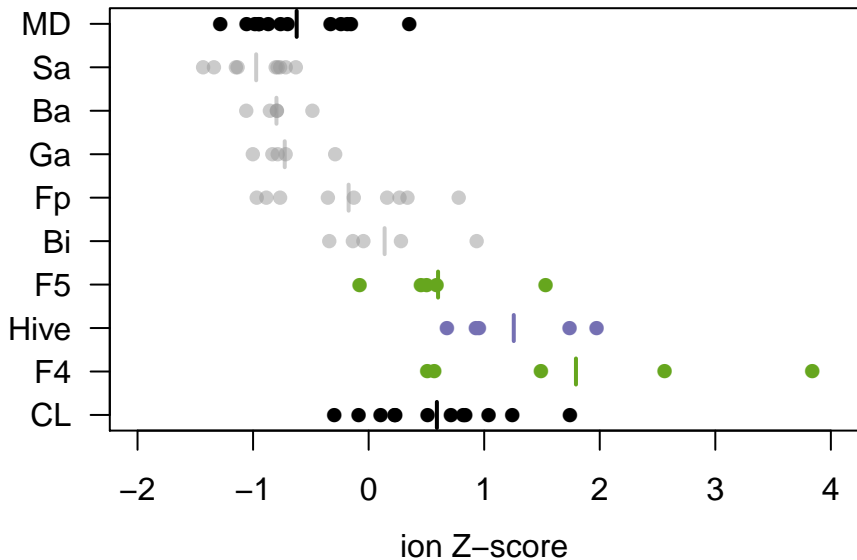

Supplement: S8 Data — (ZIP) [file pbio.2003467.s008.zip › Z-score_plots/38 microbial product 4-Hydroxybutyric acid.pdf]

**1-(Methylnitrosoamino)-4-(3-pyridinyl)-1,4-butane**  
**# 380 224.104 microbial product**

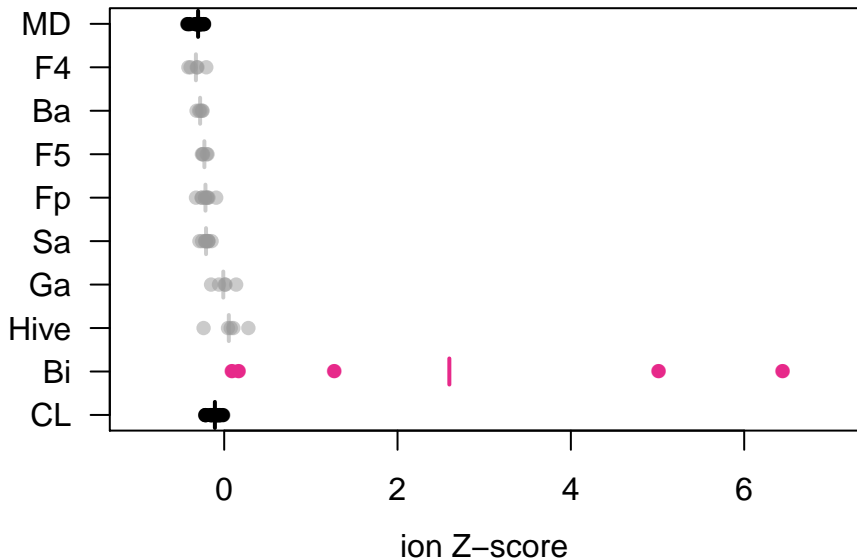

Supplement: S8 Data — (ZIP) [file pbio.2003467.s008.zip › Z-score_plots/380 microbial product 1-(Methylnitrosoamino)-4-(3-pyridinyl)-1,4-butanediol.pdf]

**Pentadecanal**  
**# 385 225.222 microbial substrate**

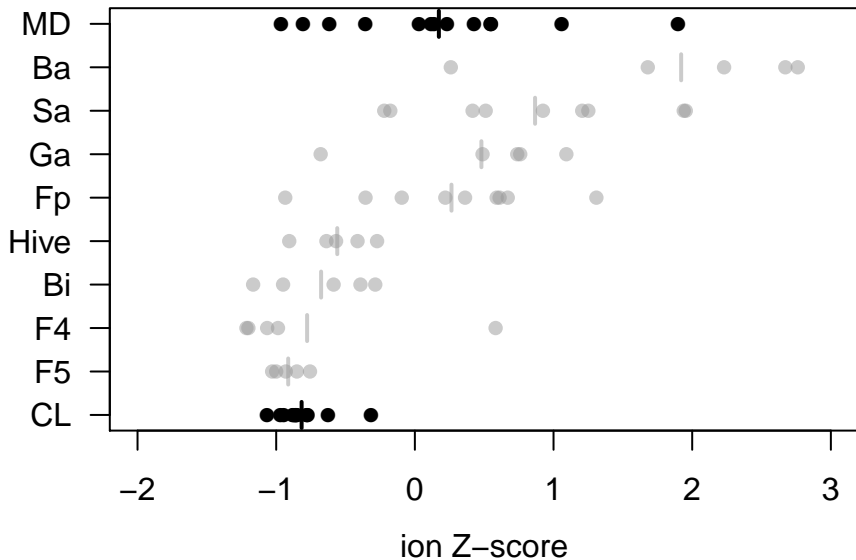

Supplement: S8 Data — (ZIP) [file pbio.2003467.s008.zip › Z-score_plots/385 microbial substrate Pentadecanal.pdf]

# Deoxycytidine

# 387 226.083 microbial substrate

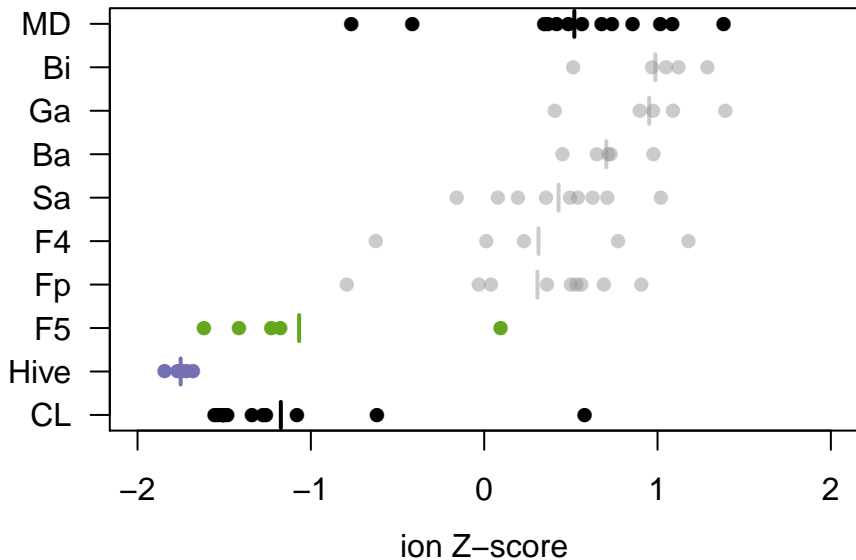

Supplement: S8 Data — (ZIP) [file pbio.2003467.s008.zip › Z-score_plots/387 microbial substrate Deoxycytidine.pdf]

# Euxanthone

# 388 227.035 microbial substrate

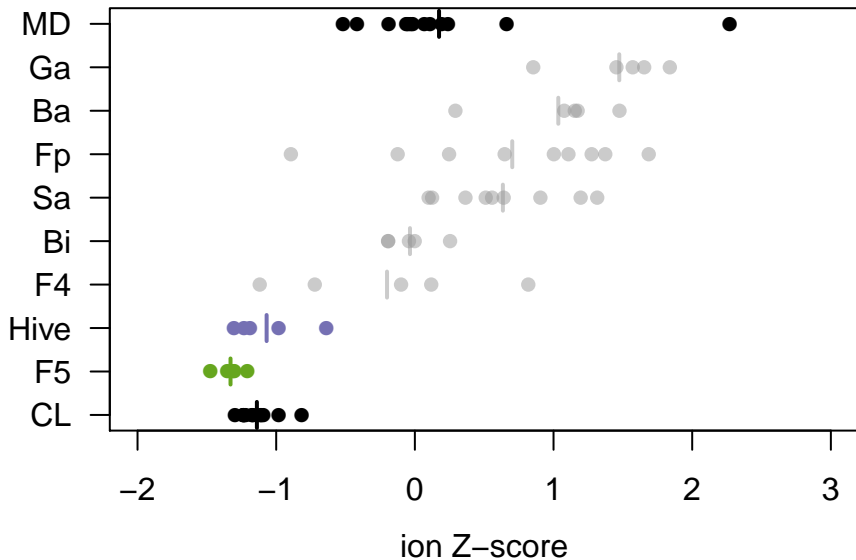

Supplement: S8 Data — (ZIP) [file pbio.2003467.s008.zip › Z-score_plots/388 microbial substrate Euxanthone.pdf]

# Deoxyuridine

# 389 227.067 microbial substrate

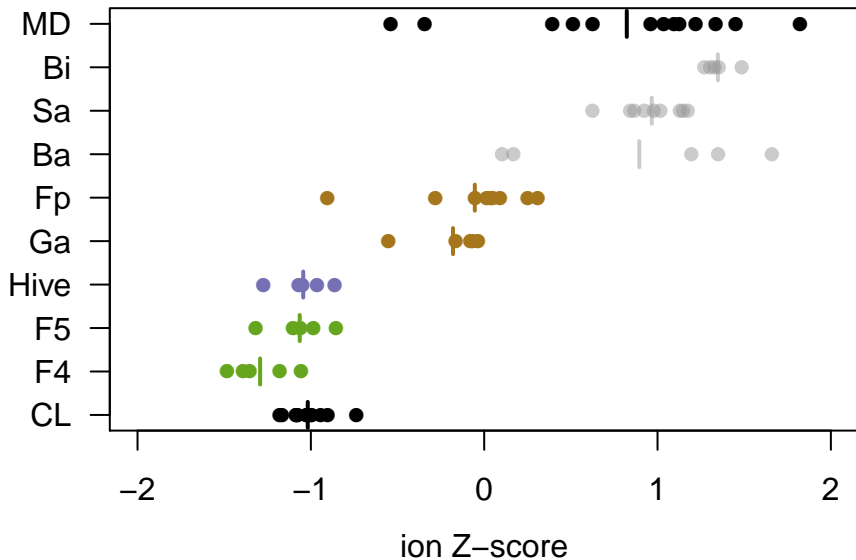

Supplement: S8 Data — (ZIP) [file pbio.2003467.s008.zip › Z-score_plots/389 microbial substrate Deoxyuridine.pdf]

# Trioxsalen\*

# 390 227.071 microbial substrate

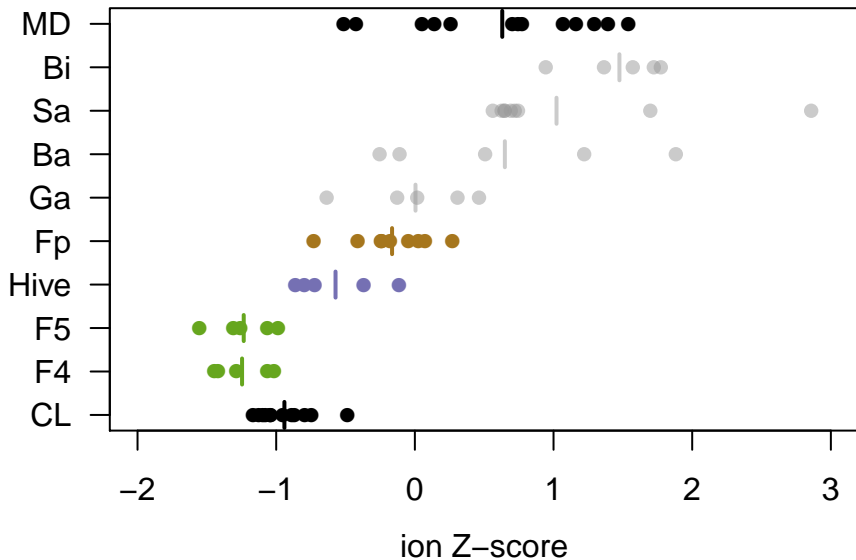

Supplement: S8 Data — (ZIP) [file pbio.2003467.s008.zip › Z-score_plots/390 microbial substrate Trioxsalen.pdf]

# Traumatic acid

# 391 227.129 microbial product

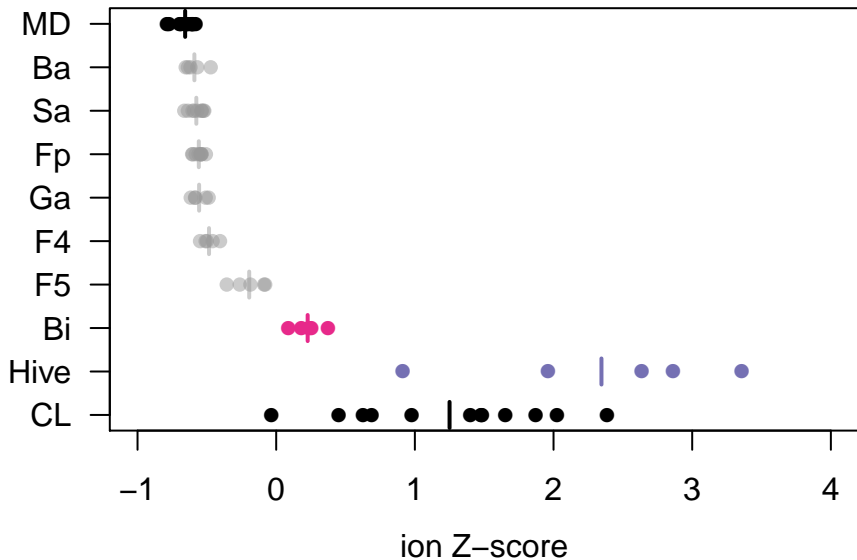

Supplement: S8 Data — (ZIP) [file pbio.2003467.s008.zip › Z-score_plots/391 microbial product Traumatic acid.pdf]

**D-Ribose 5-phosphate\***  
**# 394 229.011 microbial product**

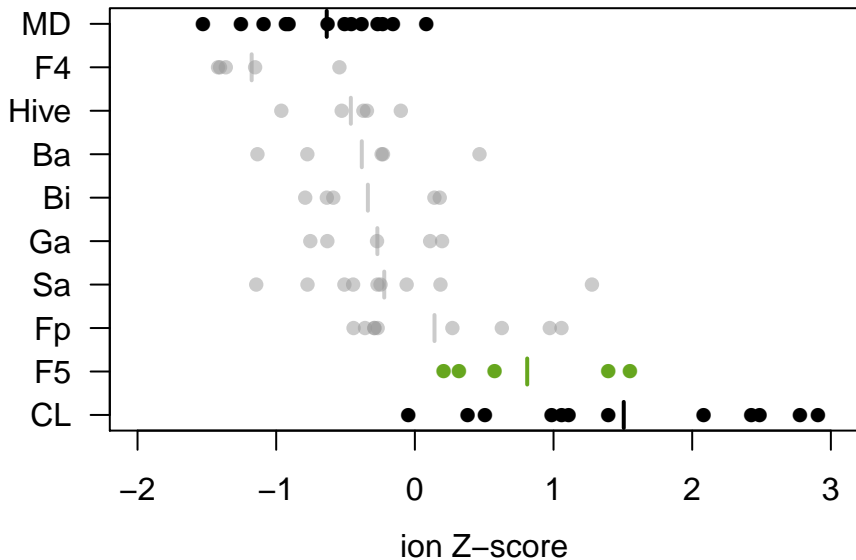

Supplement: S8 Data — (ZIP) [file pbio.2003467.s008.zip › Z-score_plots/394 microbial product D-Ribose 5-phosphate.pdf]

**Dodecanedioic acid\***  
**# 396 229.144 microbial product**

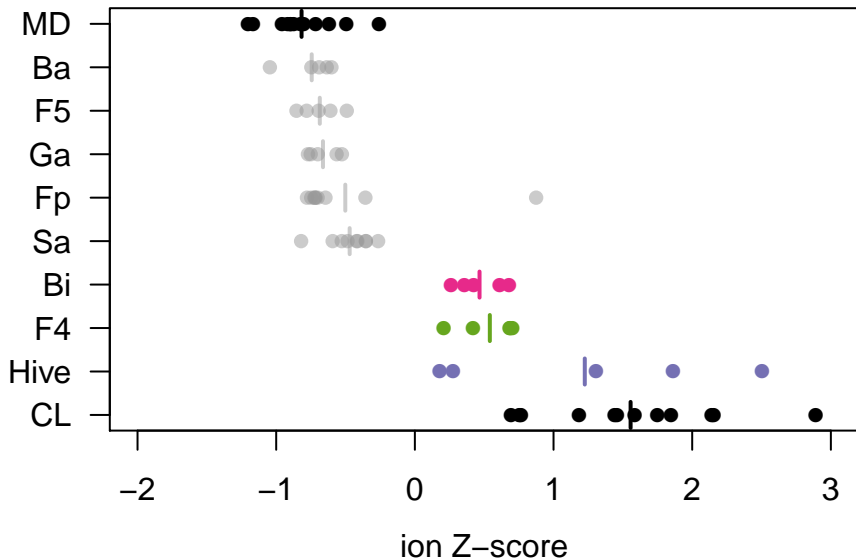

Supplement: S8 Data — (ZIP) [file pbio.2003467.s008.zip › Z-score_plots/396 microbial product Dodecanedioic acid.pdf]

# N-Phosphohypotaurocyamine

# 397 229.999 microbial product

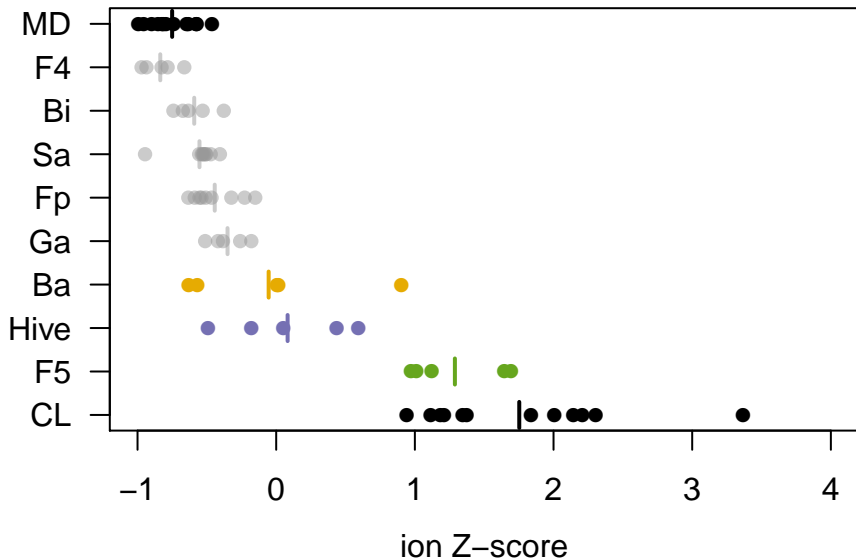

Supplement: S8 Data — (ZIP) [file pbio.2003467.s008.zip › Z-score_plots/397 microbial product N-Phosphohypotaurocyamine.pdf]

**N-Acetyl-L-2-amino-6-oxopimelate\***  
**# 398 230.067 microbial product**

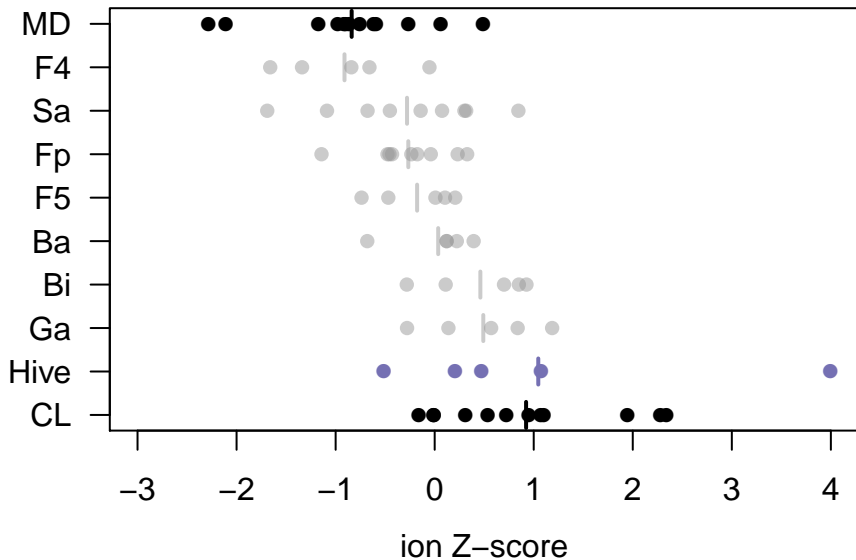

Supplement: S8 Data — (ZIP) [file pbio.2003467.s008.zip › Z-score_plots/398 microbial product N-Acetyl-L-2-amino-6-oxopimelate.pdf]

**O-Butanoylcarnitine\***  
**# 399 230.140 microbial substrate**

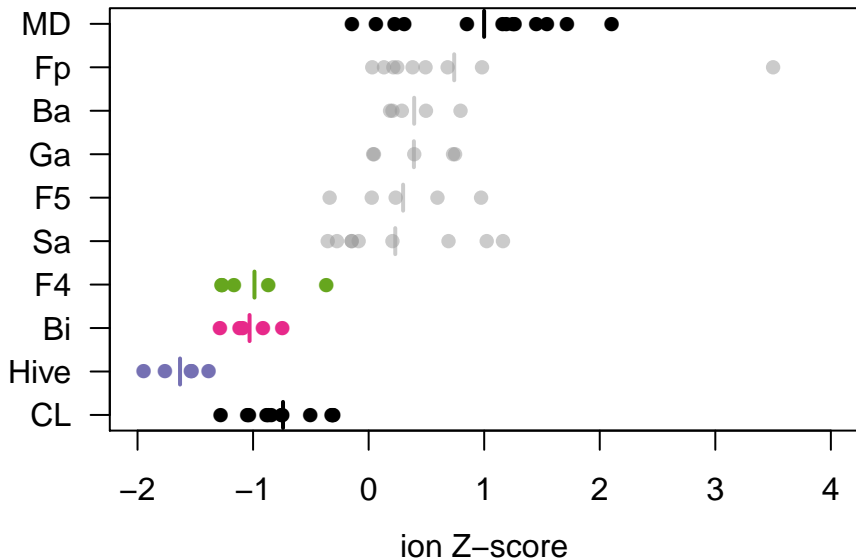

Supplement: S8 Data — (ZIP) [file pbio.2003467.s008.zip › Z-score_plots/399 microbial substrate O-Butanoylcarnitine.pdf]

**Aminoacetaldehyde\***  
**# 4 58.0293 microbial substrate**

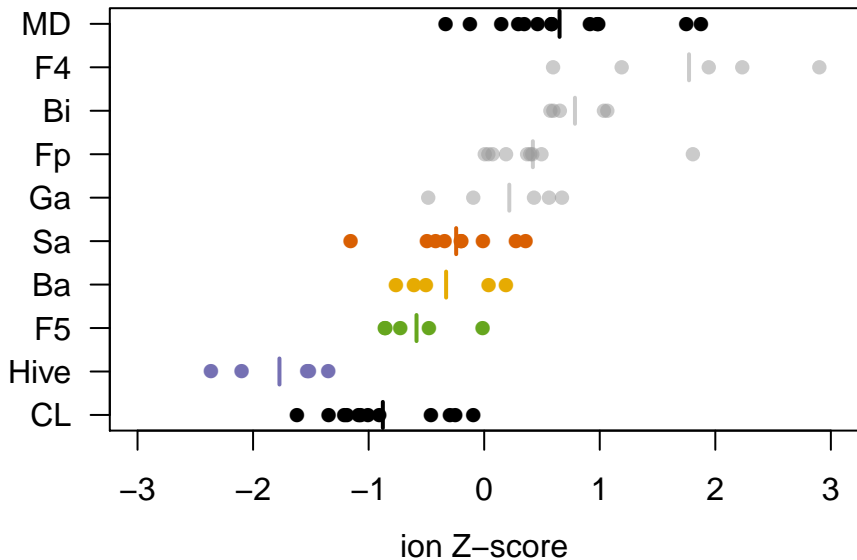

Supplement: S8 Data — (ZIP) [file pbio.2003467.s008.zip › Z-score_plots/4 microbial substrate Aminoacetaldehyde.pdf]

**p-Coumaroylputrescine\***  
**# 405 233.129 microbial substrate**

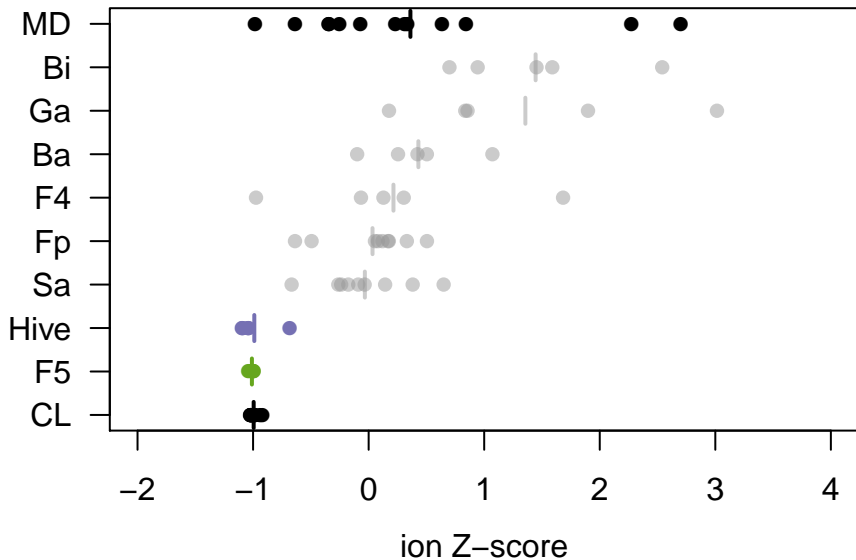

Supplement: S8 Data — (ZIP) [file pbio.2003467.s008.zip › Z-score_plots/405 microbial substrate p-Coumaroylputrescine.pdf]

**2-Imino-3-(7-chloroindol-3-yl)propanoate**  
**# 411 235.028 microbial substrate**

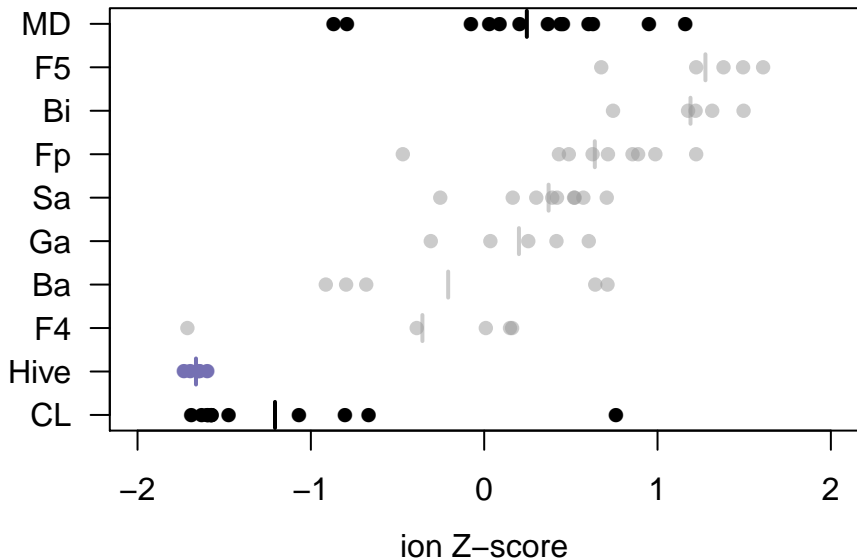

Supplement: S8 Data — (ZIP) [file pbio.2003467.s008.zip › Z-score_plots/411 microbial substrate 2-Imino-3-(7-chloroindol-3-yl)propanoate.pdf]

# Procaine\*

# 416 235.145 microbial product

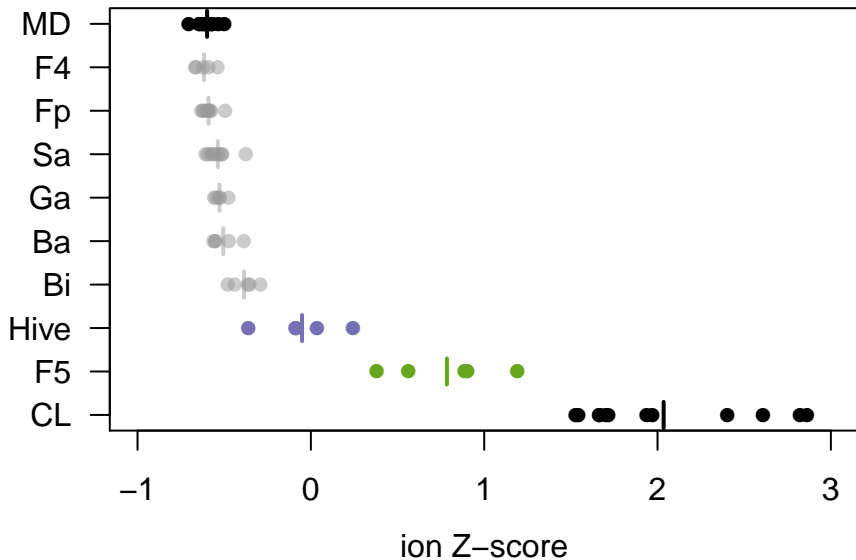

Supplement: S8 Data — (ZIP) [file pbio.2003467.s008.zip › Z-score_plots/416 microbial product Procaine.pdf]

**N-Acetyl-D-glucosamine\***  
**# 418 236.078 microbial substrate**

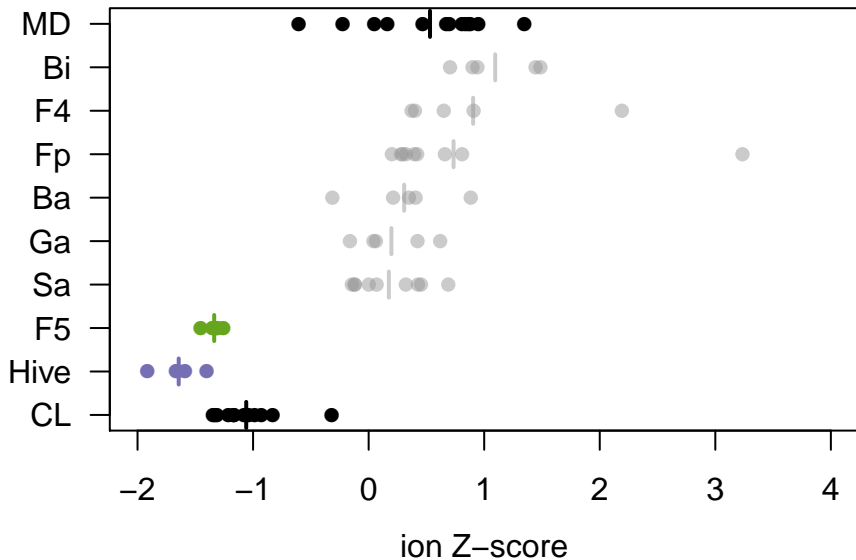

Supplement: S8 Data — (ZIP) [file pbio.2003467.s008.zip › Z-score_plots/418 microbial substrate N-Acetyl-D-glucosaminate.pdf]

# Nitrofurantoin

# 419 237.026 microbial substrate

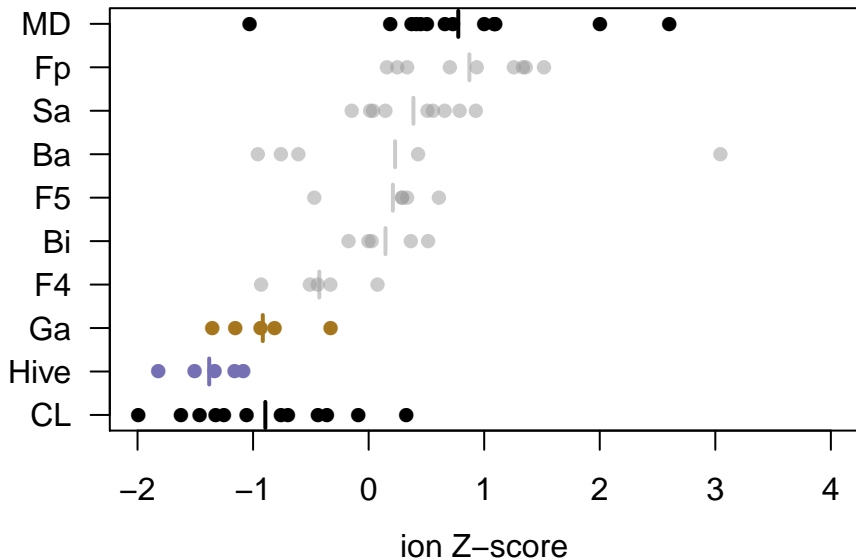

Supplement: S8 Data — (ZIP) [file pbio.2003467.s008.zip › Z-score_plots/419 microbial substrate Nitrofurantoin.pdf]

# Xanthopterin-B2

# 420 237.062 microbial substrate

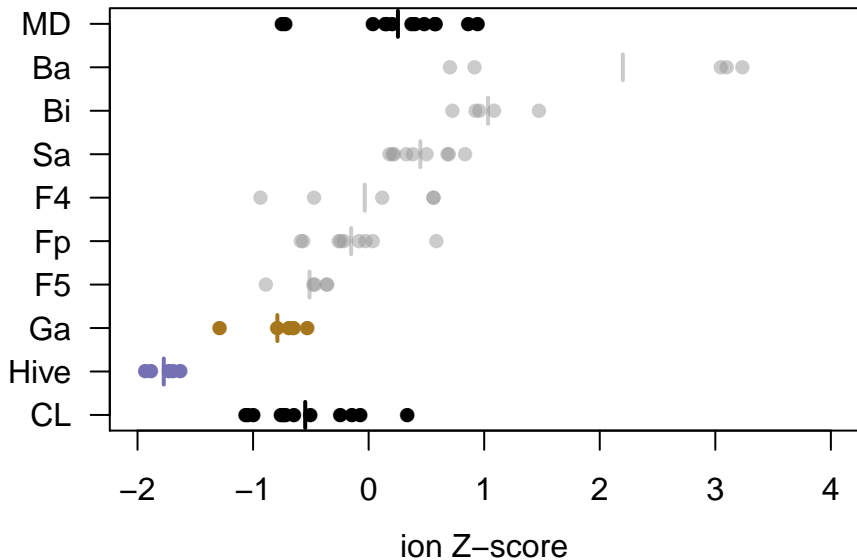

Supplement: S8 Data — (ZIP) [file pbio.2003467.s008.zip › Z-score_plots/420 microbial substrate Xanthopterin-B2.pdf]

**4S,7aS)-7a-Methyl-1,5-dioxo-octahydro-1H-inden-4-**  
**# 422 237.111 microbial product**

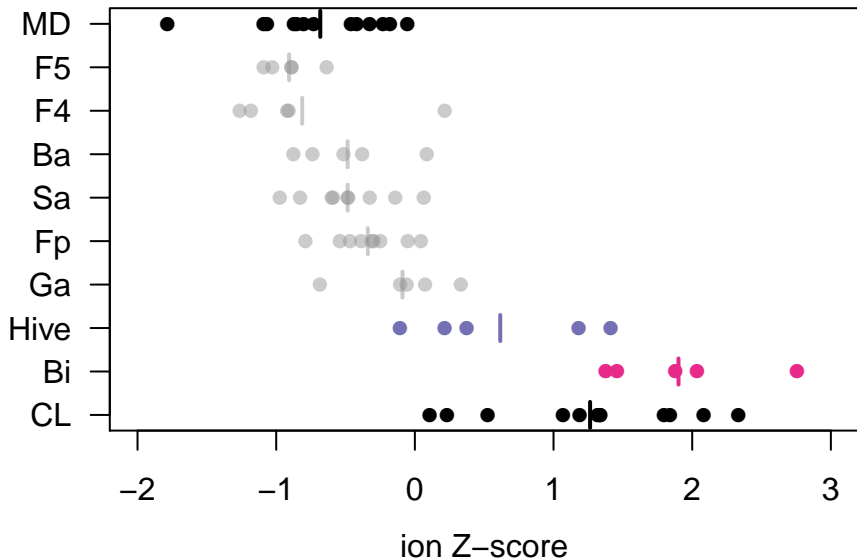

Supplement: S8 Data — (ZIP) [file pbio.2003467.s008.zip › Z-score_plots/422 microbial product 3-[(3aS,4S,7aS)-7a-Methyl-1,5-dioxo-octahydro-1H-inden-4-yl]propanoate.pdf]

**N-(3-Oxo-octanoyl)homoserine lactone**  
**# 426 240.124 microbial substrate**

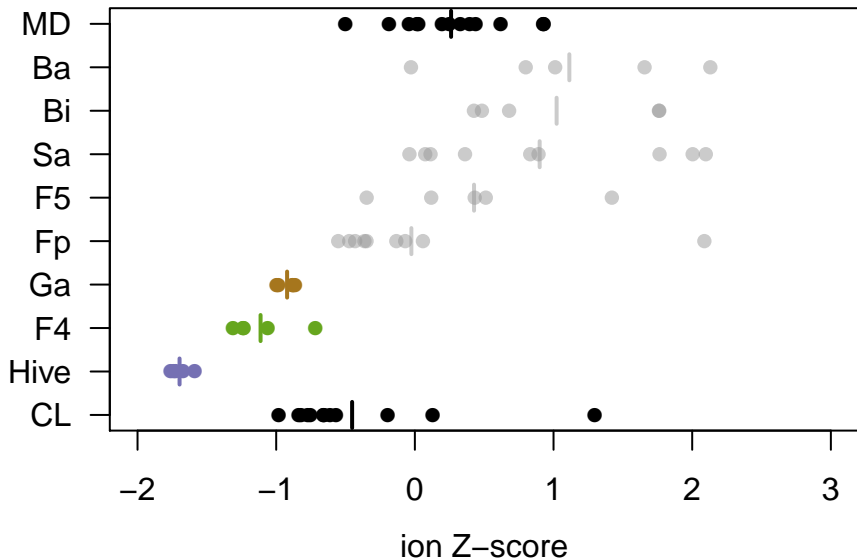

Supplement: S8 Data — (ZIP) [file pbio.2003467.s008.zip › Z-score_plots/426 microbial substrate N-(3-Oxooctanoyl)homoserine lactone.pdf]

**D-myo-Inositol 1,2-cyclic phosphate\***  
**# 427 241.012 microbial substrate**

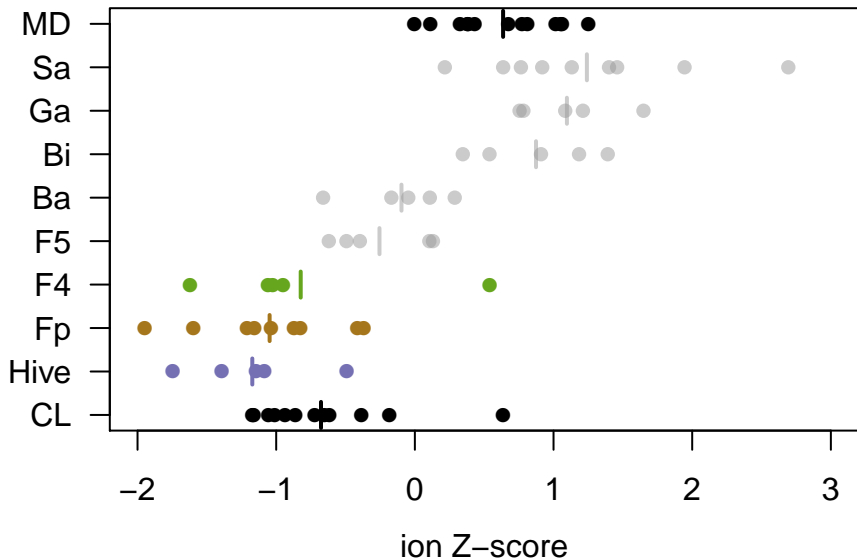

Supplement: S8 Data — (ZIP) [file pbio.2003467.s008.zip › Z-score_plots/427 microbial substrate D-myo-Inositol 1,2-cyclic phosphate.pdf]

# Thymidine\*

# 429 241.083 microbial substrate

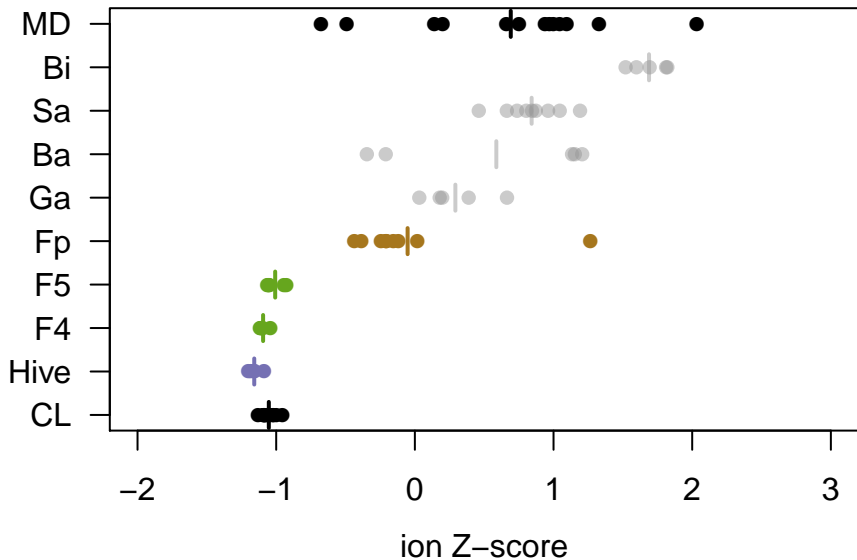

Supplement: S8 Data — (ZIP) [file pbio.2003467.s008.zip › Z-score_plots/429 microbial substrate Thymidine.pdf]

**Benzyl alcohol\***  
**# 43 107.050 microbial product**

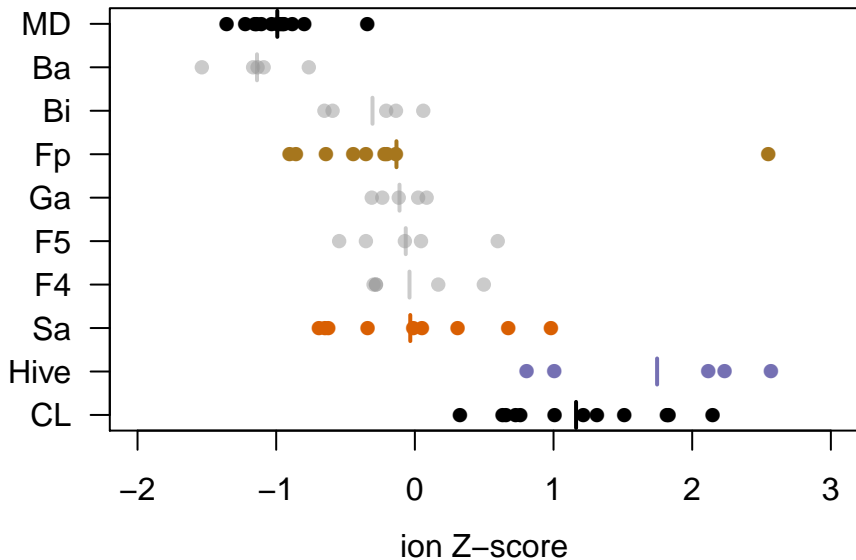

Supplement: S8 Data — (ZIP) [file pbio.2003467.s008.zip › Z-score_plots/43 microbial product Benzyl alcohol.pdf]

# Diphenylcarbazide

# 430 241.108 microbial substrate

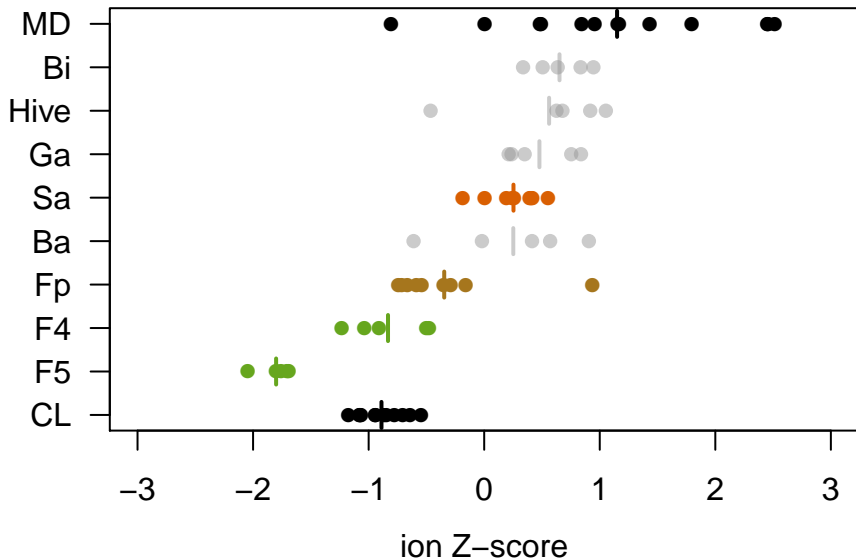

Supplement: S8 Data — (ZIP) [file pbio.2003467.s008.zip › Z-score_plots/430 microbial substrate Diphenylcarbazide.pdf]

Uridine\*

# 432 243.062 microbial substrate

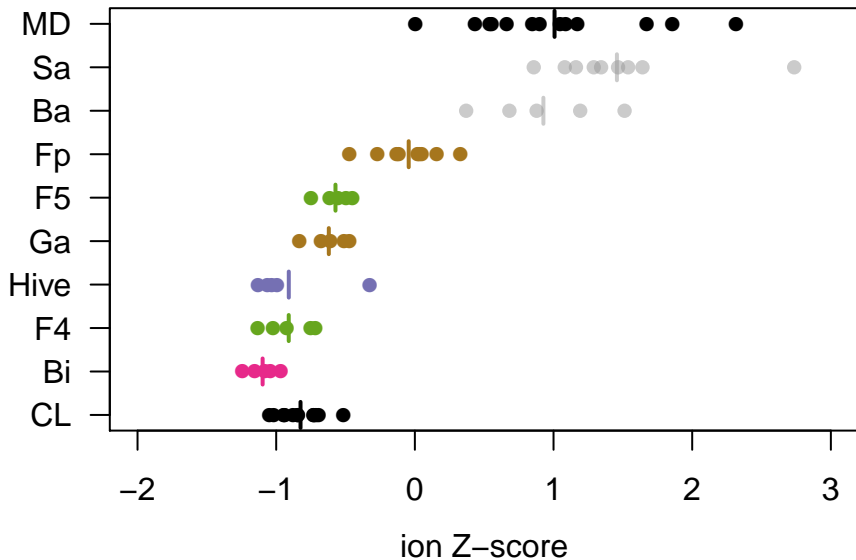

Supplement: S8 Data — (ZIP) [file pbio.2003467.s008.zip › Z-score_plots/432 microbial substrate Uridine.pdf]

**2S-Hydroxytetradecanoic acid**  
**# 434 243.196 microbial substrate**

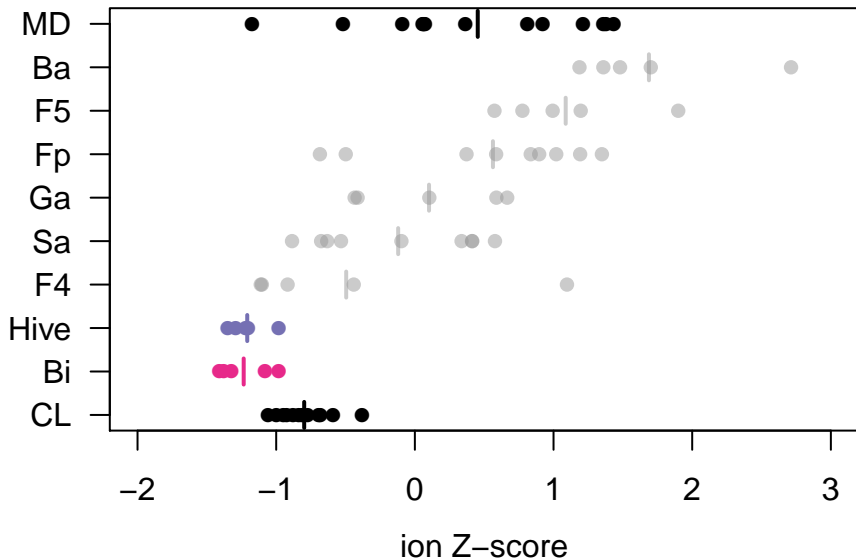

Supplement: S8 Data — (ZIP) [file pbio.2003467.s008.zip › Z-score_plots/434 microbial substrate 2S-Hydroxytetradecanoic acid.pdf]

# Isovalerylcarnitine

# 436 244.155 microbial substrate

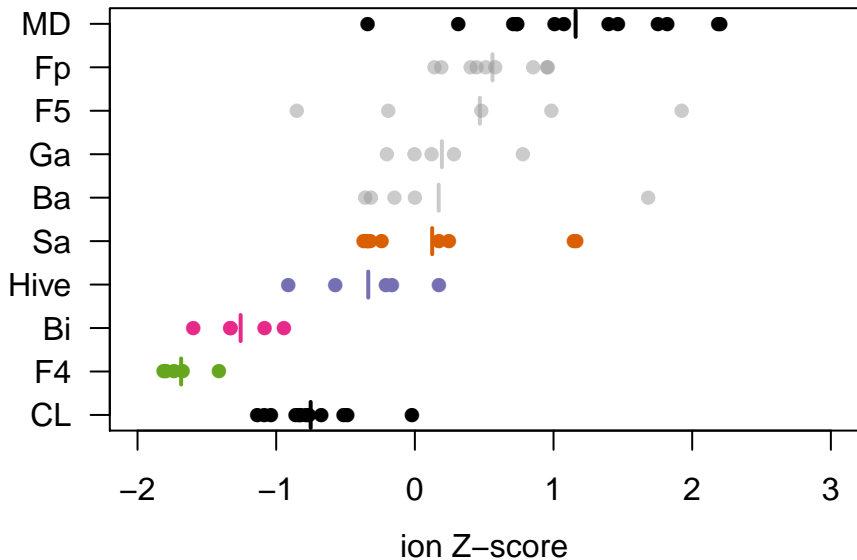

Supplement: S8 Data — (ZIP) [file pbio.2003467.s008.zip › Z-score_plots/436 microbial substrate Isovalerylcarnitine.pdf]

# Glycerophosphoglycerol

# 437 245.043 microbial product

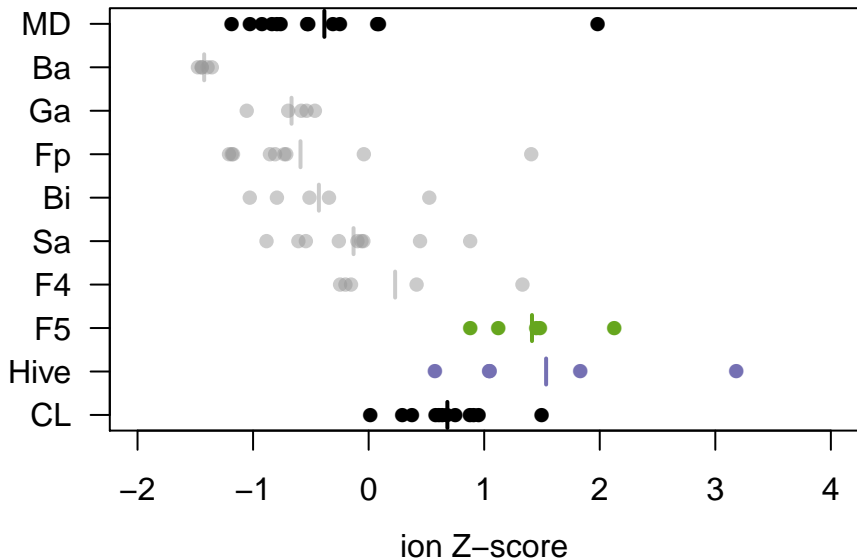

Supplement: S8 Data — (ZIP) [file pbio.2003467.s008.zip › Z-score_plots/437 microbial product Glycerophosphoglycerol.pdf]

# Dubamine

# 444 248.071 microbial substrate

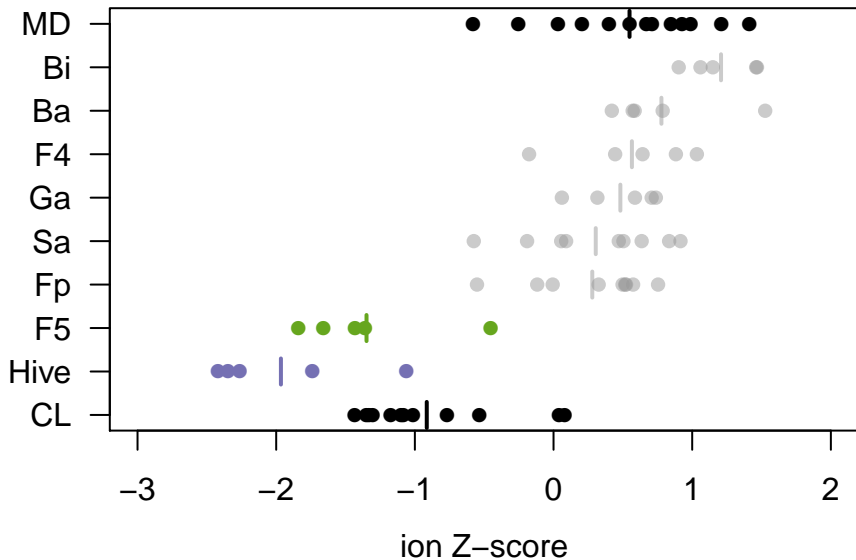

Supplement: S8 Data — (ZIP) [file pbio.2003467.s008.zip › Z-score_plots/444 microbial substrate Dubamine.pdf]

**Diisopropyl phthalate\***  
**# 447 249.112 microbial product**

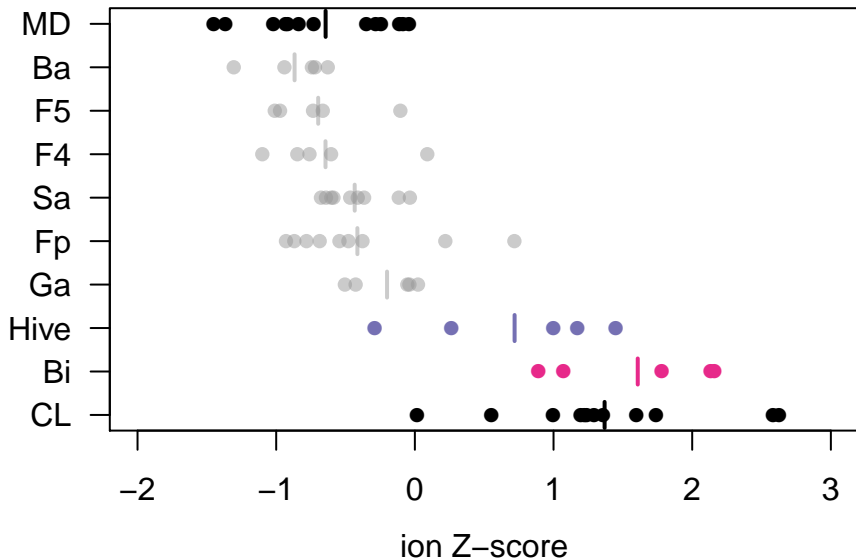

Supplement: S8 Data — (ZIP) [file pbio.2003467.s008.zip › Z-score_plots/447 microbial product Diisopropyl phthalate.pdf]

**N-Caffeoylputrescine\***  
**# 448 249.124 microbial substrate**

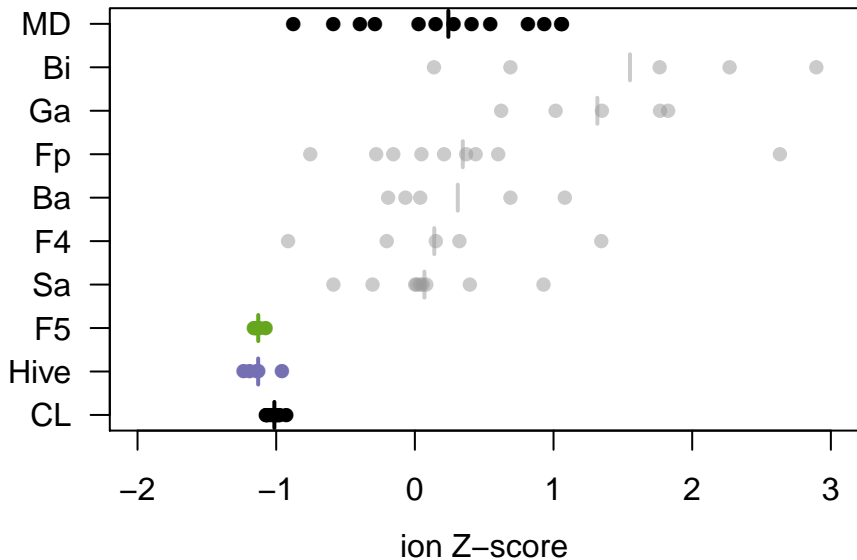

Supplement: S8 Data — (ZIP) [file pbio.2003467.s008.zip › Z-score_plots/448 microbial substrate N-Caffeoylputrescine.pdf]

**Muramic acid**  
**# 452 250.093 microbial substrate**

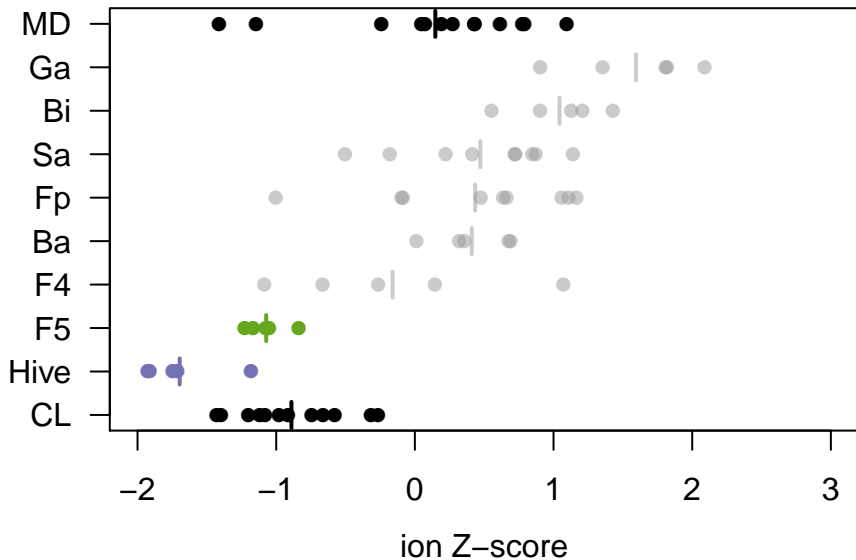

Supplement: S8 Data — (ZIP) [file pbio.2003467.s008.zip › Z-score_plots/452 microbial substrate Muramic acid.pdf]

# Prosulfocarb

# 454 250.128 microbial substrate

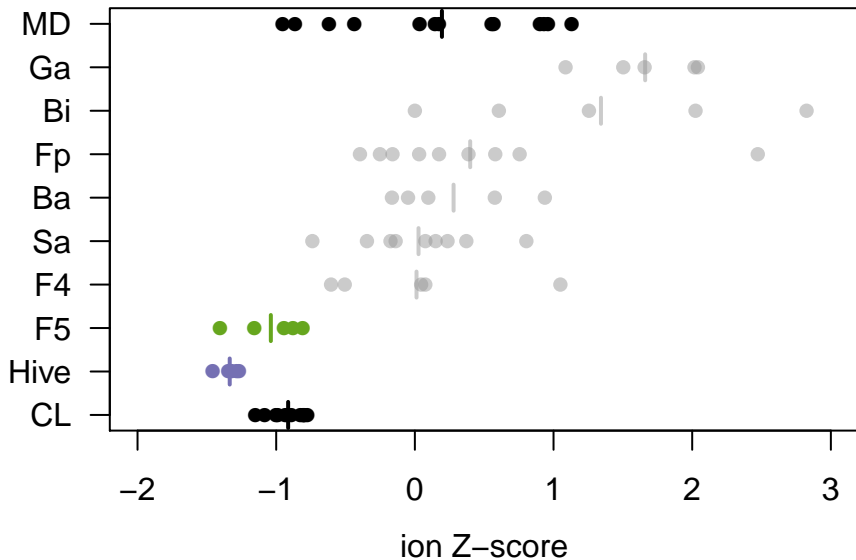

Supplement: S8 Data — (ZIP) [file pbio.2003467.s008.zip › Z-score_plots/454 microbial substrate Prosulfocarb.pdf]

# Nebularine\*

# 455 251.078 microbial substrate

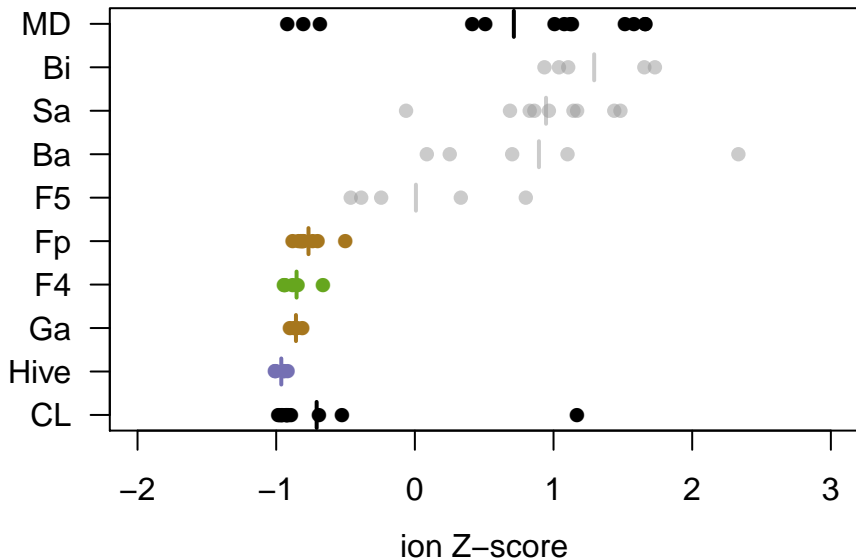

Supplement: S8 Data — (ZIP) [file pbio.2003467.s008.zip › Z-score_plots/455 microbial substrate Nebularine.pdf]

**2-Hydroxyfelbamate\***  
**# 462 253.083 microbial substrate**

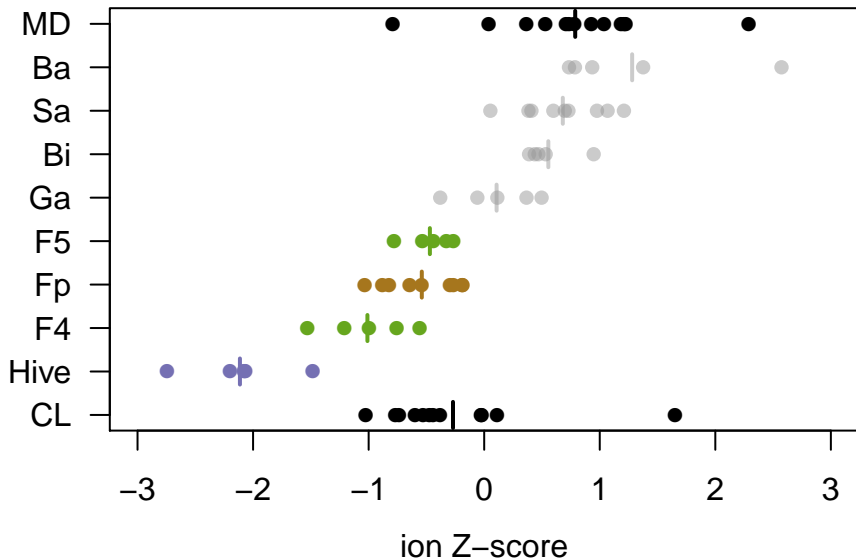

Supplement: S8 Data — (ZIP) [file pbio.2003467.s008.zip › Z-score_plots/462 microbial substrate 2-Hydroxyfelbamate.pdf]

**3-beta-D-Galactosyl-sn-glycerol\***  
**# 463 253.092 microbial substrate**

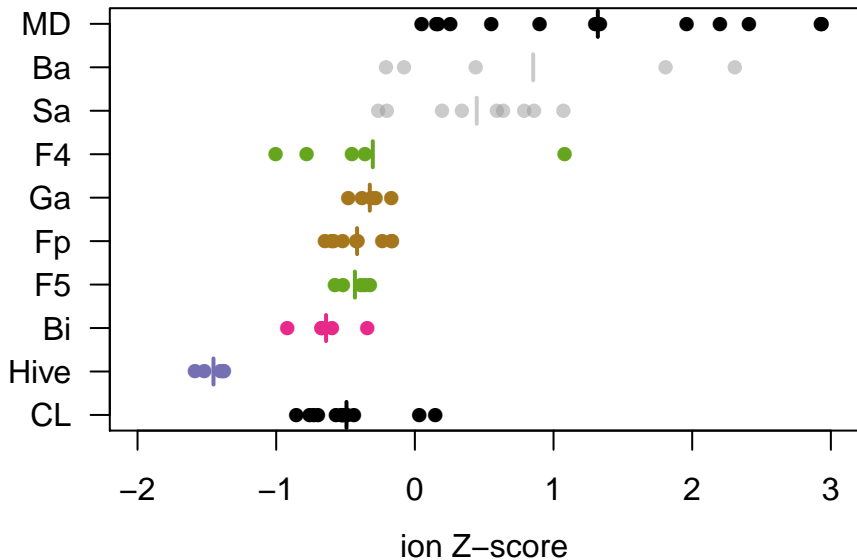

Supplement: S8 Data — (ZIP) [file pbio.2003467.s008.zip › Z-score_plots/463 microbial substrate 3-beta-D-Galactosyl-sn-glycerol.pdf]

# Kikkanol A\*

# 465 253.180 microbial product

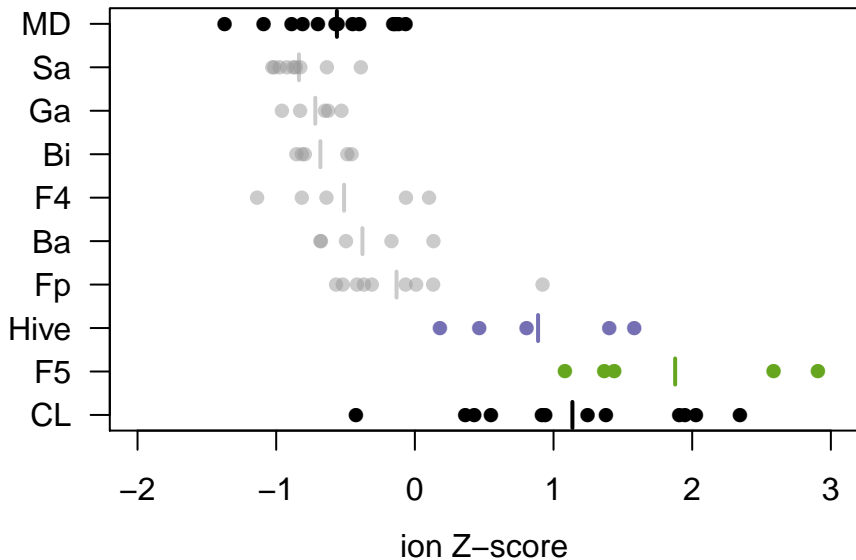

Supplement: S8 Data — (ZIP) [file pbio.2003467.s008.zip › Z-score_plots/465 microbial product Kikkanol A.pdf]

# N-D-Glucosylarylamine

# 467 254.103 microbial product

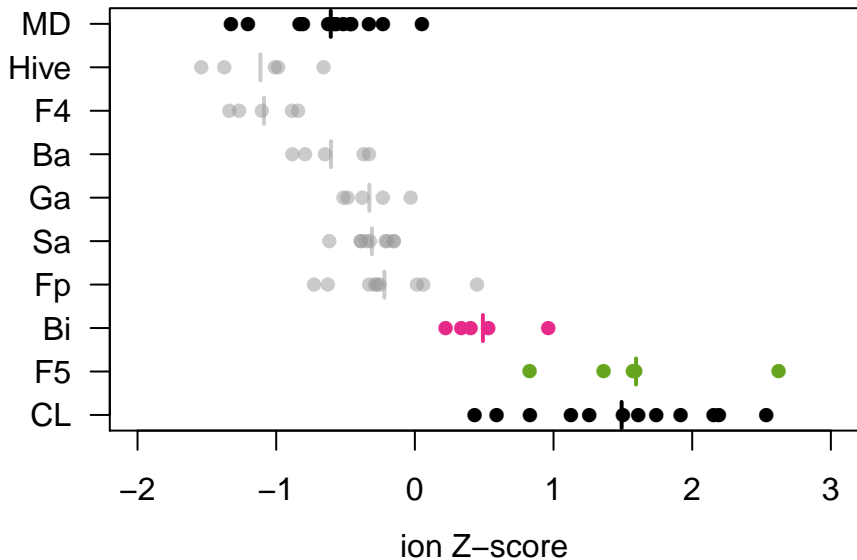

Supplement: S8 Data — (ZIP) [file pbio.2003467.s008.zip › Z-score_plots/467 microbial product N-D-Glucosylarylamine.pdf]

# Anthragallol\*

# 469 255.030 microbial substrate

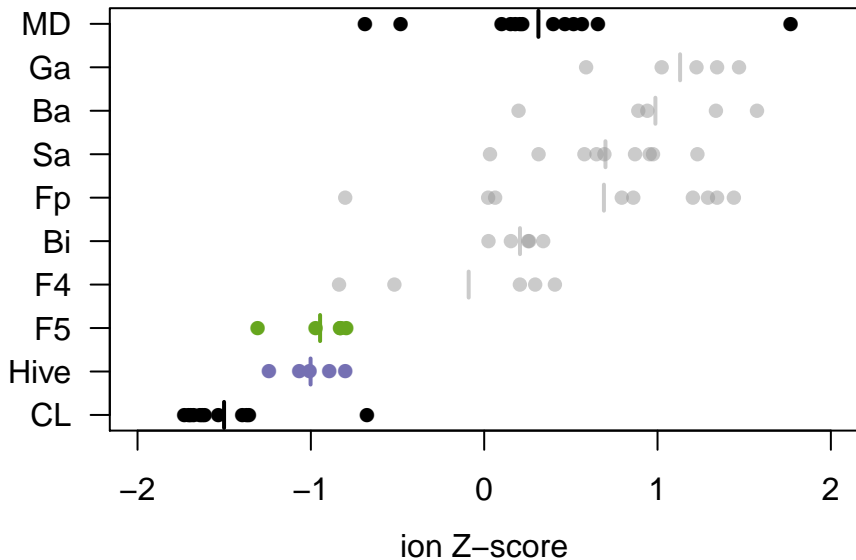

Supplement: S8 Data — (ZIP) [file pbio.2003467.s008.zip › Z-score_plots/469 microbial substrate Anthragallol.pdf]

# Buthidazole

# 472 255.091 microbial substrate

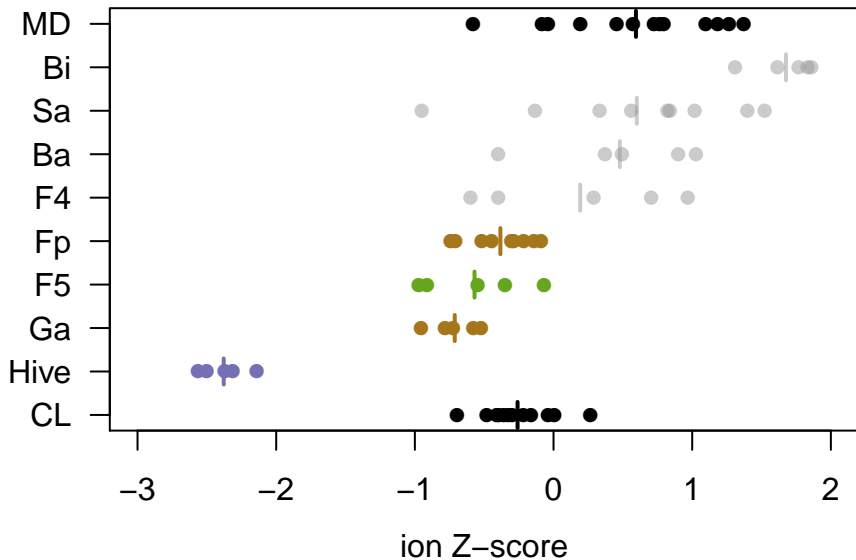

Supplement: S8 Data — (ZIP) [file pbio.2003467.s008.zip › Z-score_plots/472 microbial substrate Buthidazole.pdf]

**(1-Ribosylimidazole)-4-acetate\***  
**# 473 257.077 microbial substrate**

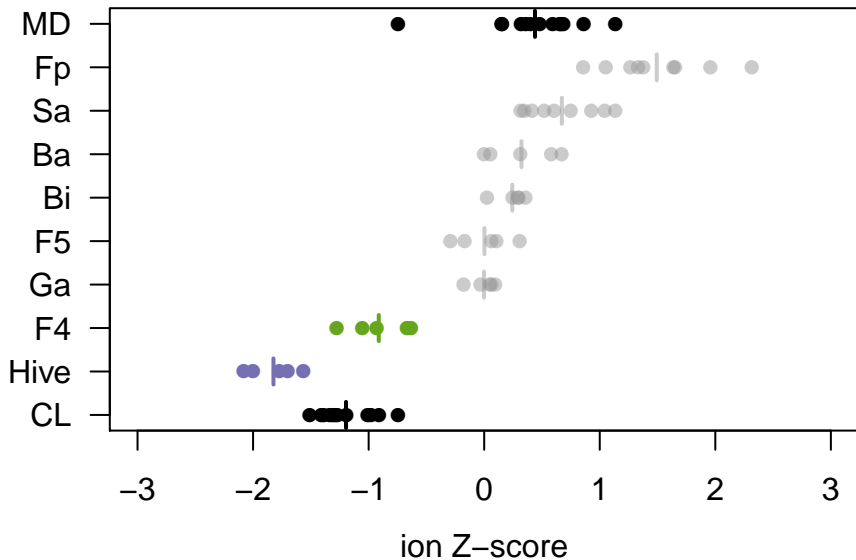

Supplement: S8 Data — (ZIP) [file pbio.2003467.s008.zip › Z-score_plots/473 microbial substrate (1-Ribosylimidazole)-4-acetate.pdf]

**D-Fructose 6-phosphate\***  
**# 476 259.022 microbial substrate**

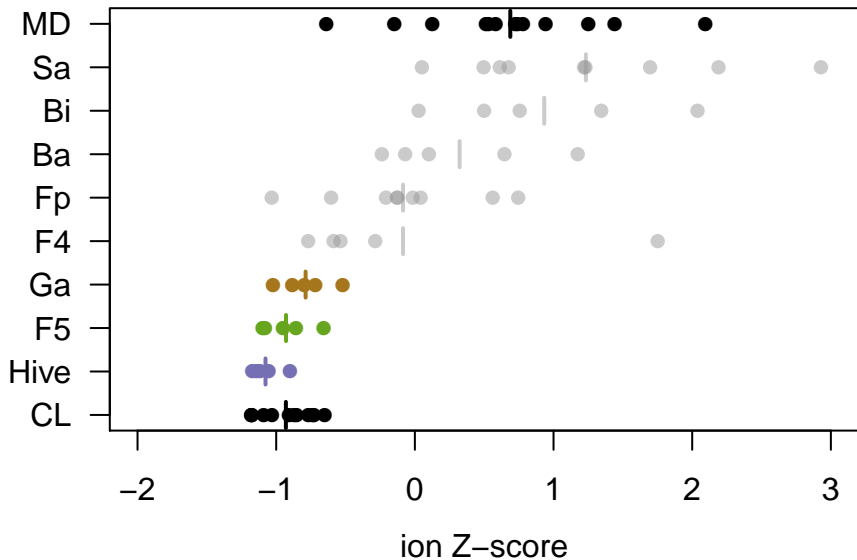

Supplement: S8 Data — (ZIP) [file pbio.2003467.s008.zip › Z-score_plots/476 microbial substrate D-Fructose 6-phosphate.pdf]

# Heptadecatrienoic acid

# 486 263.201 microbial product

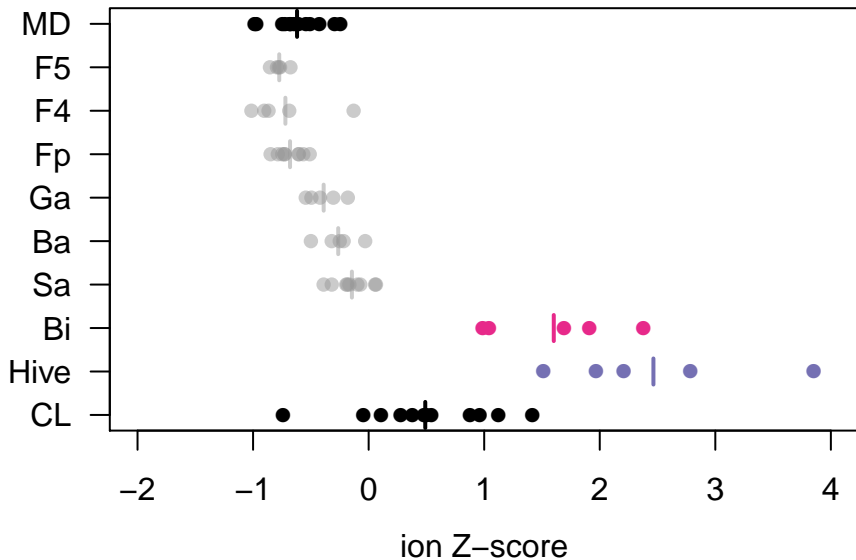

Supplement: S8 Data — (ZIP) [file pbio.2003467.s008.zip › Z-score_plots/486 microbial product Heptadecatrienoic acid.pdf]

**5'-Oxoinosine**  
**# 491 265.057 microbial product**

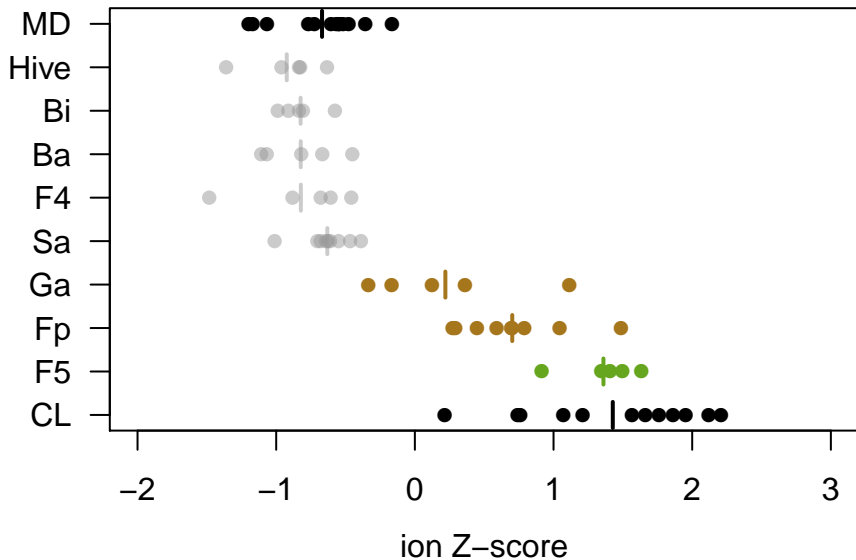

Supplement: S8 Data — (ZIP) [file pbio.2003467.s008.zip › Z-score_plots/491 microbial product 5'-Oxoinosine.pdf]

**Deoxyguanosine\***  
**# 495 266.089 microbial substrate**

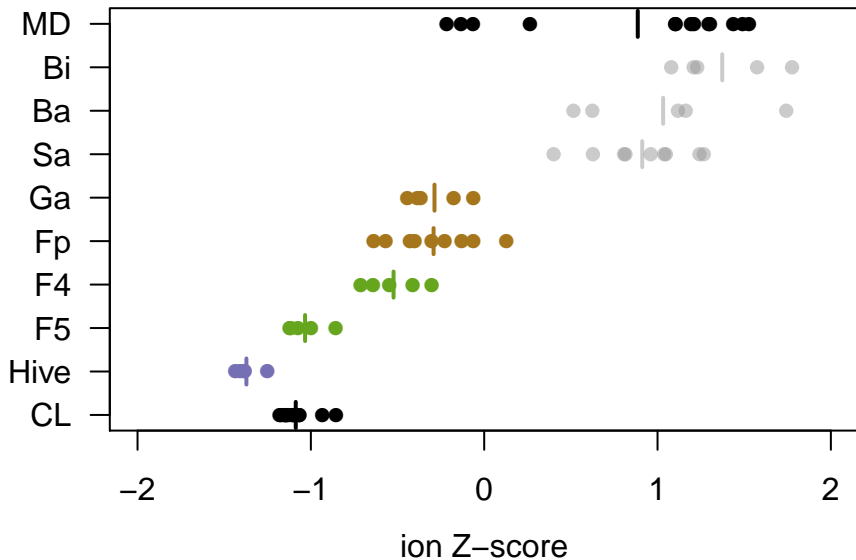

Supplement: S8 Data — (ZIP) [file pbio.2003467.s008.zip › Z-score_plots/495 microbial substrate Deoxyguanosine.pdf]

# Inosine

# 496 267.073 microbial substrate

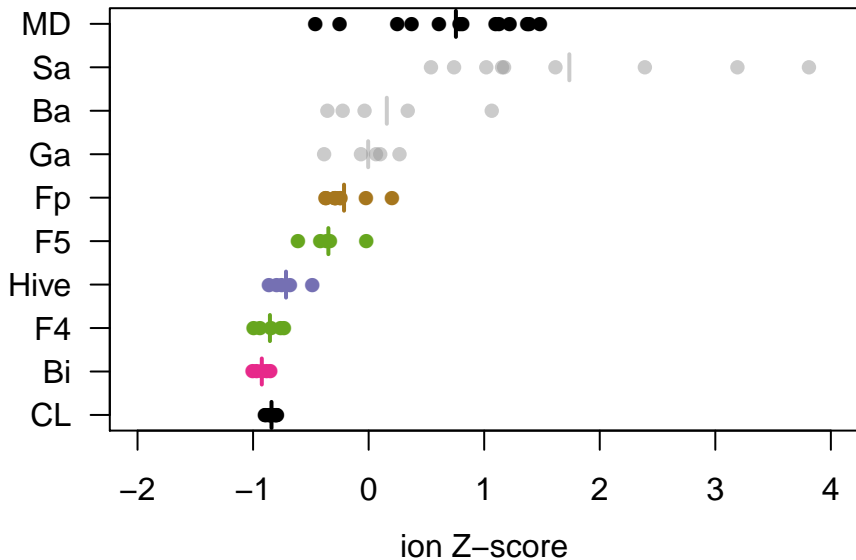

Supplement: S8 Data — (ZIP) [file pbio.2003467.s008.zip › Z-score_plots/496 microbial substrate Inosine.pdf]

**omega-Cyclohexylundecanoic acid\***  
**# 499 267.233 microbial substrate**

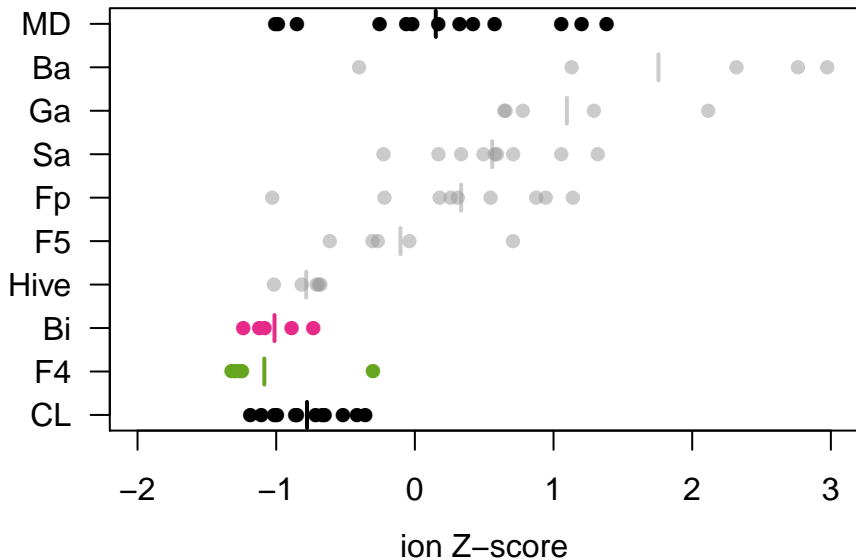

Supplement: S8 Data — (ZIP) [file pbio.2003467.s008.zip › Z-score_plots/499 microbial substrate omega-Cyclohexylundecanoic acid.pdf]

# 2-Furoate

# 50 111.008 microbial substrate

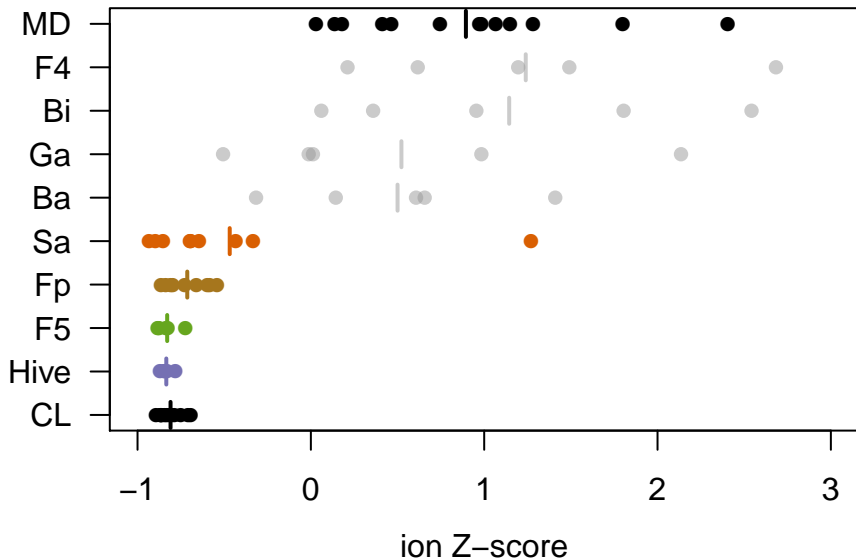

Supplement: S8 Data — (ZIP) [file pbio.2003467.s008.zip › Z-score_plots/50 microbial substrate 2-Furoate.pdf]

# Octadecanal

# 500 267.269 microbial substrate

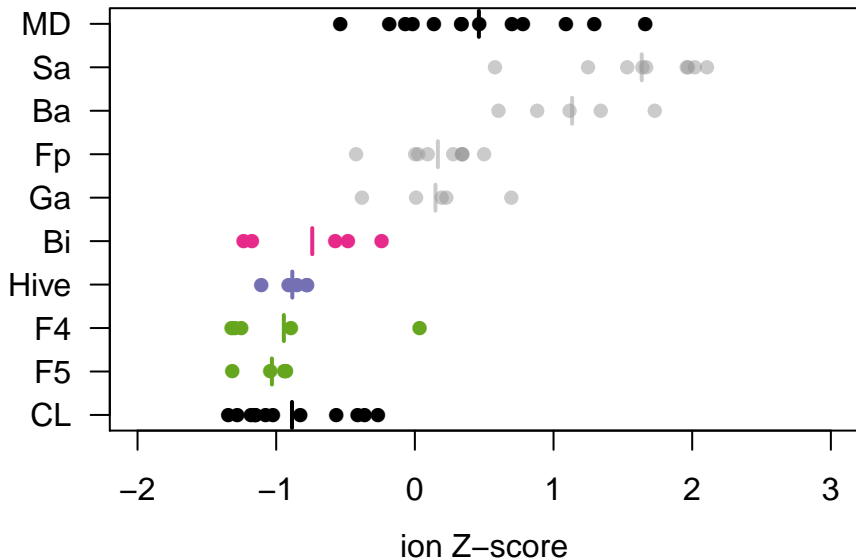

Supplement: S8 Data — (ZIP) [file pbio.2003467.s008.zip › Z-score_plots/500 microbial substrate Octadecanal.pdf]

# Sulfamethizole

# 502 269.018 microbial product

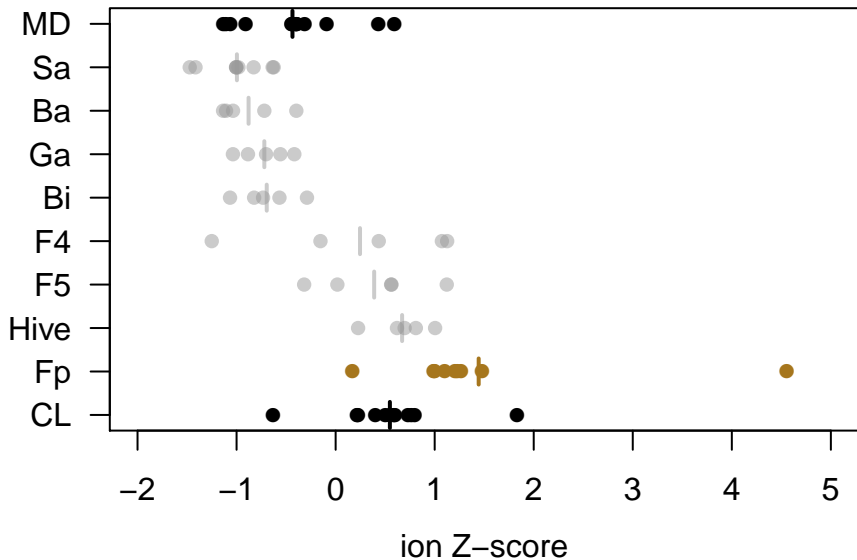

Supplement: S8 Data — (ZIP) [file pbio.2003467.s008.zip › Z-score_plots/502 microbial product Sulfamethizole.pdf]

# Apigenin\*

# 503 269.045 microbial substrate

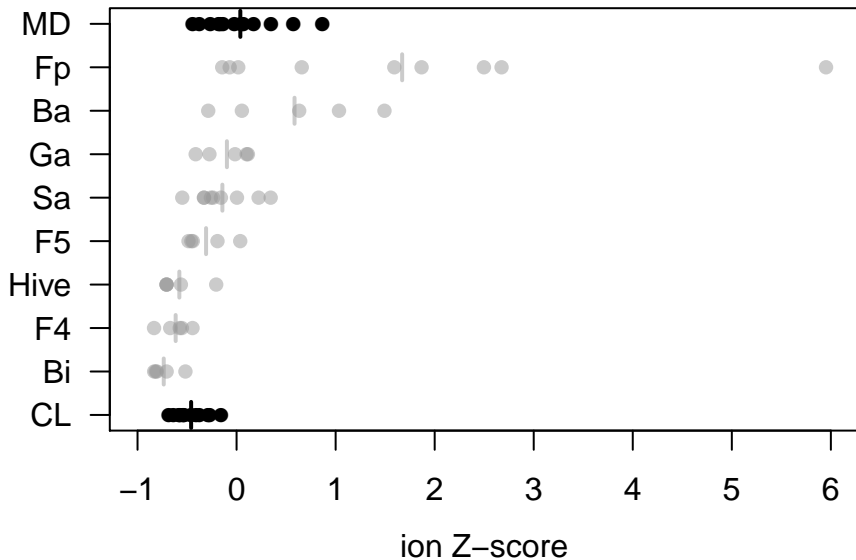

Supplement: S8 Data — (ZIP) [file pbio.2003467.s008.zip › Z-score_plots/503 microbial substrate Apigenin.pdf]

**Estrone\***

**# 506 269.155 microbial product**

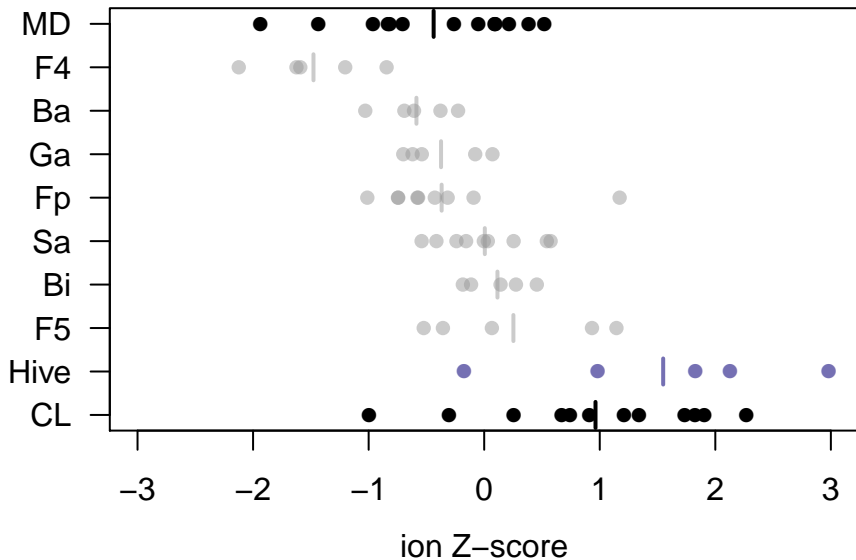

Supplement: S8 Data — (ZIP) [file pbio.2003467.s008.zip › Z-score_plots/506 microbial product Estrone.pdf]

**(10S)–Juvenile hormone III acid diol**  
**# 507 269.176 microbial product**

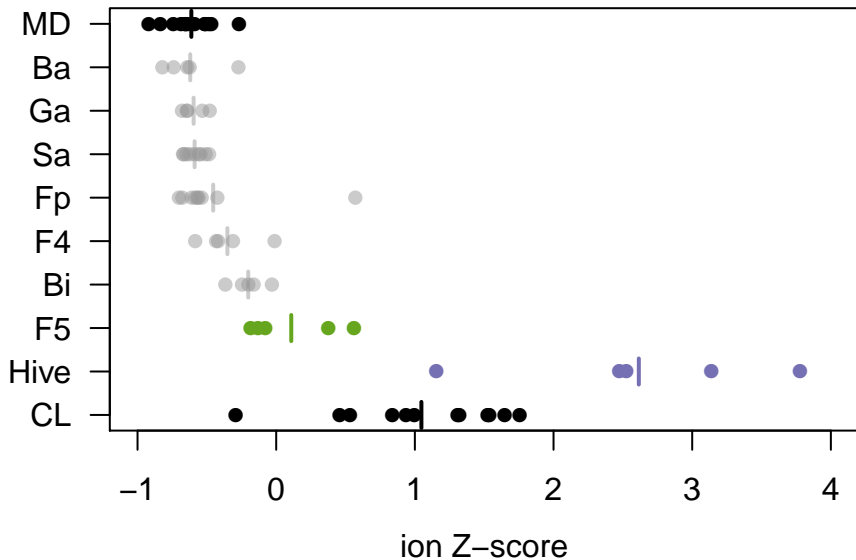

Supplement: S8 Data — (ZIP) [file pbio.2003467.s008.zip › Z-score_plots/507 microbial product (10S)-Juvenile hormone III acid diol.pdf]

# 16-Oxo-palmitate

# 508 269.212 microbial substrate

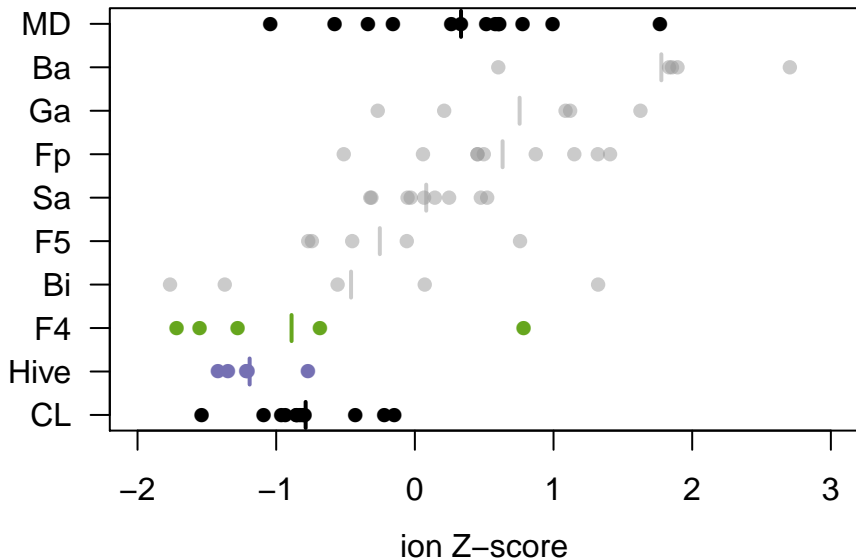

Supplement: S8 Data — (ZIP) [file pbio.2003467.s008.zip › Z-score_plots/508 microbial substrate 16-Oxo-palmitate.pdf]

Uracil\*

# 51 111.019 microbial substrate

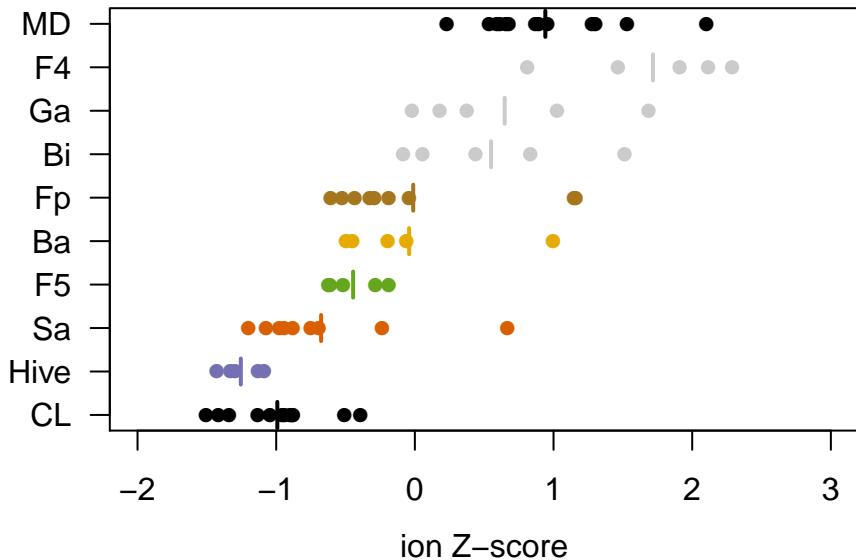

Supplement: S8 Data — (ZIP) [file pbio.2003467.s008.zip › Z-score_plots/51 microbial substrate Uracil.pdf]

**B PE**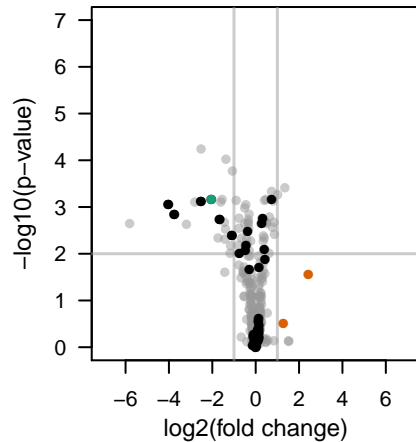**F5 PE**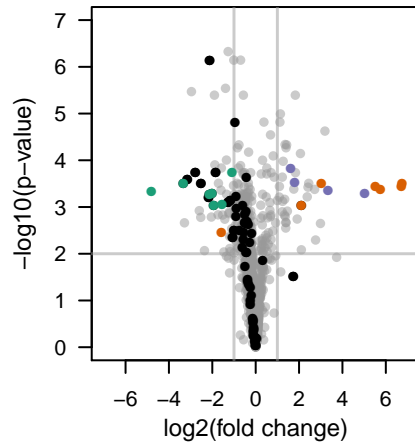**F4 PE**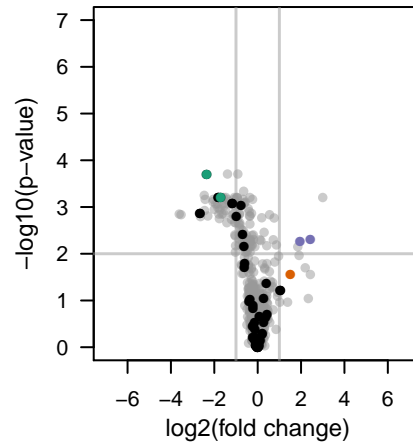**S PE**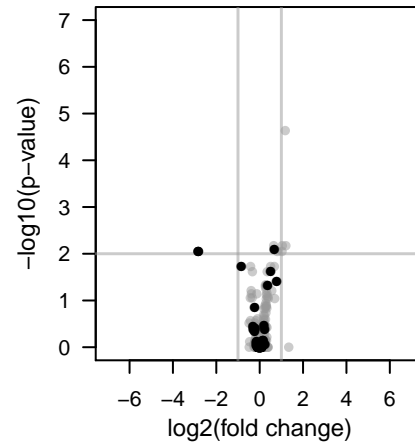**A1 PE**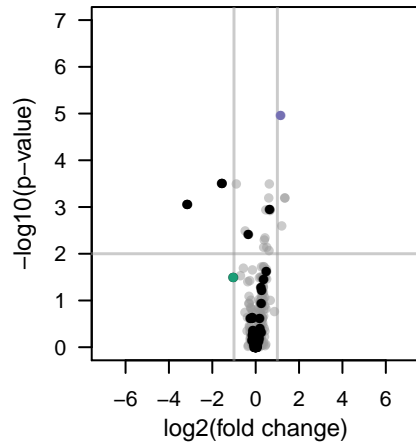**Fp PE**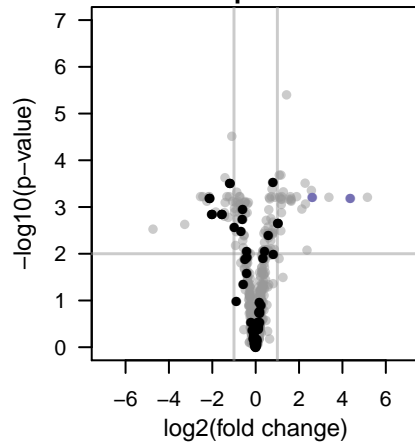**G PE**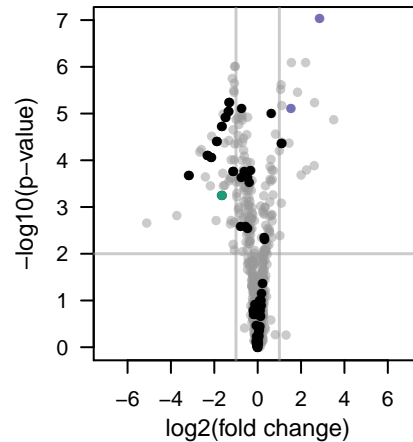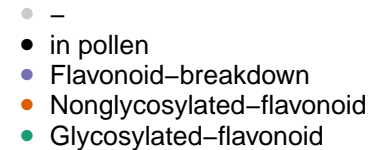

Supplement: S11 Data — (ZIP) [file pbio.2003467.s011.zip › expectedResults/bee microbiota in vitro patterns categories cutoff colored.pdf]

**B PE**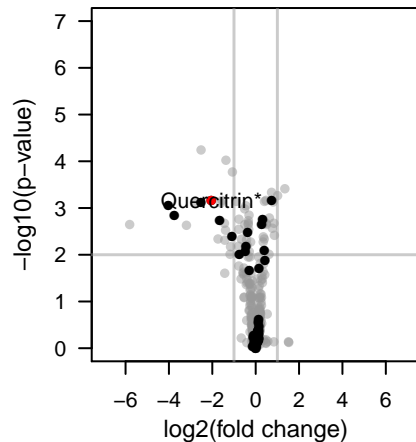**F5 PE**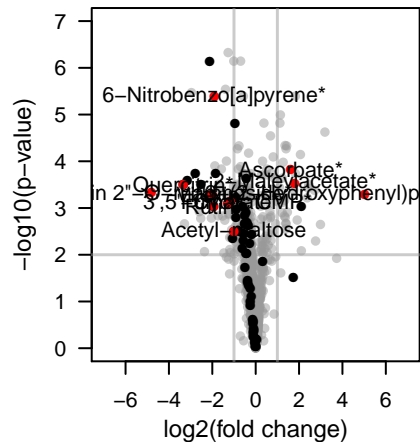**F4 PE**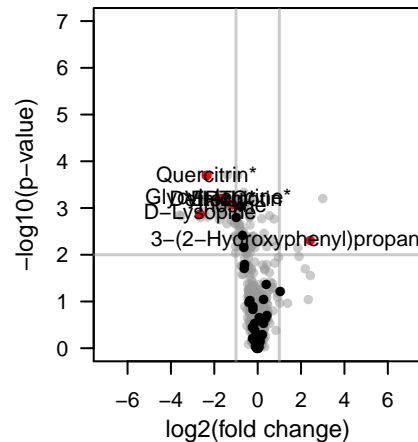**S PE**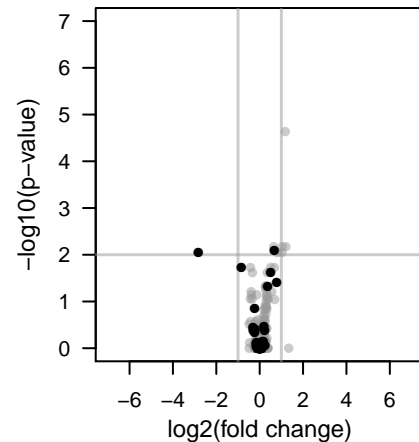**A1 PE**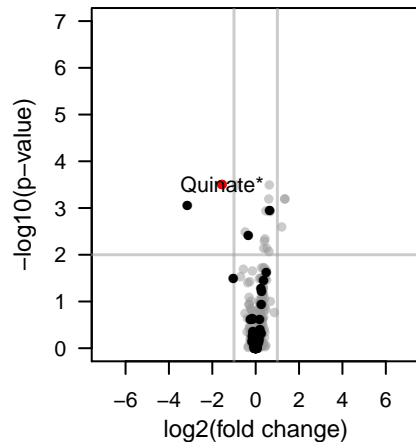**Fp PE**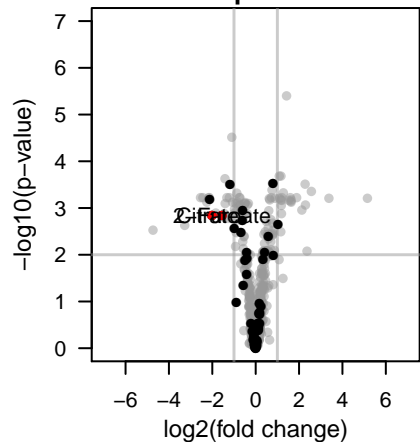**G PE**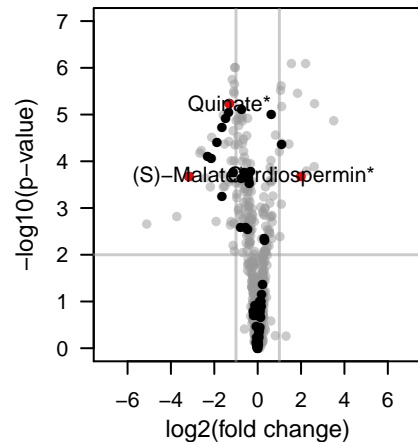

Supplement: S11 Data — (ZIP) [file pbio.2003467.s011.zip › expectedResults/bee microbiota in vivo in vitro explained volcano names.pdf]

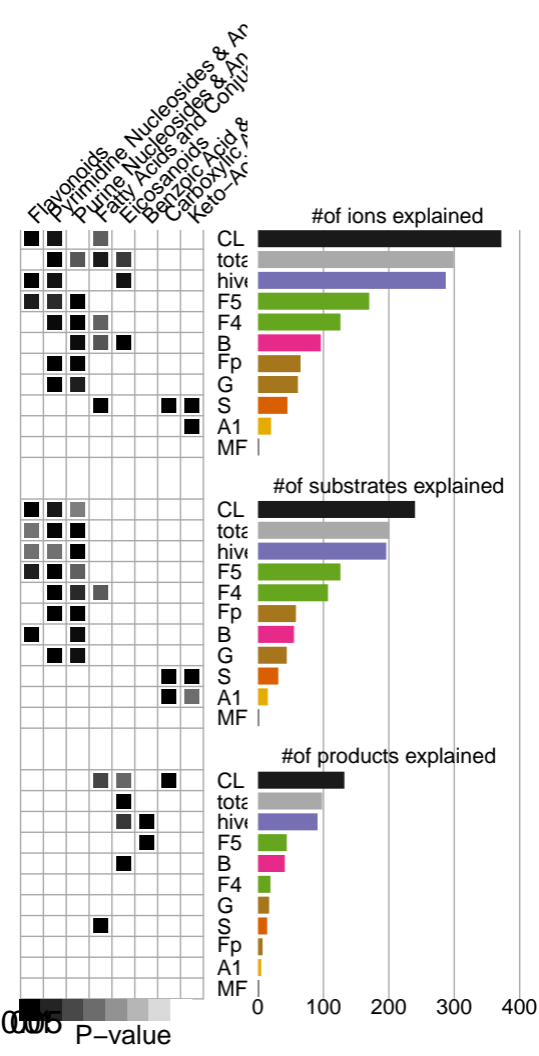

Supplement: S11 Data — (ZIP) [file pbio.2003467.s011.zip › expectedResults/heatmap and barplot selected cats v3.pdf]

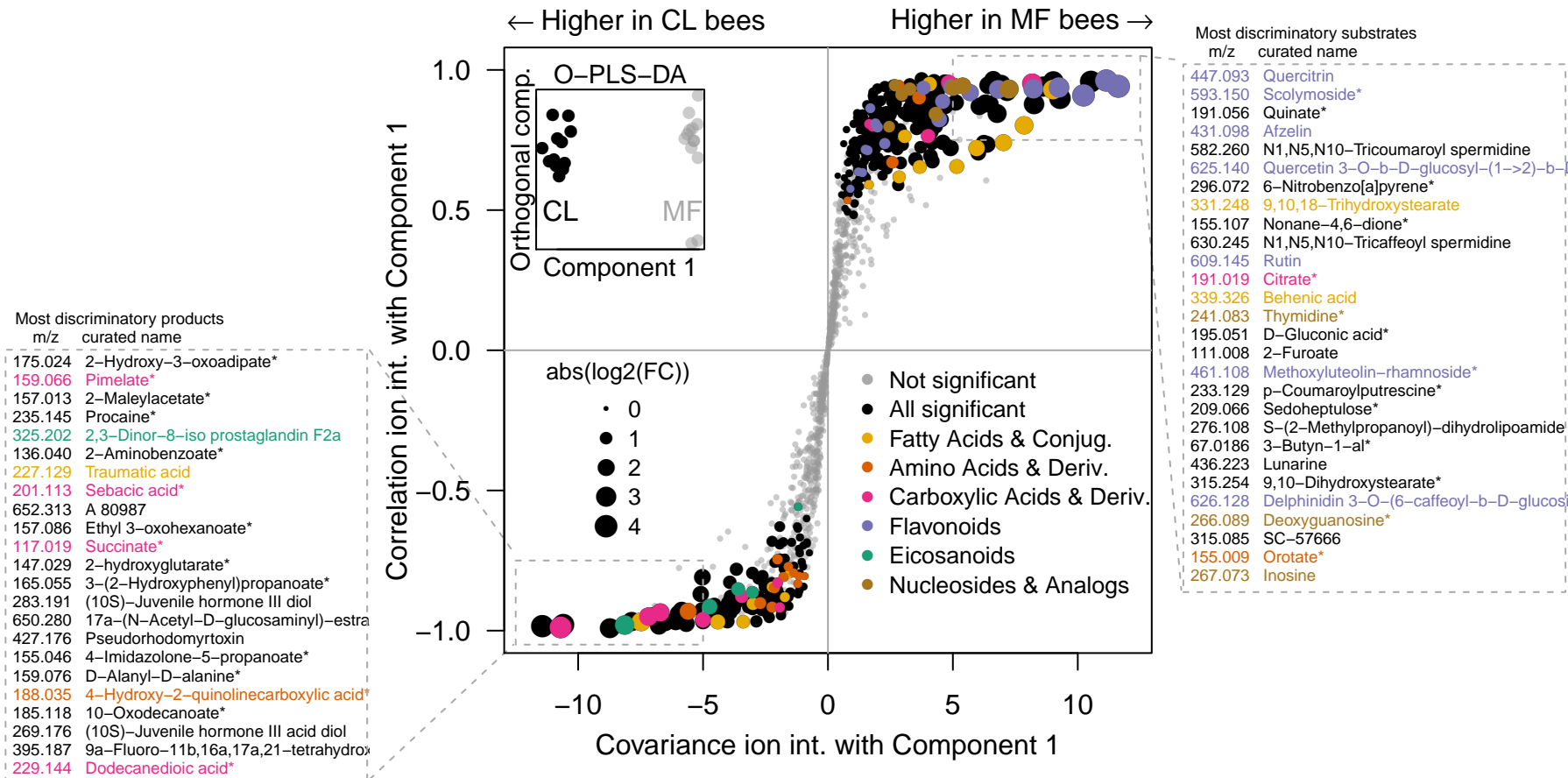

Supplement: S11 Data — (ZIP) [file pbio.2003467.s011.zip › expectedResults/oplsda fall only v5.pdf]
