# Supplementary figures and images for: Disentangling metabolic functions of bacteria in the honey bee gut (part 1 of 3)
Source: PLoS Biol. 2017 Dec 12;15(12):e2003467. doi: 10.1371/journal.pbio.2003467 (PMC5726620; doi:10.1371/journal.pbio.2003467)

**Delphinidin 3-O-(6-caffeoyl-beta-D-glucoside)**  
**# 1001 626.128 microbial substrate**

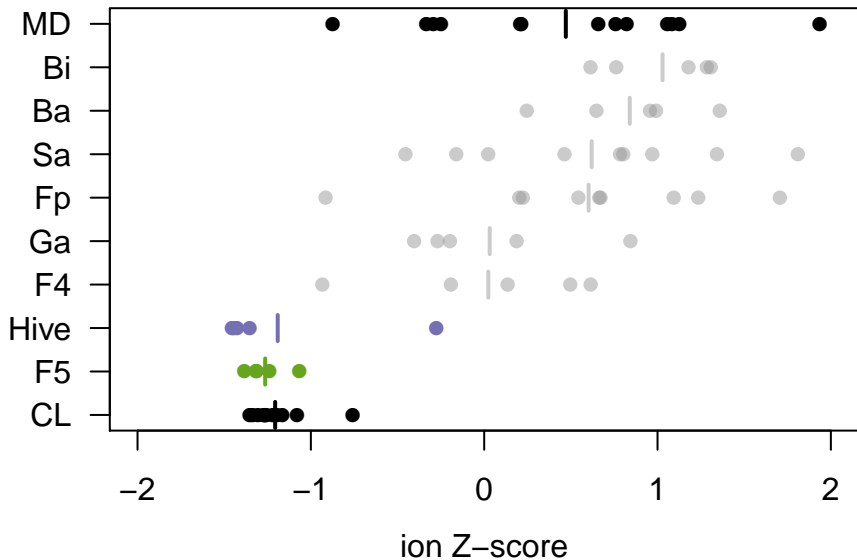

Supplement: S8 Data — (ZIP) [file pbio.2003467.s008.zip › Z-score_plots/1001 microbial substrate Delphinidin 3-O-(6-caffeoyl-beta-D-glucoside).pdf]

# Prebetanin

# 1002 629.095 microbial substrate

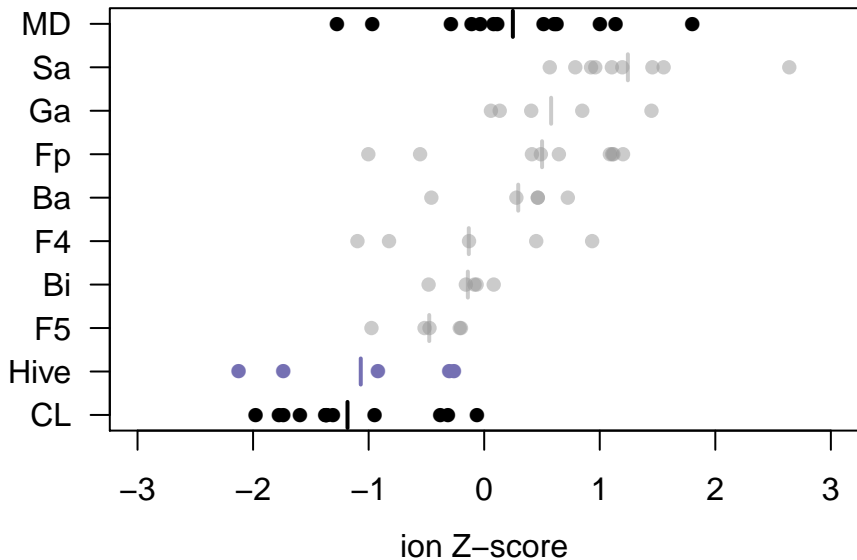

Supplement: S8 Data — (ZIP) [file pbio.2003467.s008.zip › Z-score_plots/1002 microbial substrate Prebetanin.pdf]

# Actinorhodin

# 1005 633.125 microbial substrate

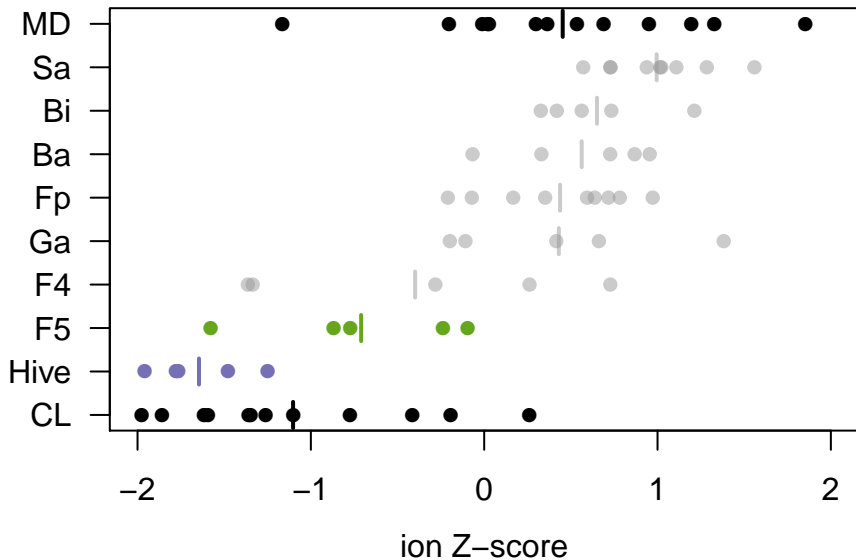

Supplement: S8 Data — (ZIP) [file pbio.2003467.s008.zip › Z-score_plots/1005 microbial substrate Actinorhodin.pdf]

# Kanokoside C

# 1006 637.235 microbial product

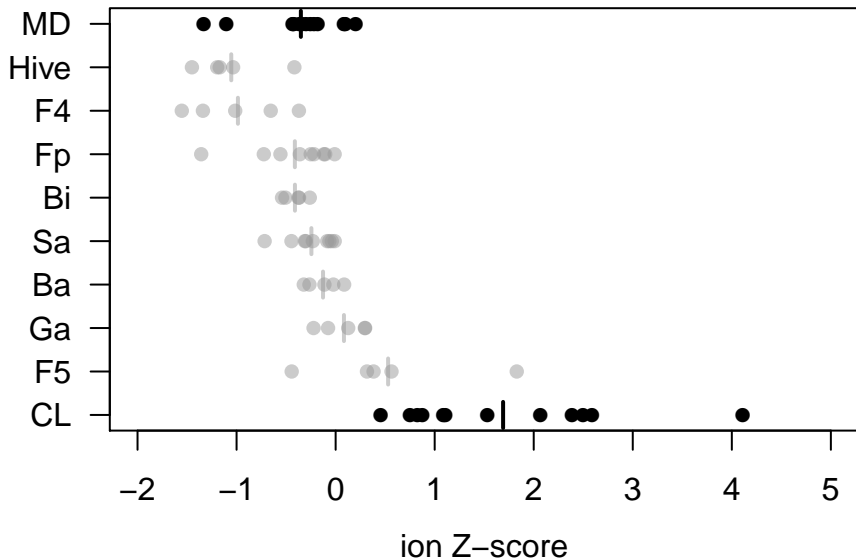

Supplement: S8 Data — (ZIP) [file pbio.2003467.s008.zip › Z-score_plots/1006 microbial product Kanokoside C.pdf]

**CMP-pseudaminic acid\***  
**# 1008 638.170 microbial substrate**

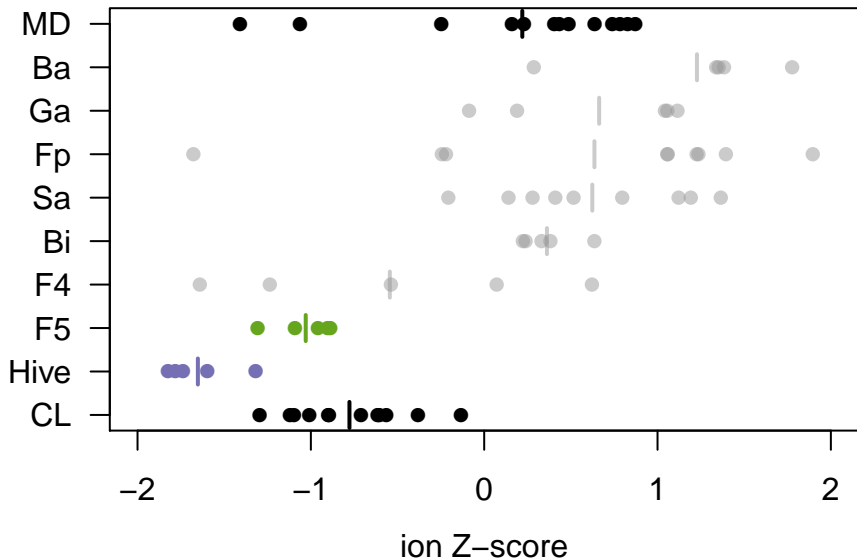

Supplement: S8 Data — (ZIP) [file pbio.2003467.s008.zip › Z-score_plots/1008 microbial substrate CMP-pseudaminic acid.pdf]

# Demethyl-desacetyl-rifamycin S

# 1009 638.259 microbial product

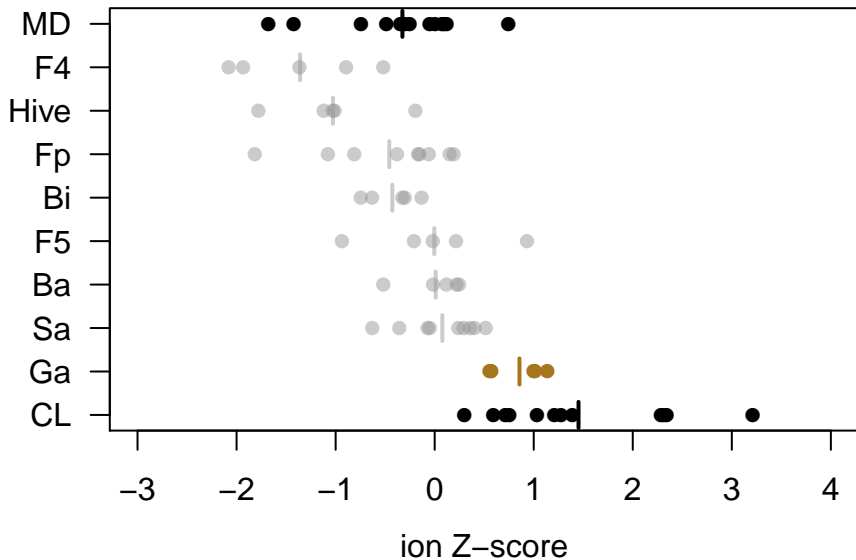

Supplement: S8 Data — (ZIP) [file pbio.2003467.s008.zip › Z-score_plots/1009 microbial product Demethyl-desacetyl-rifamycin S.pdf]

**Alpha-(N-Acetyl-D-glucosaminy)-estradiol 3-D-gluc**  
**# 1012 650.280 microbial product**

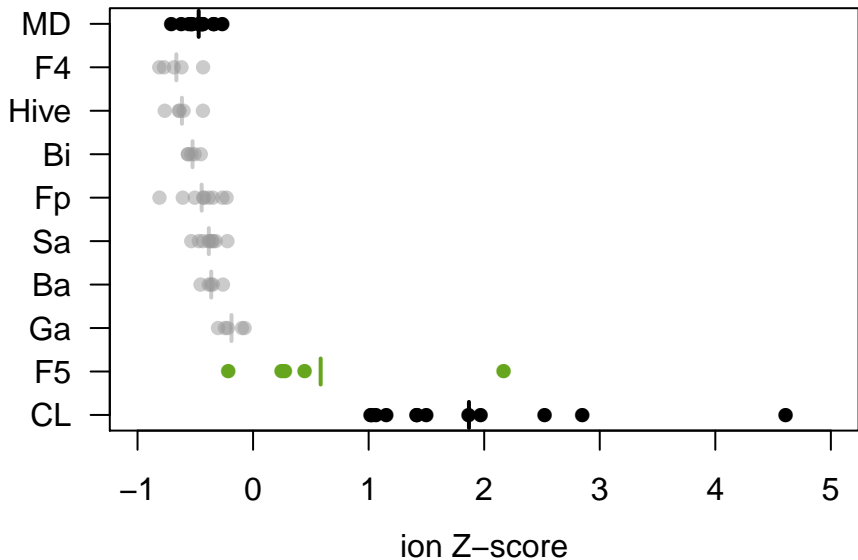

Supplement: S8 Data — (ZIP) [file pbio.2003467.s008.zip › Z-score_plots/1012 microbial product 17alpha-(N-Acetyl-D-glucosaminyl)-estradiol 3-D-glucuronoside.pdf]

**A 80987**

**# 1014 652.313 microbial product**

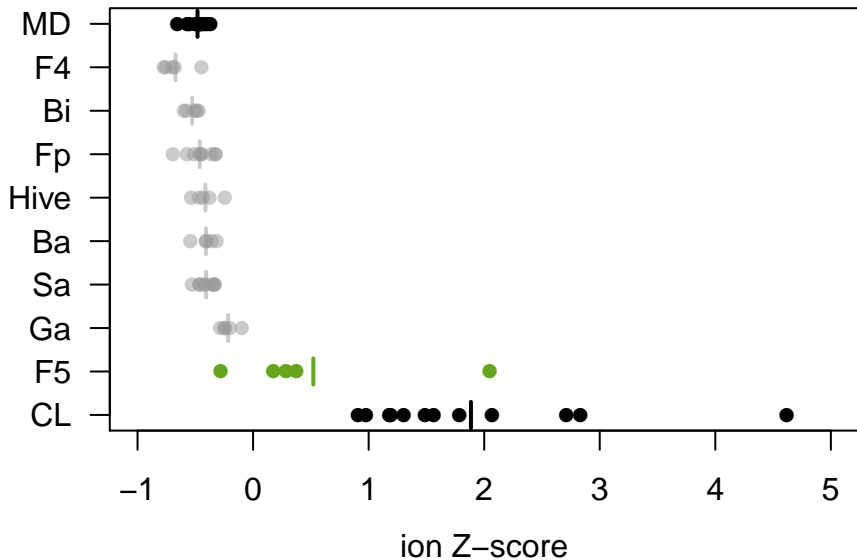

Supplement: S8 Data — (ZIP) [file pbio.2003467.s008.zip › Z-score_plots/1014 microbial product A 80987.pdf]

# Teniposide

# 1015 655.150 microbial substrate

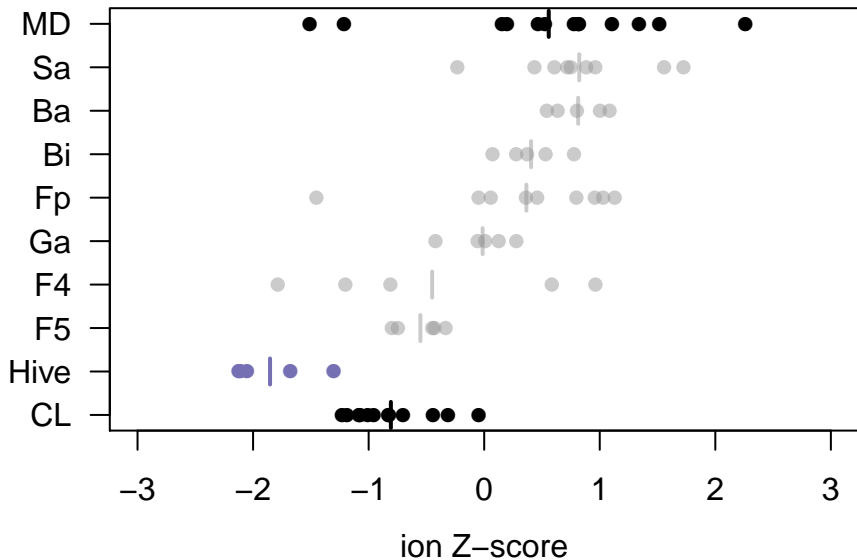

Supplement: S8 Data — (ZIP) [file pbio.2003467.s008.zip › Z-score_plots/1015 microbial substrate Teniposide.pdf]

**5-Aminolevulinate\***  
**# 102 130.050 microbial product**

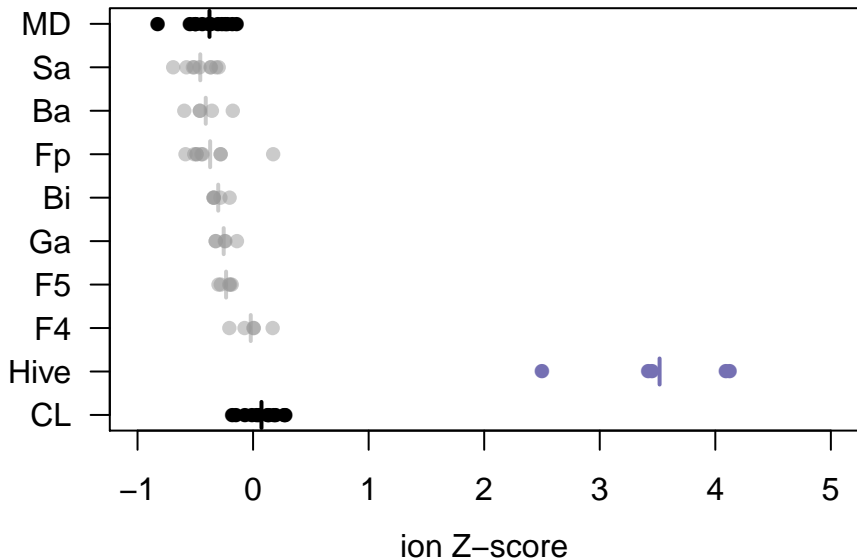

Supplement: S8 Data — (ZIP) [file pbio.2003467.s008.zip › Z-score_plots/102 microbial product 5-Aminolevulinate.pdf]

**Hexadecanoyl-2-(9Z-octadecenoyl)-sn-glycero-3-ph**  
**# 1023 673.480 microbial substrate**

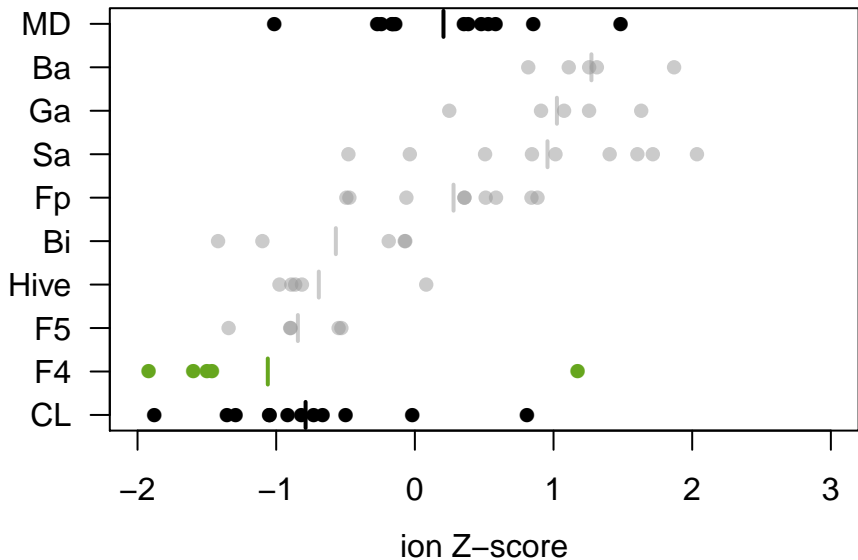

Supplement: S8 Data — (ZIP) [file pbio.2003467.s008.zip › Z-score_plots/1023 microbial substrate 1-Hexadecanoyl-2-(9Z-octadecenoyl)-sn-glycero-3-phosphate.pdf]

# Rifamycin S

# 1029 694.287 microbial product

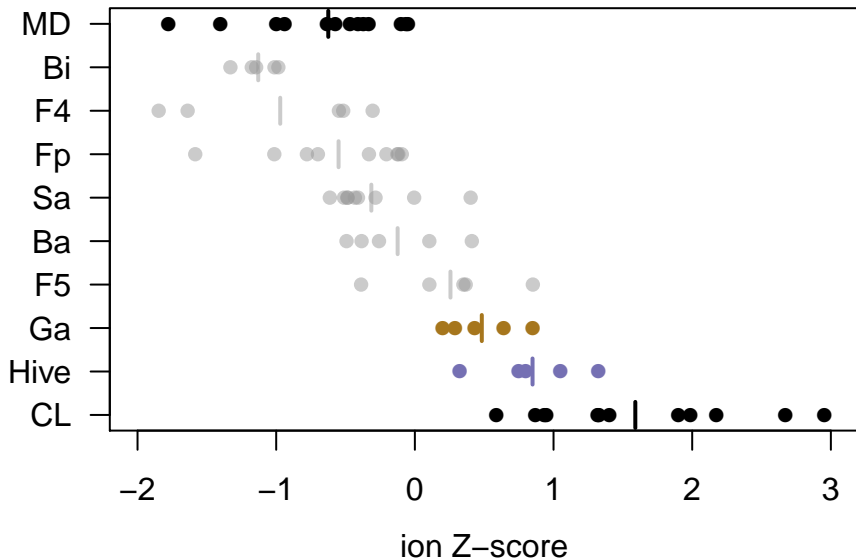

Supplement: S8 Data — (ZIP) [file pbio.2003467.s008.zip › Z-score_plots/1029 microbial product Rifamycin S.pdf]

ecanoyl-2-(9Z-octadecenoyl)-sn-glycero-3-phospho  
# 1032 700.526 microbial substrate

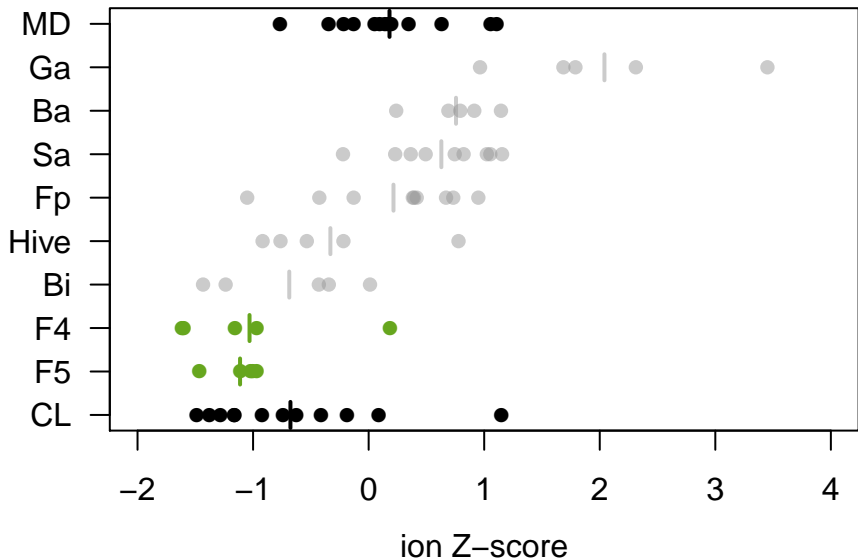

Supplement: S8 Data — (ZIP) [file pbio.2003467.s008.zip › Z-score_plots/1032 microbial substrate 1-Hexadecanoyl-2-(9Z-octadecenoyl)-sn-glycero-3-phosphonoethanolamine.pdf]

**lec canoyl-2-(9Z-octadecenoyl)-sn-glycero-3-phospho**  
**# 1035 716.522 microbial substrate**

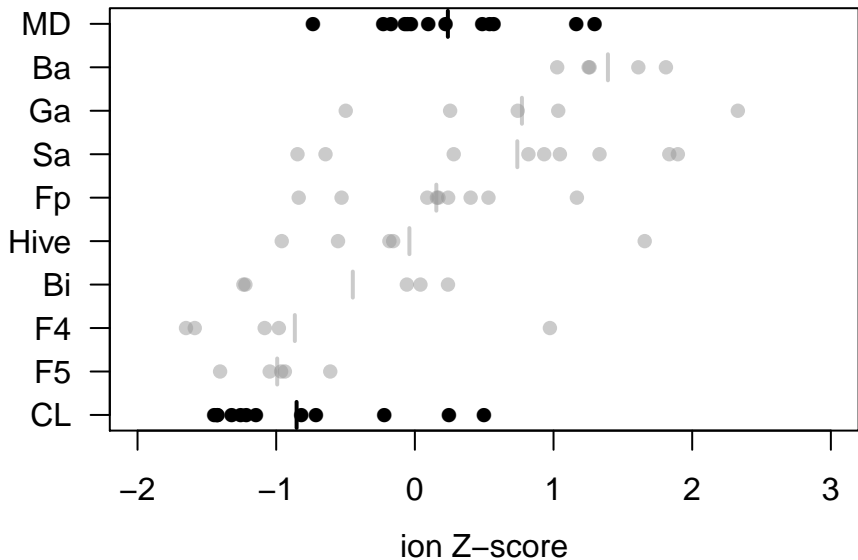

Supplement: S8 Data — (ZIP) [file pbio.2003467.s008.zip › Z-score_plots/1035 microbial substrate 1-Hexadecanoyl-2-(9Z-octadecenoyl)-sn-glycero-3-phosphoethanolamine.pdf]

# Robinin

# 1038 739.208 microbial substrate

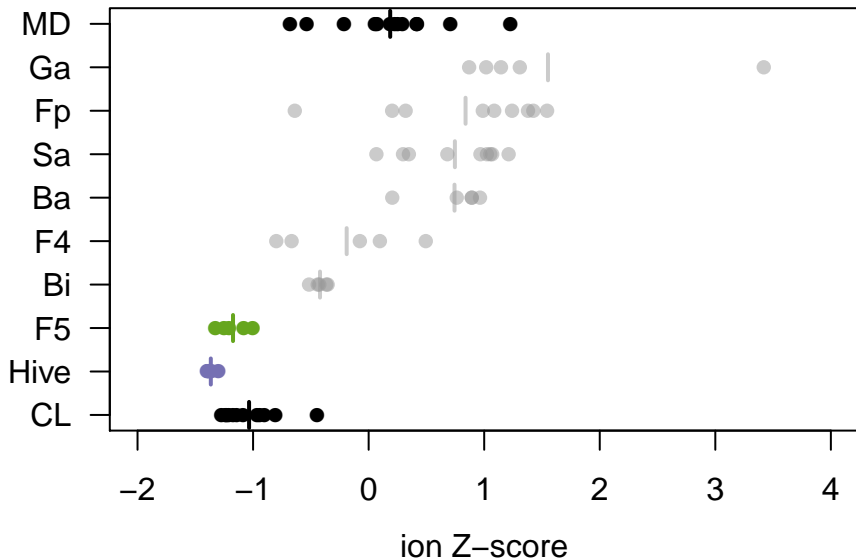

Supplement: S8 Data — (ZIP) [file pbio.2003467.s008.zip › Z-score_plots/1038 microbial substrate Robinin.pdf]

**hexadecanoyl-2-(9Z-octadecenoyl)-sn-glycerol-3-phosphate**  
**# 1044 759.577 microbial substrate**

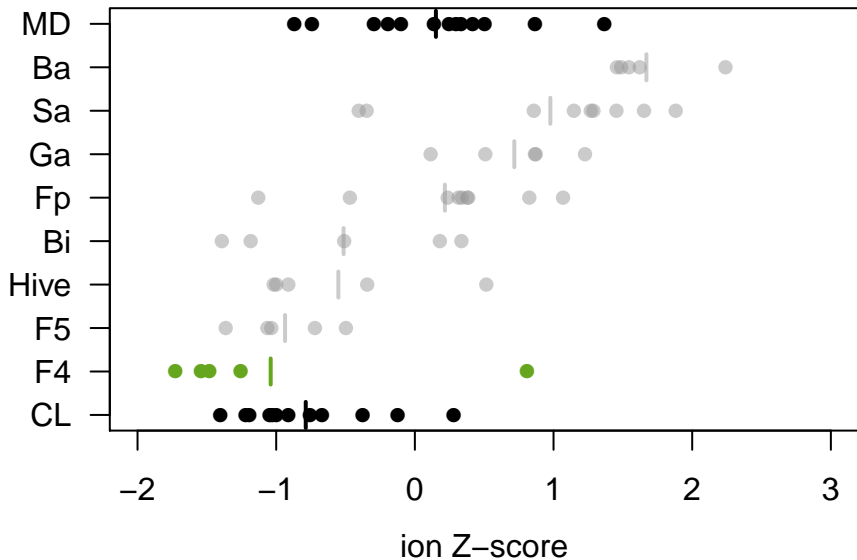

Supplement: S8 Data — (ZIP) [file pbio.2003467.s008.zip › Z-score_plots/1044 microbial substrate 1-Hexadecanoyl-2-(9Z-octadecenoyl)-sn-glycero-3-phosphocholine.pdf]

hexadecanoyl-2-(9Z-octadecenoyl)-sn-glycero-3-phosphatidylcholine  
# 1045 760.512 microbial substrate

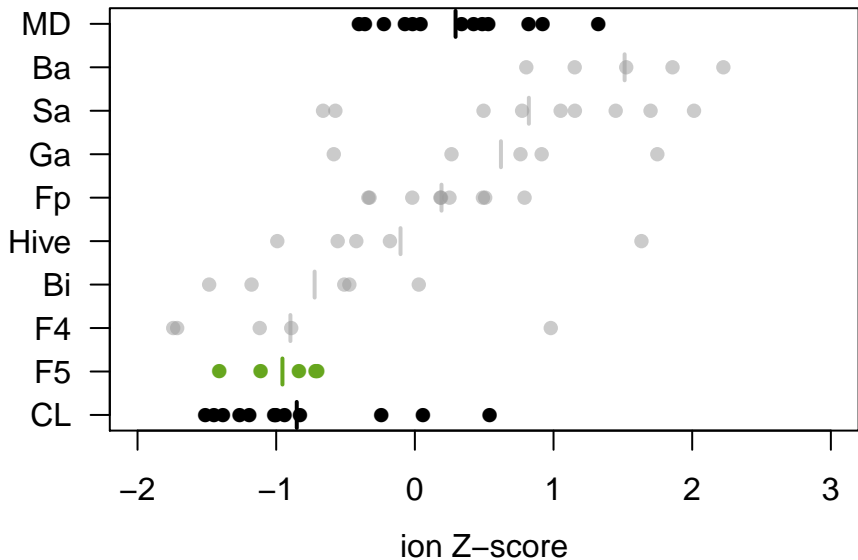

Supplement: S8 Data — (ZIP) [file pbio.2003467.s008.zip › Z-score_plots/1045 microbial substrate 1-Hexadecanoyl-2-(9Z-octadecenoyl)-sn-glycero-3-phosphoserine.pdf]

# Kaempferol 3-sophorotrioside

# 1048 771.198 microbial substrate

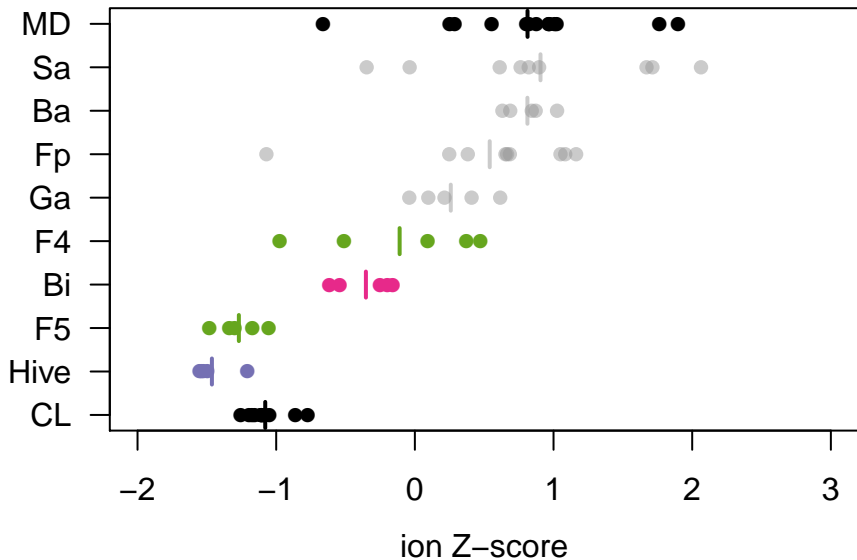

Supplement: S8 Data — (ZIP) [file pbio.2003467.s008.zip › Z-score_plots/1048 microbial substrate Kaempferol 3-sophorotrioside.pdf]

# Glutarate\*

# 105 131.035 microbial product

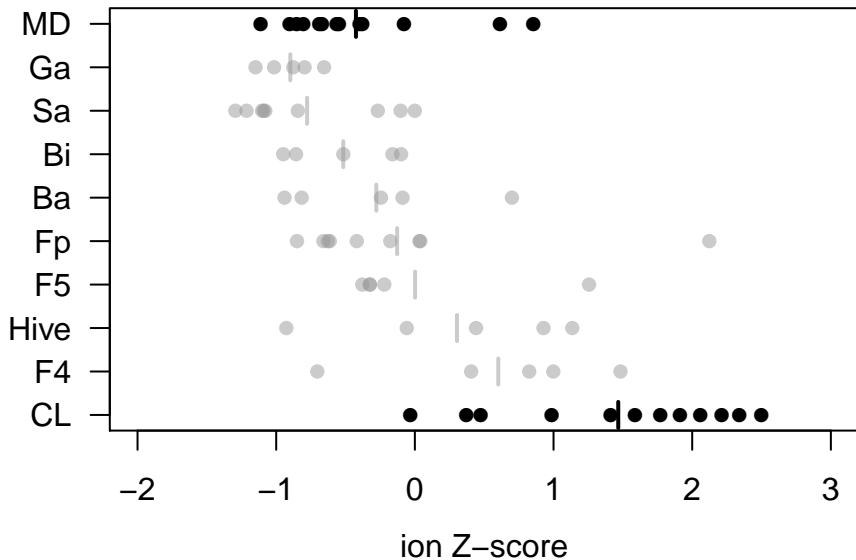

Supplement: S8 Data — (ZIP) [file pbio.2003467.s008.zip › Z-score_plots/105 microbial product Glutarate.pdf]

# 3-Demethylubiquinone-9

# 1051 779.599 microbial substrate

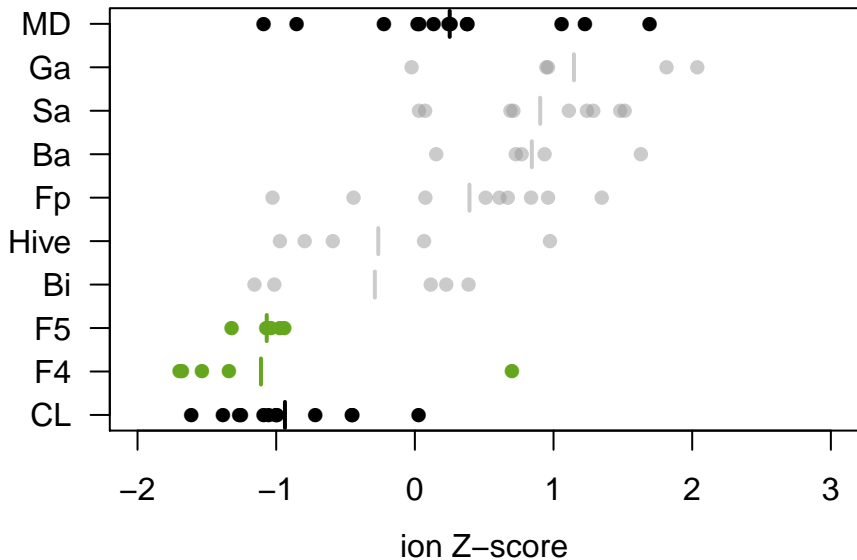

Supplement: S8 Data — (ZIP) [file pbio.2003467.s008.zip › Z-score_plots/1051 microbial substrate 3-Demethylubiquinone-9.pdf]

# 6"-Feruloylspinosin

# 1053 783.213 microbial substrate

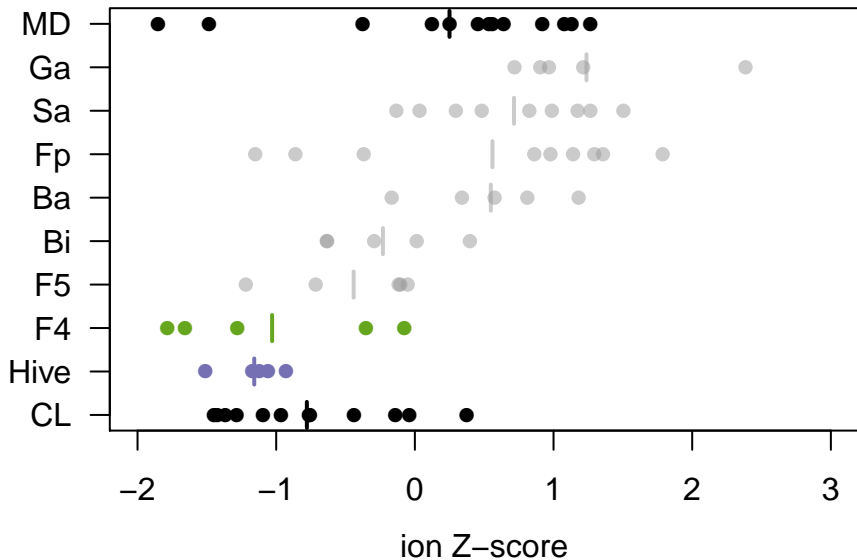

Supplement: S8 Data — (ZIP) [file pbio.2003467.s008.zip › Z-score_plots/1053 microbial substrate 6''-Feruloylspinosin.pdf]

# Asparagine\*

# 106 131.046 microbial substrate

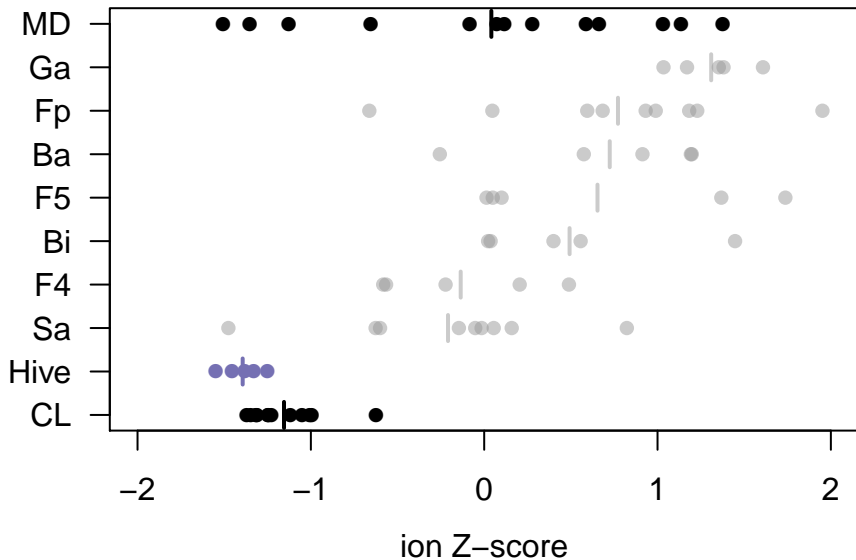

Supplement: S8 Data — (ZIP) [file pbio.2003467.s008.zip › Z-score_plots/106 microbial substrate Asparagine.pdf]

Malate\*

# 111 133.013 microbial substrate

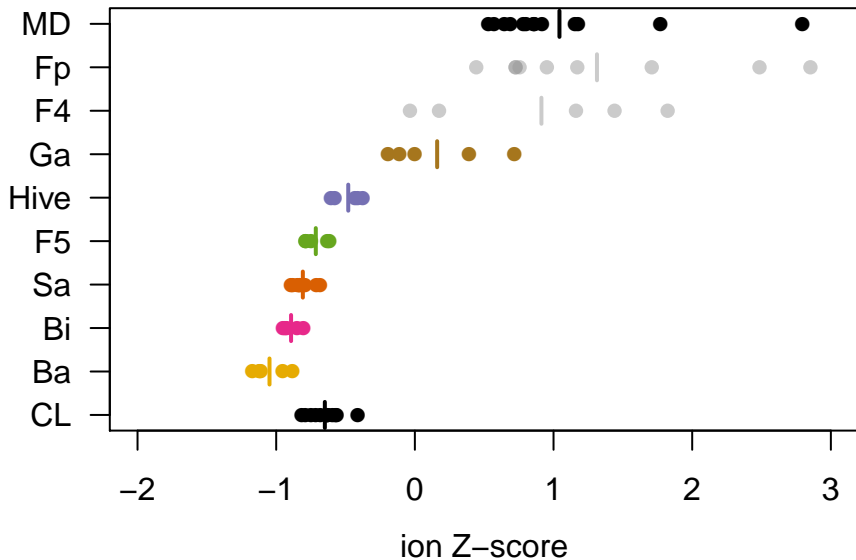

Supplement: S8 Data — (ZIP) [file pbio.2003467.s008.zip › Z-score_plots/111 microbial substrate Malate.pdf]

# Deoxyribose\*

# 112 133.050 microbial product

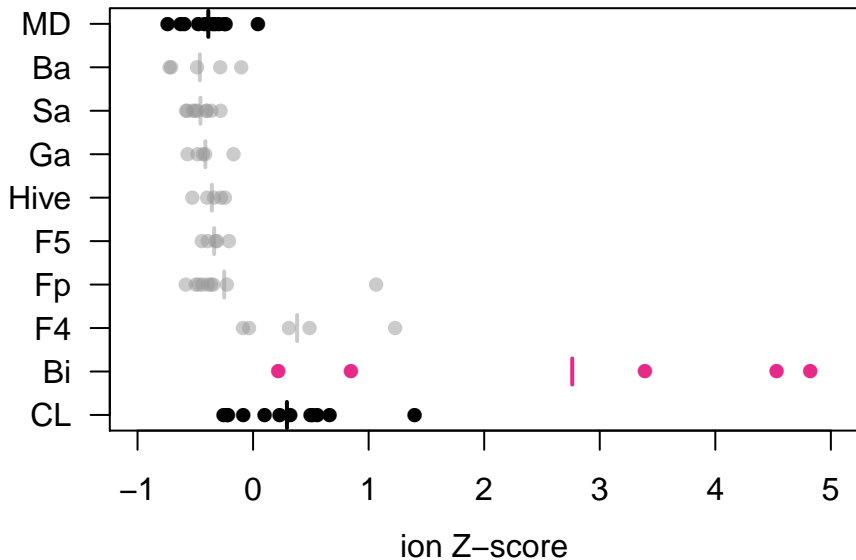

Supplement: S8 Data — (ZIP) [file pbio.2003467.s008.zip › Z-score_plots/112 microbial product Deoxyribose.pdf]

**Phenyl acetate\***  
**# 114 135.044 microbial substrate**

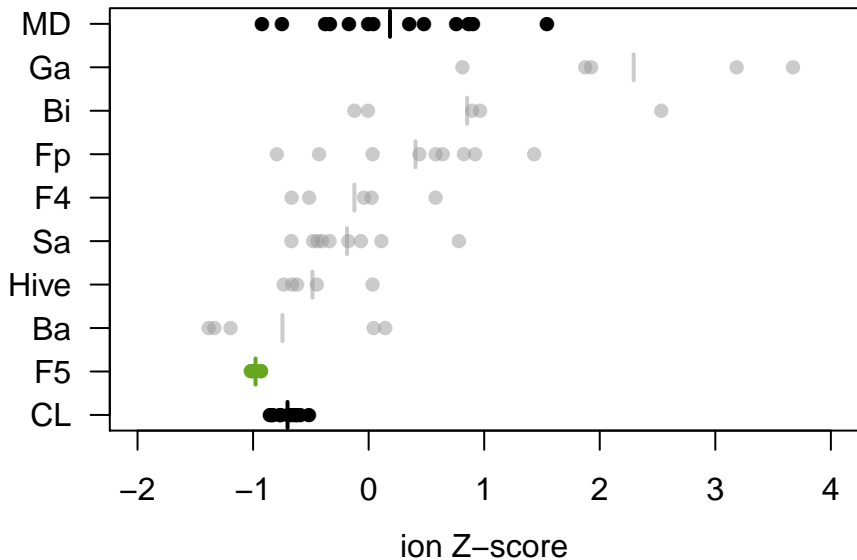

Supplement: S8 Data — (ZIP) [file pbio.2003467.s008.zip › Z-score_plots/114 microbial substrate Phenyl acetate.pdf]

# 2-Aminobenzoate\*

# 115 136.040 microbial product

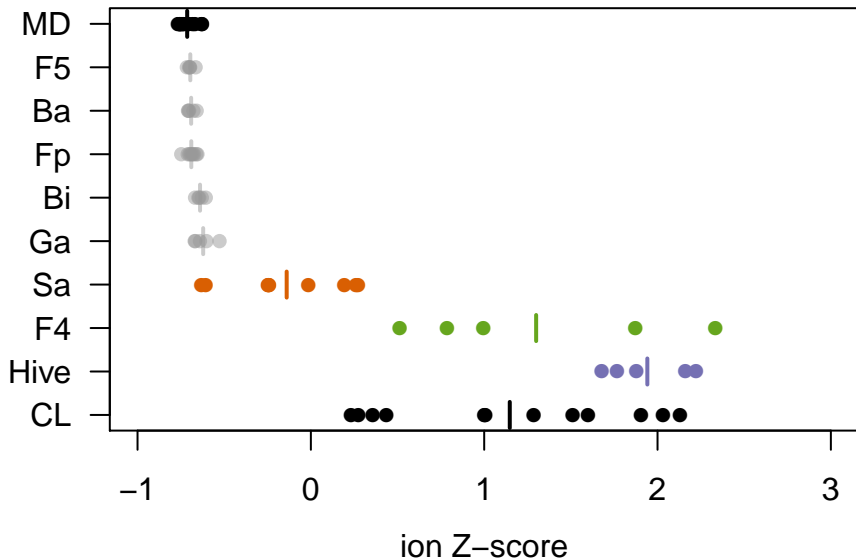

Supplement: S8 Data — (ZIP) [file pbio.2003467.s008.zip › Z-score_plots/115 microbial product 2-Aminobenzoate.pdf]

# Tyramine\*

# 116 136.076 microbial product

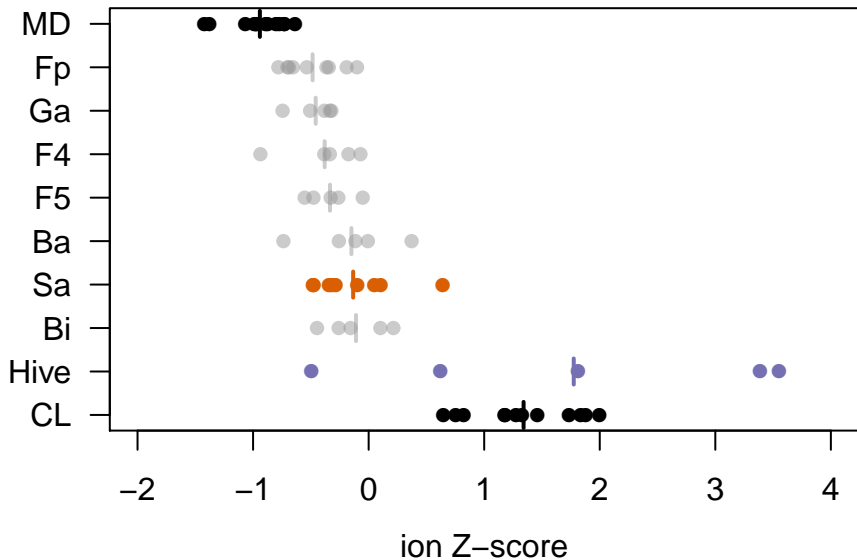

Supplement: S8 Data — (ZIP) [file pbio.2003467.s008.zip › Z-score_plots/116 microbial product Tyramine.pdf]

**Ethosuximide\***  
**# 119 140.071 microbial substrate**

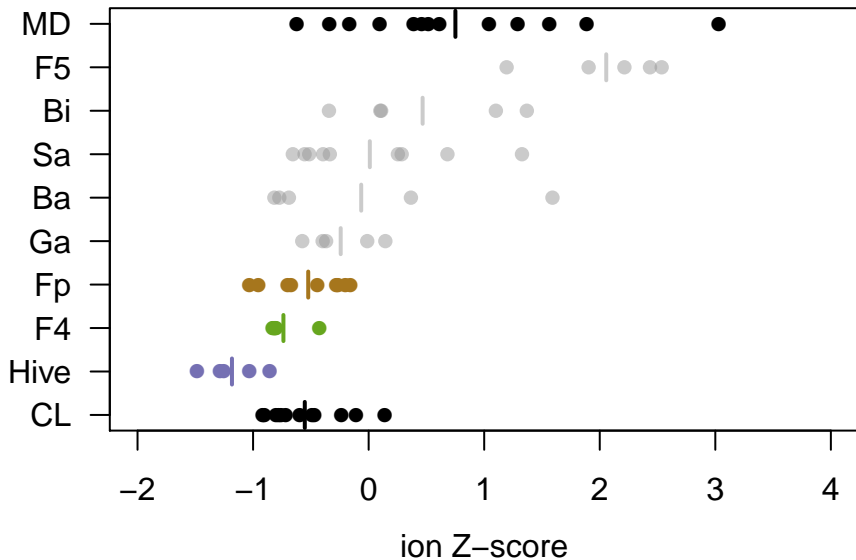

Supplement: S8 Data — (ZIP) [file pbio.2003467.s008.zip › Z-score_plots/119 microbial substrate Ethosuximide.pdf]

**Butanal\***

**# 12 71.0498 microbial product**

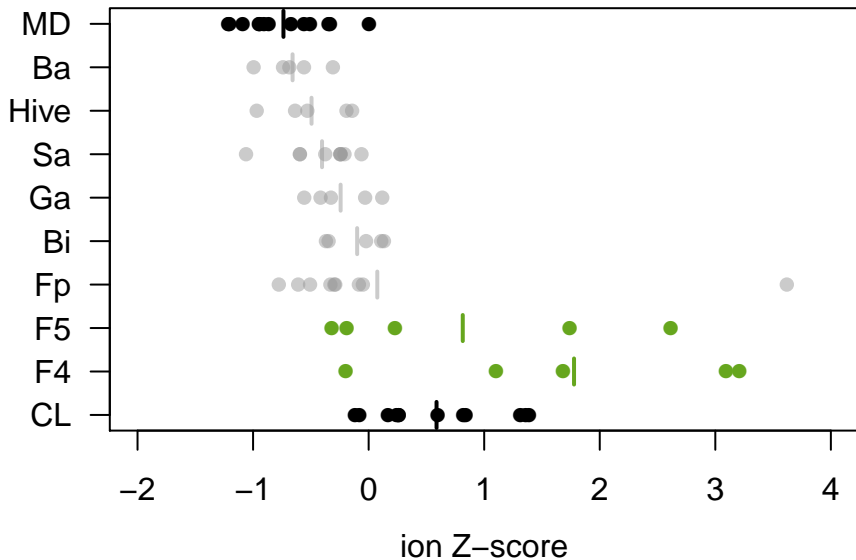

Supplement: S8 Data — (ZIP) [file pbio.2003467.s008.zip › Z-score_plots/12 microbial product Butanal.pdf]

# Tropine\*

# 120 140.108 microbial substrate

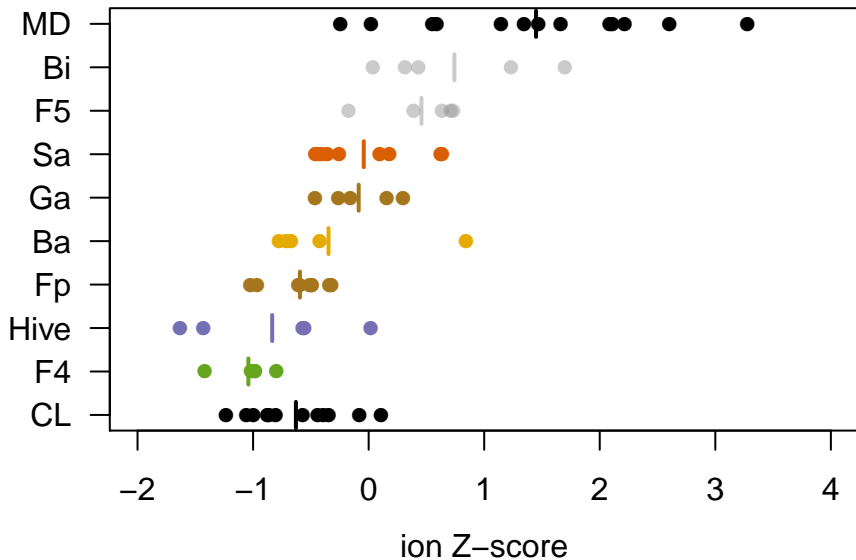

Supplement: S8 Data — (ZIP) [file pbio.2003467.s008.zip › Z-score_plots/120 microbial substrate Tropine.pdf]

**4-Oxocyclohexanecarboxylate**  
**# 121 141.055 microbial product**

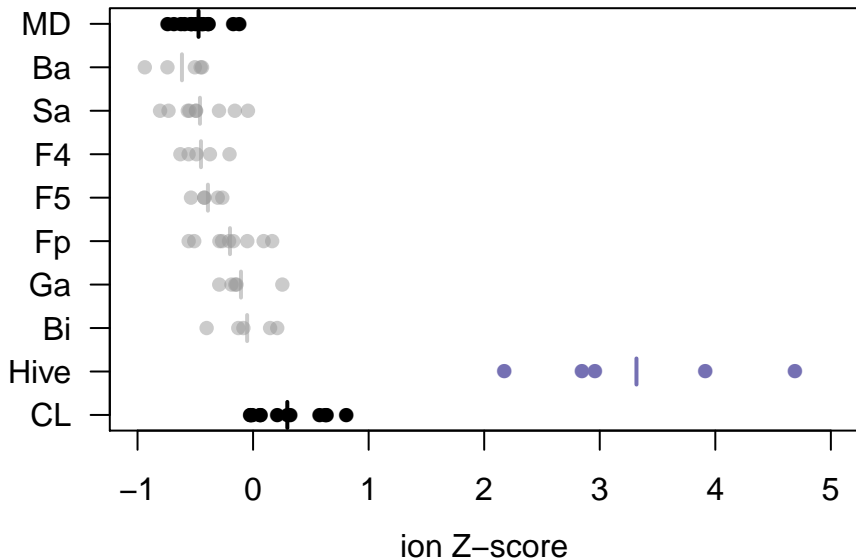

Supplement: S8 Data — (ZIP) [file pbio.2003467.s008.zip › Z-score_plots/121 microbial product 4-Oxocyclohexanecarboxylate.pdf]

**Cycloheptanecarboxylic acid\***  
**# 123 141.091 microbial product**

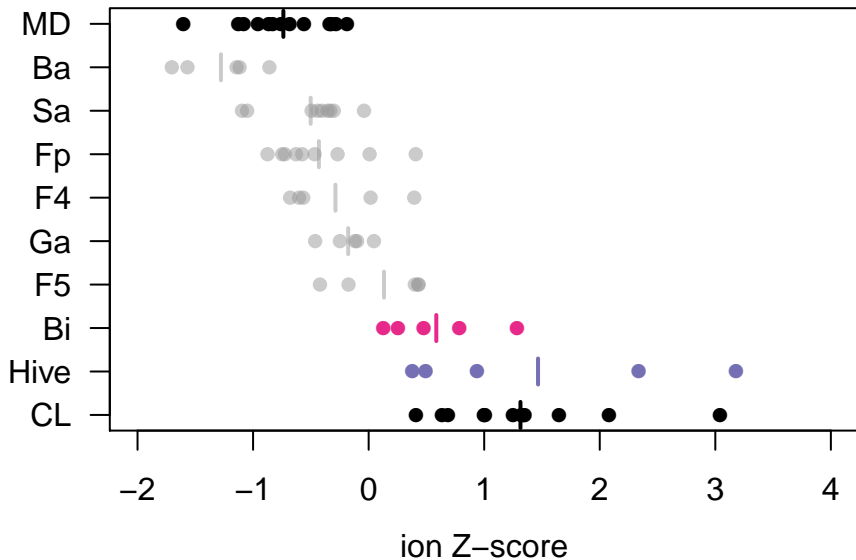

Supplement: S8 Data — (ZIP) [file pbio.2003467.s008.zip › Z-score_plots/123 microbial product Cycloheptanecarboxylic acid.pdf]

**trans-4-Hydroxycyclohexanecarboxylate\***  
**# 127 143.071 microbial product**

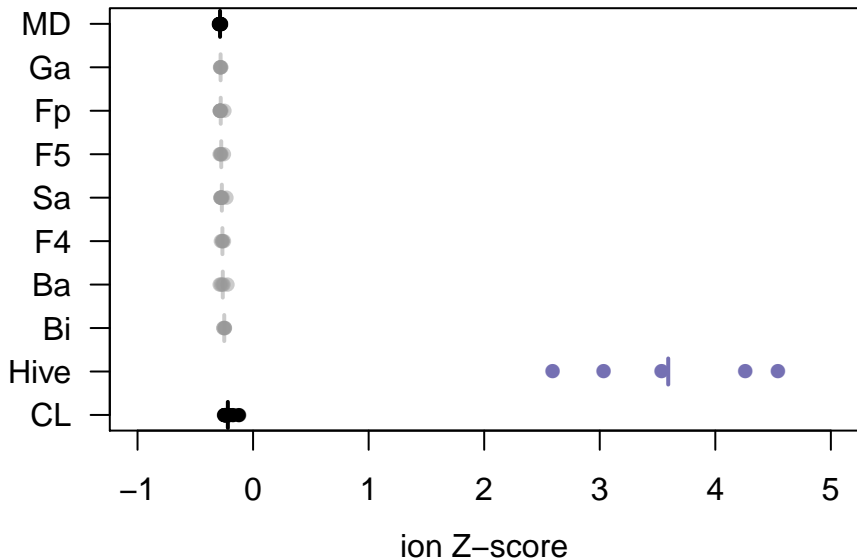

Supplement: S8 Data — (ZIP) [file pbio.2003467.s008.zip › Z-score_plots/127 microbial product trans-4-Hydroxycyclohexanecarboxylate.pdf]

**Octanoic acid\***  
**# 128 143.107 microbial product**

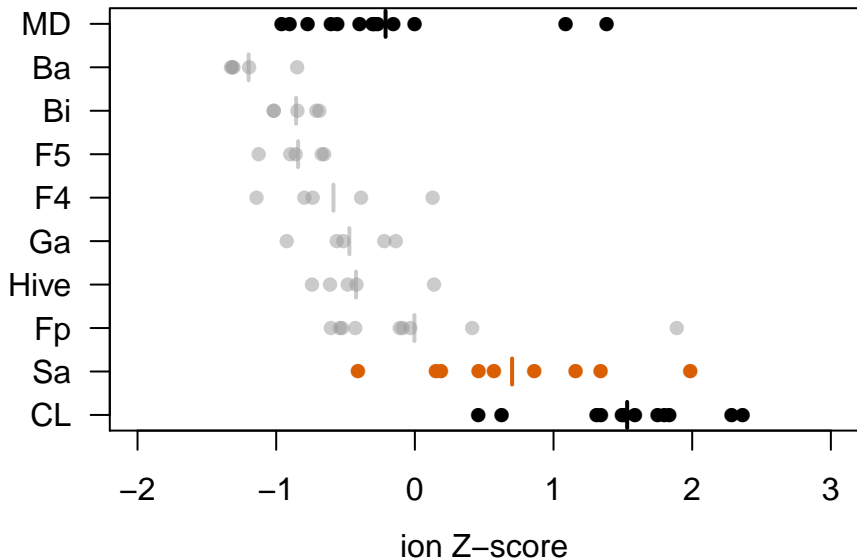

Supplement: S8 Data — (ZIP) [file pbio.2003467.s008.zip › Z-score_plots/128 microbial product Octanoic acid.pdf]

**alpha-ketoglutarate\***  
**# 131 145.014 microbial substrate**

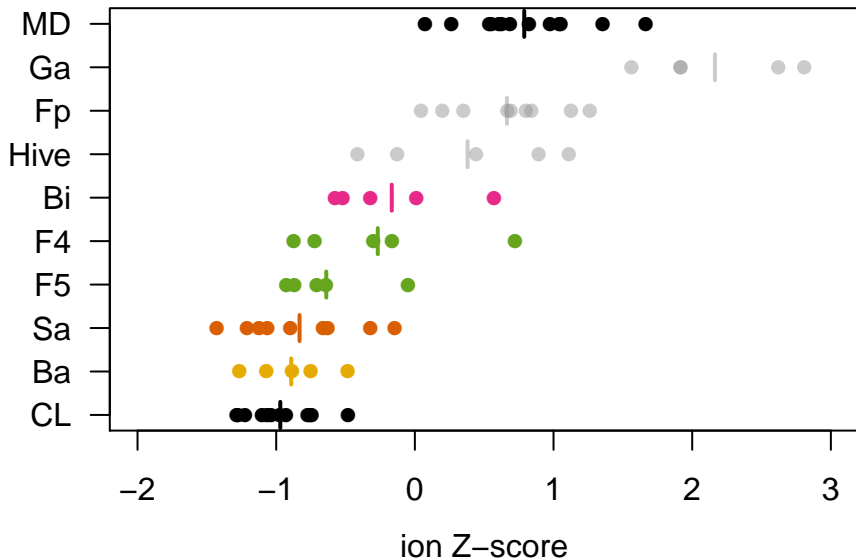

Supplement: S8 Data — (ZIP) [file pbio.2003467.s008.zip › Z-score_plots/131 microbial substrate alpha-ketoglutarate.pdf]

# 5-Methylthiopentanaldoxime

# 138 146.063 microbial substrate

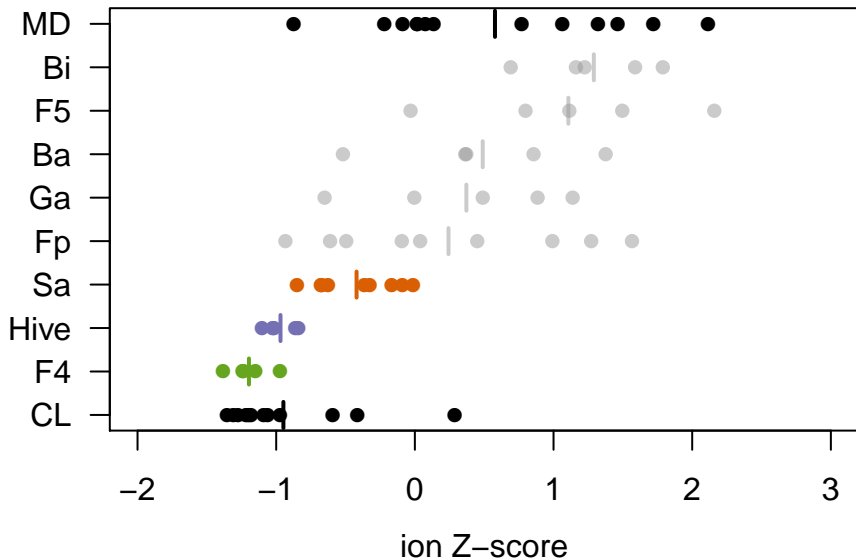

Supplement: S8 Data — (ZIP) [file pbio.2003467.s008.zip › Z-score_plots/138 microbial substrate 5-Methylthiopentanaldoxime.pdf]

**2-hydroxyglutarate\***  
**# 139 147.029 microbial product**

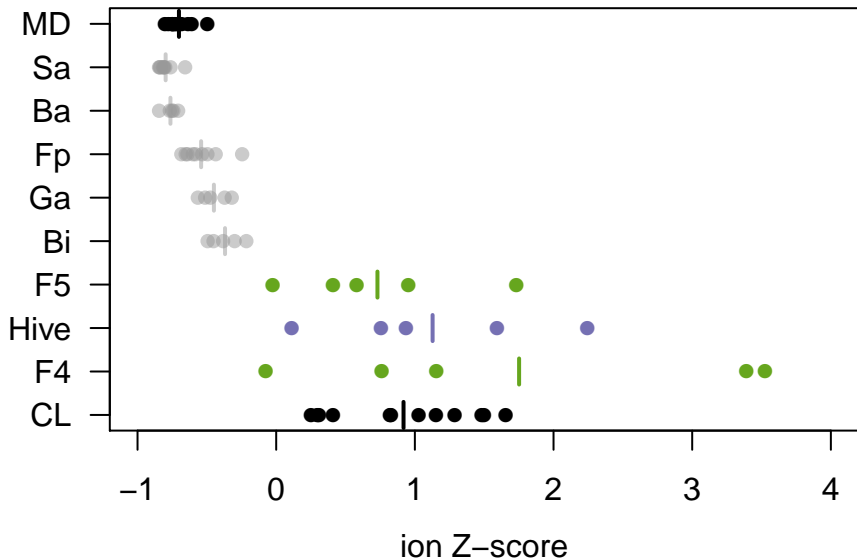

Supplement: S8 Data — (ZIP) [file pbio.2003467.s008.zip › Z-score_plots/139 microbial product 2-hydroxyglutarate.pdf]

**Cinnamic acid\***  
**# 140 147.045 microbial product**

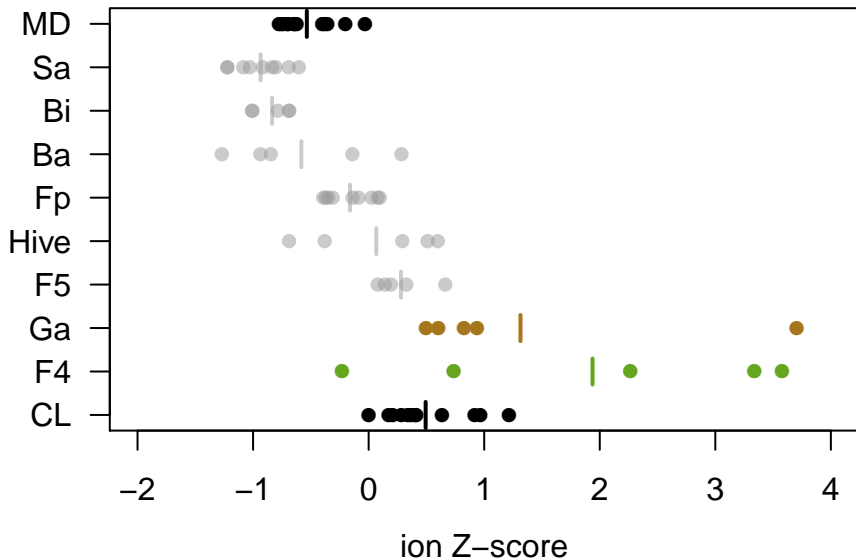

Supplement: S8 Data — (ZIP) [file pbio.2003467.s008.zip › Z-score_plots/140 microbial product Cinnamic acid.pdf]

**Xylose\***

**# 146 149.045 microbial product**

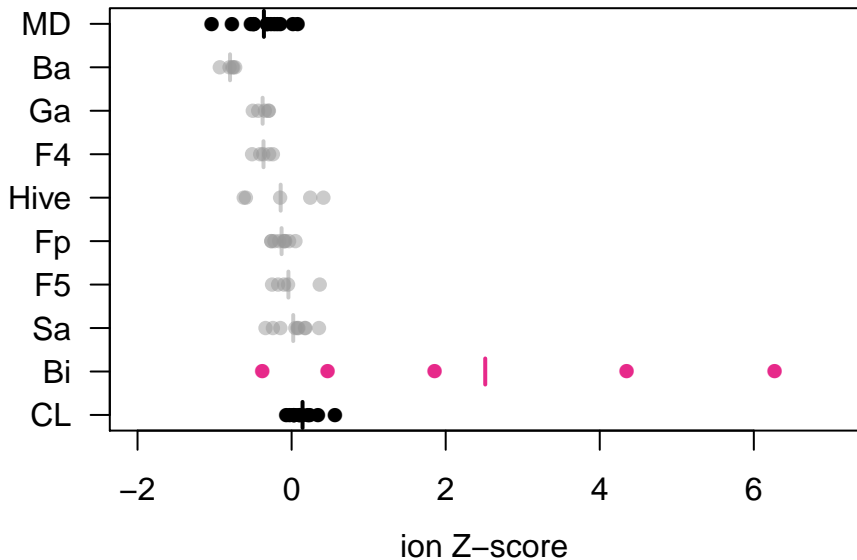

Supplement: S8 Data — (ZIP) [file pbio.2003467.s008.zip › Z-score_plots/146 microbial product Xylose.pdf]

**Methyl-4-aminobenzoate\***  
**# 149 150.055 microbial product**

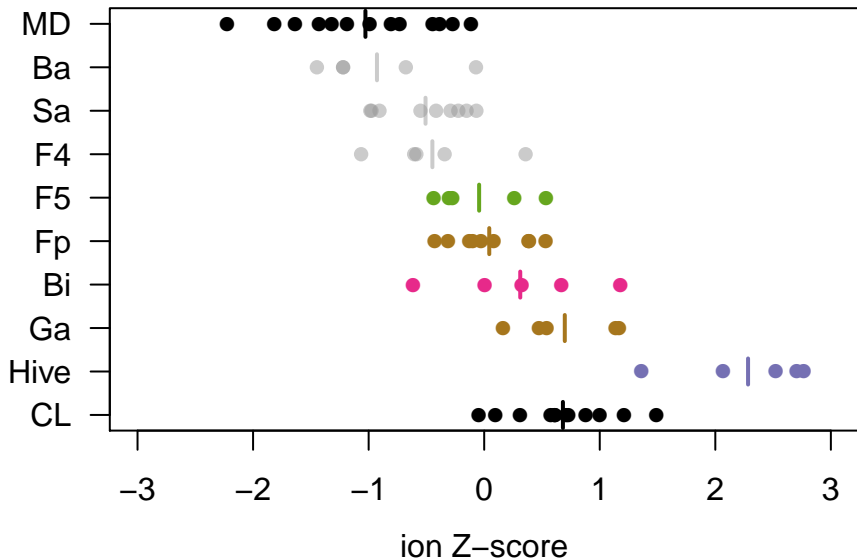

Supplement: S8 Data — (ZIP) [file pbio.2003467.s008.zip › Z-score_plots/149 microbial product Methyl-4-aminobenzoate.pdf]

# Glycine\*

# 15 74.0243 microbial substrate

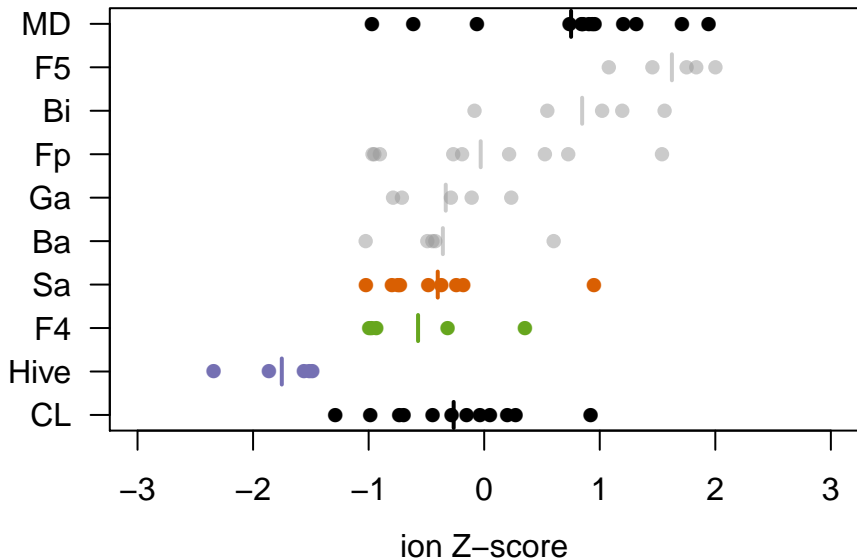

Supplement: S8 Data — (ZIP) [file pbio.2003467.s008.zip › Z-score_plots/15 microbial substrate Glycine.pdf]

**1,2-Diamino-4-nitrobenzene\***  
**# 156 152.045 microbial product**

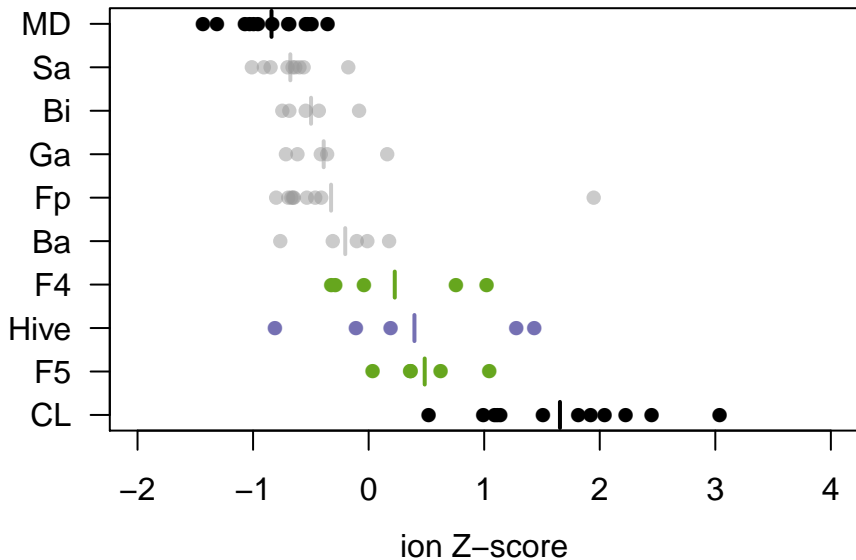

Supplement: S8 Data — (ZIP) [file pbio.2003467.s008.zip › Z-score_plots/156 microbial product 1,2-Diamino-4-nitrobenzene.pdf]

**Propanoyl phosphate\***  
**# 159 152.995 microbial product**

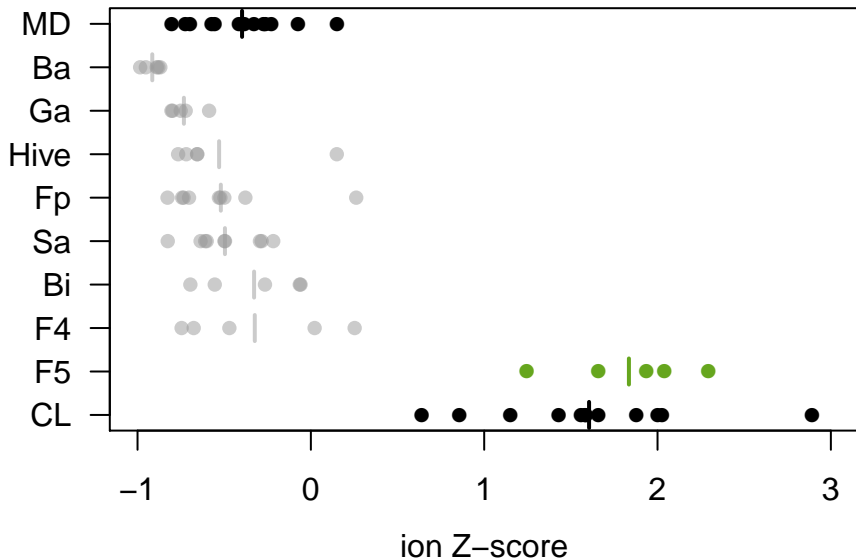

Supplement: S8 Data — (ZIP) [file pbio.2003467.s008.zip › Z-score_plots/159 microbial product Propanoyl phosphate.pdf]

**Vanillyl alcohol\***  
**# 161 153.055 microbial product**

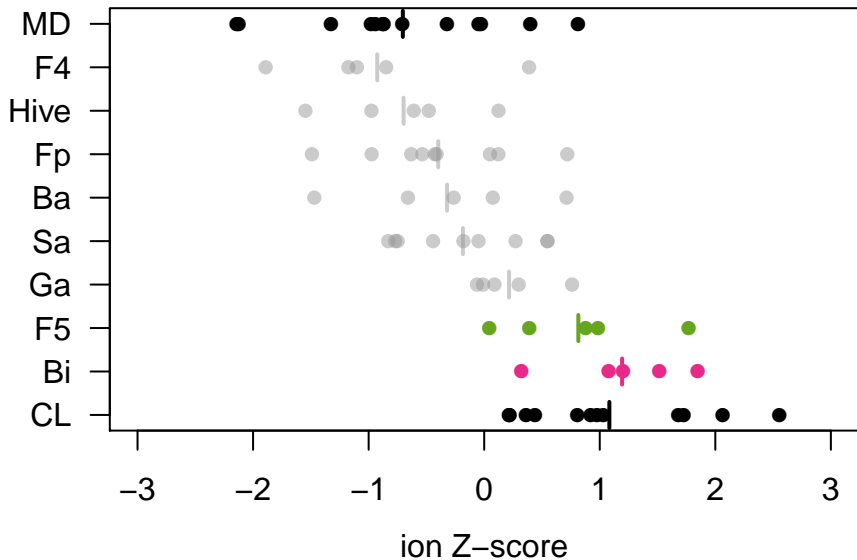

Supplement: S8 Data — (ZIP) [file pbio.2003467.s008.zip › Z-score_plots/161 microbial product Vanillyl alcohol.pdf]

# Orotate\*

# 166 155.009 microbial substrate

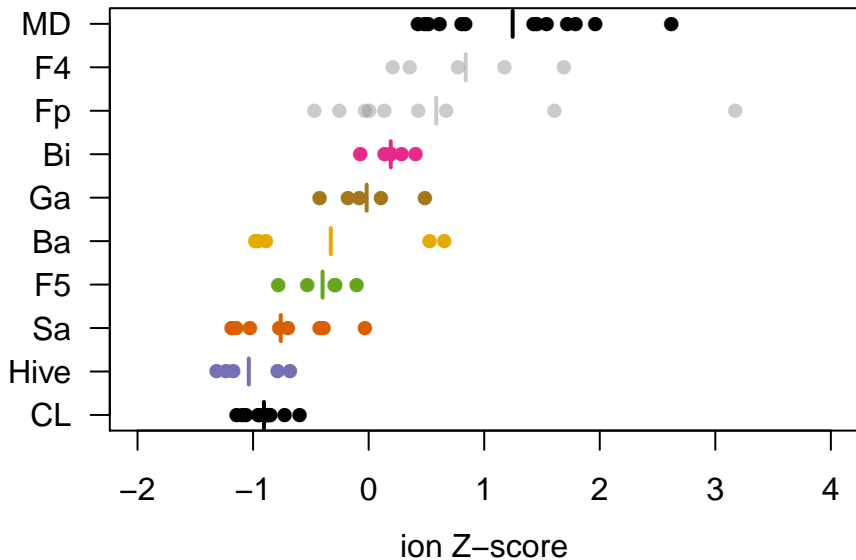

Supplement: S8 Data — (ZIP) [file pbio.2003467.s008.zip › Z-score_plots/166 microbial substrate Orotate.pdf]

**3-Methyl-cis,cis-muconate\***  
**# 167 155.034 microbial product**

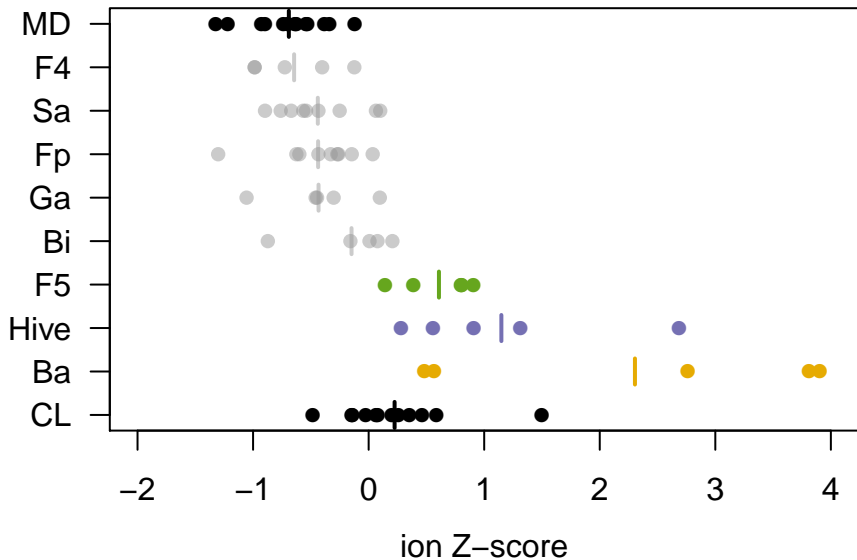

Supplement: S8 Data — (ZIP) [file pbio.2003467.s008.zip › Z-score_plots/167 microbial product 3-Methyl-cis,cis-muconate.pdf]

**4-Imidazolone-5-propanoate\***  
**# 168 155.046 microbial product**

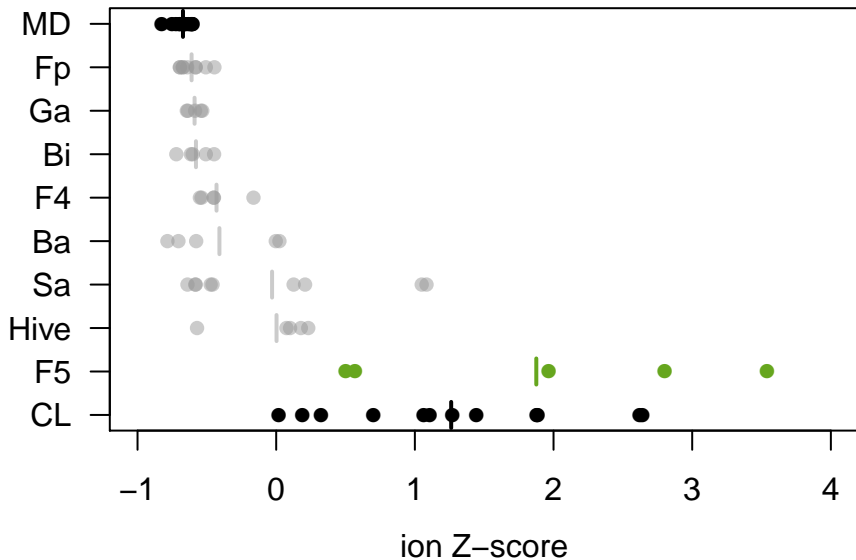

Supplement: S8 Data — (ZIP) [file pbio.2003467.s008.zip › Z-score_plots/168 microbial product 4-Imidazolone-5-propanoate.pdf]

**Nonane-4,6-dione\***  
**# 169 155.107 microbial substrate**

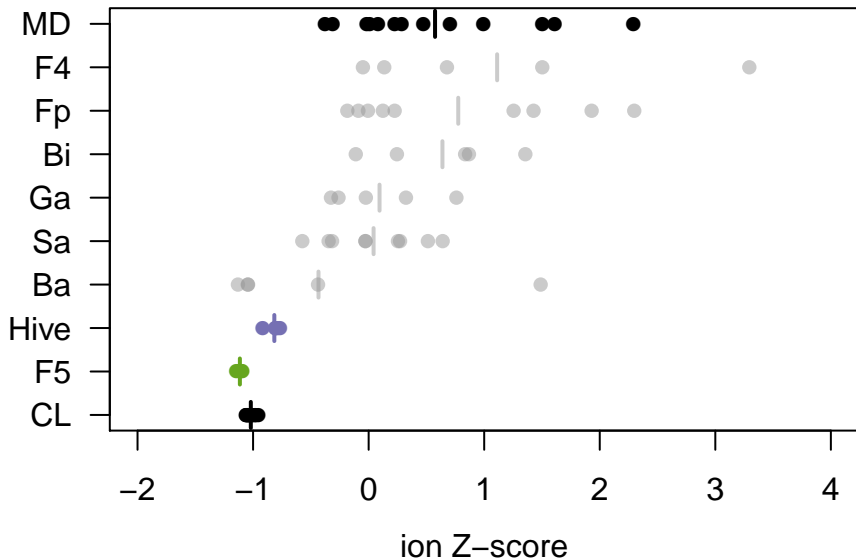

Supplement: S8 Data — (ZIP) [file pbio.2003467.s008.zip › Z-score_plots/169 microbial substrate Nonane-4,6-dione.pdf]

**2-Aminomuconate\***  
**# 171 156.030 microbial product**

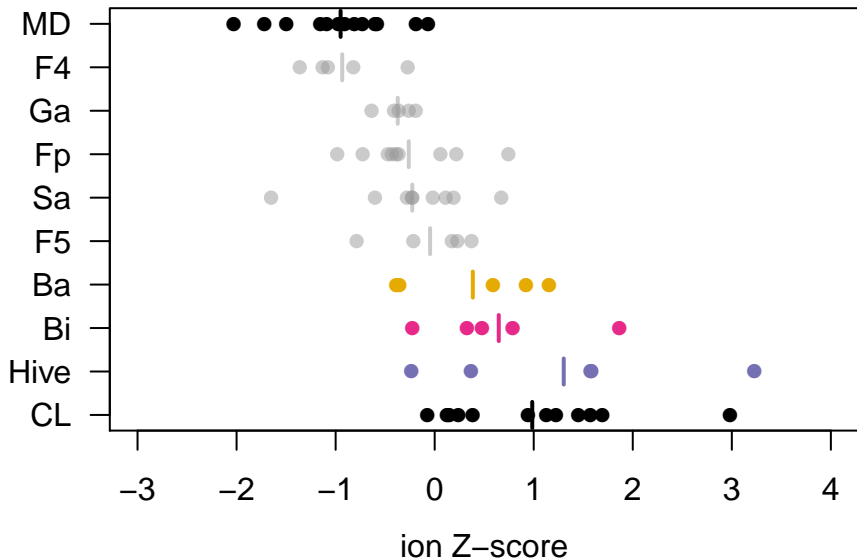

Supplement: S8 Data — (ZIP) [file pbio.2003467.s008.zip › Z-score_plots/171 microbial product 2-Aminomuconate.pdf]

# Homostachydrine\*

# 173 156.102 microbial substrate

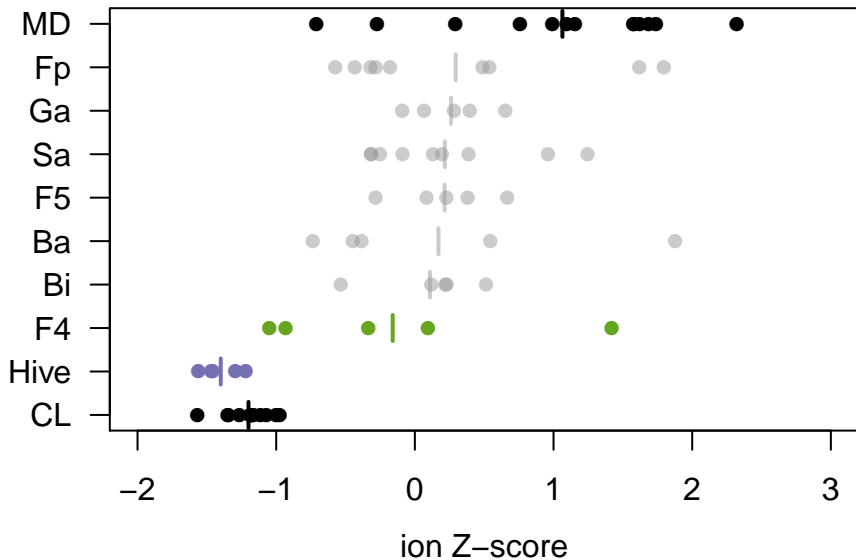

Supplement: S8 Data — (ZIP) [file pbio.2003467.s008.zip › Z-score_plots/173 microbial substrate Homostachydrine.pdf]

# 2-Maleylacetate\*

# 174 157.013 microbial product

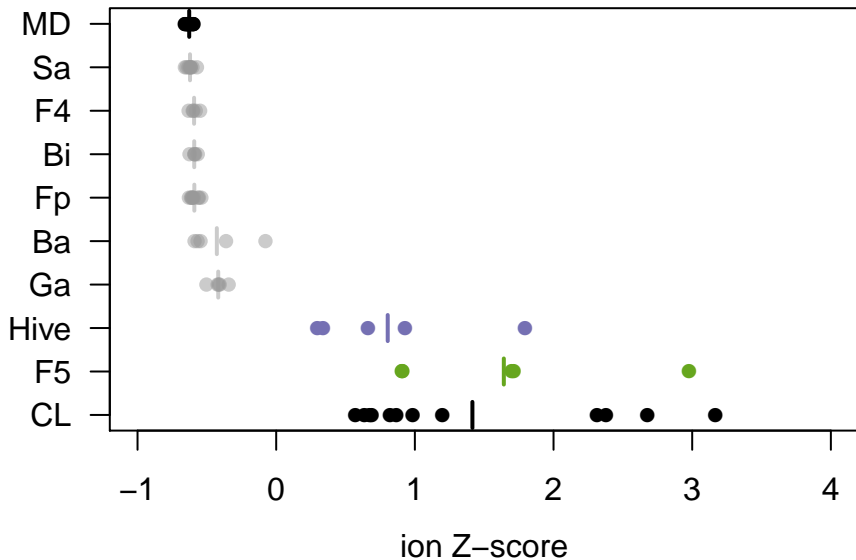

Supplement: S8 Data — (ZIP) [file pbio.2003467.s008.zip › Z-score_plots/174 microbial product 2-Maleylacetate.pdf]

# Allantoin\*

# 175 157.036 microbial product

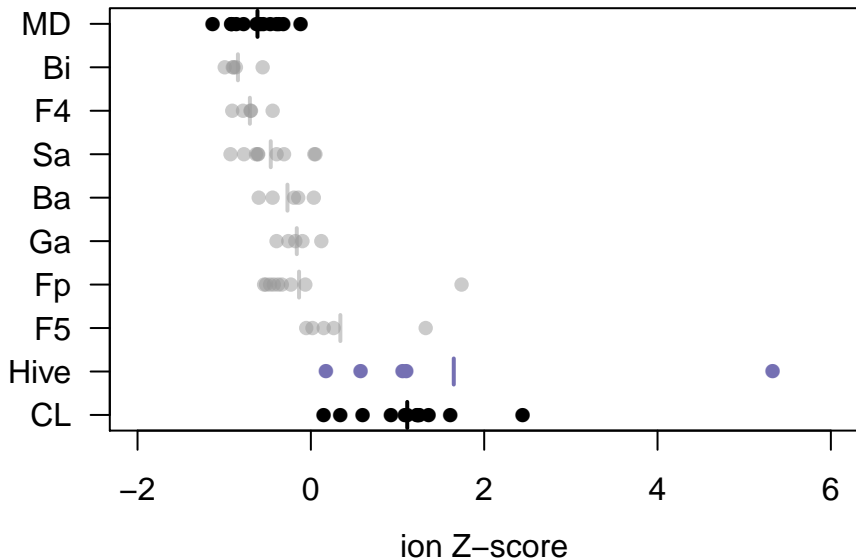

Supplement: S8 Data — (ZIP) [file pbio.2003467.s008.zip › Z-score_plots/175 microbial product Allantoin.pdf]

**2-Isopropylmaleate\***  
**# 176 157.050 microbial product**

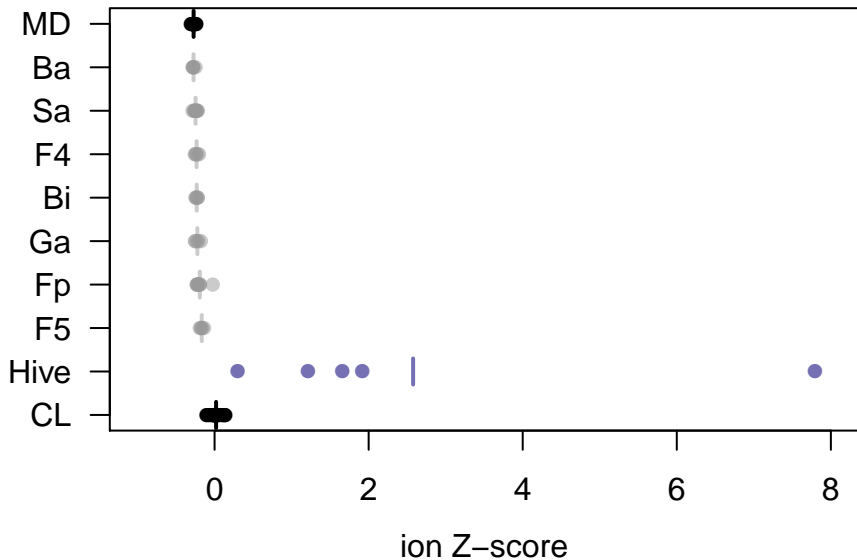

Supplement: S8 Data — (ZIP) [file pbio.2003467.s008.zip › Z-score_plots/176 microbial product 2-Isopropylmaleate.pdf]

**Ethyl 3-oxohexanoate\***  
**# 177 157.086 microbial product**

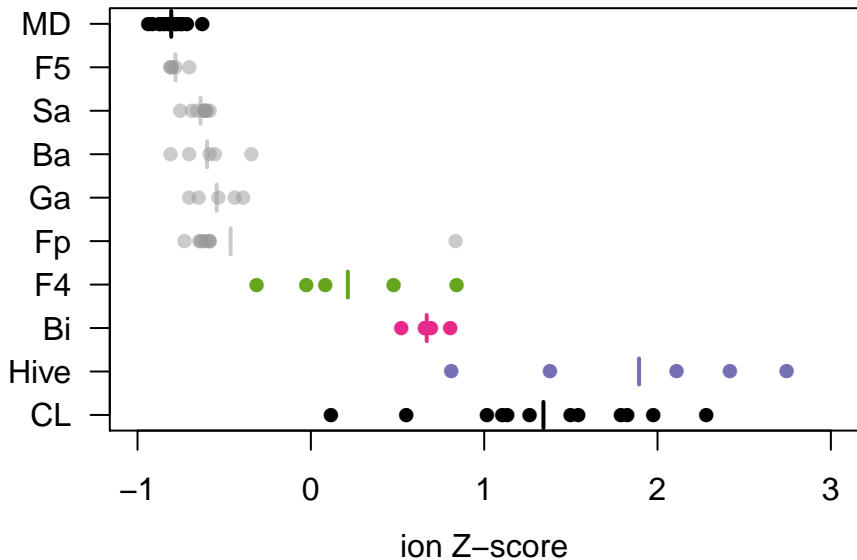

Supplement: S8 Data — (ZIP) [file pbio.2003467.s008.zip › Z-score_plots/177 microbial product Ethyl 3-oxohexanoate.pdf]

**4-Methylene-L-glutamate\***  
**# 179 158.045 microbial product**

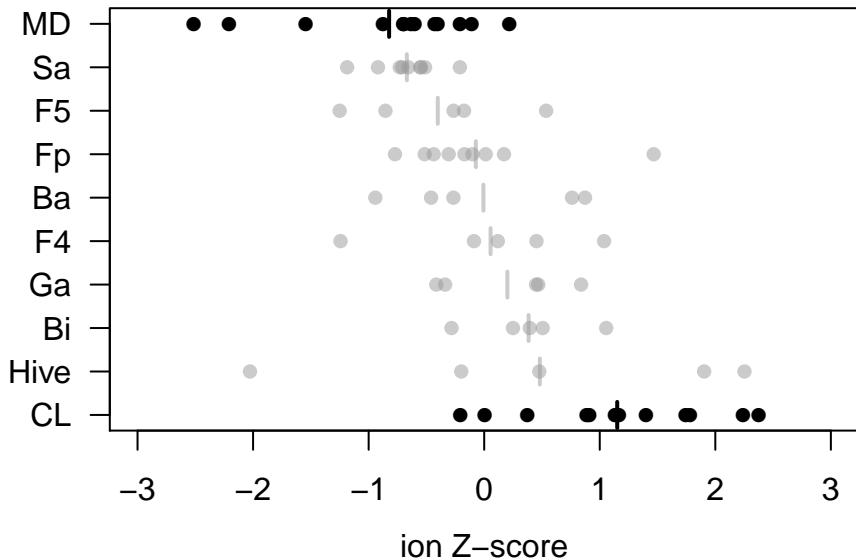

Supplement: S8 Data — (ZIP) [file pbio.2003467.s008.zip › Z-score_plots/179 microbial product 4-Methylene-L-glutamate.pdf]

**Pimelate\***

**# 183 159.066 microbial product**

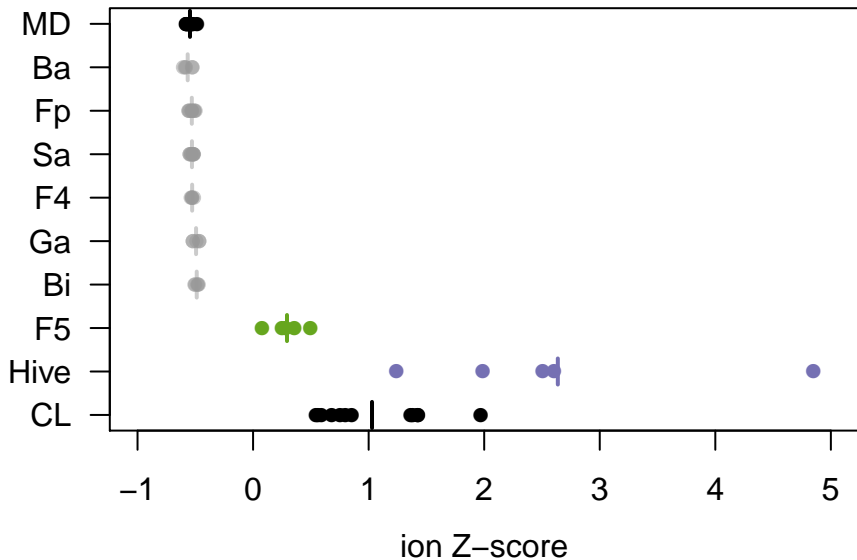

Supplement: S8 Data — (ZIP) [file pbio.2003467.s008.zip › Z-score_plots/183 microbial product Pimelate.pdf]

**D-Alanyl-D-alanine\***  
**# 184 159.076 microbial product**

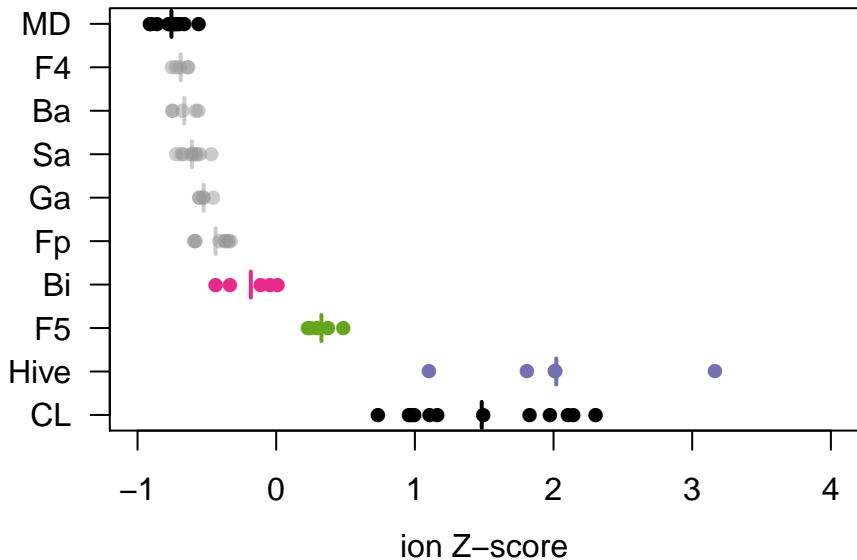

Supplement: S8 Data — (ZIP) [file pbio.2003467.s008.zip › Z-score_plots/184 microbial product D-Alanyl-D-alanine.pdf]

**L-2-Aminoadipate\***  
**# 187 160.061 microbial product**

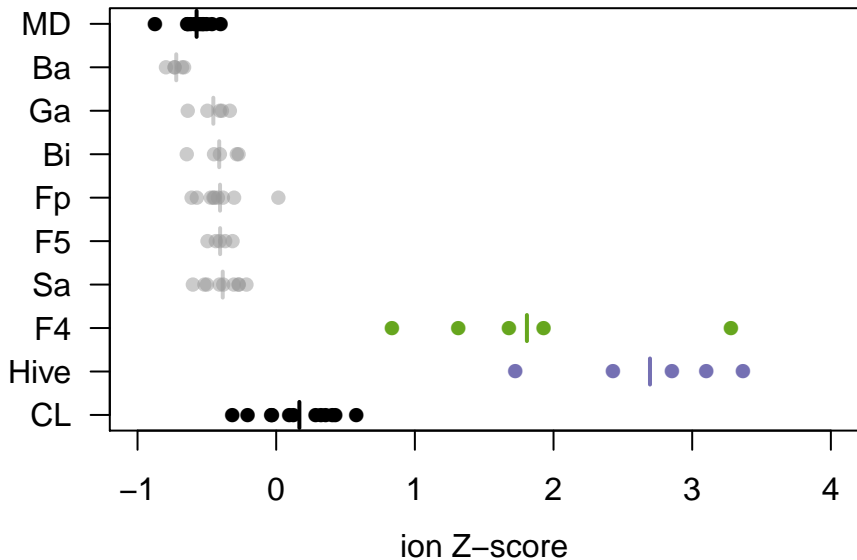

Supplement: S8 Data — (ZIP) [file pbio.2003467.s008.zip › Z-score_plots/187 microbial product L-2-Aminoadipate.pdf]

# Indole-3-ethanol\*

# 188 160.076 microbial product

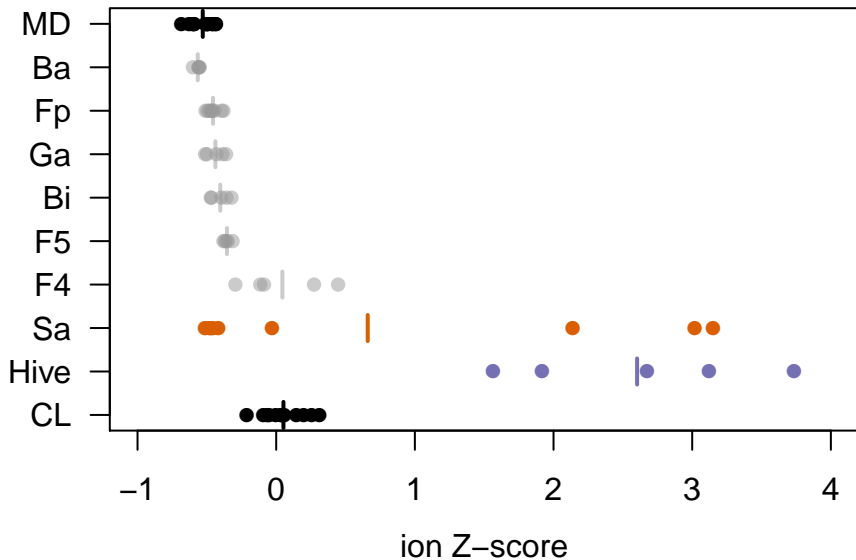

Supplement: S8 Data — (ZIP) [file pbio.2003467.s008.zip › Z-score_plots/188 microbial product Indole-3-ethanol.pdf]

**Anhydrogalactose\***  
**# 189 161.045 microbial substrate**

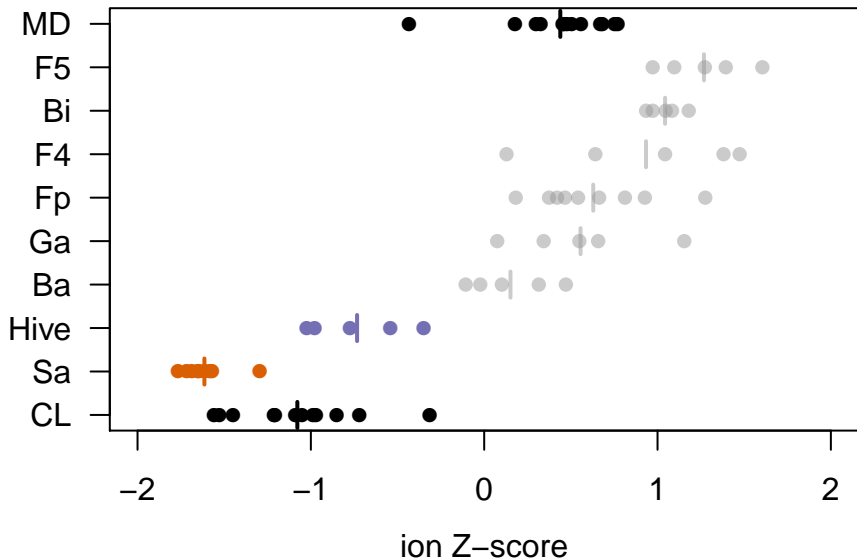

Supplement: S8 Data — (ZIP) [file pbio.2003467.s008.zip › Z-score_plots/189 microbial substrate Anhydrogalactose.pdf]

**p-coumarate\***  
**# 194 163.039 microbial substrate**

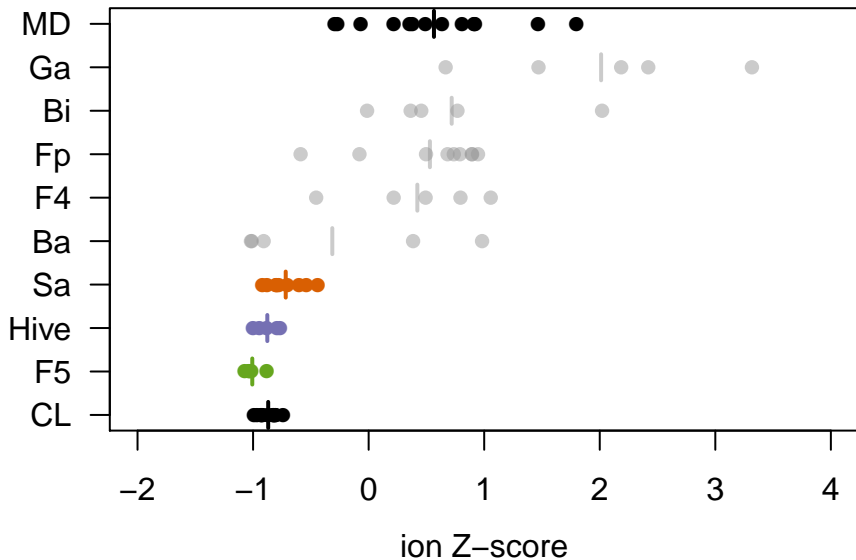

Supplement: S8 Data — (ZIP) [file pbio.2003467.s008.zip › Z-score_plots/194 microbial substrate p-coumarate.pdf]

Ribonate\*

# 199 165.040 microbial product

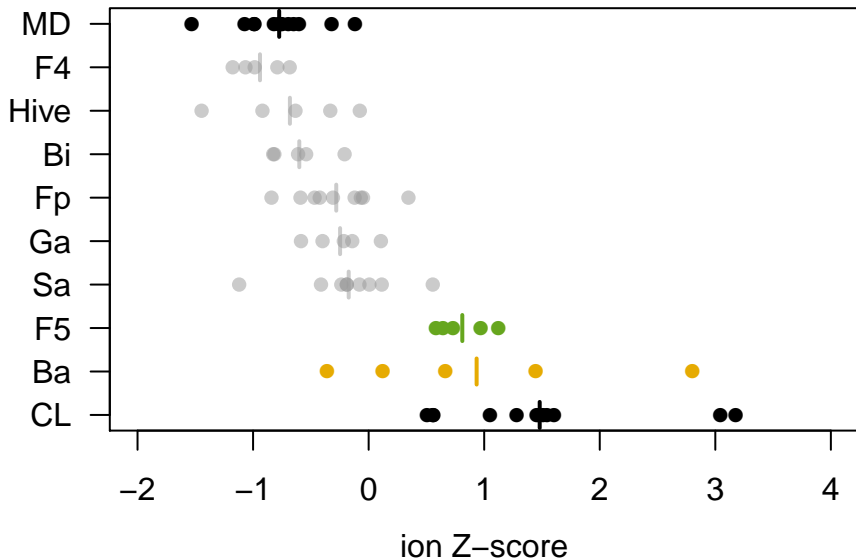

Supplement: S8 Data — (ZIP) [file pbio.2003467.s008.zip › Z-score_plots/199 microbial product Ribonate.pdf]

**3-(2-Hydroxyphenyl)propanoate\***  
**# 200 165.055 microbial product**

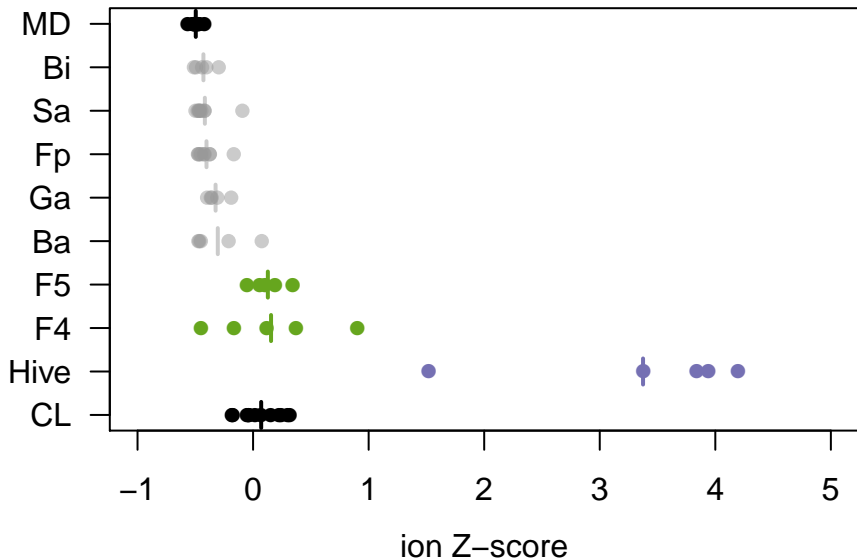

Supplement: S8 Data — (ZIP) [file pbio.2003467.s008.zip › Z-score_plots/200 microbial product 3-(2-Hydroxyphenyl)propanoate.pdf]

**6-endo-Hydroxycineole\***  
**# 206 169.123 microbial product**

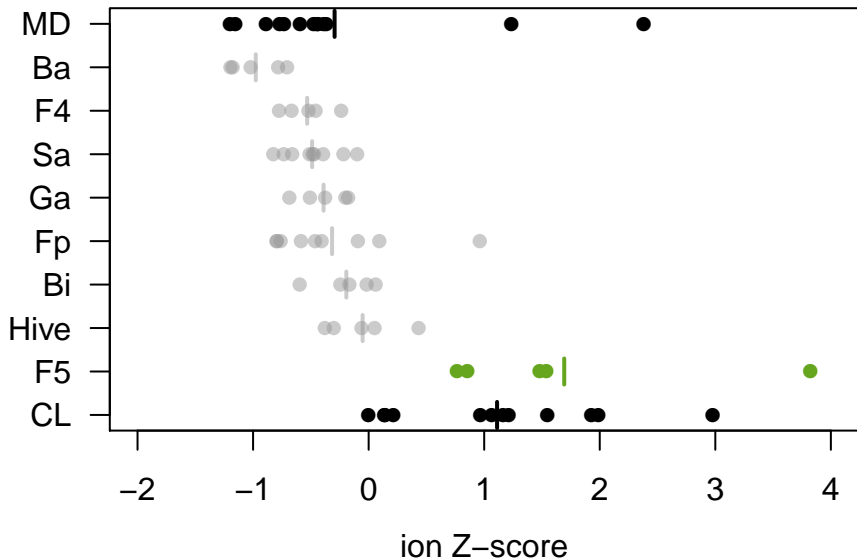

Supplement: S8 Data — (ZIP) [file pbio.2003467.s008.zip › Z-score_plots/206 microbial product 6-endo-Hydroxycineole.pdf]

# Metronidazole

# 209 170.057 microbial product

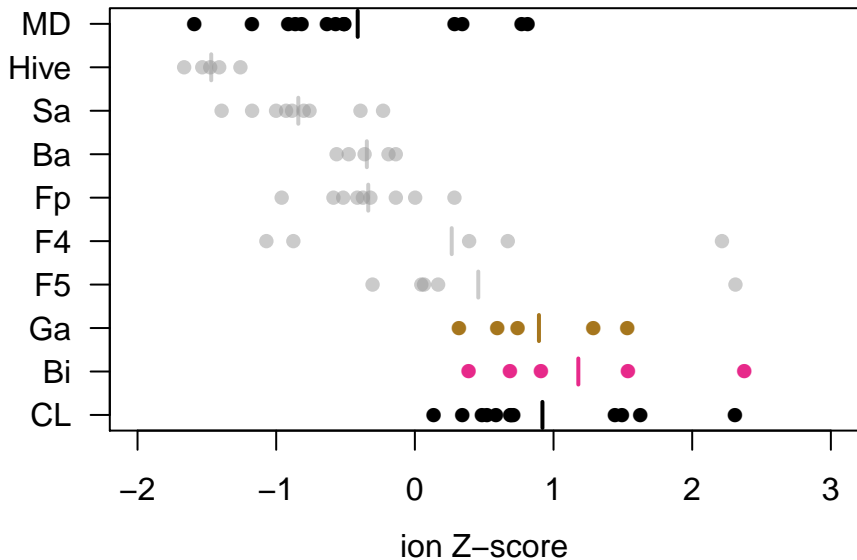

Supplement: S8 Data — (ZIP) [file pbio.2003467.s008.zip › Z-score_plots/209 microbial product Metronidazole.pdf]

# Hydantoin-5-propionate\*

# 213 171.041 microbial product

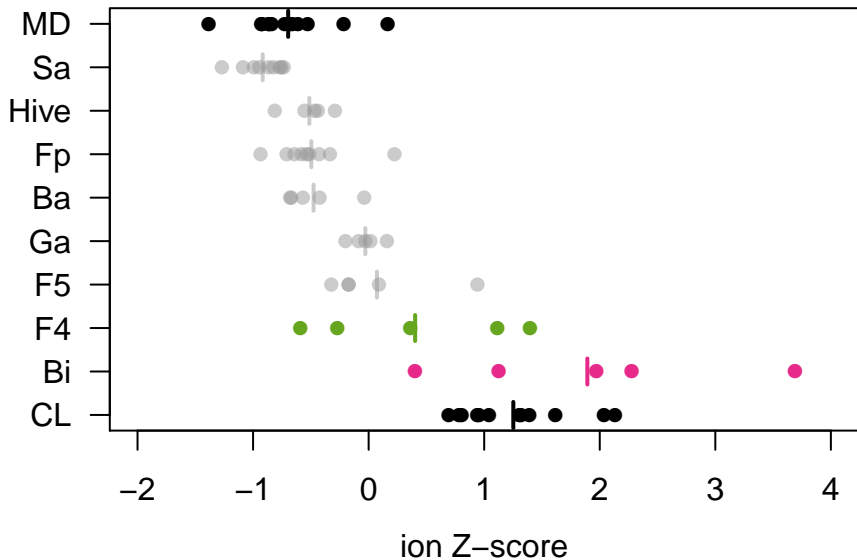

Supplement: S8 Data — (ZIP) [file pbio.2003467.s008.zip › Z-score_plots/213 microbial product Hydantoin-5-propionate.pdf]

**cis-Aconitate\***  
**# 220 173.009 microbial substrate**

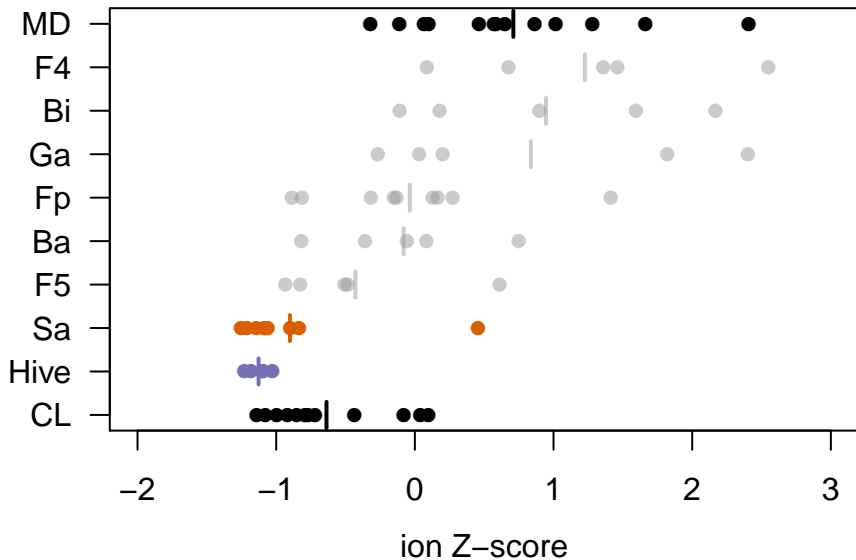

Supplement: S8 Data — (ZIP) [file pbio.2003467.s008.zip › Z-score_plots/220 microbial substrate cis-Aconitate.pdf]

**Quinoxaline-2-carboxylic acid\***  
**# 221 173.034 microbial substrate**

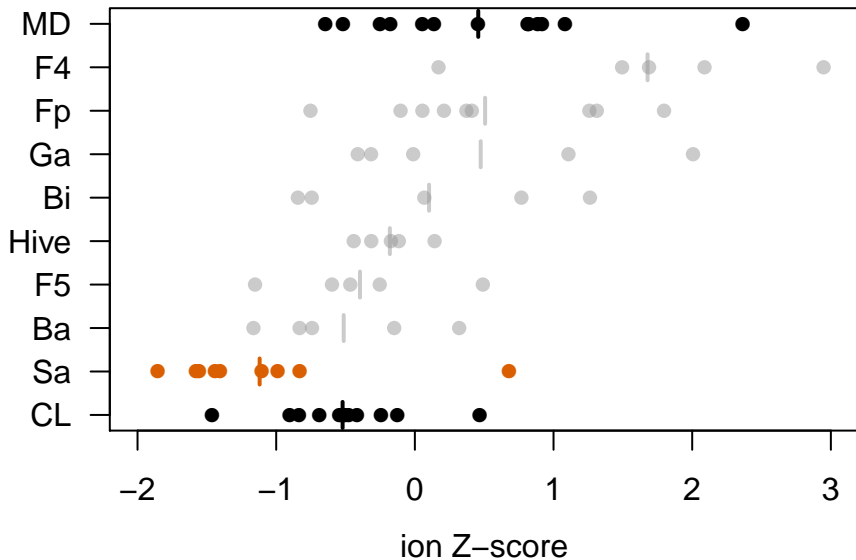

Supplement: S8 Data — (ZIP) [file pbio.2003467.s008.zip › Z-score_plots/221 microbial substrate Quinoxaline-2-carboxylic acid.pdf]

**Suberic acid\***  
**# 224 173.081 microbial product**

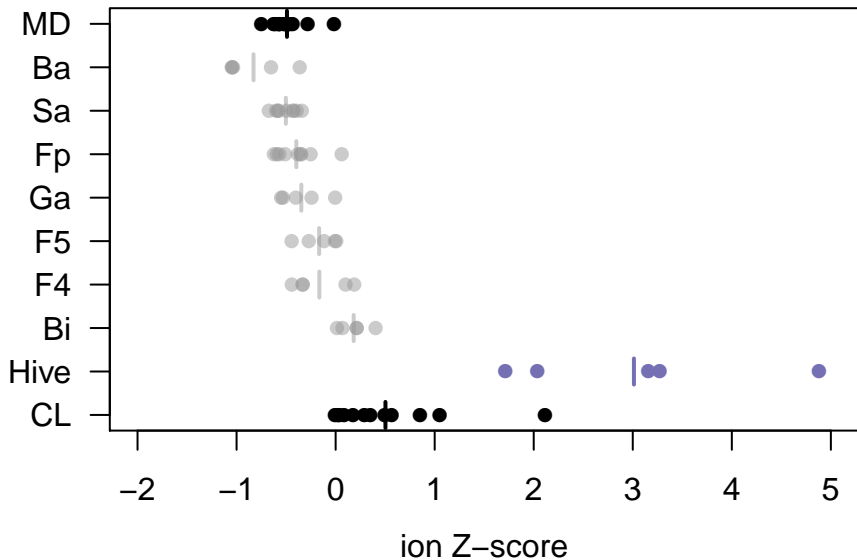

Supplement: S8 Data — (ZIP) [file pbio.2003467.s008.zip › Z-score_plots/224 microbial product Suberic acid.pdf]

**N-Acetyl-L-aspartate\***  
**# 227 174.040 microbial product**

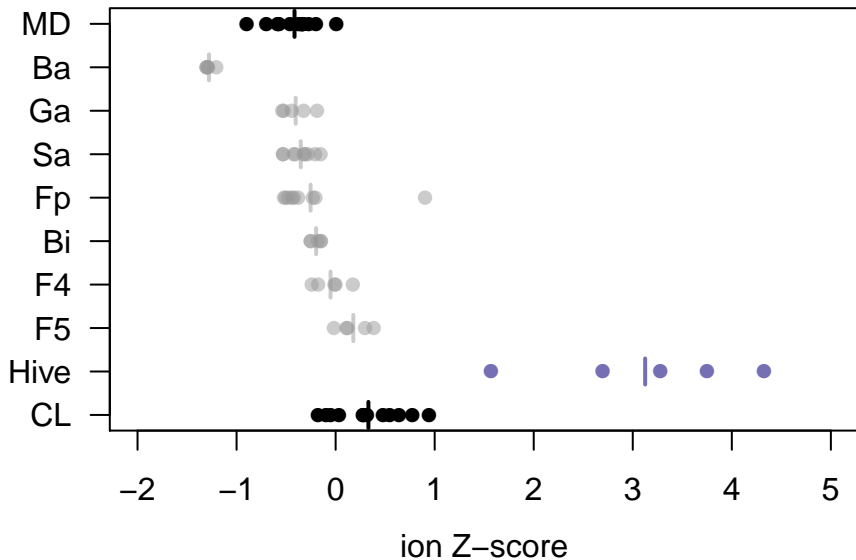

Supplement: S8 Data — (ZIP) [file pbio.2003467.s008.zip › Z-score_plots/227 microbial product N-Acetyl-L-aspartate.pdf]

# Calystegin B2

# 229 174.077 microbial substrate

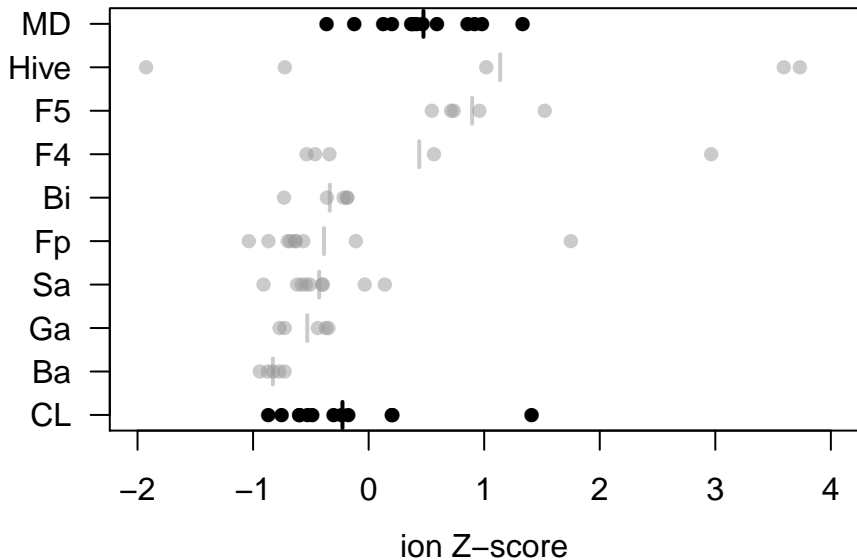

Supplement: S8 Data — (ZIP) [file pbio.2003467.s008.zip › Z-score_plots/229 microbial substrate Calystegin B2.pdf]

# Pyruvate\*

# 23 87.0086 microbial substrate

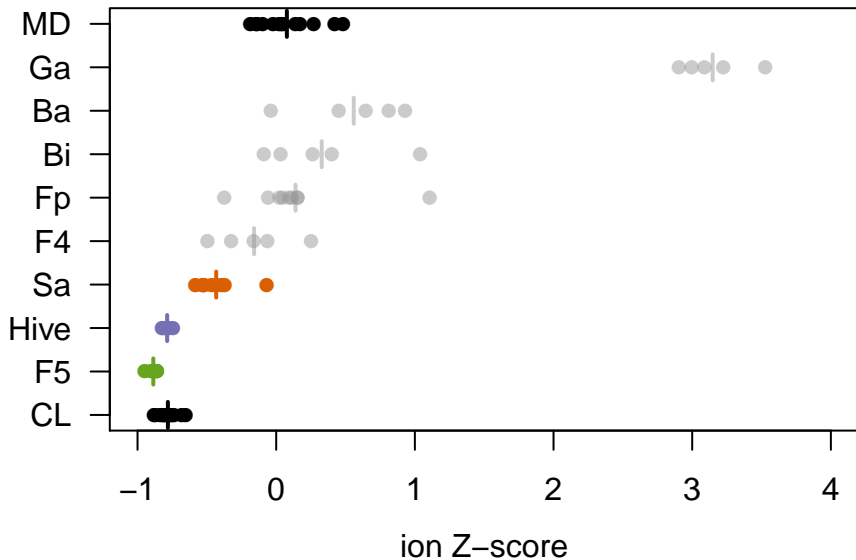

Supplement: S8 Data — (ZIP) [file pbio.2003467.s008.zip › Z-score_plots/23 microbial substrate Pyruvate.pdf]

**2-Hydroxy-3-oxoadipate\***  
**# 230 175.024 microbial product**

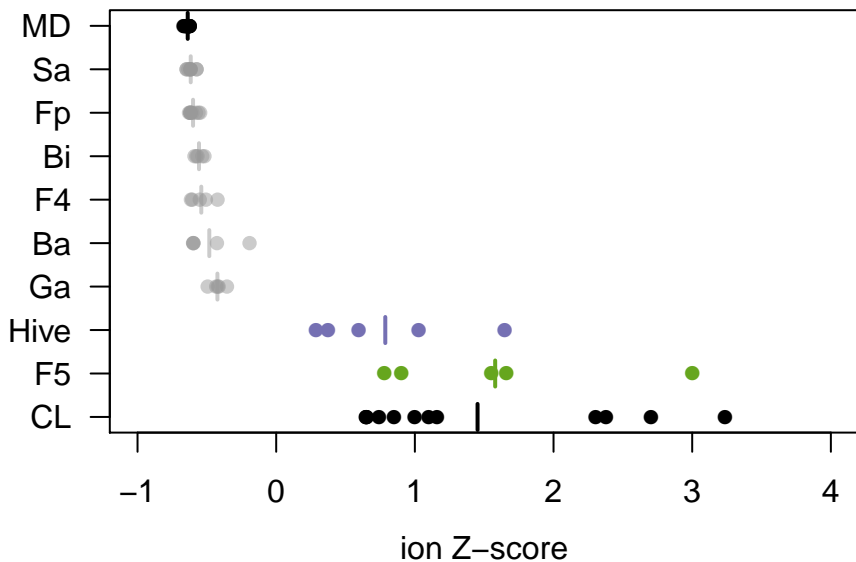

Supplement: S8 Data — (ZIP) [file pbio.2003467.s008.zip › Z-score_plots/230 microbial product 2-Hydroxy-3-oxoadipate.pdf]

**4-Hydroxyaminoquinoline N-oxide\***  
**# 231 175.050 microbial product**

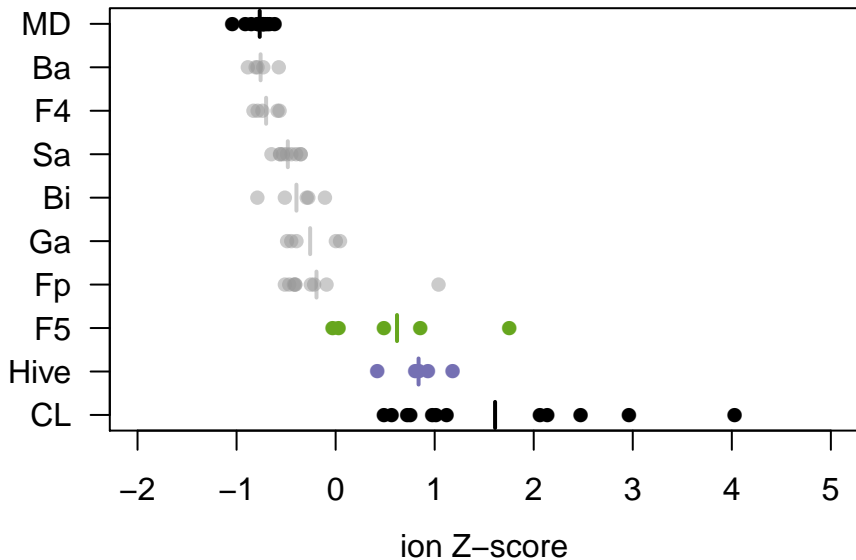

Supplement: S8 Data — (ZIP) [file pbio.2003467.s008.zip › Z-score_plots/231 microbial product 4-Hydroxyaminoquinoline N-oxide.pdf]

# Propofol\*

# 238 177.128 microbial product

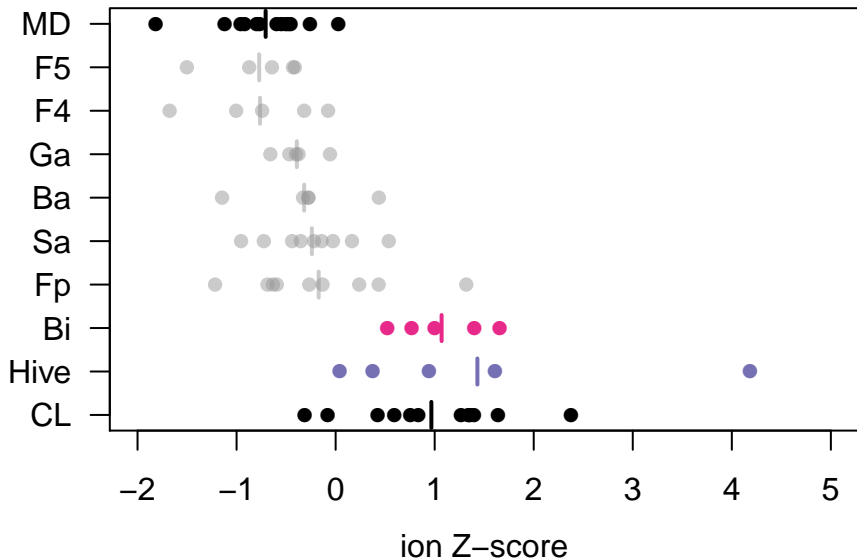

Supplement: S8 Data — (ZIP) [file pbio.2003467.s008.zip › Z-score_plots/238 microbial product Propofol.pdf]

**Butanoic acid\***  
**# 24 87.0449 microbial product**

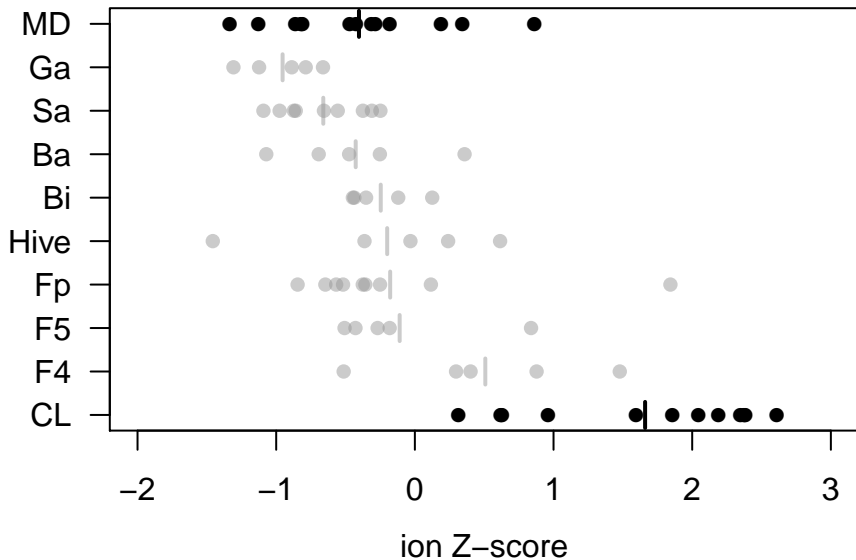

Supplement: S8 Data — (ZIP) [file pbio.2003467.s008.zip › Z-score_plots/24 microbial product Butanoic acid.pdf]

**3-(4-Hydroxyphenyl)pyruvate\***  
**# 242 179.034 microbial substrate**

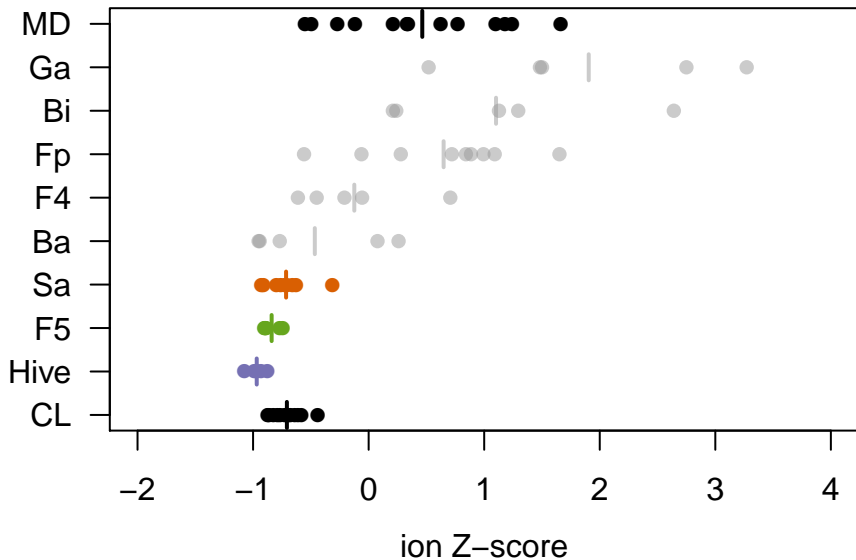

Supplement: S8 Data — (ZIP) [file pbio.2003467.s008.zip › Z-score_plots/242 microbial substrate 3-(4-Hydroxyphenyl)pyruvate.pdf]

**3-(3,4-Dihydroxyphenyl)propanoate\***  
**# 247 181.050 microbial product**

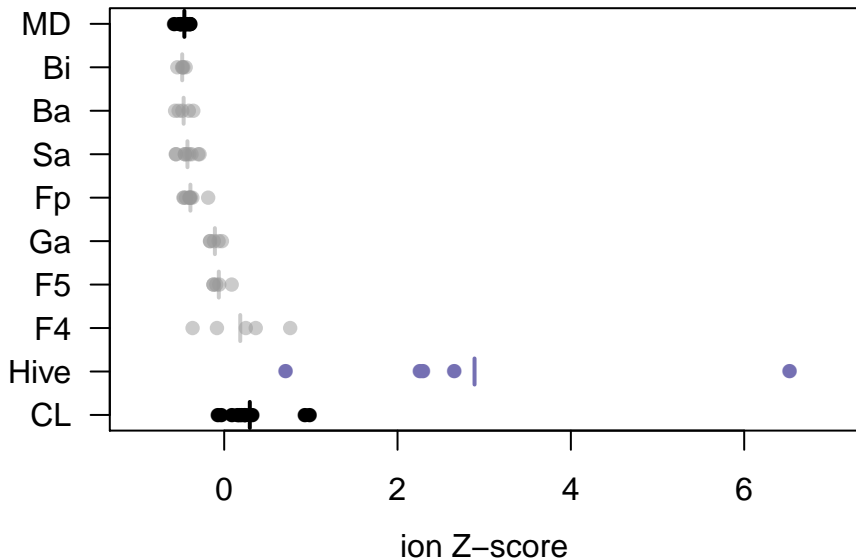

Supplement: S8 Data — (ZIP) [file pbio.2003467.s008.zip › Z-score_plots/247 microbial product 3-(3,4-Dihydroxyphenyl)propanoate.pdf]

**4-Amino-2-hydroxylamino-6-nitrotoluene**  
**# 249 182.056 microbial product**

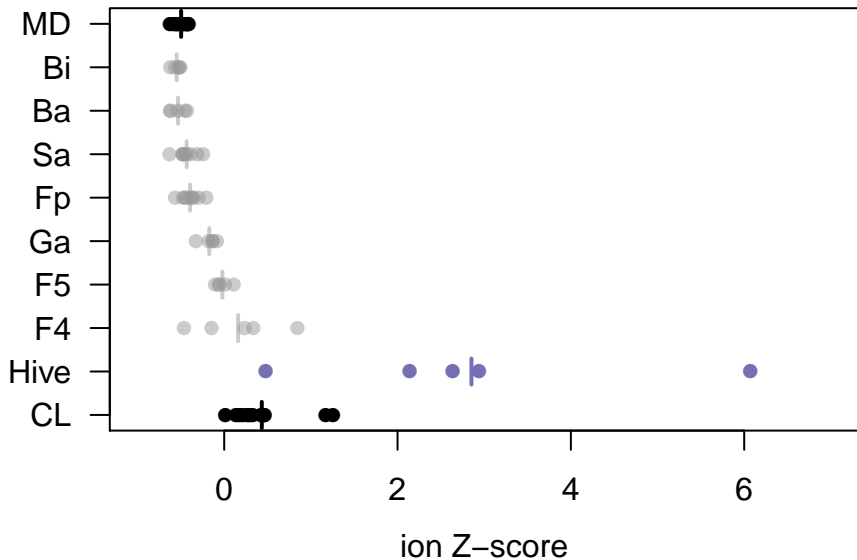

Supplement: S8 Data — (ZIP) [file pbio.2003467.s008.zip › Z-score_plots/249 microbial product 4-Amino-2-hydroxylamino-6-nitrotoluene.pdf]

**3,4-Dihydroxymandelate\***  
**# 252 183.029 microbial product**

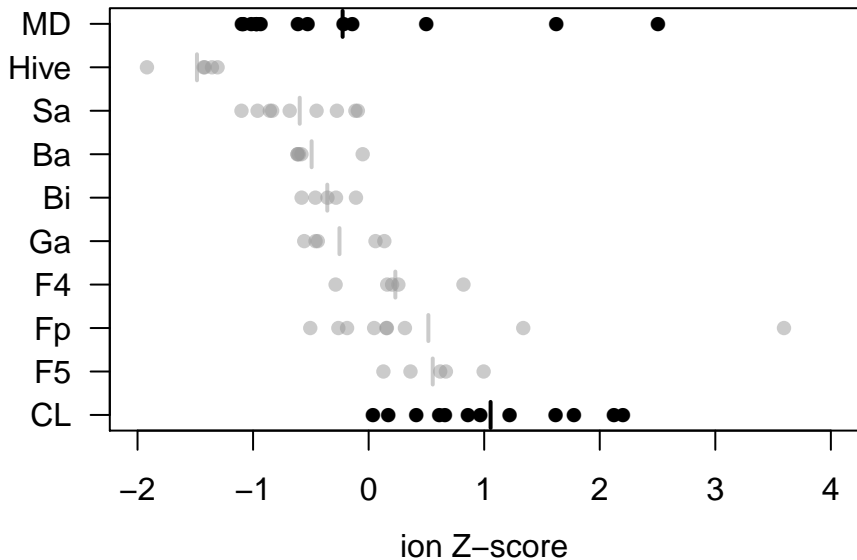

Supplement: S8 Data — (ZIP) [file pbio.2003467.s008.zip › Z-score_plots/252 microbial product 3,4-Dihydroxymandelate.pdf]

# Fuberidazole

# 253 183.055 microbial product

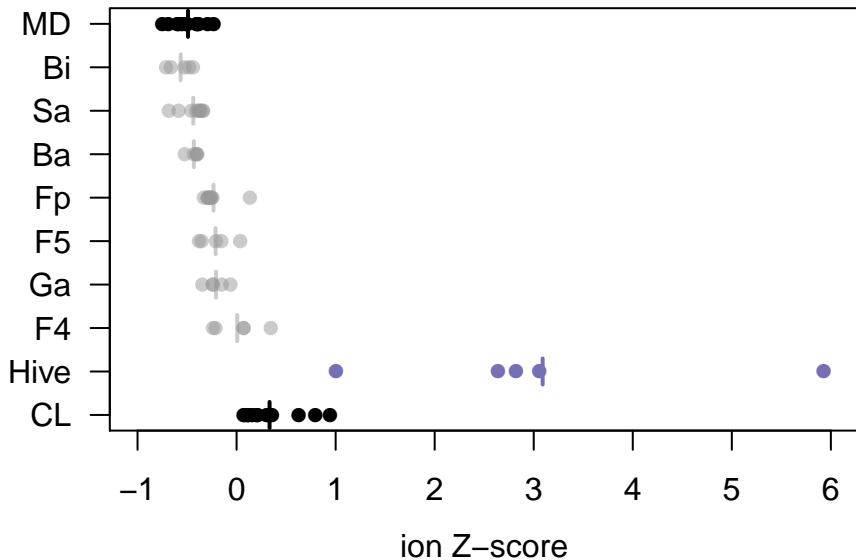

Supplement: S8 Data — (ZIP) [file pbio.2003467.s008.zip › Z-score_plots/253 microbial product Fuberidazole.pdf]

**5-exo-Hydroxy-1,2-campholide\***  
**# 254 183.102 microbial product**

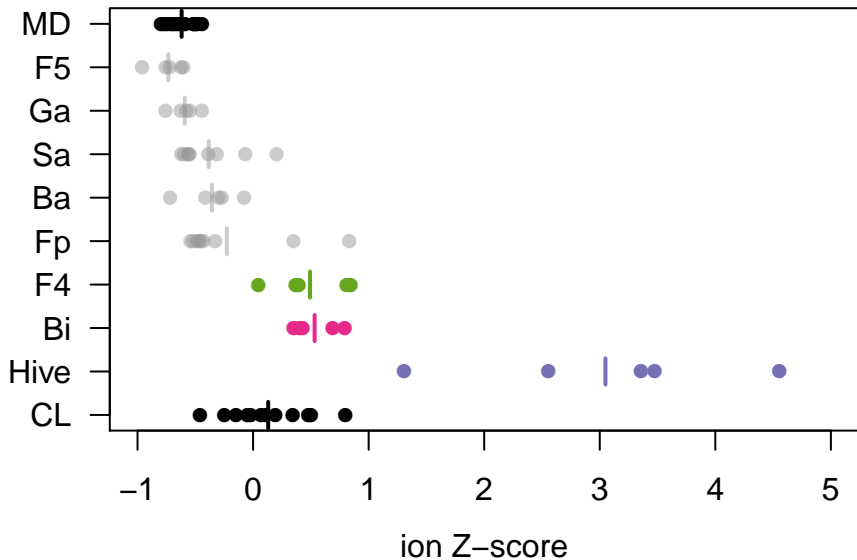

Supplement: S8 Data — (ZIP) [file pbio.2003467.s008.zip › Z-score_plots/254 microbial product 5-exo-Hydroxy-1,2-campholide.pdf]

# Citronellyl formate\*

# 255 183.139 microbial product

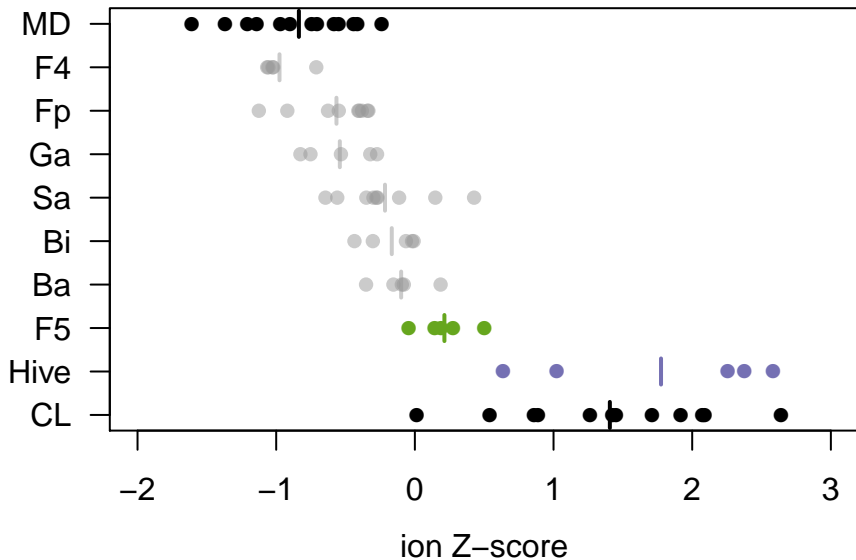

Supplement: S8 Data — (ZIP) [file pbio.2003467.s008.zip › Z-score_plots/255 microbial product Citronellyl formate.pdf]

# Isocarbamid

# 259 184.108 microbial product

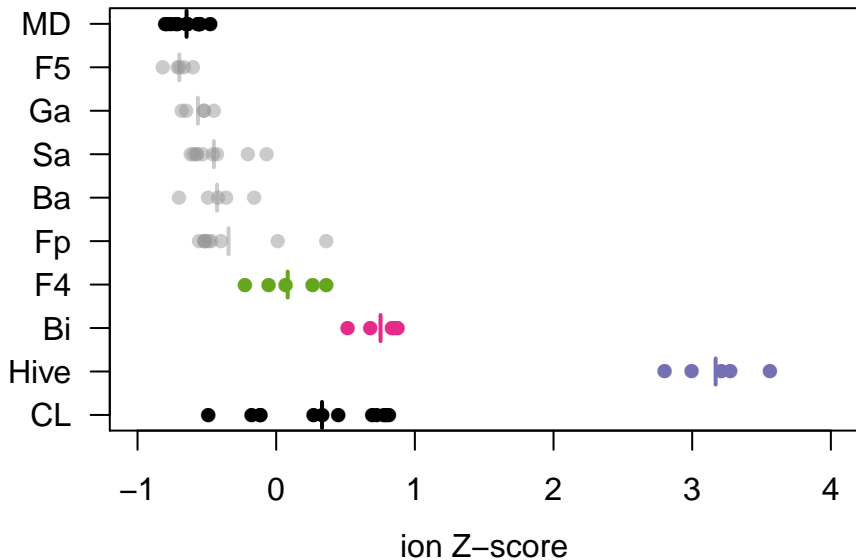

Supplement: S8 Data — (ZIP) [file pbio.2003467.s008.zip › Z-score_plots/259 microbial product Isocarbamid.pdf]

# AMPA

# 260 185.056 microbial substrate

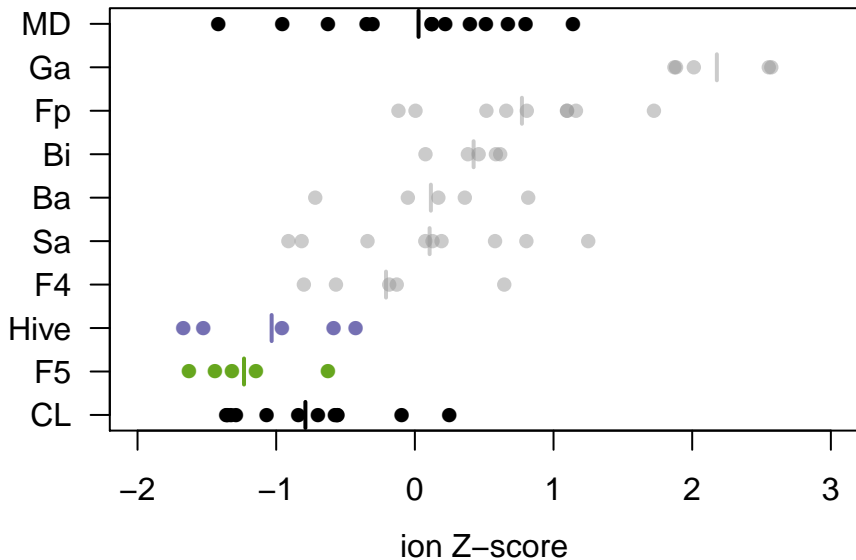

Supplement: S8 Data — (ZIP) [file pbio.2003467.s008.zip › Z-score_plots/260 microbial substrate AMPA.pdf]

**cis-2-Carboxycyclohexyl-acetic acid**  
**# 261 185.082 microbial product**

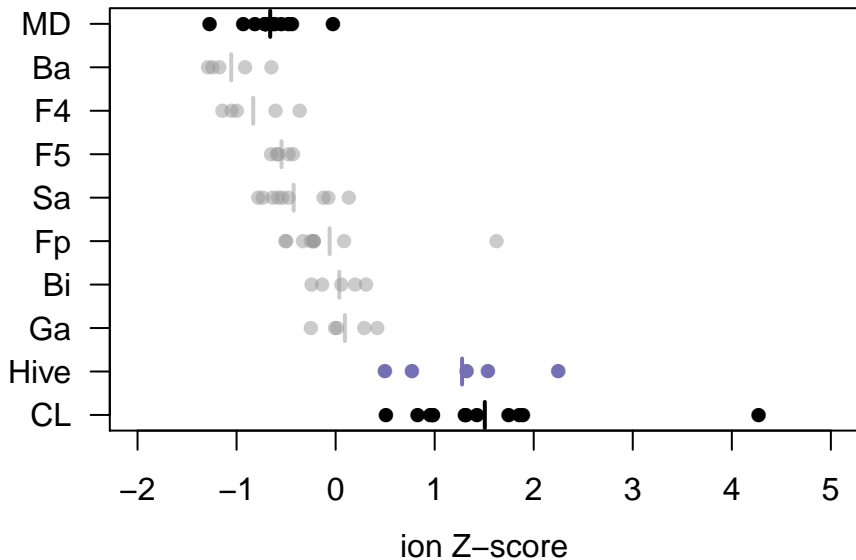

Supplement: S8 Data — (ZIP) [file pbio.2003467.s008.zip › Z-score_plots/261 microbial product cis-2-Carboxycyclohexyl-acetic acid.pdf]

# 10-Oxodecanoate\*

# 262 185.118 microbial product

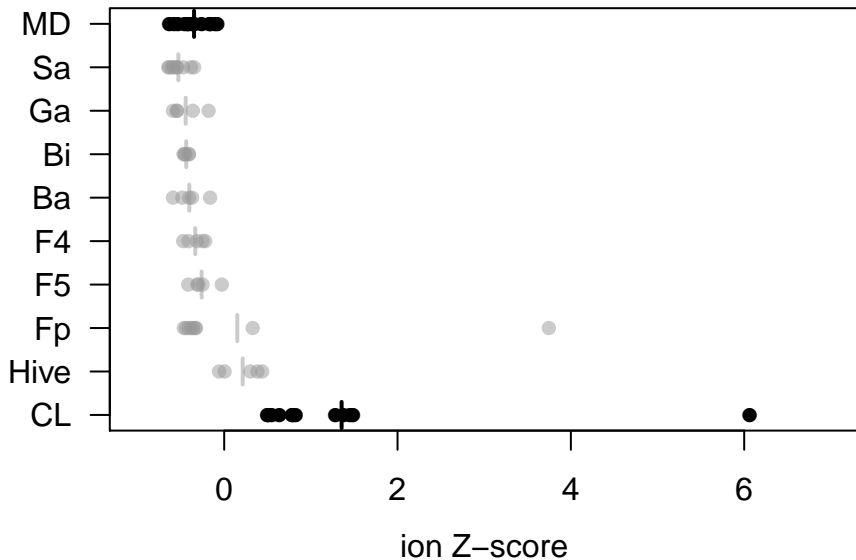

Supplement: S8 Data — (ZIP) [file pbio.2003467.s008.zip › Z-score_plots/262 microbial product 10-Oxodecanoate.pdf]

**6-Acetamido-3-oxohexanoate\***  
**# 264 186.077 microbial substrate**

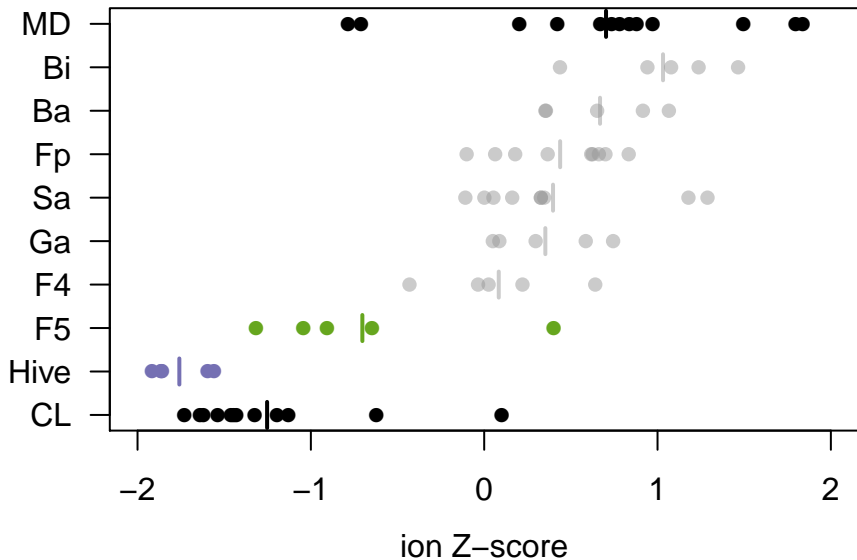

Supplement: S8 Data — (ZIP) [file pbio.2003467.s008.zip › Z-score_plots/264 microbial substrate 6-Acetamido-3-oxohexanoate.pdf]

**(Z)-But-1-ene-1,2,4-tricarboxylate\***  
**# 266 187.025 microbial product**

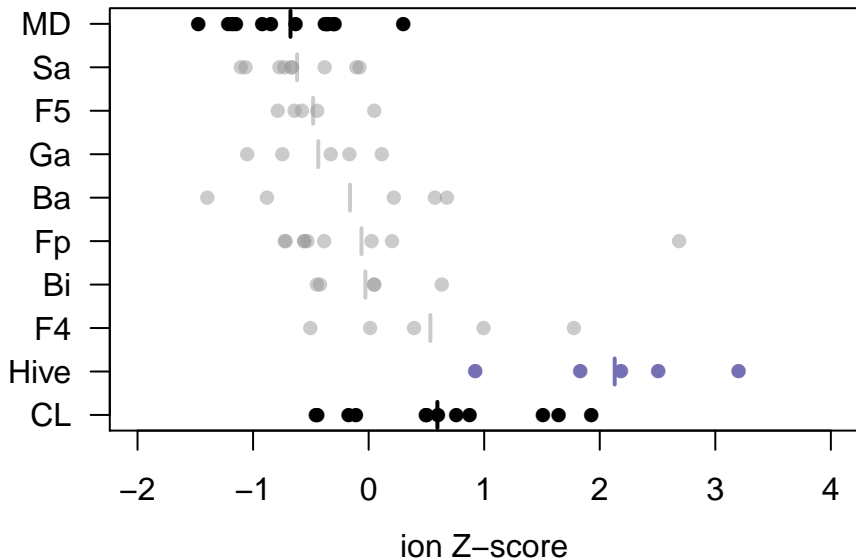

Supplement: S8 Data — (ZIP) [file pbio.2003467.s008.zip › Z-score_plots/266 microbial product (Z)-But-1-ene-1,2,4-tricarboxylate.pdf]

**Azelaic acid\***  
**# 268 187.097 microbial product**

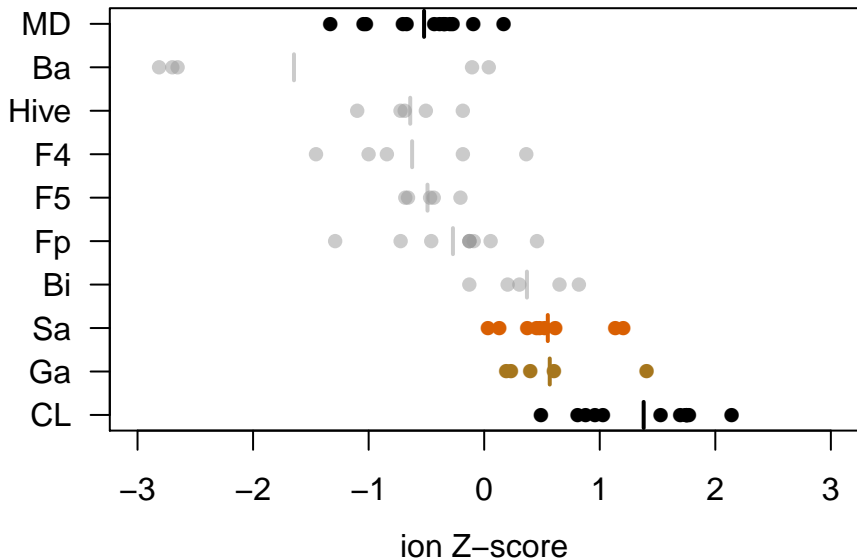

Supplement: S8 Data — (ZIP) [file pbio.2003467.s008.zip › Z-score_plots/268 microbial product Azelaic acid.pdf]

**Glycyl-leucine\***  
**# 269 187.108 microbial substrate**

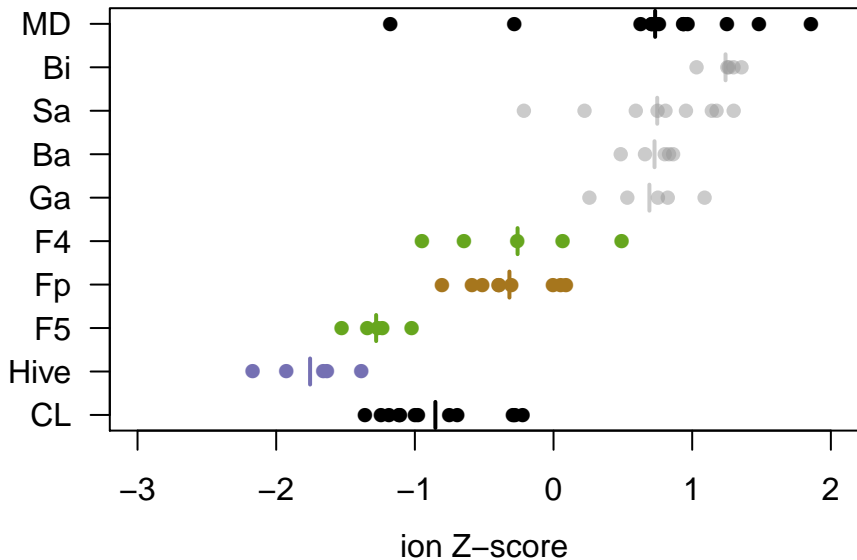

Supplement: S8 Data — (ZIP) [file pbio.2003467.s008.zip › Z-score_plots/269 microbial substrate Glycyl-leucine.pdf]

# 2-Pyridyl hydroxymethane sulfonic acid

# 271 188.001 microbial product

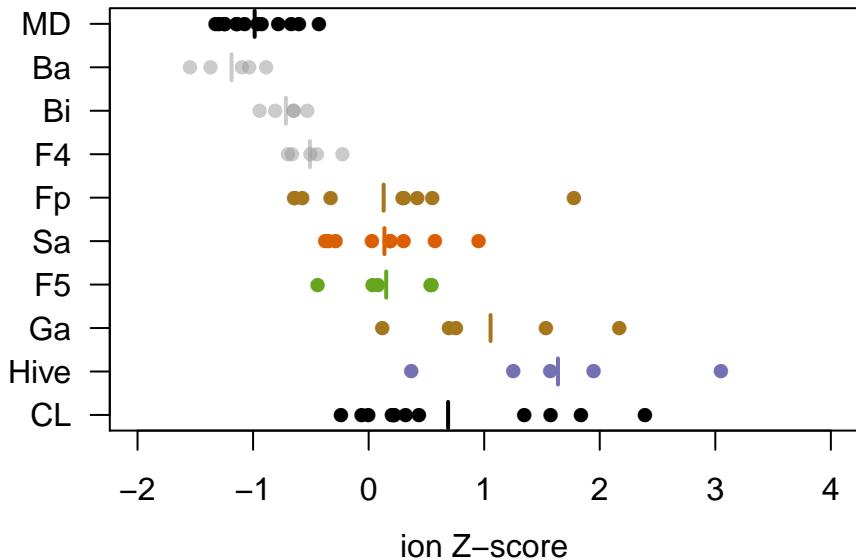

Supplement: S8 Data — (ZIP) [file pbio.2003467.s008.zip › Z-score_plots/271 microbial product 2-Pyridyl hydroxymethane sulfonic acid.pdf]

**4-Hydroxy-2-quinolinecarboxylic acid\***  
**# 272 188.035 microbial product**

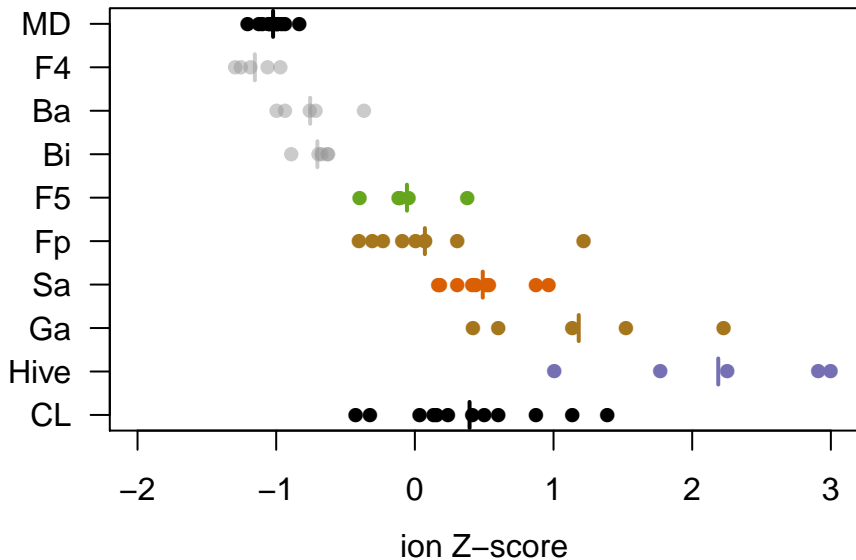

Supplement: S8 Data — (ZIP) [file pbio.2003467.s008.zip › Z-score_plots/272 microbial product 4-Hydroxy-2-quinolinecarboxylic acid.pdf]

**N-Acetyl-L-glutamate\***  
**# 273 188.056 microbial product**

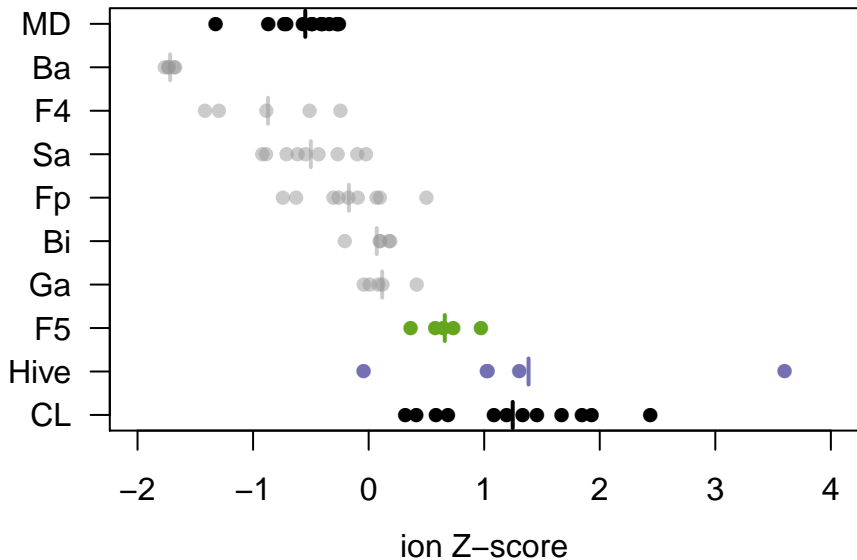

Supplement: S8 Data — (ZIP) [file pbio.2003467.s008.zip › Z-score_plots/273 microbial product N-Acetyl-L-glutamate.pdf]

# Castanospermine\*

# 275 188.092 microbial substrate

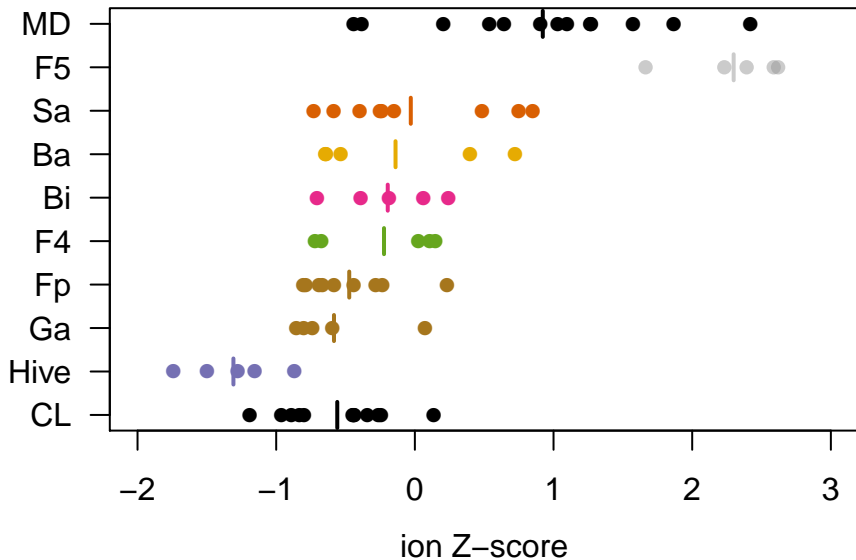

Supplement: S8 Data — (ZIP) [file pbio.2003467.s008.zip › Z-score_plots/275 microbial substrate Castanospermine.pdf]

EPTC\*

# 276 188.111 microbial substrate

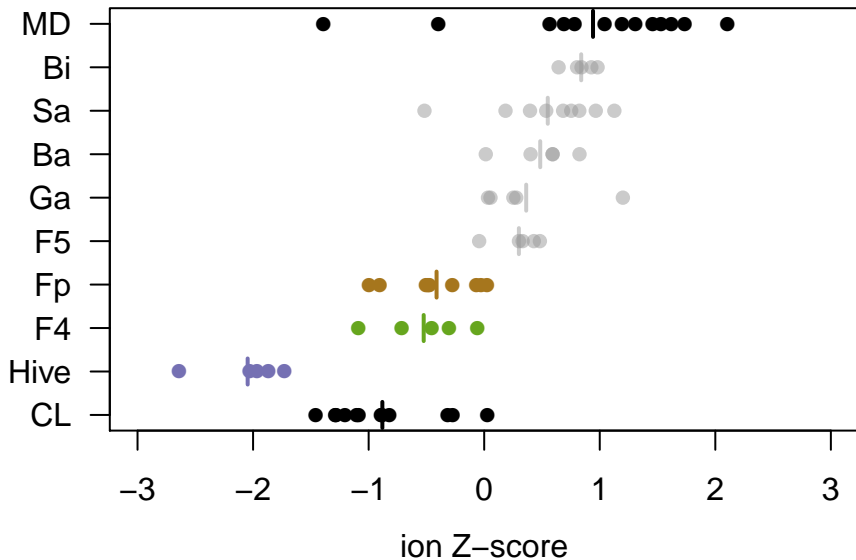

Supplement: S8 Data — (ZIP) [file pbio.2003467.s008.zip › Z-score_plots/276 microbial substrate EPTC.pdf]

**3-Sulfocatechol\***  
**# 277 188.985 microbial product**

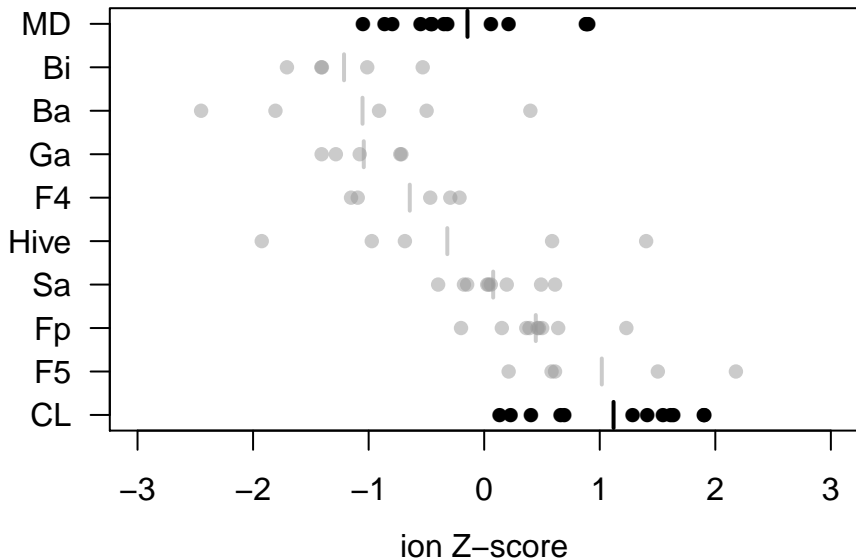

Supplement: S8 Data — (ZIP) [file pbio.2003467.s008.zip › Z-score_plots/277 microbial product 3-Sulfocatechol.pdf]

**2-Oxo-7-methylthioheptanoic acid**  
**# 279 189.059 microbial product**

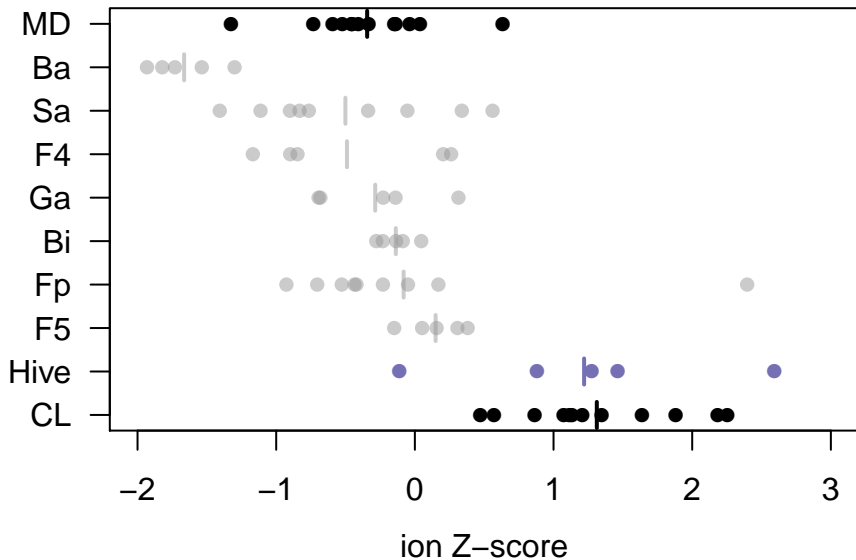

Supplement: S8 Data — (ZIP) [file pbio.2003467.s008.zip › Z-score_plots/279 microbial product 2-Oxo-7-methylthioheptanoic acid.pdf]

# 5-Hydroxyindoleacetate\*

# 282 190.051 microbial substrate

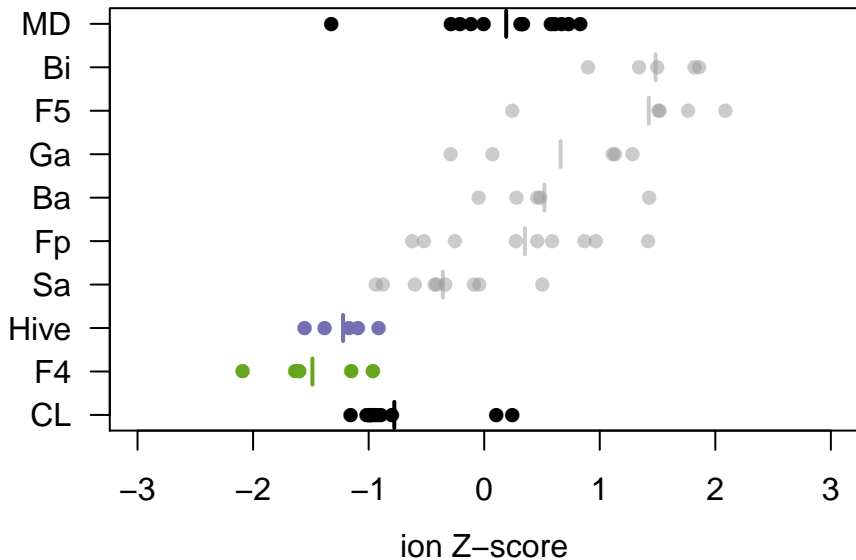

Supplement: S8 Data — (ZIP) [file pbio.2003467.s008.zip › Z-score_plots/282 microbial substrate 5-Hydroxyindoleacetate.pdf]

**Citrate\***

**# 285 191.019 microbial substrate**

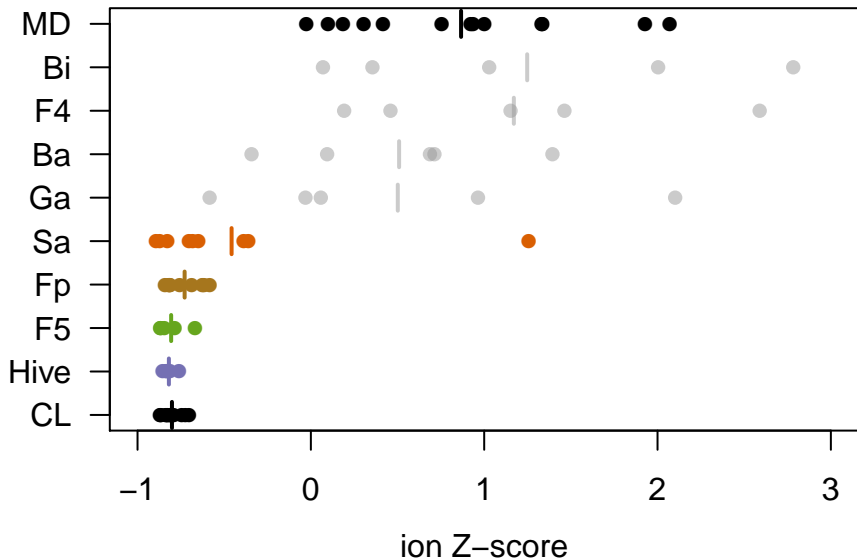

Supplement: S8 Data — (ZIP) [file pbio.2003467.s008.zip › Z-score_plots/285 microbial substrate Citrate.pdf]
