# Supplementary material for: Disentangling metabolic functions of bacteria in the honey bee gut
Source: PLoS Biol. 2017 Dec 12;15(12):e2003467. doi: 10.1371/journal.pbio.2003467 (PMC5726620; doi:10.1371/journal.pbio.2003467)
Supplement: S11 Data — (ZIP) [file pbio.2003467.s011.zip › expectedResults/exchanged G S 1 log2 microaerobic_one panel.pdf]

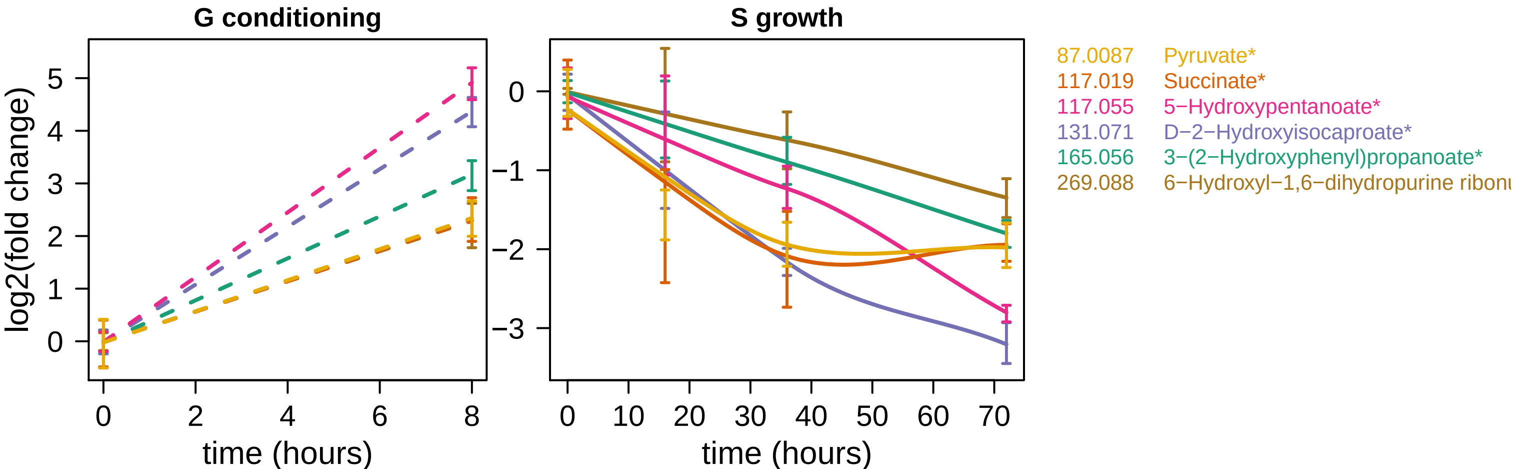

G conditioning
S growth
log2(fold change)
time (hours)
time (hours)
87.0087 Pyruvate*
117.019 Succinate*
117.055 5−Hydroxypentanoate*
131.071 D−2−Hydroxyisocaproate*
165.056 3−(2−Hydroxyphenyl)propanoate*
269.088 6−Hydroxyl−1,6−dihydropurine ribonu
