# Supplementary material for: Disentangling metabolic functions of bacteria in the honey bee gut
Source: PLoS Biol. 2017 Dec 12;15(12):e2003467. doi: 10.1371/journal.pbio.2003467 (PMC5726620; doi:10.1371/journal.pbio.2003467)
Supplement: S11 Data — (ZIP) [file pbio.2003467.s011.zip › expectedResults/pca four types.pdf]

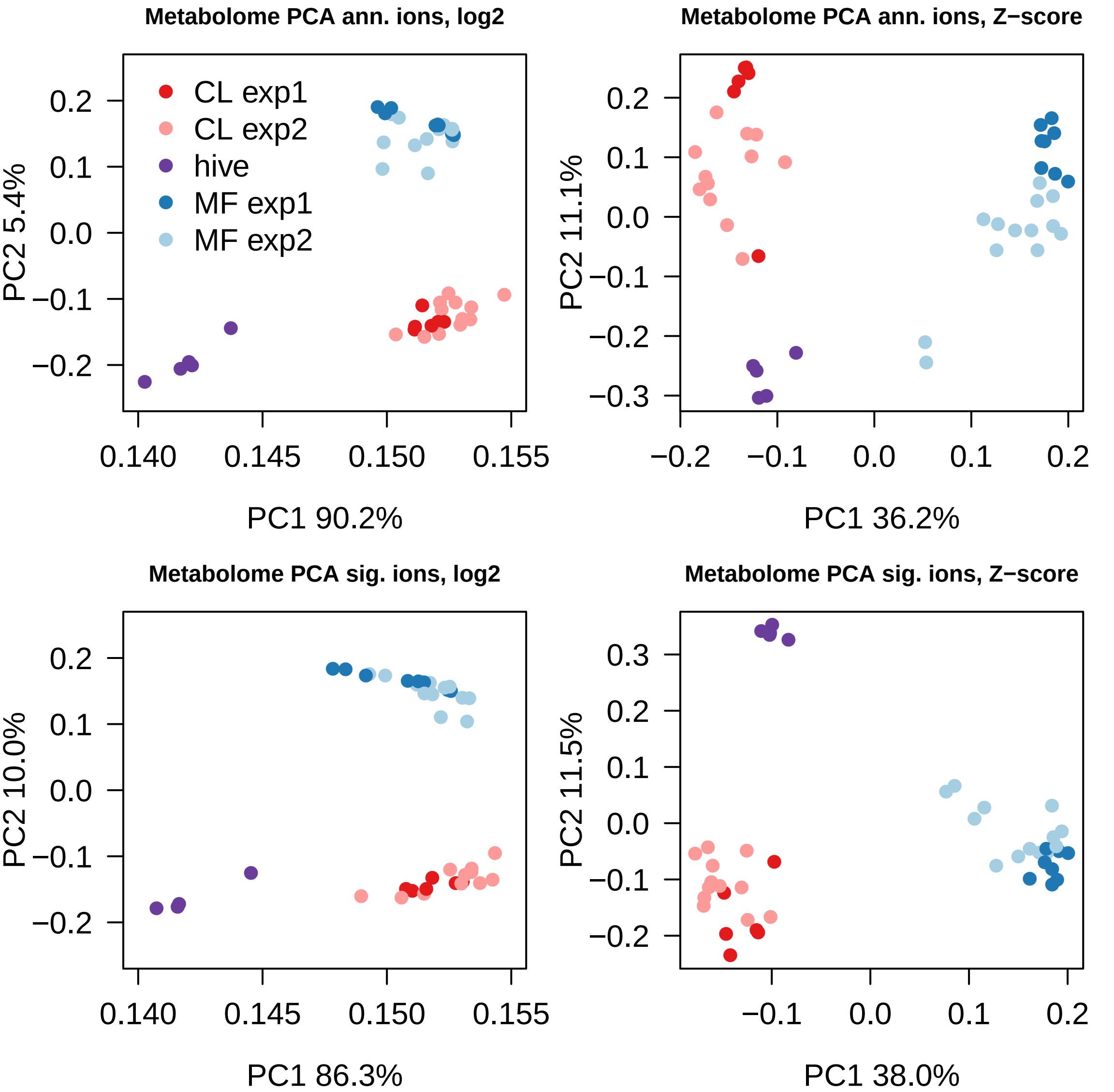

Metabolome PCA ann. ions, log2
CL exp1
CL exp2
hive
MF exp1
MF exp2
PC2 5.4%
PC1 90.2%
0.2
0.1
0.0
−0.1
−0.2
0.140
0.145
0.150
0.155
Metabolome PCA ann. ions, Z−score
PC2 11.1%
PC1 36.2%
0.2
0.1
0.0
−0.1
−0.2
−0.3
−0.2
−0.1
0.0
0.1
0.2
Metabolome PCA sig. ions, log2
PC2 10.0%
PC1 86.3%
0.2
0.1
0.0
−0.1
−0.2
0.140
0.145
0.150
0.155
Metabolome PCA sig. ions, Z−score
PC2 11.5%
PC1 38.0%
0.3
0.2
0.1
0.0
−0.1
−0.2
−0.1
0.0
0.1
0.2
