# Supplementary figures and images for: Disentangling metabolic functions of bacteria in the honey bee gut (part 3 of 3)
Source: PLoS Biol. 2017 Dec 12;15(12):e2003467. doi: 10.1371/journal.pbio.2003467 (PMC5726620; doi:10.1371/journal.pbio.2003467)

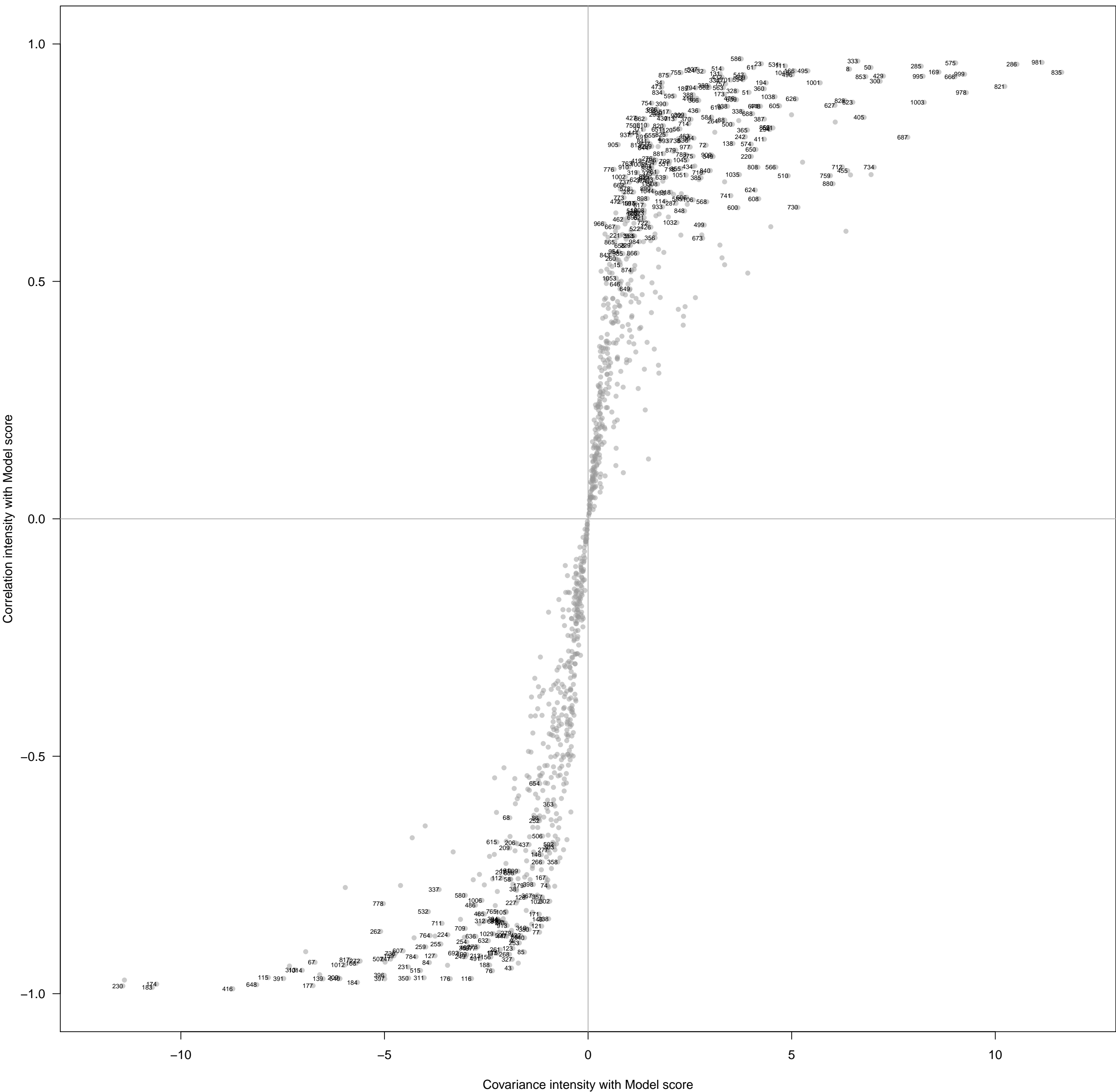

Supplement: S11 Data — (ZIP) [file pbio.2003467.s011.zip › expectedResults/oplsda gut plot ion names.pdf]

LC-MS/MS log<sub>2</sub>(FC)

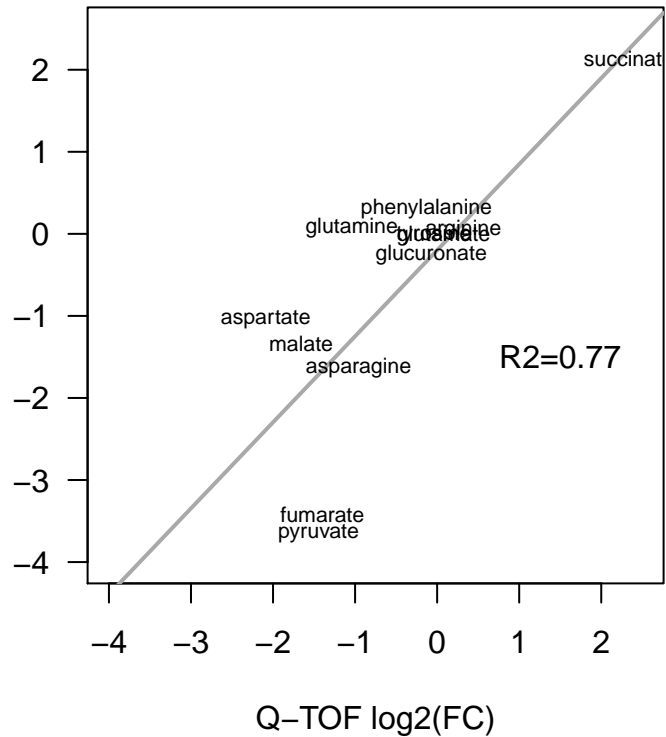

LC-MS/MS log<sub>2</sub>(FC)

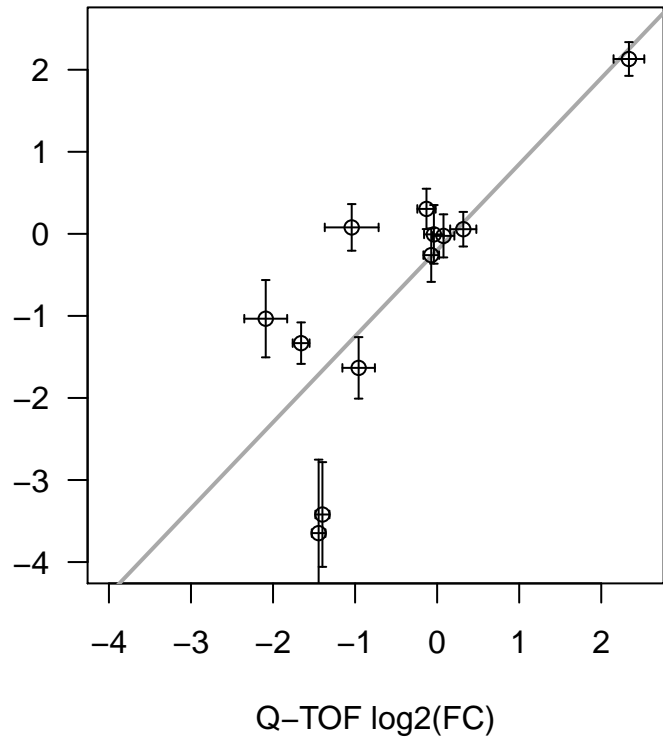

Q-TOF log<sub>2</sub>(FC)

Supplement: S11 Data — (ZIP) [file pbio.2003467.s011.zip › expectedResults/overlap untargeted and targeted v2.pdf]

- 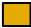 Alpha
- 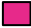 Bifido
- 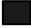 CL
- 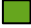 Firm
- 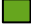 Firm
- 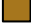 Gamma
- 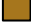 Gamma
- 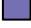 hive
- 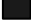 MF
- 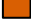 Beta-proteo

Supplement: S11 Data — (ZIP) [file pbio.2003467.s011.zip › expectedResults/phyla colors legend.pdf]

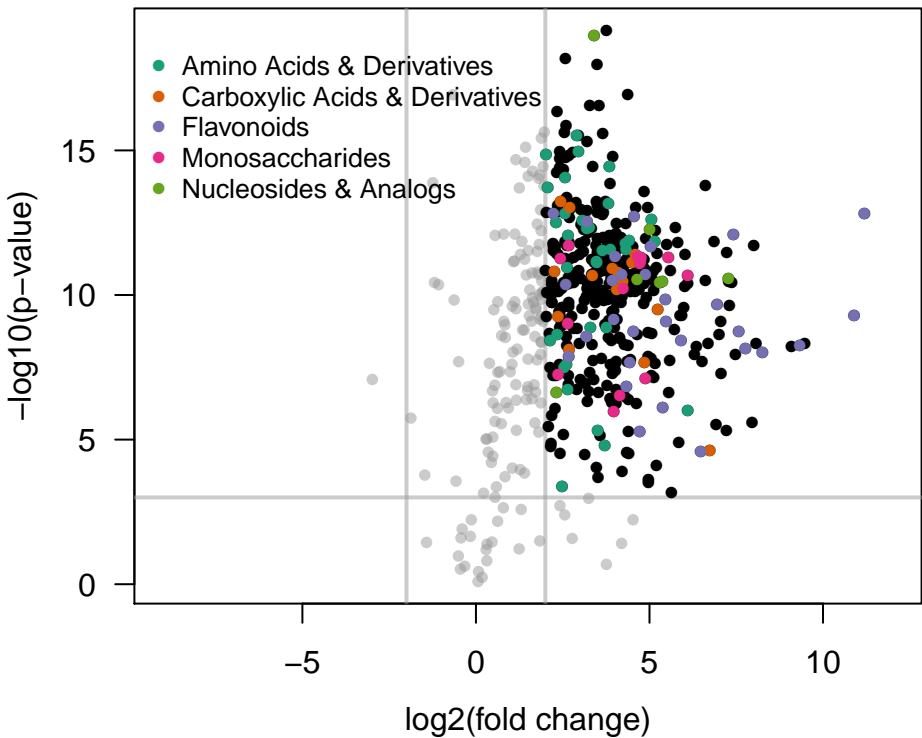

Supplement: S11 Data — (ZIP) [file pbio.2003467.s011.zip › expectedResults/pollen ions volcano seasonal v2.pdf]

log<sub>2</sub>(FC) CL:MF

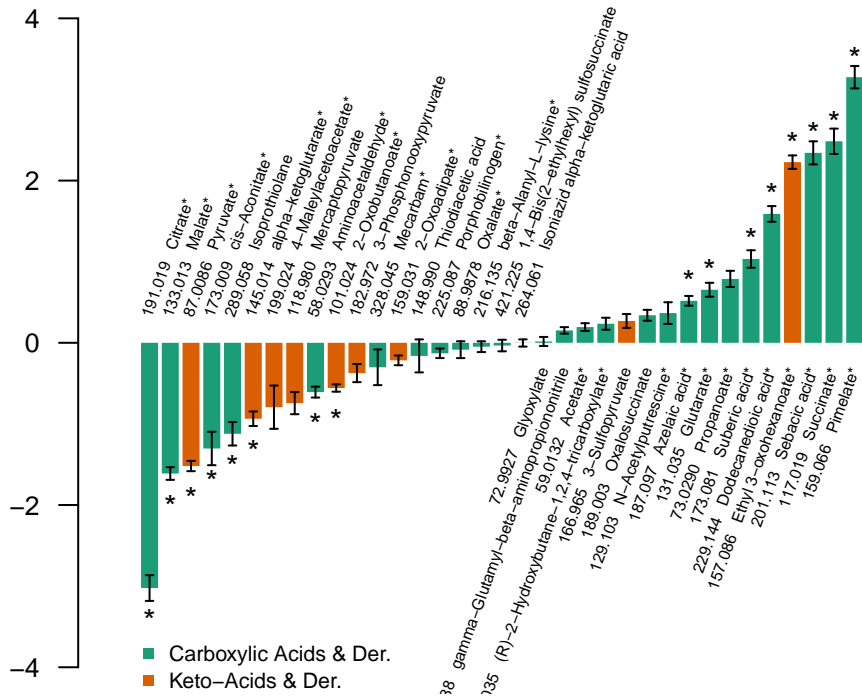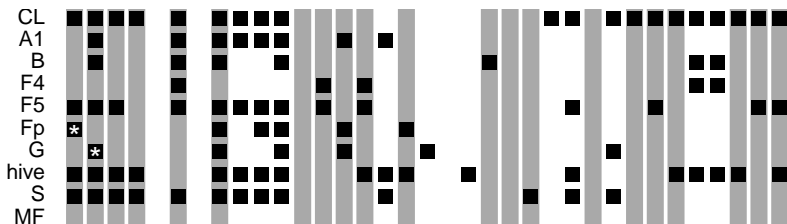

Supplement: S11 Data — (ZIP) [file pbio.2003467.s011.zip › expectedResults/supplement_v3_acids.pdf]

log<sub>2</sub>(FC) CL:MF4  
2  
0  
-2  
-4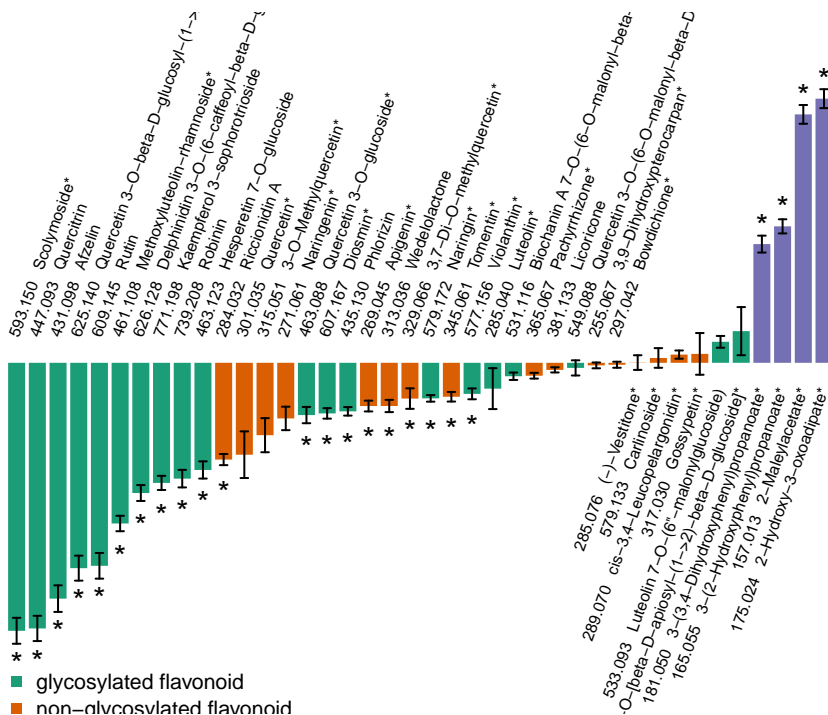CL  
A1  
B  
F4  
F5  
Fp  
G  
hive  
S  
MF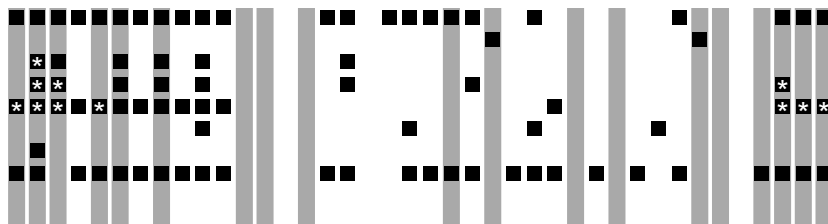

Supplement: S11 Data — (ZIP) [file pbio.2003467.s011.zip › expectedResults/supplement_v3_flavonoids.pdf]

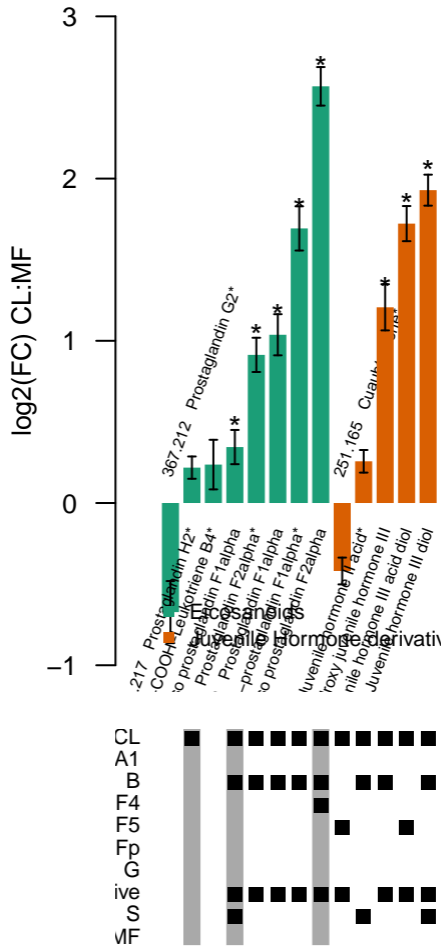

Supplement: S11 Data — (ZIP) [file pbio.2003467.s011.zip › expectedResults/supplement_v3_host compounds.pdf]

log<sub>2</sub>(FC) CL:MF

0  
-1  
-2  
-3  
-4

hydroxy  
phenol

CL  
A1  
B  
F4  
F5  
Fp  
G  
ve  
S  
MF

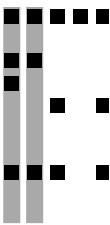

Supplement: S11 Data — (ZIP) [file pbio.2003467.s011.zip › expectedResults/supplement_v3_pollen.pdf]

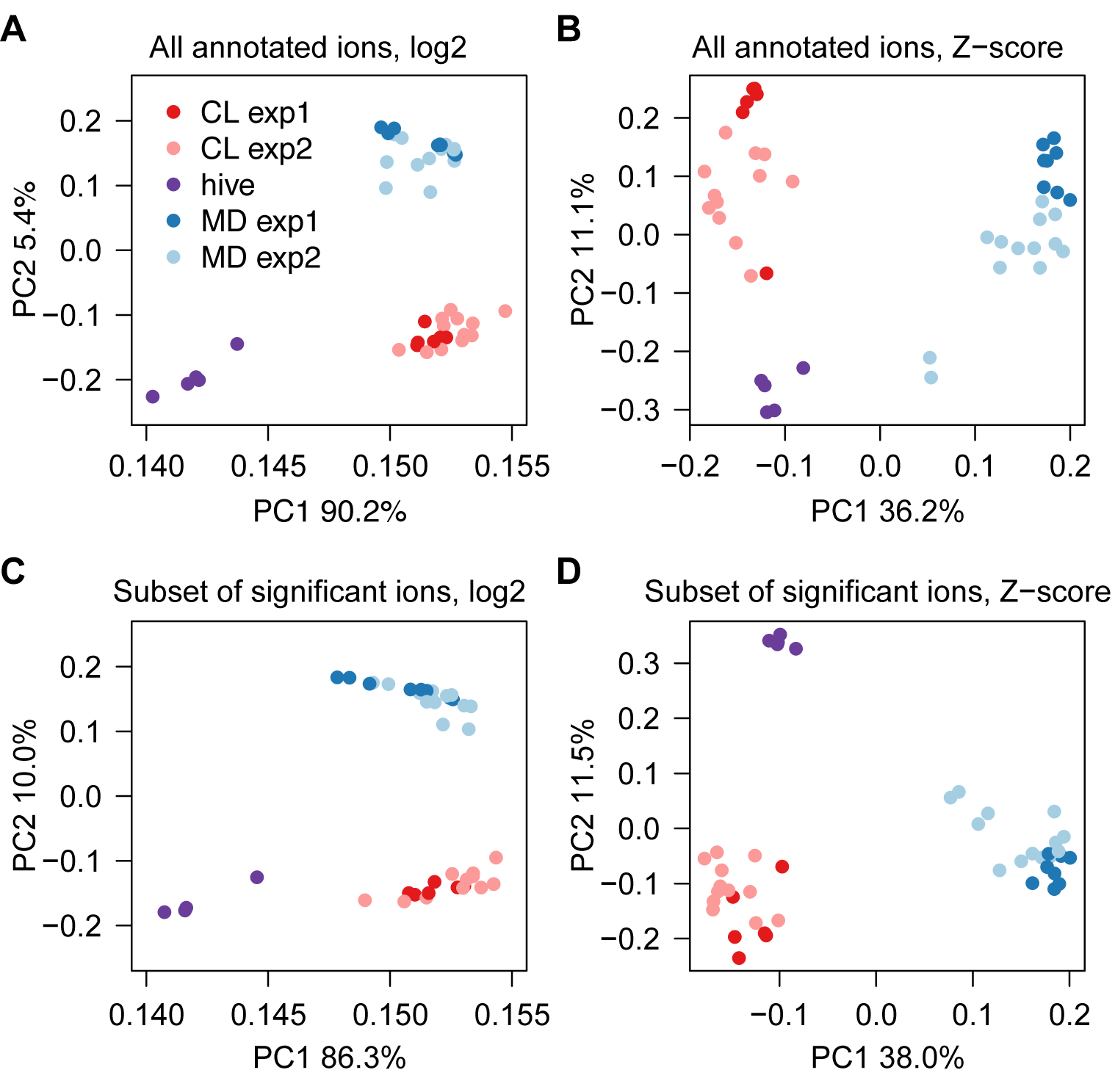

Supplement: S1 Fig — (A) Principal component analysis (PCA) based on log2-transformed ion intensities of all 1,079 annotated ions. (B) PCA based on Z-score normalized ion intensities of all 1,079 annotated ions. (C) PCA based on log2-transformed ion intensities of the 372 ions that show significant changes between CL and MD bees. (C) PCA based on Z-score normalized ion intensities of the 372 ions that show significant changes between CL and MD bees. CL and MD bees come from two independent experiments (see S5 Text and S5 Table) conducted at two different time points of the same year. Hive bees utilized for metabolomics analysis came from the second experiment. We used log2-transformed and Z-score normalized ion intensities to show the large effect of high-intensity ions on the percentage of variance explained (PC1). We conducted the analysis on all ions and the subset of significant ion changes to illustrate that the two seeming outlying samples in B are due to ions that are not of relevance for our subsequent analysis. PC1, principal component 1; PC2, principal component 2. The numerical data can be found in S1 Data. (TIF) [file pbio.2003467.s012.tif]

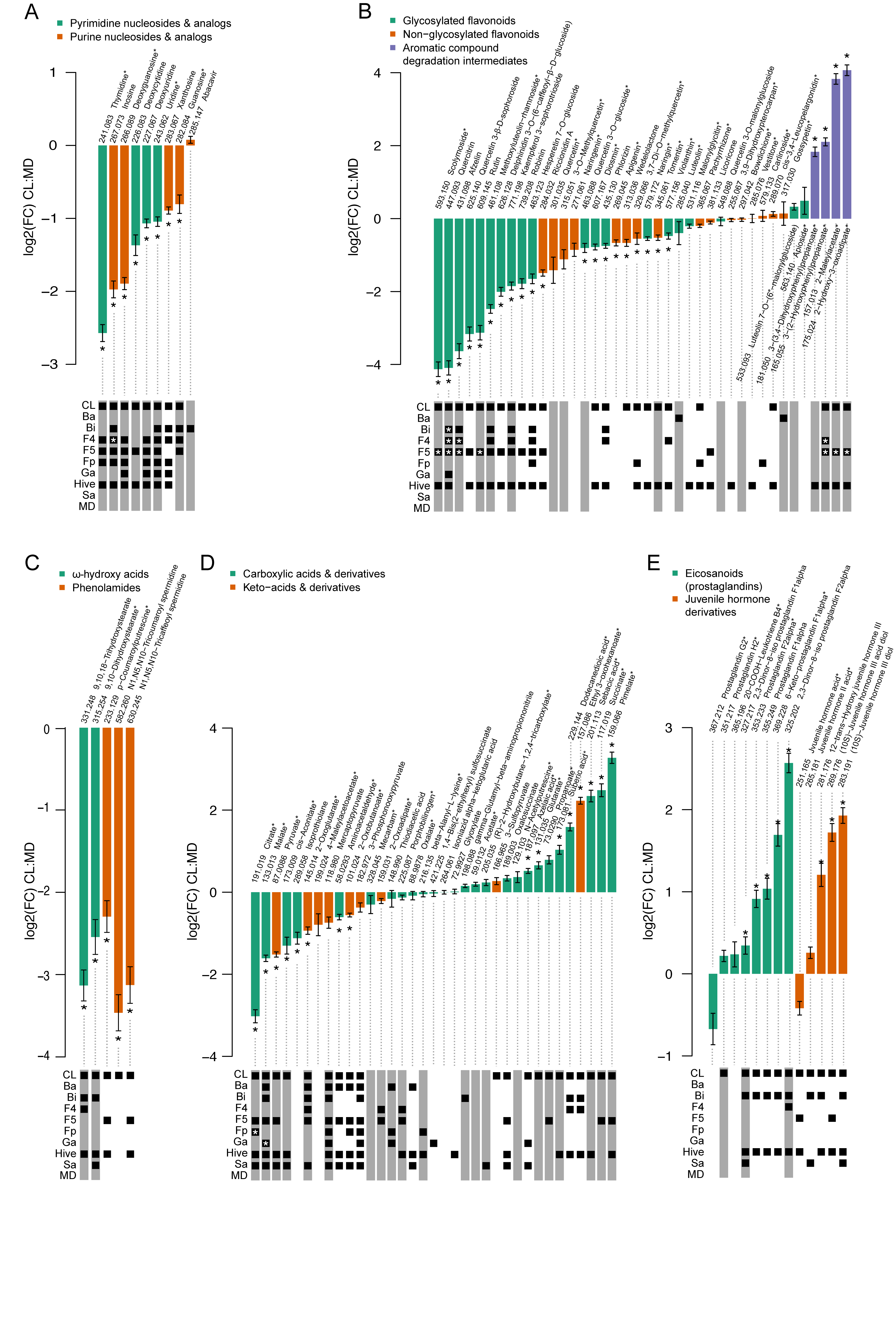

Supplement: S2 Fig — (A) Pyrimidine nucleosides and analogues (green) and purine nucleosides and analogues (orange). (B) Glycosylated flavonoids (green), non-glycosylated flavonoids (orange), and intermediates of aromatic compound degradation pathways (purple). (C) Pollen wall components with ω-hydroxy acids (green) and phenolamides (orange). (D) Carboxylic acids and derivatives (green) and keto acids and derivatives (orange). (E) Host metabolites with eicosanoids (green) and juvenile hormone derivatives (orange). For all five panels, the log2(fold change) values between CL and MD bees are plotted as bar graphs for each ion. The first annotation of each ion is provided. An asterisk indicates ambiguous annotations. Asterisks below/above bars indicate significant fold changes (based on Welch’s t test on two independent experiments, both experiments Benjamini and Hochberg adjusted [BH adj.] P < 0.01). ANOVA results for the comparison of each treatment group versus MD bees are depicted below each bar graph. Significant metabolite changes in vivo are indicated by black squares. Grey shading indicates that the metabolite was annotated in the in vitro dataset, and an asterisk in the black square indicates that the same metabolic change was recapitulated in vitro. Note that only metabolites with log2(fold changes) of ≥|1| and BH adj. P < 0.01 (Welch’s t test) from the in vitro experiments were considered. Ba, B. apis mono-colonized; Bi, B. asteroides mono-colonized; CL, colonized with the reconstituted microbiota; F4, Firm-4 mono-colonized; F5, Firm-5 mono-colonized; Fp, F. perrara mono-colonized; Ga, G. apicola mono-colonized; Hive, hive bees; MD, microbiota-depleted; Sa, S. alvi mono-colonized. The numerical data of the bar graphs can be extracted from S2A Data. (TIF) [file pbio.2003467.s013.tif]

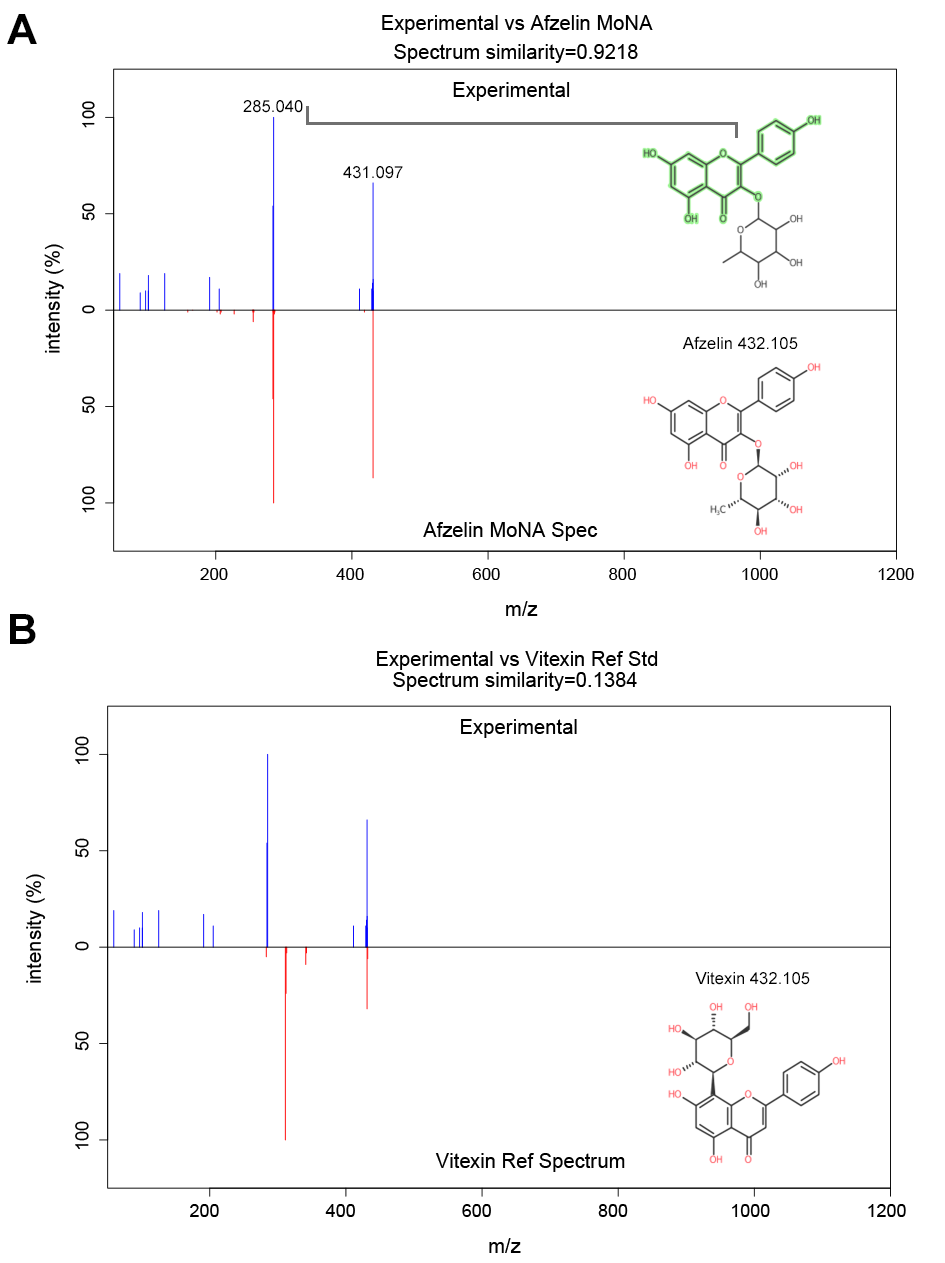

Supplement: S3 Fig — (A) displays fragmentation spectrum for ion #821 on top and a reference spectrum for afzelin below (CCMSLIB00000845703, MassBank of North America). (B) displays fragmentation spectrum for ion #821 on top and the reference spectrum obtained in house for the pure vitexin standard. A high spectral similarity score with afzelin standard confirms the annotation of ion #821 as afzelin. For masses of the fragments, raw ion spectra, and alternative annotations, see S4 Data. (TIF) [file pbio.2003467.s014.tif]

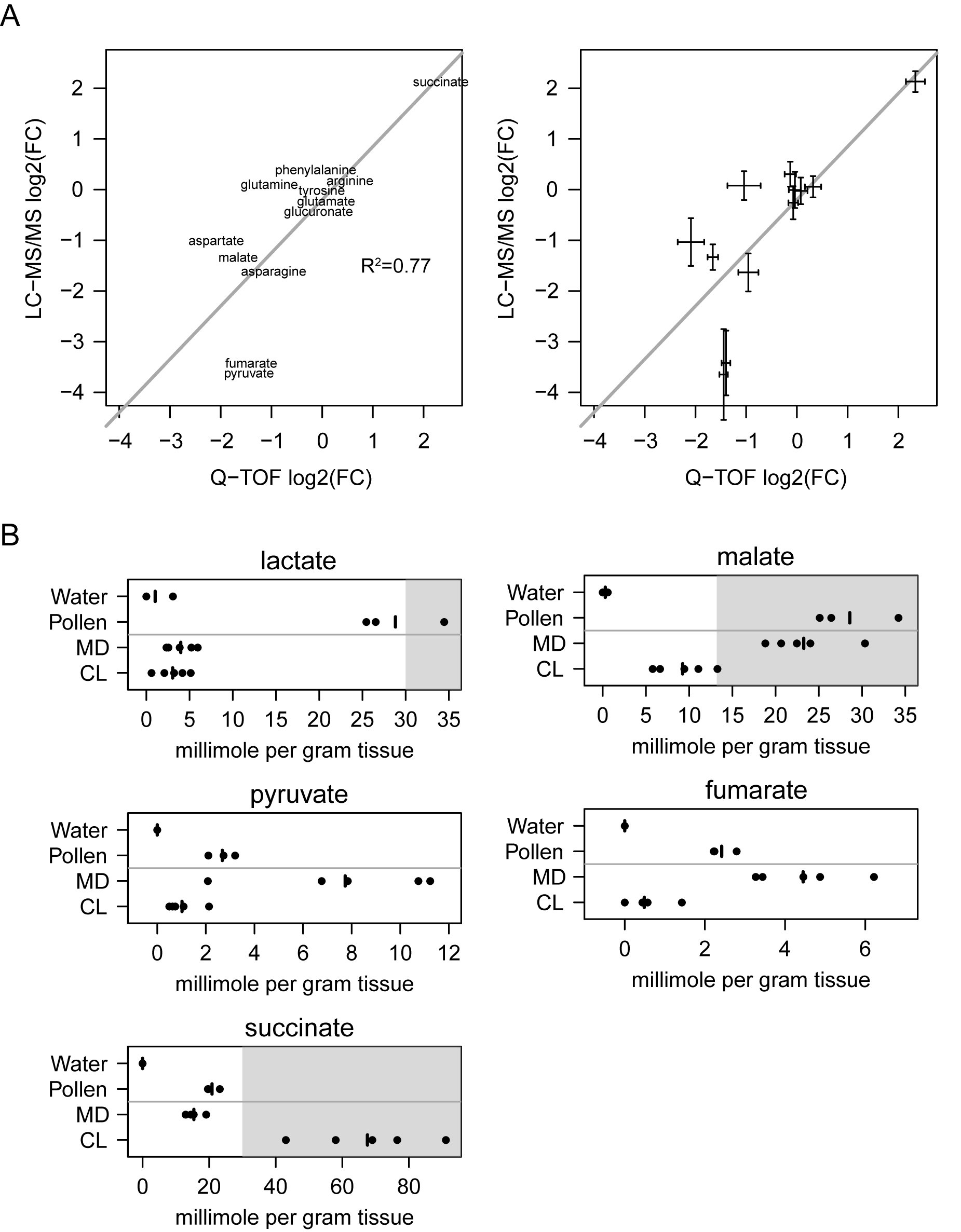

Supplement: S4 Fig — (A) Correlation in log2(fold change) of metabolites or ions annotated both in LC-MS/MS and quadrupole-time of flight (Q-TOF). Identical data are plotted in both panels, with the left panel presenting the names of the metabolites (or the most likely annotation of the corresponding ion) and the right panel presenting the standard error of the log2(fold change) for both methods. (B) Concentrations of selected metabolites in pollen extracts and gut samples. Grey zones indicate extrapolation from the standard curve, i.e., relative changes. The line indicates the arithmetic mean concentration. The numerical data of untargeted metabolomics can be extracted from S2A Data. The numerical data of targeted metabolomics can be found in S6 Data. (TIF) [file pbio.2003467.s015.tif]

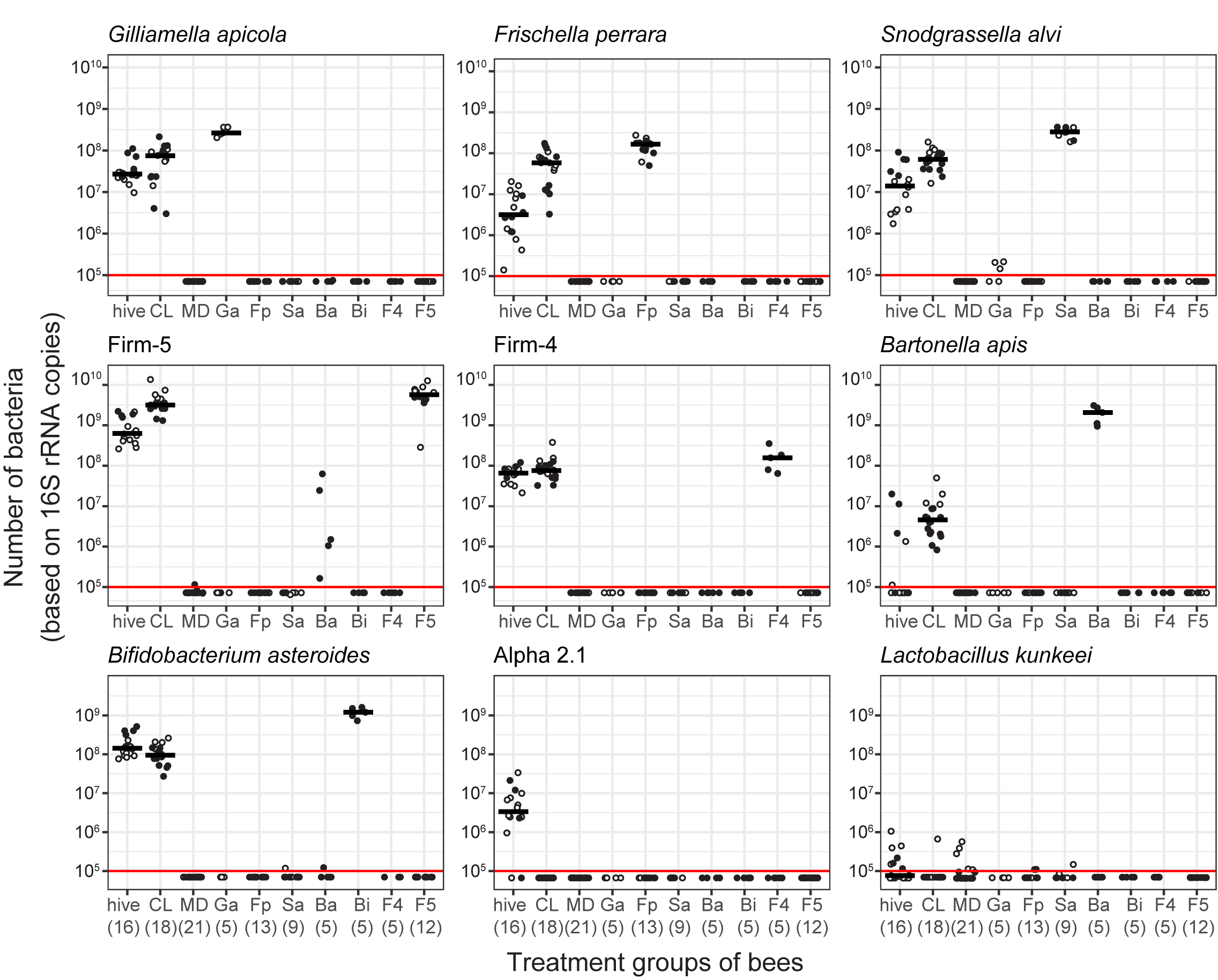

Supplement: S5 Fig — Each plot presents the colonization levels of a specific bee gut bacterial associate as based on quantitative PCR (qPCR) with species-specific 16S rRNA primers (S2 Table). In addition to the seven major community members used in our colonization experiments, we also screened for Alpha-2.1 and L. kunkeei, as these two species can constitute common contaminants in gnotobiotic bees. The results are shown according to the 10 treatment groups on the x-axis, i.e., hive bees, CL bees, MD bees (same as in Fig 2), and mono-colonized bees (Ga, Fp, Sa, Ba, Bi, F4, and F5). Open and filled circles indicate samples coming from experiment 1 and experiment 2, respectively. The red line corresponds to 105 bacterial cells per gut, which we consider as our threshold of presence of the given bacterial species. All values below this limit of the qPCR assay are plotted below this line. A few samples had slightly higher values than 105 for S. alvi, Firm-5, B. asteroides, and L. kunkeei. They were still included in our analysis to increase statistical power. Based on the metabolomics analysis and the recapitulation of our findings in vitro, we feel confident that these possible contaminants had a negligible effect on our results. Median values are shown as black lines for hive bee samples, CL bee samples, and mono-colonization samples corresponding to the primer pair used. Ba, B. apis mono-colonized; Bi, B. asteroids mono-colonized; CL, colonized with the reconstituted microbiota; F4, Firm-4 mono-colonized; F5, Firm-5 mono-colonized; Fp, F. perrara mono-colonized; Ga, G. apicola mono-colonized; Hive, hive bees; MD, microbiota-depleted; Sa, S. alvi mono-colonized. The numerical data can be found in S1 Data. (TIF) [file pbio.2003467.s016.tif]

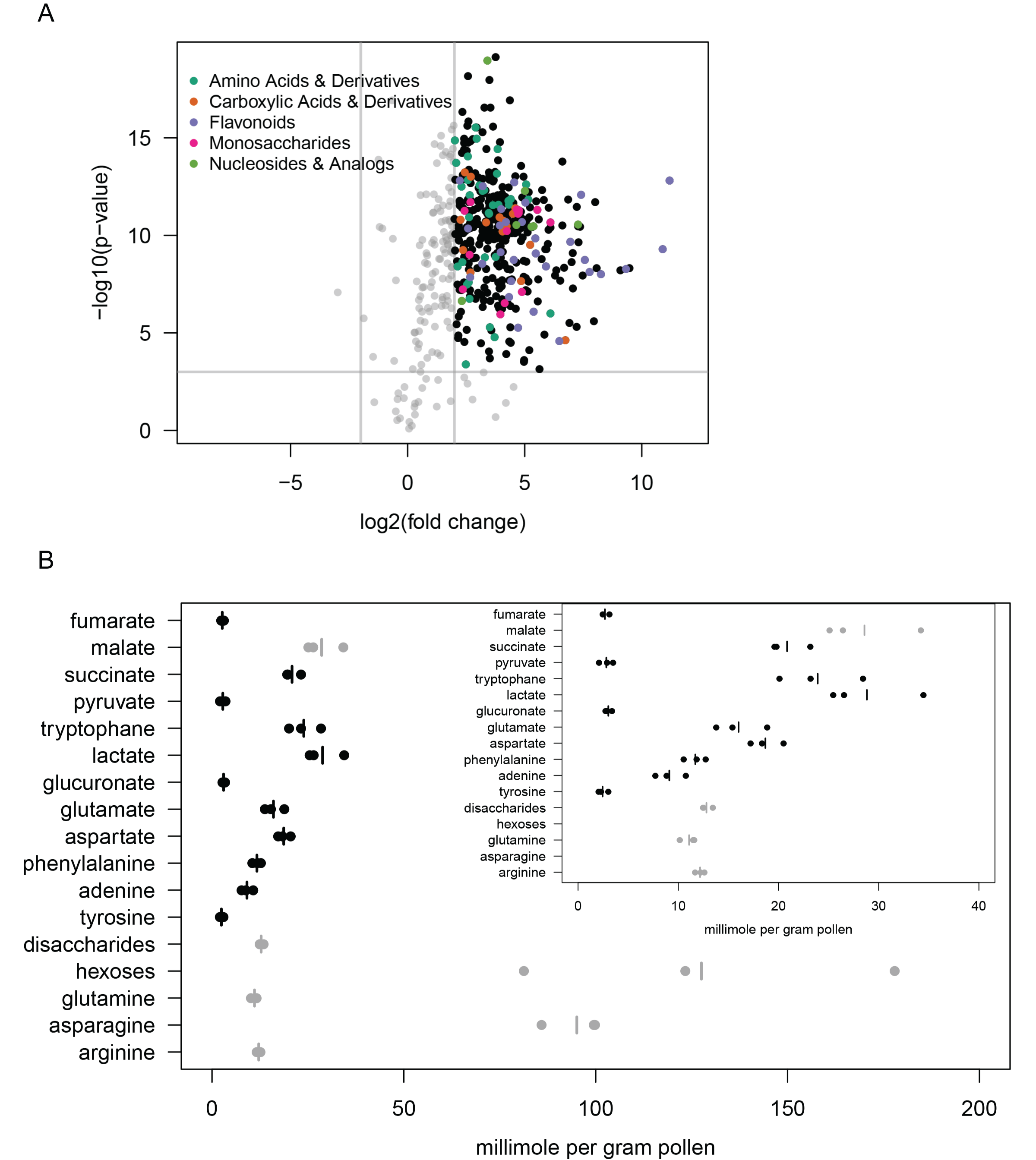

Supplement: S6 Fig — (A) A volcano plot represents ions enriched in pollen (black or colored dots). Colored dots belong to enriched categories based on their main annotation (significance levels mentioned in text), except for “nucleosides and analogs,” which consists of the pooled groups “purine nucleosides and analogues,” 4/5, one-sided Fishers exact test P = 0.066, and “pyrimidine nucleosides and analogues,” 3/4, P = 0.148. (B) Concentrations of metabolites detected in pollen extract using targeted LC-MS/MS. Three replicates are shown as dots, with a line indicating the arithmetic mean. Metabolites that could be quantified using interpolations from standard curves are indicated in black. Metabolites for which standard curves did not meet the linearity criteria or for which values had to be extrapolated are plotted in grey. The inset shows identical data with a rescaled x-axis for more fine-grained inspection. The numerical values of the volcano plot and the targeted metabolomics can be found in S2A Data and S6 Data, respectively. (TIF) [file pbio.2003467.s017.tif]

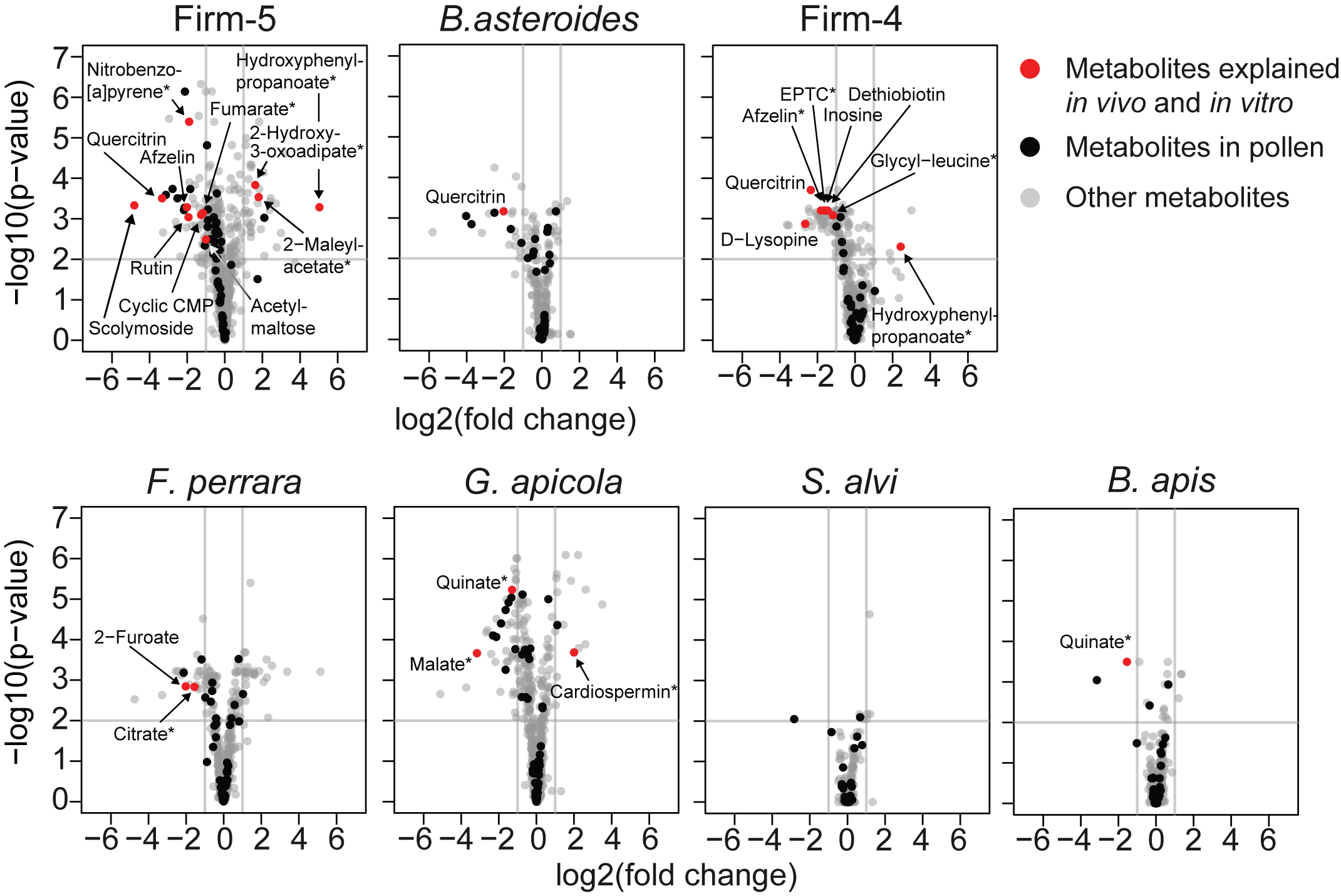

Supplement: S7 Fig — Volcano plots of significance (Welch’s t test Benjamini and Hochberg adjusted [BH adj.] P value) versus log2(fold change) showing metabolic changes in pollen-conditioned medium at time point 16 h relative to 0 h. Ions identified as likely pollen-derived are highlighted in black. Ions highlighted in red correspond to metabolites that showed robust changes between colonized (CL) and microbiota-depleted (MD) bees in vivo and are explained in vivo and in vitro by the same community member. Annotations of these ions are given and summarized in S3 Table. Other annotated ions are plotted in grey. The numerical values can be extracted from S9 Data. (TIF) [file pbio.2003467.s018.tif]

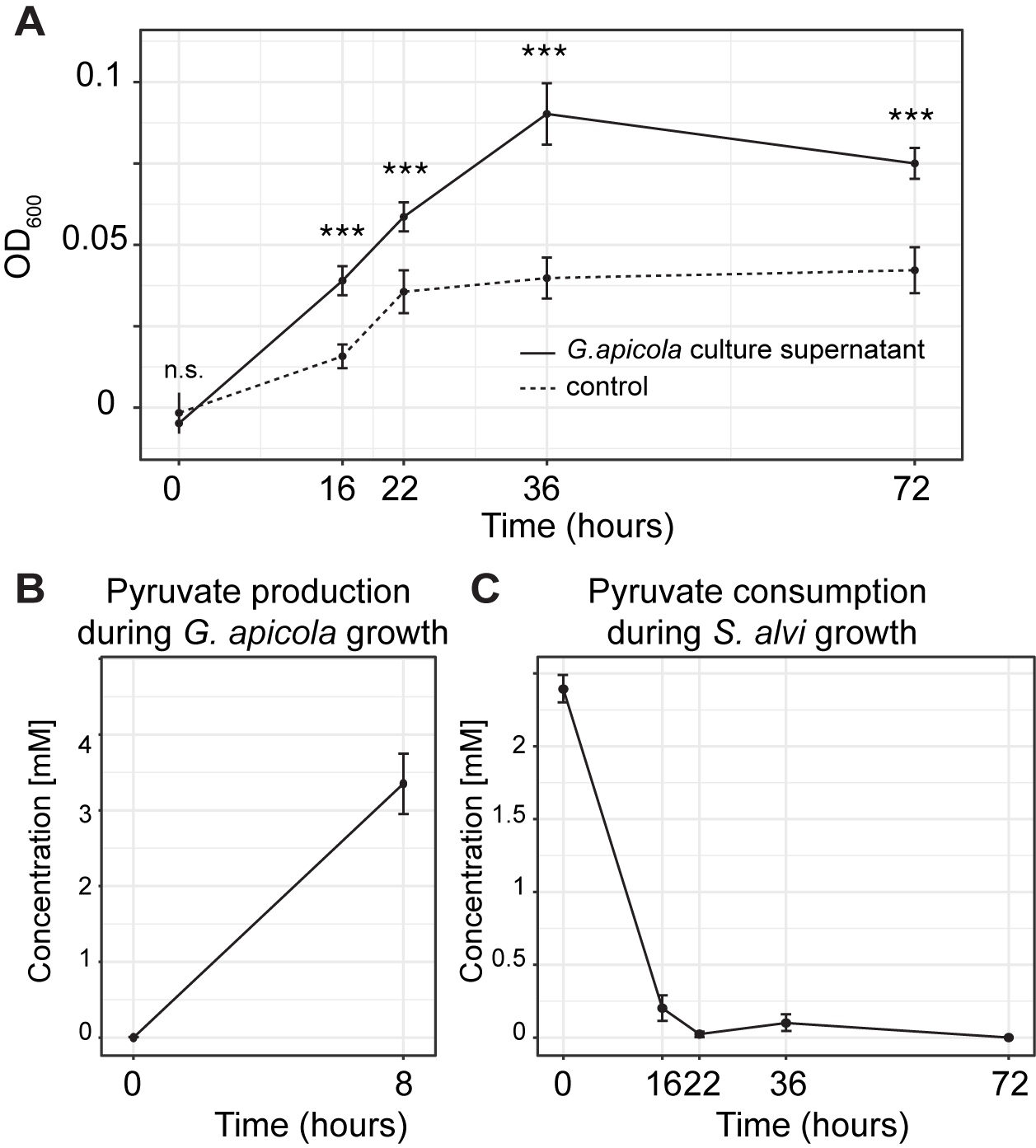

Supplement: S8 Fig — (A) Independent cross-feeding experiment that was carried out in the same way as the experiment presented in Fig 6B. S. alvi was grown in pollen-conditioned medium in the presence (black line) or absence (dashed line) of G. apicola culture supernatant (50%, v/v). (B) Quantification of pyruvate in the pollen extract-based culture medium before and after growth of G. apicola. (C) Quantification of pyruvate in the G. apicola-conditioned culture medium during growth of S. alvi at different time points. Data from panel B and C come from the cross-feeding experiment presented in panel A. The numerical values can be found in S1 Data. (TIF) [file pbio.2003467.s019.tif]

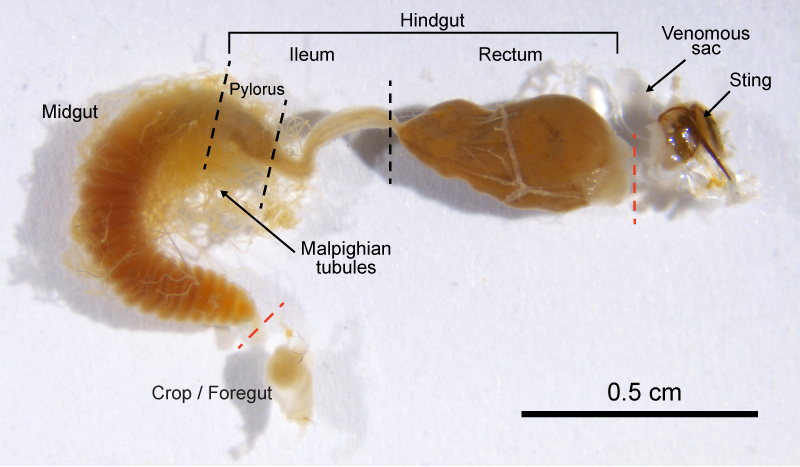

Supplement: S9 Fig — Different gut regions and connected tissues are indicated. Dashed lines depict borders of different gut regions. The red dashed lines indicate the part of the gut taken for metabolomics analysis and DNA extraction/quantitative PCR (qPCR) analysis. (TIF) [file pbio.2003467.s020.tif]
