# Supplementary material for: Disentangling metabolic functions of bacteria in the honey bee gut
Source: PLoS Biol. 2017 Dec 12;15(12):e2003467. doi: 10.1371/journal.pbio.2003467 (PMC5726620; doi:10.1371/journal.pbio.2003467)
Supplement: S1 Table — (DOCX) [file pbio.2003467.s021.docx]

**S1 Table.** Bacterial strains used in this study.

| **Bacterial strain** | **Treatment group** | **# 16S rRNA copies** | **Culturing condition** | **Strain source** | **Place of origin** |
| --- | --- | --- | --- | --- | --- |
| *Gilliamella apicola*  wkB1^T^ | Ga and CL | 4 | BHIA, 35°C,  microaerophilic | [1] | New Haven, USA |
| *Gilliamella apicola* ESL0169 | Ga and CL | 4 | BHIA, 35°C,  microaerophilic | This study | Lausanne, Switzerland |
| *Frischella perrara* PEB0191^T^ | Fp and CL | 4 | TYG, 34°C,  anaerobic | [2] | New Haven, USA |
| *Snodgrassella alvi*  wkB2^T^ | Sa and CL | 4 | TSA, 35°C,  microaerophilic | [1] | New Haven, USA |
| *Bartonella apis*  PEB0149 | Ba and CL | 2 | Blood agar, 35°C,  microaerophilic | [3] | New Haven, USA |
| *Bifidobacterium asteroides*  ESL0170 | Bi and CL | 2 | MRSA, 34°C, anaerobic | This study | Lausanne, Switzerland |
| *Lactobacillus* Firm-4 Hon2N^T^ | F4 and CL | 4 | MRSA, 34°C, anaerobic | [4] | Lund, Sweden |
| *Lactobacillus* Firm-5 ESL0183 (≙ Bma5N)* | F5 and CL | 4 | MRSA, 34°C, anaerobic | This study | Lausanne, Switzerland |
| *Lactobacillus* Firm-5 ESL0184 (≙ Hma8N)* | F5 and CL | 4 | MRSA, 34°C, anaerobic | This study | Lausanne, Switzerland |
| *Lactobacillus* Firm-5 ESL0185 (≙ Hma11N)* | F5 and CL | 4 | MRSA, 34°C, anaerobic | This study | Lausanne, Switzerland |
| *Lactobacillus* Firm-5 ESL0186 (≙ Biut2N)* | F5 and CL | 4 | MRSA, 34°C, anaerobic | This study | Lausanne, Switzerland |

*16S rRNA gene similarity of the strain is most similar to the strain in brackets [4]. The information about the number of 16S rRNA copies per genome was taken from JGI Genome database and from whole-genome analyses of corresponding strains.

**References:**

1. Kwong WK, Moran NA. Cultivation and characterization of the gut symbionts of honey bees and bumble bees: description of *Snodgrassella alvi* gen. nov., sp. nov., a member of the family *Neisseriaceae* of the *Betaproteobacteria*, and *Gilliamella apicola* gen. nov., sp. nov., a member of *Orbaceae* fam. nov., *Orbales* ord. nov., a sister taxon to the order “*Enterobacteriales*” of the *Gammaproteobacteria*. Int J Syst Evol Microbiol. 2013;63: 2008–2018. doi:10.1099/ijs.0.044875-0.

2. Engel P, Kwong WK, Moran NA. *Frischella perrara* gen. nov., sp. nov., a gammaproteobacterium isolated from the gut of the honeybee, *Apis mellifera*. Int J Syst Evol Microbiol. 2012. 63:3646-51. doi: 10.1099/ijs.0.049569-0.

3. Kešnerová L, Moritz R, Engel. *Bartonella apis* sp. nov., a honey bee gut symbiont of the class Alphaproteobacteria. Int J Syst Evol Microbiol. 2015. 66:414-21. doi: 10.1099/ijsem.0.000736.

4. Olofsson TC, Alsterfjord M, Nilson B, Butler E, Vasquez A. *Lactobacillus apinorum* sp. nov., *Lactobacillus mellifer* sp. nov., *Lactobacillus mellis* sp. nov., *Lactobacillus melliventris* sp. nov., *Lactobacillus kimbladii* sp. nov., *Lactobacillus helsingborgensis* sp. nov. and *Lactobacillus kullabergensis* sp. nov., isolated from the honey stomach of the honeybee *Apis mellifera*. Int J Syst Evol Microbiol. 2014. 64: 3109–3119 10.1099/ijs.0.059600-0.
