# Supplementary material for: Disentangling metabolic functions of bacteria in the honey bee gut
Source: PLoS Biol. 2017 Dec 12;15(12):e2003467. doi: 10.1371/journal.pbio.2003467 (PMC5726620; doi:10.1371/journal.pbio.2003467)
Supplement: S2 Table — (DOCX) [file pbio.2003467.s022.docx]

**S2 Table**. Primers used in this study and standard curve characteristics.

| Target (gene name and accession no.) | Sequence (5’ to 3’) | Tm (°C)^a^ | Amplicon size | Standard curve | | | Reference and notes |
| --- | --- | --- | --- | --- | --- | --- | --- |
|  |  |  |  | Efficiency, R^2^ | Slope, intercept | LOD Cq^b^  (# copies) |  |
| *Apis mellifera*  (*actin*, AB023025) | **F:** TGCCAACACTGTCCTTTCTG  **R:** AGAATTGACCCACCAATCCA | 58.4  56.4 | 156 bp | 1.896 =  89.6 %, 1.0 | -3.6,  37.579 | 33.726  (10) | [1] |
| *Gilliamella apicola*  (*16S rRNA*, JQ936674) | **F:** CTTTGTTGCCATCGGTTAGGCC  **R:** CCGCTTGCTCTCGCGAGG | 64.2  62.9 | 160 bp | 1.86 = 86 %, 0.997 | -3.709,  40.807 | 37.026  (10) | [2] |
| *Frischella perrara*  (*16S rRNA*, JX8783306) | **F:** GGAAGTTATGTGTGGGATAAGC  **R:** CTATTCTCAGGTTGAGCCCG | 60.1  60.5 | 185 bp | 1.946 =  94.6 %, 0.995 | -3.457,  38.042 | 31.704  (100) | this study |
| *Snodgrassella alvi*  (*16S rRNA*, JQ746651) | **F:** CTTAGAGATAGGAGAGTGCCTT  **R:** AACTTAATGATGGCAACTAATGACAA | 60.1  60.1 | 132 bp | 1.966 =  96.6 %, 0.999 | -3.406,  38.236 | 31.256  (100) | [3], modified |
| *Bartonella apis*  (*16S rRNA*, KP987885) | **F:** GTGGGAATCTACCTATTTCTACG  **R:** AACGCGGGCTCATCTATCTC | 60.9  60.5 | 103 bp | 2.051 =  105.1 %, 0.997 | -3.205,  36.771 | 30.752  (100) | this study |
| Alpha 2.1  (*16S rRNA*, HM111875) | **F:** AGGGATCTGTCCATAAGAGGG  **R:** CAAACGCAGGTTCCTCCATAAG | 61.2  62.1 | 105 bp | 2.002 =  100.2 %, 0.998 | -3.318,  37.335 | 30.865  (100) | this study |
| *Bifidobacterium asteroides*  (*16S rRNA*, AB437355) | **F:** ATGCAAGTCGAACGGGATCC  **R:** CATCCCATRCCGGTAAACCC | 60.5  60.5 – 62.5 | 174 bp | 1.948 =  94.8 %, 0.997 | -3.453,  38.983 | 34.906  (10) | this study |
| *Lactobacillus* Firm-4  (*16S rRNA*, DQ837632) | **F:** AGTCGAGCGCGGGAAGTCA  **R:** AGCCGTCTTTCAACCAGCACT | 61.6  61.2 | 169 bp | 1.912 =  91.2 %, 0.999 | -3.554,  37.196 | 29.966  (100) | this study |
| *Lactobacillus* Firm-5  (*16S rRNA*, JX099547) | **F:** GCAACCTGCCCTWTAGCTTG  **R:** GCCCATCCTKTAGTGACAGC | 60.5  60.5 – 62.5 | 118 bp | 2.144 =  114.4 %, 0.998 | -3.02,  36.306 | 29.94  (100) | this study |
| *Lactobacillus kunkeei*  (*16S rRNA*, JQ009342) | **F:** TGGGTAACCTGCCCCGAAG  **R:** TCTTGGTGGGCTTTTATCTCAC | 61.6  60.1 | 162 bp | 1.873 =  87.3 %, 0.997 | -3.669,  40.227 | 36.309  (10) | this study |
| Universal bacteria (*16S rRNA*) | **F:** AGGATTAGATACCCTGGTAGTCC  **R:** YCGTACTCCCCAGGCGG | 62.9  60 – 62 | variable | 1.946 =  94.6 %, 0.993 | -3.457,  37.986 | 33.546  (10) | this study |
| VDV-1 (complete genome, AY251269) | F: GTATATATGGCTAATCGACGTAAAG  R: AGTACTAATCTCTGAGCCAACAC | 60.9  60.9 | 210 bp | 89.2%,  0.999 | -3.61,  40.805 | 33.736  (100) | [4], modified |

^a^Melting temperatures were calculated with the online tool described in [5]. ^b^*LOD* refers to the limit of detection of primers sets, here expressed as the lowest number of plasmid copies detected by qPCR method when standard curves were performed.

**References:**

1. Zufelato MS, Lourenço AP, Simões ZL, Jorge JA, Bitondi MM. Phenoloxidase activity in *Apis mellifera* honey bee pupae, and ecdysteroid-dependent expression of the prophenoloxidase mRNA. Insect Biochem Mol Biol. 2004; 34:1257-68. Doi: 10.1016/j.ibmb.2004.08.005.

2. Engel P, Bartlett KD, Moran NA. The bacterium *Frischella perrara* causes scab formation in the gut of its honeybee host. mBio. 2016; 6: e00193-15. doi:10.1128/ mBio.00193-15.

3. Martinson VG, Danforth BN, Minckley RL, Rueppell O, Tingek S, Moran NA. A simple and distinctive microbiota associated with honey bees and bumble bees. Mol Ecol. 2011; 20: 619–628. doi: 10.1111/j.1365-294X.2010.04959.

4. Zioni N, Soroker V, Chejanovsky N. Replication of Varroa destructor virus 1 (VDV-1) and a Varroa destructor virus 1-deformed wing virus recombinant (VDV-1-DWV) in the head of the honey bee. Virology. 2011; 417:106-12. doi: 10.1016/j.virol.2011.05.009.

5. Kibbe WA. OligoCalc: an online oligonucleotide properties calculator. Nucleic Acids Res. 2007; 35: W43–W46. doi:10.1093/nar/gkm234
