# Supplementary material for: Disentangling metabolic functions of bacteria in the honey bee gut
Source: PLoS Biol. 2017 Dec 12;15(12):e2003467. doi: 10.1371/journal.pbio.2003467 (PMC5726620; doi:10.1371/journal.pbio.2003467)
Supplement: S3 Table — (DOCX) [file pbio.2003467.s023.docx]

**S3 Table**. List of ions explained *in vivo* and *in vitro* by the same community members.

| **Ion #** | **Annotation^a^** | **Compound class** | **Species^b^** | **Type** | **Process involved** |
| --- | --- | --- | --- | --- | --- |
| 50 | 2-Furoate | Furans | Fp | Substrate | furans have been identified in bee pollen |
| 61 | Fumarate | Fatty Acids and Conjugates | F5 | Substrate | substrate of lactobacilli to produce succinate |
| 111 | (S)-Malate | Carboxylic Acids and Derivatives | Ga | Substrate | TCA cycle intermediate |
| 269 | Glycyl-leucine | Peptides | F4 | Substrate | dipeptide with known bacteriostatic activity |
| 276 | EPTC | - | F4 | Substrate | herbicide, accumulating in pollen |
| 285 | Citrate | Carboxylic Acids and Derivatives | Fp | Substrate | TCA cycle intermediate, can be fermented into succinate and others |
| 286 | Quinate | - | Ba, Ga | Substrate |  |
| 349 | Dethiobiotin | Fatty Acids and Conjugates | F4 | Substrate | Precursor of biotin (vitamin B7) |
| 364 | D-Lysopine | - | F4 | Substrate | Lysine precursor/degradation product |
| 496 | Inosine | Purine Nucleosides and Analogues | F4 | Substrate | Nucleotide precursor |
| 575 | 6-Nitrobenzo[a]pyrene | - | F5 | Substrate | Polycyclic aromatic compound and environmental chemical pollutant |
| 595 | 3',5'-Cyclic CMP | - | F5 | Substrate | cAMP, effect on pollen tube and self-incompatibility |
| 757 | Acetyl-maltose | - | F5 | Substrate | Carbohydrate derviative from starch breakdown |
| 821 | Afzelin | Flavonoids | F4, F5 | Substrate | Glycosylated flavonoid |
| 835 | Quercitrin | Flavonoids | Bi, F4, F5 | Substrate | Glycosylated flavonoid |
| 981 | Scolymoside | Flavonoids | F5 | Substrate | Glycosylated flavonoid |
| 995 | Rutin | Flavonoids | F5 | Substrate | Glycosylated flavonoid |
| 200 | 3-(2-Hydroxyphenyl)propanoate | Phenols and Derivatives | F4, F5 | Product | Aromatic compound degradation intermediate |
| 174 | 2-Maleylacetate |  | F5 | Product | Aromatic compound degradation intermediate |
| 230 | 2-Hydroxy-3-oxoadipate |  | F5 | Product | Aromatic compound degradation intermediate |
| 515 | 1,6-Anhydro-N-acetyl-beta-muramate |  | Ga | Product | Bacterial cell wall component |

^a^ Only top annotation is shown. Asterisks indicate alternative annotations.

^b^ List of species responsible for a given ion change *in vivo* and *in vitro*; F4, Firm-4; F5, Firm-5; Bi, *B. asteroides;* Fp, *F. perrara*; Ga, *G. apicola*; Ba, *B. apis*.
