# Supplementary material for: Disentangling metabolic functions of bacteria in the honey bee gut
Source: PLoS Biol. 2017 Dec 12;15(12):e2003467. doi: 10.1371/journal.pbio.2003467 (PMC5726620; doi:10.1371/journal.pbio.2003467)
Supplement: S4 Table — (DOCX) [file pbio.2003467.s024.docx]

**S4 Table.** List of ions for which we detected possible cross-feeding based on ANOVA results.

| **Ion#** | **Annotation** | **Donors** | **Consumers** | **# Annotations** |
| --- | --- | --- | --- | --- |
| 23 | Pyruvate* | Ba;Ga | CL;F5;Sa | 4 |
| 97 | N-Methylhexanamide | Bi;Ga | Ba;Sa | 1 |
| 189 | Anhydrogalactose* | Bi;F5 | CL;Sa | 19 |
| 282 | 5-Hydroxyindoleacetate* | Bi;F5 | CL;F4 | 3 |
| 288 | alpha-Ionone* | Bi;F4 | F5;Fp | 3 |
| 324 | S-(2-Hydroxyethyl)-N-acetyl-L-cysteine | Bi;Fp | Ba;Sa | 1 |
| 448 | N-Caffeoylputrescine* | Bi;Ga | CL;F5 | 2 |
| 454 | Prosulfocarb | Bi;Ga | CL;F5 | 1 |
| 498 | Histidylleucine | Fp;Ga | F4;F5 | 1 |
| 542 | Riccionidin A | Ba;Fp;Ga | CL;F5 | 1 |
| 566 | N-Glycosyl-L-asparagine | Ba;Fp;Ga | CL;F5 | 1 |
| 568 | (9Z)-(13S)-12,13-Epoxyoctadeca-9,11-dienoic acid* | Ba;Sa | CL;F4 | 11 |
| 575 | 6-Nitrobenzo[a]pyrene* | Ba;Fp;G;Sa | CL;F5 | 2 |
| 617 | Isazofos | Ba;Bi | CL;F5 | 1 |
| 627 | 9,10-Dihydroxystearate* | Ba;Sa | Bi;CL | 3 |
| 650 | Anisatin* | Ba;F4 | Bi;CL;F5;Fp | 2 |
| 658 | 3,7-Di-O-methylquercetin* | Bi;F5 | CL;Fp | 8 |
| 666 | 9,10,18-Trihydroxystearate | F5;Ga | Bi;CL;F4 | 1 |
| 722 | Nitrovin | Bi;F5 | CL;Fp | 1 |
| 800 | Magnoshinin* | F5;Ga | Ba;B;F4 | 4 |
| 808 | Decyl oleate | Ba;Sa | CL;F5 | 1 |
| 837 | Estradiol-17alpha 3-D-glucuronoside* | F5;Ga | Ba;B;F4 | 4 |
| 839 | Laserpitin | Ga;Sa | Bi;F4 | 1 |
| 902 | β-fabatriose | Ga;Sa | Bi;CL | 1 |
| 909 | N-(Tetradecanoyl)-sphing-4-enine | Ba;Sa | CL;F4;F5 | 1 |
| 981 | Scolymoside* | Ba;Fp;Ga;Sa | CL;F5 | 8 |

^a^ ions with alternative annotations are marked with an asterisks.

**^b^** Ba, *B. apis,* F4, Firm-4, F5, Firm-5, Bi, *B. asteroides,* Fp, *F. perrara*, Ga, *G. apicola,* Sa, *S. alvi*.
