# Supplementary material for: Disentangling metabolic functions of bacteria in the honey bee gut
Source: PLoS Biol. 2017 Dec 12;15(12):e2003467. doi: 10.1371/journal.pbio.2003467 (PMC5726620; doi:10.1371/journal.pbio.2003467)
Supplement: S5 Table — (DOCX) [file pbio.2003467.s025.docx]

**S5 Table.** Total number of bees analyzed by qPCR and number of bees selected for metabolomics analysis.

|  | no. of analyzed bees | | no. of selected bees^a^ | | |
| --- | --- | --- | --- | --- | --- |
| Treatment | Experiment 1 | Experiment 2 | Experiment 1 | Experiment 2 | Final |
| MD | 19 | 44 | 8 | 13 | **21** |
| CL | 10 | 17 | 6 | 12 | **18** |
| Ga | 12 | 14 | 5 | 0 | **5** |
| Fp | 7 | 10 | 4 | 9 | **13** |
| Sa | 16 | 15 | 6 | 3 | **9** |
| Ba | 11 | 9 | 0 | 5 | **5** |
| Bi | 10 | 20 | 0 | 5 | **5** |
| F4 | 7 | 10 | 0 | 6 | **5** |
| F5 | 12 | 5 | 7 | 5 | **12** |
| Hive | 12 | 10 | 0 | 5 | **5** |
| TOTAL | **116** | **154** | **47** | **63** | **109** |

^a^ bees were selected based on qPCR results with the species-specific primers. Some bees had to be excluded from further analysis due to contaminations with other bacteria or due to elevated levels of VDV-1 virus. Hence, the number of bees varied between treatment groups. At least 5 bees per treatment group were analyzed.
