# Supplementary material for: Disentangling metabolic functions of bacteria in the honey bee gut
Source: PLoS Biol. 2017 Dec 12;15(12):e2003467. doi: 10.1371/journal.pbio.2003467 (PMC5726620; doi:10.1371/journal.pbio.2003467)
Supplement: S1 Text — (PDF) [file pbio.2003467.s026.pdf]

S1 Text.

MS/MS analysis to establish identity of selected flavonoids.

Because ions annotated as glycosylated flavonoids displayed remarkable biological patterns in both the *in vivo* and *in vitro* datasets, we aimed to get more certainty on their annotation using MS/MS fragmentation. Dramatically changing ions with glycosylated flavonoid annotation from representative microbiota-depleted (MD) gut samples were selected for fragmentation. These were ions #821 (*m/z* 431.099, confirmed as afzelin), #835 (*m/z* 447.093, confirmed as quercitrin), #853 (*m/z* 461.109, first annotation name 6-methoxyluteolin 7-rhamnoside, 5 annotations), #981 (*m/z* 593.151, likely scolymoside), and #995 (*m/z* 609.146 m/z, confirmed as rutin), with ion numbers (#) corresponding to those in the *in vivo* dataset (**S2A Data**). Pure flavonoid standards were commercially available for vitexin (alternative annotation for #821), rutin, and quercitrin, and 50 μM of these standards were fragmented accordingly. Fragmentation of the precursor ion was performed by collision-induced dissociation, and fragment-ion spectra were recorded in scanning mode by high-resolution time-of-flight MS. Since flow injection analysis was used, this procedure cannot exclude that a single *m/z* feature corresponds to two or more metabolites and is thus biased towards identifying the most dominant metabolite. However, this would be reflected in poorer similarity values for the matches.

Spectra and analysis results are presented in **S4 Data**. For interpretation we decided on the following pipeline. After extracting the most abundant peaks from the product spectra we used MetFrag 2.3 [1] on the PubChem database (https://pubchem.ncbi.nlm.nih.gov/) to confirm that the top hits had the same

molecular formula and were from the same compound class, i.e. flavonoids. In PubChem the presence of the “chromen-4-one” signature in the IUPAC name was used as sign for flavonoids, since this bicyclic heterocycle with a benzene ring fused to one of dihydro-pyran is the main building block of flavonoid aglycones. After confirming flavonoid identity, we queried the KEGG database with MetFrag for detailed inspection of matched flavonoid fragmentation patterns between the alternative annotations. Note that fragmentation patterns and interpretation are the same between PubChem and KEGG databases. KEGG was only selected in this case to obtain the same names as those used in the ion annotations. When available, spectral similarities were compared to reference spectra obtained in-house or library spectra from the MassBank of North America (MoNA, http://mona.fiehnlab.ucdavis.edu/), and similarity was calculated as spectral cosine similarity scores [2] in R using OrgMassSpecR v. 0.4.4 [3], RChemMass v. 0.1.8 [4] and ReSOLUTION v. 0.1.3 [5]. Certainty levels in the annotations were expressed as described by Schymanski et al. [6].

The first ion was #821 (*m/z* 431.099, confirmed as afzelin). MetFrag analysis matched 7 of the selected 11 most dominant peaks. The top scoring annotation of this matching was for the afzelin flavonoid, with a MetFrag score of 1.89 compared to <1.53 for the alternative annotations (**S4 Data**). Indeed, detailed inspection of the fragments matching to afzelin showed that fragments correspond to the sugar part, fragments of the aglycone, and the full kaempferol aglycone. In contrast, matching the observed fragments to the next-best match, vitexin, showed that the expected mass corresponding to the intact apigenin aglycone was not matched fully, while

apigenin was matched in the fragments of the vitexin standard (3/6 peaks matched). We next computed a spectral similarity of 0.92 (maximum of 1) with an afzelin reference spectrum from the MoNA database (CCMSLIB00000845703) (**S3 Fig**). In contrast, the similarity of ion #821 with a vitexin reference standard measured in-house under the same conditions was 0.14. Thus, ion #821 (*m/z* 431.099) corresponds predominantly to afzelin (Level 2a).

The second ion was #835 (*m/z* 447.093, confirmed as quercitrin). MetFrag analysis matched 12 of the selected 18 most dominant peaks. The top scoring annotation resulting from matching the product spectra was indeed the quercitrin flavonoid, with a MetFrag score of 1.89 compared to <1.71 for the alternative annotations (**S4 Data**). Detailed inspection of the fragments matching to quercitrin showed that fragments correspond to the sugar part, sub fragments of the aglycone, and the full quercetin aglycone. The same fragments were obtained when fragments obtained for the quercitrin standard were analyzed (17/22 peaks matched). Spectral similarity between the spectrum of ion #835 and the pure reference spectrum was reasonably high with 0.62. However, the differences in relative intensities between the experimental and reference spectrum suggest that ion #835 could contain signals from another metabolite besides quercitrin, for instance the second MetFrag hit plantaginin. Still, considering quercitrin is the top scoring metabolite and knowing that it is one of the main flavonoids in pollen [7], ion #835 (*m/z* 447.093) most likely corresponds predominantly to quercitrin (Level 2a).

The third ion was #853 (*m/z* 461.109, first annotation name 6-methoxyluteolin 7-rhamnoside). MetFrag analysis matched 10 of the selected 25

most dominant peaks from the product spectra. However overall intensity counts were low for this ion, suggesting relatively low abundance in the MD bees. Keeping the resulting uncertainty in mind, the top scoring annotation from matching the product spectra was for the 6-methoxyluteolin 7-rhamnoside flavonoid, with a MetFrag score of 1.90 compared to <1.73 for the alternative annotations (**S4 Data**). Indeed, detailed inspection of the fragments matching to 6-methoxyluteolin 7-rhamnoside showed that fragments correspond to the sugar part, sub fragments of the aglycone, and the full methoxyluteolin aglycone. In contrast, matching the observed fragments to isoscoparine showed that the mass corresponding to the intact chrysoeriol aglycone was not matched fully. No MoNA reference spectra were available for the various annotations of this ion. With the inherent uncertainty we decided to name ion #853 (461.109 m/z) 6-methoxyluteolin 7-rhamnoside but the confidence of this specific annotation is not high (Level 3).

The fourth ion was #981 (*m/z* 593.151, likely scolymoside). MetFrag analysis matched 10 of the selected 14 most dominant peaks and attributed a score of around 2 to three annotations, the isoorientin-O-rhamnoside, multiflorin B, and scolymoside flavonoids (**S4 Data**). We queried MoNA for reference spectra for these three flavonoids but only found a relevant spectrum for scolymoside. The spectral similarity of spectrum CCMSLIB00000846797 with the observed spectrum for ion #981 was very high with 0.94. The main fragment from this spectrum includes the oxygen linking the aglycone and sugar, and this linkage is not present for isoorientin-O-rhamnoside. This spectral similarity strongly suggests that this ion is predominantly either multiflorin B or scolymoside. Because of the high similarity

and absence of a reference spectrum for multiflorin B, we suggest naming ion #981 (m/z 593.151) scolymoside, but a mixed or alternative annotation of multiflorin B cannot be excluded (Level 3).

The fifth ion was #995 (*m/z* 609.146, confirmed as rutin). MetFrag analysis matched 11 of the selected 21 most dominant peaks, and attributed a score of around 2 to two annotations, the rutin and multinoside A flavonoids (**S4 Data**). Detailed inspection of the fragments matching to rutin showed that fragments correspond to the sugar part, sub fragments of the aglycone, and the full quercetin aglycone. Overlapping fragments were obtained when fragments obtained for the rutin standard were analyzed (5/6 matched). We queried MoNA for reference spectra for multinoside A (CCMSLIB00000846096) and computed a spectrum similarity score of 0.48 with the spectrum for ion #995. In contrast, the spectral spectrum similarity score of this spectrum with that of the rutin standard was 0.88. Because this score was predominantly based on one ion we cannot exclude a possible mixed composition of this ion. Still, considering rutin is the top scoring metabolite, has a spectral similarity, and knowing that it is one of the main flavonoids in pollen [7] leads us to conclude that ion #995 (*m/z* 609.146) most likely corresponds predominantly to rutin (Level 2a).

References:

1. Ruttkies C, Schymanski EL, Wolf S, Hollender J, Neumann S. MetFrag relaunched: incorporating strategies beyond in silico fragmentation. J Cheminformatics. 2016; 8: 3. doi:10.1186/s13321-016-0115-9

2. Stein SE, Scott DR. Optimization and testing of mass spectral library search algorithms for compound identification. J Am Soc Mass Spectrom. 1994; 5: 859–866. doi:10.1016/1044-0305(94)87009-8.

3. Dodder NG. OrgMassSpecR: Organic Mass Spectrometry. R package version 0.4-4. https://CRAN.R-project.org/package=OrgMassSpecR. and with code contributions from Katharine M. Mullen; 2014.

4. E.L. Schymanski. RChemMass: Various Cheminformatic and Mass Spectrometry Functions. R package version 0.1.8. Eawag, Dübendorf, Switzerland. Code available upon request.; 2017.

5. Schymanski EL. ReSOLUTION: SOLUTIONS for High ReSOLUTION Mass Spectrometry. R package version 0.1.3. Eawag, Dübendorf, Switzerland. Code available upon request.; 2017.

6. Schymanski EL, Jeon J, Gulde R, Fenner K, Ruff M, Singer HP, et al. Identifying Small Molecules via High Resolution Mass Spectrometry: Communicating Confidence. Environ Sci Technol. 2014; 48: 2097–2098. doi:10.1021/es5002105.

7. Rzepecka-Stojko A, Stojko J, Kurek-Górecka A, Górecki M, Kabała-Dzik A, Kubina R, et al. Polyphenols from Bee Pollen: Structure, Absorption, Metabolism and Biological Activity. Molecules. 2015; 20: 21732–21749. doi:10.3390/molecules201219800.
