# Supplementary material for: Disentangling metabolic functions of bacteria in the honey bee gut
Source: PLoS Biol. 2017 Dec 12;15(12):e2003467. doi: 10.1371/journal.pbio.2003467 (PMC5726620; doi:10.1371/journal.pbio.2003467)
Supplement: S2 Text — (PDF) [file pbio.2003467.s027.pdf]

S2 Text.

Overlap between metabolomics data in Zheng et al. and this study.

In the recent study by Zheng et al. [1] untargeted GC-MS was used to profile several bee tissues, namely hemolymph, ileum, midgut, and rectum. In our study we used direct injection coupled with MS (FIA-qTOF) on whole gut samples. We inspected the overlap between the annotations between the two studies. Zheng et al. [1] annotate 238 ions, we annotate 1079 ions. Based on the supplied KEGG ids, the overlap between these annotation sets is 150 ions, or 63% (**S5 Data**). Because our whole gut samples contain mostly rectum we used the values of the rectum samples from Zheng et al. [1] (obtained from their Supplemental information S2) to compare the observed changes.

The first observation from the overall comparison between the metabolome changes in both studies is that for ions that overlap, the magnitudes of the fold changes between CL and MD bees are smaller in the FIA-qTOF data than in the GC-MS data of Zheng et al. [1]. These fold change differences are most intuitively explained by the rectum being only a part of the whole gut metabolomics sample in the FIA-qTOF data. The Pearson correlation of fold changes of the 150 overlapping ions was 0.28 across both datasets. Besides the difference in sample location, this relatively low correlation was also expected, because most of the overlapping ions do not, or barely change in abundance between CL and MD bees. We thus argued that only considering the ions that change most in both methods would result in a larger correlation between the observed fold changes of the two methods. To this

end, we first took the top 50% changing ions (≥ median in absolute fold changes) for the overlapping ions in both datasets.

The 38 ions that were present in the top 50% changing in both datasets displayed an improved correlation in log2(fold change) of 0.60. Interestingly, this set of 38 ions contains two out of three polyphenol breakdown products annotated in both studies. When in similar manner only the top quartile was considered this lead to 9 ions (isopropylmalic acid, gluconic acid, quinic acid, kynurenine, inosine, thymidine, linoleic acid, linolenic acid, and pinitol), with a correlation in log2(fold change) of 0.63. This suggests that for ions displaying a robust change the overlap between both methods is acceptable, and this despite the different sampling sites, composition of the microbiota (gut homogenate versus selected strains) and metabolite measurements.

To obtain more details on specific metabolite changes between the two studies, we inspected the main categories of interest emerging from our study. Since no flavonoids were determined by Zheng et al. [1], we selected polyphenol breakdown products, nucleosides, and organic acids for the comparison. The polyphenol breakdown products belong to the metabolites accumulating in the presence of the microbiota, while the nucleosides are substrates of the microbiota.

Polyphenol breakdown products annotated in both studies are hydroxyphenylacetic acid, hydroxyphenylpropionic acid, and p-hydroxylphenyllactic acid, with the latter two being alternative annotations of a single ion in our FIA-qTOF data (#247). An additional polyphenol breakdown product that was not annotated in our dataset is dihydroxyphenylacetic acid.

Remarkably, these polyphenol breakdown products display similar log2(fold change) when rectum samples from Zheng et al. [1] are compared with whole gut in our data, with a log2(fold change) of ~2 (i.e. higher in CL bees). Interestingly, Zheng et al. [1] report these changes to be most dramatic in the rectum compared to other tissues, consistent with our hypothesis that flavonoid breakdown takes place in the hindgut. This localization is also concordant with the observation that changes are a bit more pronounced in the Zheng et al. [1] rectum data than on whole gut in the FIA-qTOF data, since the rectum is only part of the whole gut and changes observed by us therefore will be diluted.

Nucleosides annotated in both studies are adenosine, guanosine, inosine, and thymidine (**S5 Data**). Not annotated in our dataset is deoxyguanosine, but since deoxyguanosine has the same mass as adenosine these two compounds could anyway not have been distinguished. For guanosine, inosine, and thymidine the changes are consistent between both studies (negative fold changes), in line with our classification of nucleosides as bacterial substrates originating from pollen. As above for the polyphenol breakdown products the fold changes are more pronounced in the dataset of Zheng et al. [1]. Adenosine patterns do not overlap since the data from Zheng et al. [1] suggests production of this nucleoside in the midgut while our FIA-qTOF data suggests the ion corresponding to adenosine originating from pollen and being depleted by specific members of the microbiota. It seems that the pattern observed by us could be the sum of the change for adenosine and deoxyguanosine, where adenosine mildly accumulates with log2(fold change) 0.6, and deoxyguanosine strongly decreases with a log2(fold change) of -6.4. Overall,

our *in vitro* data is in support of gut bacteria utilizing adenosine or deoxyguanosine, as the corresponding ion was depleted during growth of Firm-4, Firm-5, and *B. asteroides* (**S9 Data**).

Organic acids annotated in both untargeted metabolomics datasets are succinate, fumarate, malate, citrate, and pyruvate. The log2(fold change) of these ions show high overlap, with succinate being the only organic acid that accumulates in rectum and total gut (log2(fold change) 1.4 and 2.5, respectively), showing it is a bacterial product. Fumarate displays a less pronounced negative log2(fold change) in the Zheng et al. [1] study compared to our study but the other organic acids are all depleted with more than -1.5 to -3 log2(fold change). This supports our classification of bacterial substrates as was outlined above. The high correlation between the organic acids further confirms the concordance between the two independent untargeted metabolomics studies.

References:

1. Zheng H, Powell JE, Steele MI, Dietrich C, Moran NA. Honeybee gut microbiota promotes host weight gain via bacterial metabolism and hormonal signaling. Proc Natl Acad Sci. 2017; 114: 4775–4780. doi:10.1073/pnas.1701819114
