# Supplementary material for: Disentangling metabolic functions of bacteria in the honey bee gut
Source: PLoS Biol. 2017 Dec 12;15(12):e2003467. doi: 10.1371/journal.pbio.2003467 (PMC5726620; doi:10.1371/journal.pbio.2003467)
Supplement: S8 Data — (ZIP) [file 1003 microbial substrate N1,N5,N10-Tricaffeoyl spermidine.pdf › Z-score_plots/1003 microbial substrate N1,N5,N10-Tricaffeoyl spermidine.pdf]

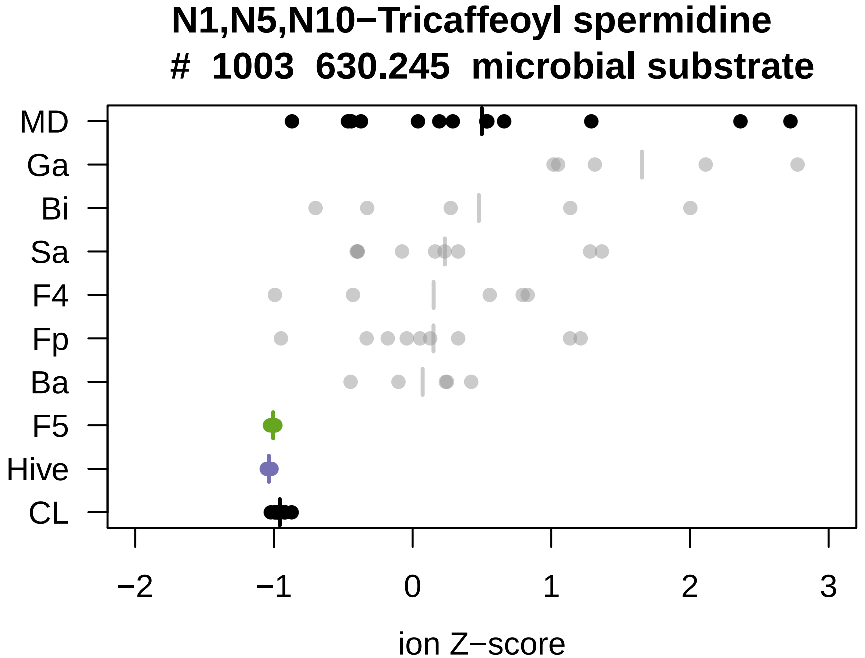

N1,N5,N10−Tricaffeoyl spermidine
1003 630.245 microbial substrate
MD
Ga
Bi
Sa
F4
Fp
Ba
F5
Hive
CL
−2
−1
0
1
2
3
ion Z−score
