# Supplementary material for: Disentangling metabolic functions of bacteria in the honey bee gut
Source: PLoS Biol. 2017 Dec 12;15(12):e2003467. doi: 10.1371/journal.pbio.2003467 (PMC5726620; doi:10.1371/journal.pbio.2003467)
Supplement: S8 Data — (ZIP) [file 316 microbial product Xanthurenic acid.pdf › Z-score_plots/316 microbial product Xanthurenic acid.pdf]

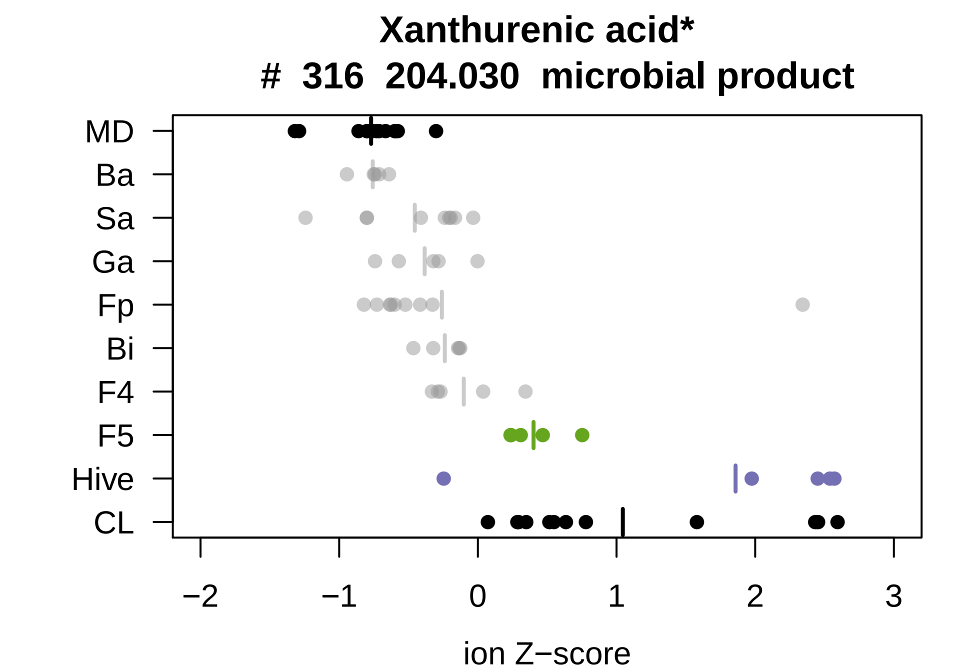

Xanthurenic acid*
316 204.030 microbial product
MD
Ba
Sa
Ga
Fp
Bi
F4
F5
Hive
CL
−2
−1
0
1
2
3
ion Z−score
