# Supplementary material for: Disentangling metabolic functions of bacteria in the honey bee gut
Source: PLoS Biol. 2017 Dec 12;15(12):e2003467. doi: 10.1371/journal.pbio.2003467 (PMC5726620; doi:10.1371/journal.pbio.2003467)
Supplement: S11 Data — (ZIP) [file supplement_v3_nucleosides.pdf › expectedResults/supplement_v3_nucleosides.pdf]

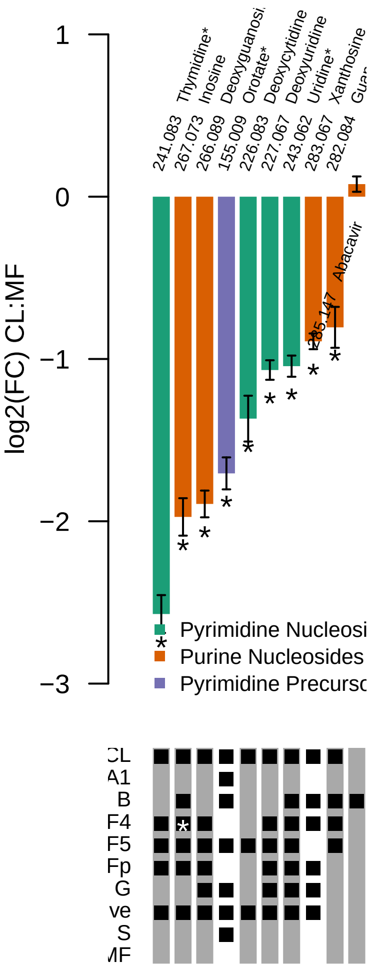

1
0
−1
−2
−3
log2(FC) CL:MF
Thymidine*
Inosine
Deoxyguanosi
Orotate*
Deoxycytidine
Deoxyuridine
Uridine*
Xanthosine
241.083
267.073
266.089
155.009
226.083
227.067
243.062
283.067
282.084
Abacavir
285.147
Pyrimidine Nucleosi
Purine Nucleosides
Pyrimidine Precurso
CL
A1
B
F4
F5
Fp
G
ve
S
