# Supplementary material for: Disentangling metabolic functions of bacteria in the honey bee gut
Source: PLoS Biol. 2017 Dec 12;15(12):e2003467. doi: 10.1371/journal.pbio.2003467 (PMC5726620; doi:10.1371/journal.pbio.2003467)
Supplement: S3 Text — (PDF) [file pbio.2003467.s028.pdf]

## S3 Text.

## Overlap between targeted and untargeted metabolomics for CL and MD bees.

To get more certainty on annotations in general and to quantify the extent to which some of the organic acids accumulated in our samples, we used ultra-high performance liquid chromatography-coupled tandem mass spectrometry ((UHPLC)-MS/MS) with previously published settings [1] on gut samples from colonized (CL) and microbiota-depleted (MD) bees, and on pollen extracts. This allowed for the identification of 17 chromatogram peaks, 15 corresponding to unique metabolites and two corresponding to either hexoses or disaccharides. Among the identified metabolites were eight amino acids; arginine, asparagine, glutamine, tyrosine, phenylalanine, aspartate, glutamate, and tryptophan. Besides these amino acids, the nucleobase adenine was identified, the uric acid glucuronate, plus five organic acids, being pyruvate, lactate, succinate, malate, and fumarate (**S6 Data**).

Of these 15 metabolites, 12 were also annotated in the untargeted FIA qTOF data (**S2A Data**). For these 12, we first computed the log2(fold-change) of the CL samples compared to MD samples and inspected the correlation between the two methods. We determined a Pearson correlation with an $R^2$ of 0.77 (**S4A Fig**). From this figure it is again apparent that most amino acids do not change markedly between CL and MD bees, consistent with them being taken up by the host in case of diet-derived amino acids. This trend excludes aspartate and asparagine, which seem to be utilized by the microbiota. Organic acids displayed the largest change, with

succinate being produced by the microbiota, and fumarate and pyruvate being most strongly consumed by the microbiota (**S4A Fig**).

Encouraged by the overlap in log2(fold change) we used standard curves to attempt to quantify the identified metabolites, focusing on the organic acids because they display the largest change in the CL vs MD comparison. To attempt to infer the origin of the five organic acids we also determined their concentration in pollen extracts, and expressed concentrations for the samples originating from gut and pollen as millimole per gram tissue. Note however that the gut samples will obviously not fully consist of pollen grains, making the pollen concentrations an overestimation for direct comparisons.

The concentrations of the five organic acids are in the low millimoles per gram tissue range (corresponding to μM range per undiluted sample) (**S4B Fig**). These are physiologically relevant concentrations while accumulation does not seem as dramatic as is reported for instance for short chain fatty acids in mammals, these being in the 100 mM range. Besides this, three clearly interpretable patterns could be distinguished for succinate, malate, fumarate, and lactate.

The first pattern is represented by a strong accumulation in CL bees and was observed for succinate, confirming that succinate is a product of the microbiota that does not seem to be taken up by the host. The second pattern is characterized by presence in pollen and depletion only in CL bees. Malate and fumarate display this pattern, suggesting that malate and fumarate cannot be taken up by the host directly, but are substrates of the microbiota. Pyruvate shows a similar trend since it is also depleted in CL guts, but is present at much higher concentrations in MD guts

than in pollen extracts. This suggests that pyruvate, and to a lesser extent malate and fumarate might be actively or passively produced in the bee gut, potentially to supply the microbiota with energy. Lactate displays the last pattern suggesting host uptake. Lactate is present in considerable amount in the pollen extract but completely depleted in both CL and MD bees. This observation is relevant because lactate is expected to be a main fermentation product of *Lactobacilli*.

To summarize, we conclude that the log2(fold change) obtained in both methods correlate sufficiently to have confidence in the ion annotations of the untargeted metabolomics data and that some organic acids are present in considerable amounts in either the guts of CL or MD bees, or pollen extract. Whereas malate, fumarate, and pyruvate seems to originate either from the pollen diet or the host, succinate is the main accumulating product originating from fermentation of the honey bee microbiota but is not taken up by the host. Lactate seems to be taken up by the host and consistent with this does not accumulate in CL guts, while its production is expected via sugar fermentation by the *Lactobacilli* in the Firm-4 and Firm-5 group.

**References:**

1. Buescher JM, Moco S, Sauer U, Zamboni N. Ultrahigh performance liquid chromatography–tandem mass spectrometry method for fast and robust quantification of anionic and aromatic metabolites. Anal Chem. 2010; 82: 4403–4412. doi:10.1021/ac100101d.
