# Supplementary material for: Disentangling metabolic functions of bacteria in the honey bee gut
Source: PLoS Biol. 2017 Dec 12;15(12):e2003467. doi: 10.1371/journal.pbio.2003467 (PMC5726620; doi:10.1371/journal.pbio.2003467)
Supplement: S4 Text — (PDF) [file pbio.2003467.s029.pdf]

S4 Text.

Pollen ions and targeted metabolomics on pollen extracts.

To enrich for substrates that likely originate from pollen, we profiled the metabolome of pollen extract (two replicate extractions, five samples each) with untargeted metabolomics, and selected ions with a relatively high minimal intensities and strongly significant and large fold change difference (log2(fold change) ≥ 2, Welch BH-adjusted P-value ≤ 0.001) compared to control water samples. This resulted in 402 of 1,079 ions, on which enrichment was computed using a one-sided Fishers exact test (**S6A Fig**). Consistent with what is known about pollen composition [1,2], the 402 resulting ions were significantly enriched for expected annotation categories, with *Amino acids and derivatives*, *Flavonoids*, and *Monosaccharides* displaying the highest level of enrichment (34/63, 29/36, and 14/19 ions, respectively, one-sided Fishers exact test P <0.001), and *Carboxylic acids and derivatives* showing a lower level of enrichment (15/26 ions, P <0.03). To determine whether pollen extracts contain physiologically meaningful levels of nutrients we performed quantitative (UHPLC)-MS/MS on pollen extracts [3]. Peaks for 17 metabolites could be integrated from three replicate pollen extract samples, 11 of which could be reliably quantified by interpolation from standard curves (**S6B Fig**). In addition, 12 of the 17 metabolites were also annotated in the untargeted metabolomics data (**S2A Data**), and 10 out of these 12 were present in the 402 ions that were enriched in pollen extracts using untargeted metabolomics. In the hypothetical case of perfect extractions from pollen grains, these quantification show that pollen contains between 10-30 millimoles per gram pollen for five amino

acids (tryptophan, glutamate, aspartate, phenylalanine), and the nucleotide adenine (corresponding to low - mid micromolar in the liquid extract) (**S6 Data**). However, since extraction of these compounds from pollen is probably incomplete these values most likely represent an underestimation. The amino acid asparagine was the most abundant amino acid and the hexose sugars - which cannot be differentiated with this method - the most abundant sugars in pollen extract (**S6 Data**). While many pollen components were not covered in these targeted measurements this shows that pollen grains and extract is a rich food source for the bee and bacteria.

References:

1. Campos MGR, Bogdanov S, Almeida-Muradian LB de, Szczesna T, Mancebo Y, Frigerio C, et al. Pollen composition and standardisation of analytical methods. J Apic Res. 2008; 47: 154–161. doi:10.1080/00218839.2008.11101443.
2. Serra Bonvehí J, Escolà Jordà R. Nutrient Composition and Microbiological Quality of Honeybee-Collected Pollen in Spain. J Agric Food Chem. 1997; 45: 725–732. doi:10.1021/jf960265q.
3. Buescher JM, Moco S, Sauer U, Zamboni N. Ultrahigh performance liquid chromatography−tandem mass spectrometry method for fast and robust quantification of anionic and aromatic metabolites. Anal Chem. 2010; 82: 4403–4412. doi:10.1021/ac100101d.
