# Supplementary material for: Disentangling metabolic functions of bacteria in the honey bee gut
Source: PLoS Biol. 2017 Dec 12;15(12):e2003467. doi: 10.1371/journal.pbio.2003467 (PMC5726620; doi:10.1371/journal.pbio.2003467)
Supplement: S5 Text — (PDF) [file pbio.2003467.s030.pdf]

S5 Text.

Supporting methods.

Bacterial cultures.

Bacterial strains were inoculated from glycerol stocks on different solid media. Bacteria were restreaked twice before they were used for colonization of bees. Depending on the strain (see **S1 Table**), the following media were used for culturing: brain heart infusion agar (BHIA; BD), tryptone yeast glucose agar (TYG; [1], tryptic soy agar (TSA; BD), Colombia agar base with 5 % defibrinated sheep blood (CBA; bioMérieux), and de Man, Rogosa, Sharpe agar (MRSA; Oxoid) supplemented with 2 % fructose (Sigma) and 0.1% L-cysteine (Fluka). All strains were cultivated at 34 – 35°C in either an anaerobic vinyl chamber (Coy, Michigan, USA, gas mix of 8% H_2, 20% CO_2, 72% N_2) or in microaerophilic atmosphere enriched with 5 % CO_2. All strains used in this study are listed in **S1 Table**.

Experimental colonization of newly emerged bees and bee gut sampling.

Bacterial strains were harvested and resuspended in 1x PBS, pelleted by centrifugation at 8000x g and again resuspended in 1x PBS. Bacterial suspensions were adjusted to an OD_{600} of 1 by dilution in 1x PBS. For colonization of newly emerged bees with the reconstituted community (‘CL’ treatment group), bacterial suspensions of the 11 strains listed in **S1 Table** were mixed together (1ml of each), pelleted and resuspended in 3 ml of 1x PBS/sugar water (1:1, v/v). For mono-colonizations of newly emerged bees, one strain per treatment group was used, except for *G. apicola* and Firm-5 for which two and four strains, respectively, were

mixed together at equal proportions. The OD_{600}-adjusted cultures (1ml, 2ml, and 4ml in case of one, two, and four strains, respectively) were pelleted and resuspended in 1.5 ml of 1x PBS/sugar water. One gram of sterilized pollen (treated with 10 MeV electron beam and plated on different media to ensure sterility) was distributed in a 6-well piece cut out of a 96-well plate (referred to as pollen feeder) and 300 µl of the bacterial suspension was added. In case of microbiota-depleted bees (MD treatment group), the pollen was inoculated with 300 µl of sterile 1x PBS/sugar water (1:1, v/v). After 2 h of starvation, 20 newly emerged were transferred to the sterile boxes containing pollen feeders with the inoculated bacteria. Three hours later, sterile sugar water was provided *ad libitum* in 15 ml Falcon tubes. In total, there were nine conditions of experimental colonizations: microbiota-depleted bees (MD), colonized bees (CL, i.e. reconstituted community), and the mono-colonizations (*Bartonella apis*, *Bifidobacterium asteroides*, Firm-4, Firm-5, *F. perrara*, *Gilliamella apicola*, *Snodgrassella alvi*). Bees were exposed every day to 0.1 of queen equivalent (Qeq) of synthetic QMP (Contech Enterprises, USA) to mimic the presence of a queen [2]. The bees were kept in an insect chamber at a constant temperature of 30°C and a humidity of 80% - 90% for ten days.

To dissect the gut, bees were first anesthetized by exposing them to CO_2 and then kept on ice. The mid- and hindgut without the crop, but with attached Malpighian tubules was dissected and the wet weight of each gut sample was determined with an electric balance. All samples were stored at -80°C until further use. Notably, the entire experiment was repeated at two different time-points of the year (spring and fall, referred to as experiment 1 and experiment 2 in this study).

Whenever possible, we included bees from both experiments in our analysis, such as for CL and MD bees. However, this was not possible for all mono-colonizations due to bacterial contaminations (as detected by qPCR) or due to the presence of high levels of viruses. The exact numbers of bees per condition are listed in **S5 Table**.

Preparation of pollen extracts.

To prepare pollen extract, 2 g of sterilized pollen (the same batch also used as diet for gnotobiotic bees) was crushed in a 50ml tube, and aliquots of 50 mg transferred to a bead-beating tube. After adding 1ml Milli-Q® water, pollen was homogenized with the same settings as the gut samples. Subsequently the 20x-diluted gut homogenates and pollen samples were incubated on a pre-heated thermo-mixer (Eppendorf AG) at 80°C and 1400 rpm for 3 min. After each minute, the samples were vortexed for 10 s. Subsequently, the samples were deprived of large particles by centrifugation at 20000x g and 4°C for 5 min followed by another centrifugation of supernatants at 20000x g and 4°C for 30 min. In case of the pollen extracts, all aliquots were pooled, vortexed, redistributed into aliquots, and stored at -80°C until further use.

Compound identification by MS/MS fragmentations.

MS/MS spectra were acquired on an Agilent 6550 time-of-flight mass spectrometer (ESI-iFunnel Q-TOF, Agilent Technologies) operated in negative ionization mode, with instrument settings as described previously [3]. Precursor ions of selected compounds were targeted as $[M\text{-}H]^-$ electrospray derivatives with a window size of

± 4 m/z in Q1. Fragmentation of the precursor ion was performed by collision-induced dissociation at 0, 10, 20, and 40 eV collision energy. Fragment-ion spectra were recorded in scanning mode by high-resolution time-of-flight MS. Results were inspected in Agilent MassHunter Qualitative Analysis software (Agilent Technologies) and spectra at 20 or 40 eV were extracted. Peak intensities in these MS/MS spectra were sorted by their relative abundance and only the 10-30 most abundant ions were kept, depending on the intensities of the background. These peaks were matched with compounds in the PubChem (https://pubchem.ncbi.nlm.nih.gov/) [4] and KEGG (http://www.kegg.jp/) [5] database using MetFrag [6]. Spectral cosine similarity scores [7] were calculated with reference spectra obtained in-house or library spectra from MassBank of North America (MoNA, http://mona.fiehnlab.ucdavis.edu/) in R using OrgMassSpecR v. 0.4.4 [8], RChemMass v. 0.1.8 [9] and ReSOLUTION v. 0.1.3 [10].

DNA/RNA extraction from honey bee guts.

Homogenized gut tissues were thawed and the total volume was adjusted to 500 µl by adding 200 µl Milli-Q® water and 250 µl CTAB lysis buffer (0.2M Tris-HCl, pH 8; 2.8 M NaCl; 0.04 M EDTA, pH 8; 4% CTAB, w/v, dissolved at 56°C; 0.5% β-mercaptoethanol, v/v). Samples (still containing the zirconia beads, see Methods in main text) were homogenized in a Fast-Prep24™5G homogenizer (MP Biomedicals) at 6 m/s for 45 s, briefly centrifuged, and 1 ml of Roti®-Phenol (Carl Roth, pH 7.5-8) was added. After mixing thoroughly, the samples were incubated in a water bath at 64°C for 6 min with occasional shaking. Samples were then transferred to a new

tube containing 400 µl of chloroform, mixed and the phases were separated by centrifugation at 16000x g for 10 min at room temperature. The upper aqueous phase (500 µl) was transferred and mixed with 500 µl phenol:chloroform:isoamyl alcohol (Fischer Bioreagents, pH 6.5). After centrifugation at 16000x g for 3 min at room temperature, the upper aqueous phase was transferred and mixed with the same volume of chloroform. After another centrifugation, 300-350 µl of the upper aqueous phase was transferred to a new tube, mixed with 900 µl of pre-cooled 100% RNase-free ethanol, and incubated overnight at -80°C for precipitation of nucleic acids. Precipitated nucleic acids were pelleted at 16000x g at 4°C for 30 min. Pellets were washed with 900 µl of 70% ethanol, dried for 5-15 min and resuspended in 200 µl of nuclease-free water (Invitrogen) by shaking in a thermo-mixer (64°C, 400 rpm, 10 min). DNA and RNA concentrations were assessed with Qubit™ (Thermo Fisher) and ranged usually between 7 – 15 ng/µl of DNA and 30 - 150 ng/µl of RNA.

Quantitative PCR (qPCR) for determining bacterial loads in gut samples.

All qPCR reactions were carried out in a 96-well plate on a StepOnePlus instrument (Applied Biosystems) with the thermal cycling conditions as follows: denaturation stage at 50°C for 2 min followed by 95°C for 2 min, 40 amplification cycles at 95°C for 15 s, and 60°C for 1 min. Melting curves were generated after each run (95°C for 15 s, 60°C for 20 s and increments of 0.3°C until reaching 95°C for 15 s) to compare dissociation characteristics of the PCR products obtained from gut samples and positive control. Each reaction was performed in triplicates in a total volume of 10

μl (0.2 μM of each forward and reverse primer; and 1x SYBR® Select Master Mix, Applied Biosystems) with 1 μl of DNA or cDNA (to assess virus loads). Each plate contained a positive control and a water control.

To determine the absolute quantity of each target in the samples we performed standard curves on serial dilutions of plasmids (pGEM®-T Easy vector; Promega) containing the target sequence. qPCR conducted on genomic DNA from the bee gut or on pure plasmid DNA results in different primer efficiencies (E) (due to the complexity of DNA samples, different methods of extraction, presence of inhibitors, etc.) [11]. Therefore, to assess more realistic primer efficiencies plasmid dilutions were mixed 1:1 with DNA isolated with the above CTAB-based protocol from the gut of a newly emerged microbiota-free bee that was negative for all investigated targets. The final concentrations of the plasmid in these template samples ranged from 10^7 – 10^1 copies per μl. The plasmid copy number was calculated from the molecular weight of the plasmid and the DNA concentration of the purified plasmid measured with Qubit™ (Thermo Fisher). The slope and intercept of standard curves were calculated based on the Cq values obtained from the dilutions 10^7 – 10^2 copies. Frequently, no amplification was obtained at the highest dilution or the amplification came up at a similar Cq as the negative control. As the *limit of detection* (LOD) of a given primer pair, we consider the Cq value of the highest plasmid dilution, at which the target was detected. The E values were estimated from the slopes according to the equation: $E = 10^{(-1/\text{slope})}$ [12]. Primer characteristics and their performance are summarized in **S2 Table**.

The specificity of the primer pairs used to detect different gut bacteria was

tested on genomic DNA isolated from bacterial cultures with the GenElute Bacterial Genomic DNA kit (Sigma). Genomic DNA of all bacterial species was diluted to 15 ng/µl and tested with each primer pair by qPCR. Using non-target genomic DNA as template, amplification was either undetected or resulted in Cq values >30 for all primer pairs.

Analysis of qPCR data and selection of samples for metabolomics analysis.

The MIQE guidelines (minimum information for publication of qPCR experiments) were followed throughout the data analysis of the qPCR experiments [13]. A uniform detection value of fluorescence intensity was set for each target and kept the same across all qPCR plates of the study. Only samples with dissociation curves matching the curves of the positive control were kept. Technical outliers from each triplicate were eliminated and mean *quantification cycle* (Cq) and SD values were calculated. Then, the data was exported from the StepOnePlus qPCR instrument for further processing in R. We only considered data from plates for which no signal in the negative control was detected or for which the Cq value of the negative control was higher than the highest dilution (10 copies) included in the standard curve. All samples for which the Cq value of actin was >24 were excluded from the analysis, as the extracted DNA was considered to be not of sufficient quality. For each DNA sample that passed the initial quality check, we determined the number of bacterial cells per gut as follows. We first calculated the 'raw' copy number of each target in 1 µl of DNA from the Cq value and the standard curve using the formula $n = E^{(\text{intercept} - \text{Cq})}$ [14]. Then, we normalized the bacterial 16S rRNA gene copies to the median

number of actin gene copies by dividing by the 'raw' copy number of actin for the given sample and multiplying by the median number of actin gene copies across all samples. Normalization with the actin gene was carried out to reduce the effect of gut size variation and extraction efficiency. To infer the number of bacteria from the normalized 16S rRNA gene copy number we divided by the number of rRNA loci present in the genome of the given species (as listed in **S1 Table**) and multiplied by 200 as we only analyzed 1 µl of the 200 µl of DNA obtained from each sample.

We used the qPCR analysis to check the gnotobiotic status of the colonized bees and to exclude bees that were colonized by other gut bacteria than those we exposed them to. Bees for which signals of undesired bacteria were above 500 copies per reaction (i.e. 10^5 cells per gut) were removed from further analysis. An exception had to be made for some bees from the *B. apis* mono-colonization condition (see **S5 Fig**), for which qPCR revealed the presence of Firm-5 bacteria slightly above this threshold. However, these signals were significantly lower than for the CL condition, suggesting that Firm-5 was present at relatively low abundance. We also included a few other samples in our analysis for which colonization levels of other bacteria were marginally above the threshold of 500 copies per reaction (i.e. 10^5 cells per gut). We did so to have a sufficient number of samples for these two conditions (see **S5 Fig**). Based on the metabolomics analysis and the recapitulation of our findings *in vitro*, we feel confident that these possible contaminants had little if no effect on our results.

To exclude that MD samples were heavily colonized with other non-specific bacteria from the environment, we additionally screened MD, CL and hive samples

with universal bacterial 16S rRNA primers. The total number of 16S rRNA gene copies was divided by 4, which is the number of rRNA loci carried by most bee gut bacteria in their genome, to estimate the total number of bacteria in the gut (**S1A Fig**).

We also screened cDNA reverse-transcribed from the RNA samples for the presence of viruses, to exclude possible effects of high viral titers on gut metabolism. Varroa destructor virus 1 (VDV-1) was the only detectable virus in our bees (we screened for the viruses DWV, BQPV, ABPV, and CBPV). When the relative abundance of VDV-1 was below 1 compared the reference transcript (*A. mellifera* actin gene) the samples were selected for metabolomics analysis.

References:

1. Holdeman LV, Cato ED, Moore, W. E. C. Anaerobe Laboratory Manual (Blacksburg, Va.: Virginia Polytechnic Institute and State University Anaerobe Laboratory). 1977.
2. Richard F-J, Tarpy DR, Grozinger CM. Effects of Insemination Quantity on Honey Bee Queen Physiology. PLOS ONE. 2007; 2: e980. doi:10.1371/journal.pone.0000980.
3. Fuhrer T, Heer D, Begemann B, Zamboni N. High-throughput, accurate mass metabolome profiling of cellular extracts by flow injection-time-of-flight mass spectrometry. Anal Chem. 2011; 83: 7074–7080. doi:10.1021/ac201267k.

4. Kim S, Thiessen PA, Bolton EE, Chen J, Fu G, Gindulyte A, et al. PubChem Substance and Compound databases. Nucleic Acids Res. 2016; 44: D1202–D1213. doi:10.1093/nar/gkv951.

5. Kanehisa M, Furumichi M, Tanabe M, Sato Y, Morishima K. KEGG: new perspectives on genomes, pathways, diseases and drugs. Nucleic Acids Res. 2017; 45: D353–D361. doi:10.1093/nar/gkw1092.

6. Ruttkies C, Schymanski EL, Wolf S, Hollender J, Neumann S. MetFrag relaunched: incorporating strategies beyond in silico fragmentation. J Cheminformatics. 2016; 8: 3. doi:10.1186/s13321-016-0115-9

7. Stein SE, Scott DR. Optimization and testing of mass spectral library search algorithms for compound identification. J Am Soc Mass Spectrom. 1994; 5: 859–866. doi:10.1016/1044-0305(94)87009-8

8. Dodder NG. OrgMassSpecR: Organic Mass Spectrometry. R package version 0.4-4. https://CRAN.R-project.org/package=OrgMassSpecR. and with code contributions from Katharine M. Mullen; 2014.

9. E.L. Schymanski. RChemMass: Various Cheminformatic and Mass Spectrometry Functions. R package version 0.1.8. Eawag, Dübendorf, Switzerland. Code available upon request.; 2017.

10. Schymanski EL. ReSOLUTION: SOLUTIONS for High ReSOLUTION Mass Spectrometry. R package version 0.1.3. Eawag, Dübendorf, Switzerland. Code available upon request.; 2017.

11. Brankatschk R, Bodenhausen N, Zeyer J, Bürgmann H. Simple absolute quantification method correcting for quantitative PCR efficiency variations for

microbial community samples. Appl Environ Microbiol. 2012; 78:4481-9. doi: 10.1128/AEM.07878-11.

12. Pfaffl MW. A new mathematical model for relative quantification in real-time RT-PCR. Nucleic Acids Res. 2001. 29:e45.

13. Bustin SA, Benes V, Garson JA, Hellemans J, Huggett J, Kubista M, Mueller R, Nolan T, Pfaffl MW, Shipley GL, Vandesompele J, Wittwer CT. The MIQE guidelines: minimum information for publication of quantitative real-time PCR experiments. Clin Chem. 2009; 55:611-22. doi: 10.1373/clinchem.2008.112797.

15. Gallup JM. qPCR Inhibition and Amplification of Difficult Templates. PCR Troubleshooting and Optimization: The Essential Guide. Horizon Scientific Press; 2011. pp. 23–65.
